# Integration of 117 machine learning algorithms and single-cell transcriptomics identifies macrophage polarization and ER stress signatures for cancer prognosis and precision therapy

**DOI:** 10.1007/s12672-026-05126-6

**Published:** 2026-04-30

**Authors:** Shengrong Long, Kewei Xiao, Zhipeng Hao

**Affiliations:** Department of Thoracic Surgery, Tongji Hospital, Tongji Medical College, Huazhong University of Science and Technology, Wuhan, China

**Keywords:** Macrophage polarization, Endoplasmic reticulum stress, Machine learning, Single-cell transcriptomics, Lung adenocarcinoma, Prognostic model

## Abstract

**Background:**

Macrophage polarization and endoplasmic reticulum (ER) stress play critical yet incompletely understood roles in cancer progression and therapeutic resistance.

**Methods:**

Here, we conduct a systematic pan-cancer analysis of macrophage polarization and ER stress-related genes (MPERSRGs) by integrating multi-omics data from The Cancer Genome Atlas (TCGA), Genotype-Tissue Expression (GTEx), Cancer Cell Line Encyclopedia (CCLE), and single-cell RNA sequencing datasets across 33 cancer types.

**Results:**

We identify distinct expression patterns of seven core MPERSRGs (CEBPB, NUPR1, ATF3, CASP3, TNFSF10, BRSK2, NOD2) that correlate significantly with tumor stage, immune infiltration, and patient prognosis. Employing 117 machine learning algorithm combinations, we develop a robust five-gene prognostic signature (FAM83A, RHOV, CPS1, STRIP2, SLC2A1) for lung adenocarcinoma (LUAD) with area under the curve values of 0.692, 0.688, and 0.614 for 1-, 3-, and 5-year overall survival, respectively. Single-cell transcriptomic analysis of 86,378 cells reveals three functionally distinct fibroblast subpopulations (MFAP5+, MATK+, HP+) with differential MPERSRG expression profiles, with MFAP5 + fibroblasts showing the highest enrichment in epithelial-mesenchymal transition and angiogenesis pathways. Cell–cell communication analysis identifies fibroblasts and epithelial cells as the most interactive populations, with the CLEC2C-KLRB1 ligand-receptor pair mediating the strongest signaling between mast cells and NK cells. Drug sensitivity predictions across multiple databases identify vorinostat, nilotinib, olaparib, and paclitaxel as potential therapeutic agents showing differential efficacy based on MPERSRG expression stratification.

**Conclusions:**

These findings establish MPERSRGs as key determinants of tumor-immune interactions and provide actionable biomarkers for risk stratification and precision therapy selection in cancer.

**Supplementary Information:**

The online version contains supplementary material available at 10.1007/s12672-026-05126-6.

## Introduction

Cancer remains a leading cause of mortality worldwide, with lung adenocarcinoma (LUAD) being one of the most prevalent and lethal malignancies [1, 2]. Despite significant advances in surgical interventions, radiotherapy, chemotherapy, and immunotherapy, the heterogeneity of tumors and the emergence of drug resistance continue to limit therapeutic efficacy [3, 4]. The tumor microenvironment (TME), particularly the polarization states of macrophages and endoplasmic reticulum (ER) stress responses, has emerged as a critical determinant in cancer initiation, progression, metastasis, and immune evasion [5–7]. Understanding these mechanisms is essential for developing precision diagnostic and therapeutic strategies.

Macrophages are key immune effector cells within the TME that exhibit remarkable plasticity, capable of polarizing into distinct functional phenotypes. M1 macrophages (classically activated) promote anti-tumor immunity through pro-inflammatory cytokine secretion and tumor cell phagocytosis, whereas M2 macrophages (alternatively activated) facilitate tumor progression by secreting immunosuppressive factors, promoting angiogenesis, and tissue remodeling [8–10]. Recent studies have demonstrated that the balance between M1 and M2 polarization significantly influences cancer prognosis and therapeutic response [11, 12]. However, the molecular mechanisms governing macrophage polarization in different cancer types remain incompletely understood.

Endoplasmic reticulum stress, triggered by the accumulation of misfolded proteins, activates the unfolded protein response (UPR), a complex signaling network involving three major pathways: IRE1α, PERK, and ATF6 [13, 14]. In the tumor context, ER stress plays a paradoxical role: it can promote cell survival under metabolic stress or induce apoptosis when stress is overwhelming [15, 16]. Accumulating evidence suggests that ER stress modulates the immune landscape of tumors by affecting immune cell function, antigen presentation, and cytokine production [17, 18]. Furthermore, ER stress has been implicated in resistance to chemotherapy and targeted therapies [19, 20].

Emerging evidence indicates a complex interplay between macrophage polarization and ER stress in the TME. ER stress can influence macrophage polarization by modulating metabolic reprogramming and inflammatory signaling pathways [21, 22]. Conversely, polarized macrophages can affect ER stress responses in tumor cells through secreted factors and direct cell–cell interactions [23]. Despite these insights, a comprehensive pan-cancer analysis integrating macrophage polarization and ER stress-related genes (MPERSRGs) has not been conducted, limiting our understanding of their coordinated role across different cancer types.

While previous studies have examined macrophage polarization and ER stress independently, systematic investigations of their synergistic effects across multiple cancer types are lacking. Most existing research has focused on single cancer types or individual genes, failing to capture the complexity of these interconnected processes [24, 25]. The advent of large-scale genomic databases and single-cell sequencing technologies has provided unprecedented opportunities for comprehensive multi-omics analysis [26, 27]. However, integrative approaches combining bulk and single-cell transcriptomics with machine learning algorithms for risk stratification and therapeutic prediction remain underutilized.

In this study, we performed a comprehensive pan-cancer analysis of MPERSRGs by integrating data from The Cancer Genome Atlas (TCGA), Genotype-Tissue Expression (GTEx), Cancer Cell Line Encyclopedia (CCLE), and single-cell RNA sequencing datasets. Our multi-faceted approach included differential expression analysis, unsupervised clustering, pathway enrichment analysis, immune infiltration assessment, prognostic modeling using machine learning algorithms, single-cell transcriptomics, cell–cell communication analysis, and drug sensitivity prediction. We aimed to: (1) characterize the expression patterns of MPERSRGs across 33 cancer types; (2) elucidate their associations with clinical features and immune microenvironment; (3) develop a robust prognostic model for LUAD; (4) identify cellular subpopulations with distinct MPERSRGs expression profiles; and (5) discover potential therapeutic compounds targeting MPERSRGs pathways.

Our findings provide novel insights into the molecular mechanisms underlying cancer progression and immune regulation, offering potential biomarkers for risk stratification and therapeutic targets for precision oncology. By integrating macrophage polarization and ER stress biology, this study bridges critical gaps in our understanding of tumor-immune interactions and drug resistance mechanisms [28, 29]. The identification of key MPERSRGs and their associated cellular networks may facilitate the development of combination therapies targeting both tumor cells and the immunosuppressive microenvironment [30].

## Materials and methods

### Data acquisition

Transcriptome data from cancer cohorts were downloaded from TCGA database using the R package TCGAbiolinks, with corresponding clinical data obtained through the UCSC Xena database. Both count and TPM data for all 33 cancer types were acquired, and GTEx data were integrated for cancer types lacking adequate control samples (Table S1).

LUAD validation cohorts GSE19188 (82 samples) and GSE31210 (226 samples) were downloaded from the GEO database; only samples with prognostic information were retained (Table 1). To ensure data consistency and comparability, the raw data from the independent validation datasets (GSE19188 and GSE31210) were systematically preprocessed prior to analysis. Utilizing the *limma* package (Version 3.58.1) in R, the preprocessing pipeline included standard probe annotation, log2 transformation of the raw gene expression values, and subsequent quantile normalization.

**Table 1:** GEO Microarray chip information

	GSE31210	GSE19188	GSE131907
Platform	GPL570	GPL570	GPL16791
Species	Homo sapiens	Homo sapiens	Homo sapiens
Tissue	Lung	Lung	Lung
Samples in LUAD group	226	82	22
Reference	PMID: 22,080,568 PMID: 23,028,479	PMID: 20,421,987	PMID: 32,385,277 PMID: 40,517,220

*GEO* gene expression omnibus, *LUAD* lung adenocarcinoma

MPERSRGs were identified by intersecting macrophage polarization-related genes (MPRGs) and endoplasmic reticulum stress-related genes (ERSRGs) collected from the GeneCards database and PubMed, with TCGA and GEO datasets. This process yielded seven MPERSRGs: *CEBPB, NUPR1, ATF3, CASP3, TNFSF10, BRSK2*, and *NOD2* (Tables S2–S3). The CCLE cell line dataset (946 human cancer cell lines) was additionally downloaded to examine MPERSRGs expression patterns across cancer types.

### Expression patterns of MPERSRGs in tumor cells

Immunofluorescence staining images of MPERSRGs in four tumor cell lines were retrieved from the Human Protein Atlas (HPA) database to analyze intracellular protein localization.

Based on the CCLE database, 1,595 tumor cell lines were categorized into 26 cancer types. Consensus clustering (R package ConsensusClusterPlus) was applied to the MPERSRGs mRNA expression matrix, classifying cell lines into two distinct subgroups (see Supplementary Methods for parameters). Heatmaps illustrated expression differences and subgroup distributions across 26 organ-specific cancer types.

TCGA RNA-seq data were integrated with GTEx normal tissue data, and Wilcoxon rank-sum tests were used to analyze differential MPERSRGs expression across 30 cancer types. CPTAC LUAD proteome data from the UALCAN platform validated protein-level expression differences between tumor and normal tissues.

### Enrichment of macrophage polarization and endoplasmic reticulum stress-related genes in tumor progression and pathways

Samples were stratified by T, N, M and pathological stage to explore MPERSRGs expression differences across clinical subgroups. Single-gene GSEA was performed for each MPERSRG to identify enriched pathways in high-expression samples, using the c2 gene set from MSigDB (see Supplementary Methods for parameters). ESTIMATE immune and stromal scores for pan-cancer transcriptomes were calculated using the R package IOBR, and Spearman correlations between MPERSRGs expression and ESTIMATE scores across 33 cancers were visualized in heatmaps.

GSVA was performed using the H hallmark gene set from MSigDB to obtain pathway scores. Three hallmark pathways significantly correlated with MPERSRGs were selected: HALLMARK_INFLAMMATORY_RESPONSE, HALLMARK_INTERFERON_GAMMA_RESPONSE, and HALLMARK_TNFA_SIGNALING_VIA_NFKB. Spearman correlations between MPERSRGs expression and these pathway scores were visualized in heatmaps.

### Construction of prognostic model for lung adenocarcinoma

Genome-wide univariate Cox regression was performed on the TCGA-LUAD dataset to screen prognostic genes, followed by Spearman correlation analysis to identify MPERSRGs-correlated genes for machine learning. Genes with correlation coefficients (cor) > 0.3 were selected for machine learning analysis. Statistically, 0.3 serves as an empirical threshold for moderate correlation; it ensures a sufficient strength of association with the core MPERSRGs while retaining an adequate number of candidate genes (n = 168) for subsequent machine learning-based feature selection. This avoids restricting the model's exploratory space with an overly strict threshold or introducing excessive noise with an overly lenient one. Biologically, this threshold helps guarantee that the candidate genes are functionally associated with the macrophage polarization and endoplasmic reticulum stress pathways. Simultaneously, it allows the data-driven machine learning algorithms to autonomously identify the optimal gene combination possessing independent prognostic value in LUAD from a rigorously defined pool of moderately correlated genes.Ten classical algorithms were integrated to generate 117 algorithm combinations, with TCGA-LUAD as the training cohort and GSE19188/GSE31210 as validation cohorts. The optimal model was selected by C-index performance across all cohorts; 'StepCox[forward] + RSF' was identified as the best model (Supplementary Table S8). The top 5 genes by importance were designated as Model Genes, and a RiskScore was calculated using multivariate Cox regression. Model performance was evaluated using Kaplan–Meier survival curves, time-dependent ROC curves (1-, 3-, and 5-year AUC), calibration curves, and Decision Curve Analysis (DCA). A nomogram was constructed to visualize the prognostic weight of each Model Gene.

This design was intended to comprehensively evaluate the effects of different algorithmic features on predictive performance, aiming to avoid the inherent biases associated with single-algorithm approaches [31]. Model combinations exhibiting robust performance across datasets were selected based on the concordance index (C-index) evaluated in the training cohort (TCGA-LUAD) and two independent validation cohorts (GSE19188, GSE31210). Drawing upon analytical frameworks from recent prognostic biomarker studies, this multi-algorithm comparative strategy ultimately improves the reliability and generalizability of the established model.

### Quality control of single-cell dataset

The LUAD single-cell dataset GSE131907 (22 samples) was processed using the R package Seurat. Low-quality cells were removed based on standard quality control thresholds (see Supplementary Methods), followed by normalization, identification of hypervariable genes, dimensionality reduction (PCA and UMAP), and unsupervised clustering. Cell type annotation was performed using SingleR with the BlueprintEncodeData reference dataset and marker genes. AUCell scoring identified cell populations with high MPERSRGs activity. Fibroblast subpopulations were re-clustered and characterized by differential gene expression. Cell–cell communication was inferred using CellChat, and copy number variations (CNVs) in epithelial cells were assessed using inferCNV, with lymphocytes and myeloid cells serving as reference populations (see Supplementary Methods for detailed parameters).

### Construction of regulatory networks

MPERSRGs-related miRNAs, transcription factors (TFs), and drug interactions were retrieved from the TarBase, ChIPBase, and Comparative Toxicogenomics databases, respectively. Networks were visualized using Cytoscape software. The mRNA-miRNA, mRNA-drug, and mRNA-TF regulatory networks for MPERSRGs were constructed using data retrieved from the TarBase, CTD, and ChIPBase databases, respectively. To ensure the reliability and biological relevance of the networks, strict evidence-based inclusion criteria were applied rather than arbitrary predictive score thresholds. Specifically, for the mRNA-miRNA network (TarBase), only experimentally validated interaction pairs supported by robust physical evidence (e.g., reporter assays, Western blotting, CLIP-seq) were included. For the mRNA-drug network (CTD), only compounds exhibiting direct mechanistic interactions with MPERSRGs (designated as "M" in the database) were selected, while indirect or purely predicted associations were strictly excluded. For the mRNA-TF network (ChIPBase), transcription factor binding sites were selected solely based on experimental validation (e.g., ChIP-seq binding events) to maintain high-confidence regulatory relationships. By relying exclusively on database-curated experimental evidence without applying subjective artificial thresholds, we minimized selection bias and ensured the accuracy and reproducibility of the constructed networks.

### Prediction of drugs targeting genes involved in macrophage polarization and endoplasmic reticulum stress

Consensus clustering divided TCGA samples into two subgroups (Cluster1 and Cluster2). Differential analysis (R package limma) identified six cancer types with significant MPERSRGs stratification: BLCA, SARC, BRCA, OV, STAD, and LUAD. Potential therapeutic compounds were identified using the CMap database, and drug sensitivity (IC50) was predicted using the oncoPredict package based on GDSC data. Intersections between CMap- and oncoPredict-predicted compounds were analyzed using Venn diagrams.

### Association of macrophage polarization and endoplasmic reticulum stress-related genes with immune characteristics

Immune cell infiltration abundance was quantified by ssGSEA across 33 cancer datasets, and Spearman correlations with MPERSRGs expression were visualized in heatmaps. TIDE immunoscores were obtained from the TIDE web platform, and IPS scores for 20 cancers were downloaded from the TCIA website; differences between high and low MPERSRGs expression groups were assessed by Wilcoxon rank-sum tests. ESTIMATE and TIP analyses were additionally performed to characterize the tumor immune microenvironment.

### Statistical analysis

All analyses were performed in R. Two-group comparisons used Student's t-test (normal distribution) or Wilcoxon rank-sum test; multi-group comparisons used Kruskal–Wallis test. Spearman correlation was applied for molecular association analyses. All *p*-values were two-sided; *p* < 0.05 was considered statistically significant.

## Results

### Technology roadmap

Figure S1 Flow Chart of the Comprehensive Analysis.

### Expression patterns of MPERSRGs in tumor cells

Immunofluorescence scanning images of MPERSRGs in four tumor cell lines (A-431 skin cancer, SK-MEL-30 skin cancer, U2OS bone cancer, U-251MG brain cancer) were obtained from the HPA database. Colocalization features of target proteins (green), tubulin (red), and nuclei (blue) were analyzed (Figure S2A).

Unsupervised clustering analysis of the MPERSRG mRNA expression matrix from CCLE was performed yielding two subgroups(Figure S3). Heatmaps illustrated MPERSRG expression in cell lines across 26 cancer types (Figure S2B, left) and the proportions of different cluster subgroups in cell lines across 26 organ-specific cancer types (Figure S2B, right).

MPERSRG expression differences between tumor and control samples across 30 cancer types (11 from TCGA; 19 from TCGA-GTEx combined) were and visualized in heatmaps (Figure S2C).The UALCAN database validated protein expression levels of MPERSRGs in lung adenocarcinoma (LUAD) (Figure S2D).

### Enrichment of macrophage polarization and endoplasmic reticulum stress-related genes in tumor progression and pathways

**Fig. 1:**
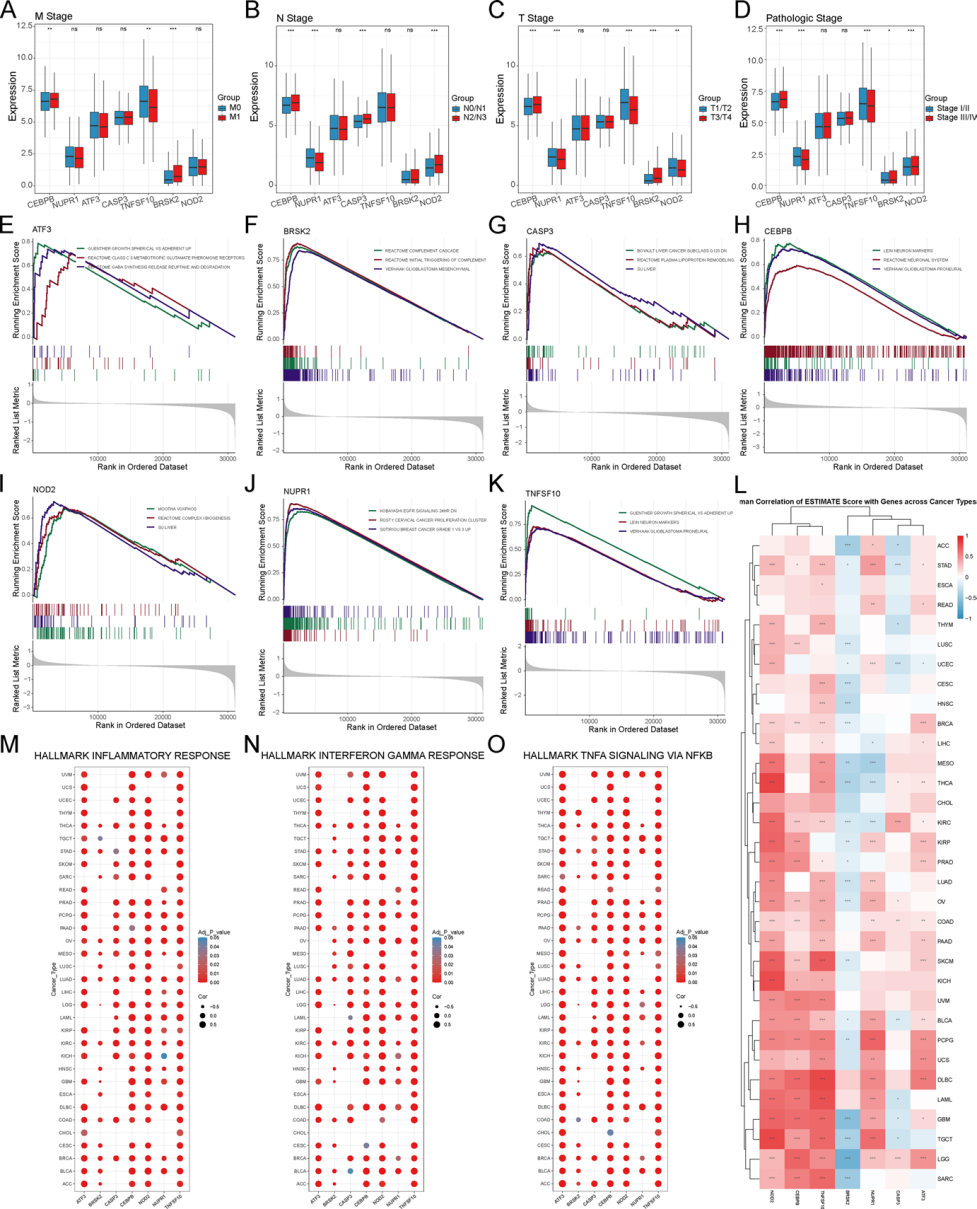
Correlation Analysis Between Enrichment and Immune Infiltration of MPERSRGs. **A**–**D** Violin plots comparing MPERSRG expression based on T, N, M stages and pathological stages. **E**–**K** GSEA analysis of MPERSRGs in pan-cancer, displaying the top 3 positively correlated pathways. **L** Correlation between MPERSRG expression and ESTIMATE scores across 33 cancers. **M**–**O** Correlations between MPERSRG expression and GSVA enrichment scores for HALLMARK_INFLAMMATORY_RESPONSE, HALLMARK_INTERFERON_GAMMA_RESPONSE, and HALLMARK_TNFA_SIGNALING_VIA_NFKB pathways. The color gradient (red to blue) represents the adjusted *p*-value, with deeper red indicating a smaller *p*-value and higher statistical significance. The size of the points corresponds to the absolute value of the correlation coefficient, reflecting the strength of the correlation. ns: *p* ≥ 0.05; *p < 0.05; **p < 0.01; ***p < 0.001

Group comparison plots (Fig. 1A–D) illustrate differential expression analysis results of the seven MPERSRGs across different clinical groups stratified by T, N, M stages and pathological stages.

GSEA identified the top three predominantly upregulated pathways associated with each MPERSRG (detailed in Table 2). Specifically, these enriched pathways included: GUENTHER_GROWTH_SPHERICAL_VS_ADHERENT_UP, REACTOME_CLASS_C_3_METABOTROPIC_GLUTAMATE_PHEROMONE_RECEPTORS, and REACTOME_GABA_SYNTHESIS_RELEASE_REUPTAKE_AND_DEGRADATION for **ATF3** (Fig. 1E); REACTOME_COMPLEMENT_CASCADE, REACTOME_INITIAL_TRIGGERING_OF_COMPLEMENT, and VERHAAK_GLIOBLASTOMA_MESENCHYMAL for **BRSK2** (Fig. 1F); BOYAULT_LIVER_CANCER_SUBCLASS_G123_DN, REACTOME_PLASMA_LIPOPROTEIN_REMODELING, and SU_LIVER for **CASP3** (Fig. 1G); LEIN_NEURON_MARKERS, REACTOME_NEURONAL_SYSTEM, and VERHAAK_GLIOBLASTOMA_PRONEURAL for **CEBPB** (Fig. 1H); MOOTHA_OXPHOS, REACTOME_COMPLEX_I_BIOGENESIS, and SU_LIVER for **NOD2** (Fig. 1I); KOBAYASHI_EGFR_SIGNALING_24HR_DN, ROSTY_CERVICAL_CANCER_PROLIFERATION_CLUSTER, and SOTIRIOU_BREAST_CANCER_GRADE_1_VS_3_UP for **NUPR1** (Fig. 1J); and GUENTHER_GROWTH_SPHERICAL_VS_ADHERENT_UP, LEIN_NEURON_MARKERS, and VERHAAK_GLIOBLASTOMA_PRONEURAL for **TNFSF10** (Fig. 1K).

**Table 2:** Results of GSEA for Pan-Cancer PANCAN by MPERSRGs

ID	Set size	Enrichment score	NES	*p* value	*p* adjust	*q* value
GUENTHER GROWTH SPHERICAL VS ADHERENT UP	22	0.786387	2.699801	2.68E−07	1.67E−06	6.31E−07
REACTOME CLASS C 3 METABOTROPIC GLUTAMATE PHEROMONE RECEPTORS	25	0.706248	2.511761	2.02E−06	1.08E−05	4.09E−06
REACTOME GABA SYNTHESIS RELEASE REUPTAKE AND DEGRADATION	17	0.737949	2.263192	9.47E−05	0.00034	0.000128
VERHAAK GLIOBLASTOMA MESENCHYMAL	216	0.839448	4.117208	1.00E−10	2.39E−09	1.41E−09
REACTOME COMPLEMENT CASCADE	107	0.876634	4.018116	1.00E−10	2.39E−09	1.41E−09
REACTOME INITIAL TRIGGERING OF COMPLEMENT	78	0.909196	3.911074	1.00E−10	2.39E−09	1.41E−09
SU LIVER	44	0.694889	2.861535	1.00E−10	1.21E−09	4.48E−10
REACTOME PLASMA LIPOPROTEIN REMODELING	30	0.661317	2.846708	7.28E−07	4.59E−06	1.70E−06
BOYAULT LIVER CANCER SUBCLASS G123 DN	49	0.628948	2.814041	6.80E−10	7.22E−09	2.67E−09
VERHAAK GLIOBLASTOMA PRONEURAL	174	0.729443	2.929195	1.00E−10	1.43E−09	7.43E−10
LEIN NEURON MARKERS	69	0.774932	2.692071	1.00E−10	1.43E−09	7.43E−10
REACTOME NEURONAL SYSTEM	386	0.590606	2.587866	1.00E−10	1.43E−09	7.43E−10
MOOTHA VOXPHOS	87	0.674167	2.883289	1.00E−10	1.25E−09	5.20E−10
SU LIVER	44	0.727975	2.819568	2.70E−10	3.19E−09	1.33E−09
REACTOME COMPLEX I BIOGENESIS	57	0.687834	2.805479	1.00E−10	1.25E−09	5.20E−10
ROSTY CERVICAL CANCER PROLIFERATION CLUSTER	140	0.901476	3.475464	1.00E−10	1.84E−09	1.07E−09
KOBAYASHI EGFR SIGNALING 24HR DN	252	0.826709	3.399357	1.00E−10	1.84E−09	1.07E−09
SOTIRIOU BREAST CANCER GRADE 1 VS 3 UP	152	0.855054	3.314126	1.00E−10	1.84E−09	1.07E−09
VERHAAK GLIOBLASTOMA PRONEURAL	174	0.707437	3.037412	1.00E−10	1.66E−09	7.58E−10
LEIN NEURON MARKERS	69	0.728397	2.824301	1.00E−10	1.66E−09	7.58E−10
GUENTHER GROWTH SPHERICAL VS ADHERENT UP	22	0.934572	2.75057	1.00E−10	1.66E−09	7.58E−10

*GSEA* gene set enrichment analysis, *NES* normalized enrichment score, *Pancancer* Pan-Cancer PANCAN

Spearman correlations between MPERSRG expression and ESTIMATE scores were evaluated across 33 cancer types and visualized in heatmaps (Fig. 1L). Multiple MPERSRGs, particularly CEBPB, ATF3, and TNFSF10, exhibit cancer-type-specific positive correlations with Immune and Stromal scores, reflecting increased immune and stromal cell infiltration within the tumor microenvironment. These heterogeneous patterns suggest that MPERSRG expression contributes to an "inflamed" tumor phenotype shaped by macrophage polarization and endoplasmic reticulum stress, highlighting their potential as indicators for immunotherapy response. Spearman correlations were evaluated between MPERSRG expression and the GSVA enrichment scores of three key pathways associated with macrophage polarization and endoplasmic reticulum stress (adj.*p* < 0.05): HALLMARK_INFLAMMATORY_RESPONSE, HALLMARK_INTERFERON_GAMMA_RESPONSE, and HALLMARK_TNFA_SIGNALING_VIA_NFKB (Fig. 1M-O).

### Construction of prognostic model for lung adenocarcinoma

**Fig. 2:**
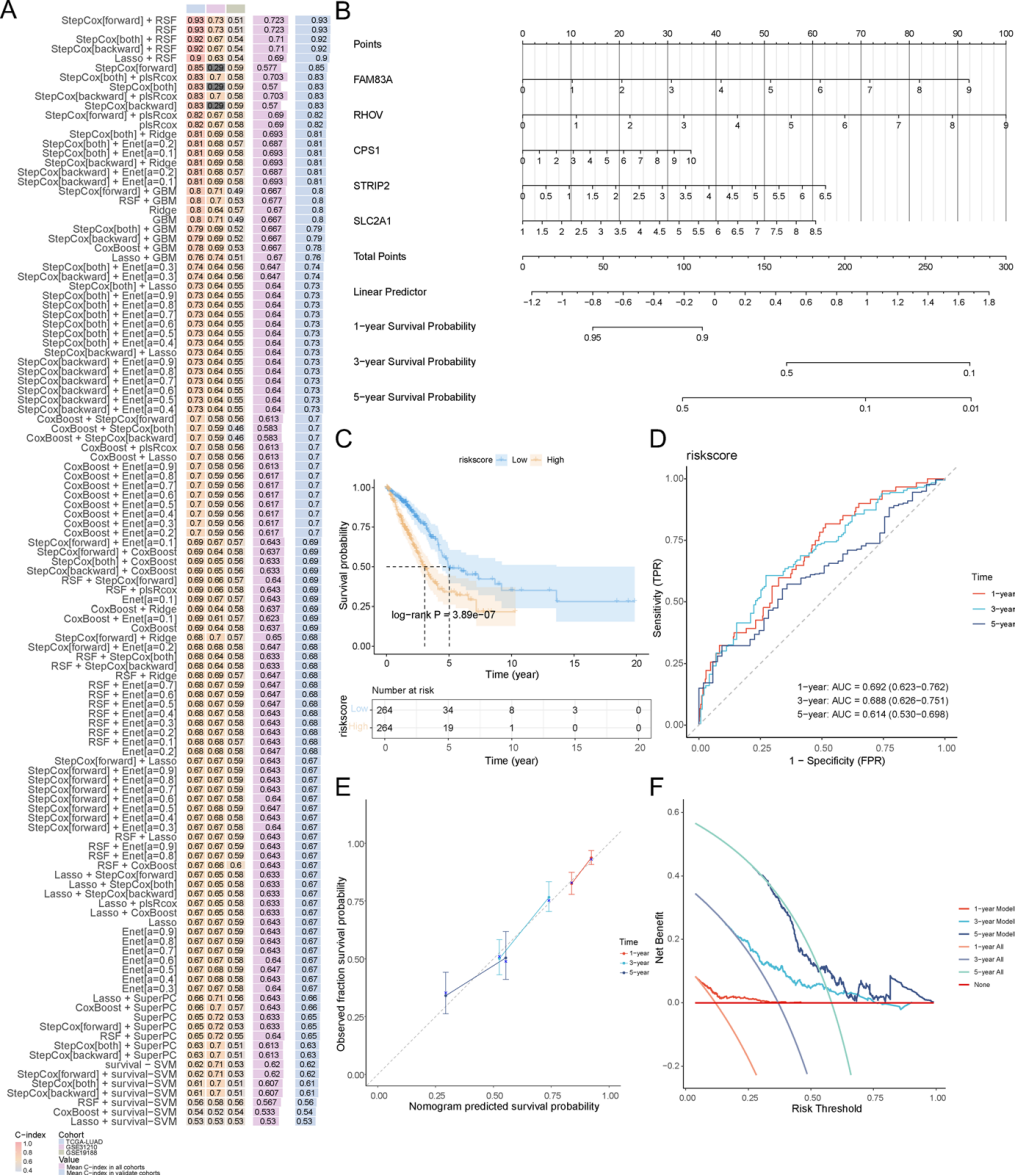
Machine Learning Models Based on MPERSRGs. **A** C-index heatmap of machine learning model combinations based on 168 MPERSRGs in the training set and validation sets GSE19188 and GSE31210. **B** Nomogram of Model Genes with top 5 importance indices calculated in 'StepCox[forward] + RSF'. **C** KM curve for prognostic analysis of high and low risk groups in TCGA-LUAD dataset. **D** Time-dependent ROC curve of TCGA-LUAD dataset. **E** Calibration curves for prognostic risk models at 1, 3, and 5 years. **F** DCA plots of prognostic risk models at 1, 3, and 5 years

Univariate Cox regression and Spearman correlation analyses in the TCGA-LUAD dataset identified 168 prognostic genes significantly correlated with the seven MPERSRGs (Table S4). To construct a robust prognostic model, these candidates were screened using 117 machine learning algorithm combinations (Fig. 2A). The 'StepCox[forward] + RSF' algorithm was selected as the optimal model due to its highest average C-index (0.723). Based on their importance indices within this model, the top five genes (FAM83A, RHOV, CPS1, STRIP2, and SLC2A1) were utilized to calculate risk scores and construct a nomogram (Fig. 2B). LUAD samples from the TCGA-LUAD dataset were then stratified into low-risk and high-risk groups based on median RiskScore values. The RiskScore was calculated using the following formula:

$$ \begin{aligned} {\mathrm{RiskScore}} & = {\mathrm{FAM83A}} \times 0.{1}0{84196} + {\mathrm{RHOV}} \times 0.{11741896} \\ & \quad + {\mathrm{CPS1}} \times 0.0{3681726} + {\mathrm{STRIP2}} \times 0.{1}0{18659} \\ & \quad + {\mathrm{SLC2A1}} \times 0.0{8546539} \\ \end{aligned} $$

Subsequently, KM curves for the TCGA-LUAD dataset were generated (Fig. 2C), demonstrating that patients in the high-risk group exhibited significantly worse prognosis, with highly significant survival differences between high and low risk groups (*p* < 0.001). Time-dependent ROC curves (Fig. 2D) for the TCGA-LUAD dataset were then plotted, revealing that AUC values for 1-year, 3-year, and 5-year OS were 0.692, 0.688, and 0.614, respectively, indicating that the model possessed certain predictive capability.

1-, 3-, and 5-year prognostic calibration analysis was performed on the nomogram of the prognostic model, generating calibration curves (Fig. 2E). Lines and points of different colors represent model-predicted survival probabilities at different time points. The closer the different colored lines are to the gray ideal line, the better the prediction performance at that time point. Results demonstrated that the LUAD prognostic risk model achieved optimal clinical prediction performance at 3 years. Finally, DCA evaluated the predictive performance of the prognostic model at 1, 3, and 5 years, with results presented (Fig. 2F).

### Quality control and cell type annotation of single-cell dataset

**Fig. 3:**
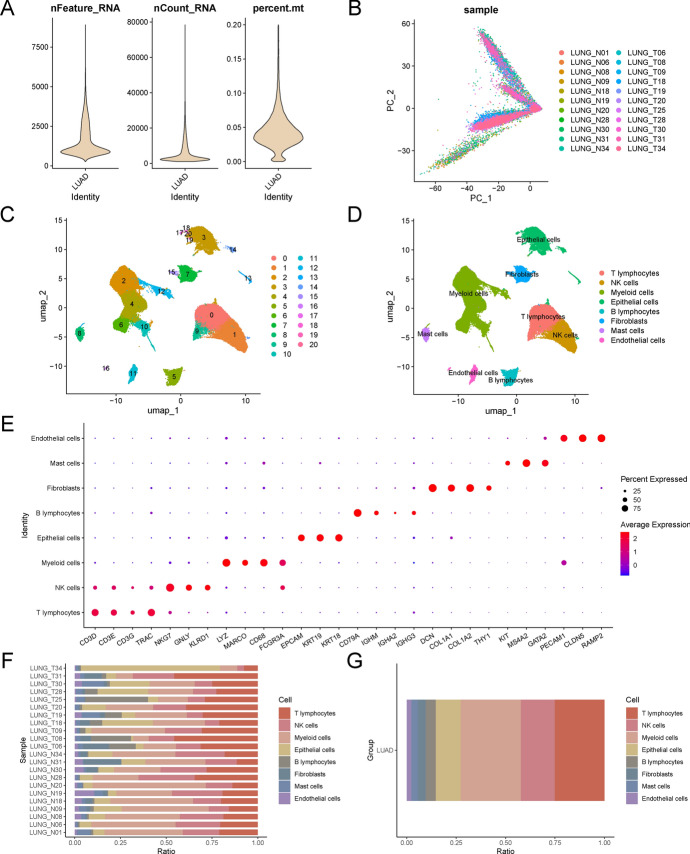
Single-Cell Analysis of Lung Adenocarcinoma. **A** Violin plots showing the distribution of gene expression metrics in the GSE131907 dataset. **B** PCA visualization of cellular expression patterns across different samples. **C** UMAP clustering visualization of 86,378 cells partitioned into 21 distinct clusters. **D** Cell type annotation identifying eight major cell types using SingleR. **E** Bubble plot depicting marker gene expression levels across the eight cell types. **F** Bar plot illustrating cell type proportions across individual samples in GSE131907. **G** Bar plot showing the cellular composition in LUAD samples

Following standard quality control of 22 samples from the single-cell RNA sequencing dataset GSE131907, a total of 86,378 high-quality cells were retained. PCA was utilized to visualize cellular expression patterns across samples (Fig. 3A, B), and subsequent UMAP dimensionality reduction (resolution = 0.3) successfully partitioned these cells into 21 distinct clusters (Fig. 3C).Cell clusters were annotated into eight major cell types (Fig. 3D) using the R package SingleR in combination with established cell marker genes: T lymphocytes, NK cells, myeloid cells, epithelial cells, B lymphocytes, fibroblasts, mast cells, and endothelial cells. A bubble plot was generated to visualize the expression levels of marker genes across the eight cell types within the dataset (Fig. 3E). Bar plots were constructed to display the cellular composition across individual LUAD samples (Fig. 3F) and between sample groups (Fig. 3G), revealing substantial variations in cell type proportions among different samples.

### AUCell analysis

**Fig. 4:**
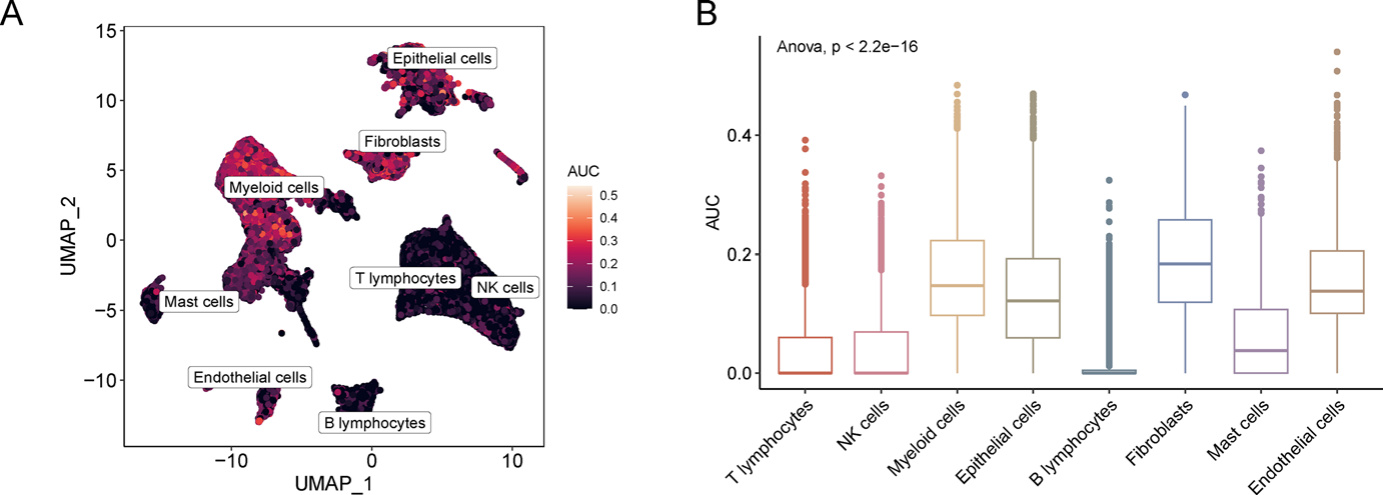
AUCell Analysis of MPERsrg Enrichment Across Cell Types. **A** UMAP projection displaying the distribution of enrichment scores, with color intensity representing score magnitude. **B** Comparative visualization of enrichment scores across annotated cell types

We employed the R package AUCell to calculate enrichment scores for MPERSRGs across individual cells in the GSE131907 dataset. The enrichment scores were visualized using UMAP projections (Fig. 4A) and comparative plots across cell types (Fig. 4B). The analysis revealed that fibroblasts exhibited the highest AUCell enrichment scores among all cell types examined.

### Subpopulation analysis of fibroblasts

**Fig. 5:**
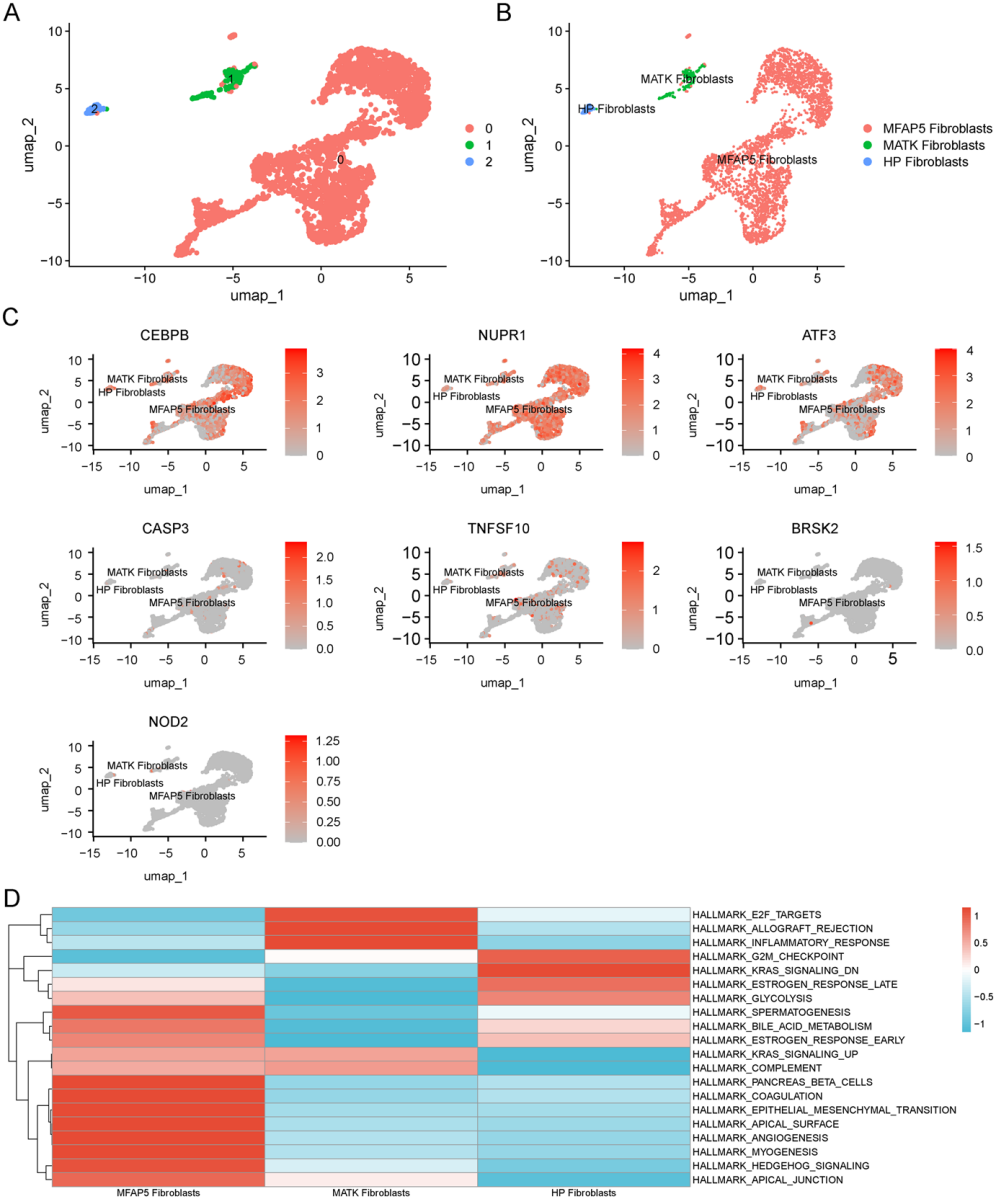
Subtype Analysis of Fibroblasts. **A** UMAP visualization of fibroblast clustering identifying three distinct subpopulations. **B** UMAP projection showing annotated fibroblast subtypes based on marker gene expression. **C** UMAP visualization displaying the expression patterns of MPERSRGs across fibroblast subpopulations. **D** Heatmap of GSVA showing differential pathway enrichment among fibroblast subtypes

To identify novel fibroblast subpopulations, we extracted and re-clustered fibroblasts from the GSE131907 dataset, revealing three distinct subpopulations (Fig. 5A). Differentially expressed genes among these subpopulations were identified using the "FindAllMarkers" function, and the top marker genes were used to annotate each subpopulation (Fig. 5B), resulting in the designation of MFAP5+, MATK+, and HP+ fibroblast subtypes. To determine whether MPERSRGs exhibited differential expression patterns across fibroblast subpopulations, we visualized their expression using UMAP projections (Fig. 5C).

The MFAP5 + fibroblast subpopulation exhibits high expression of the extracellular matrix-related gene MFAP5, suggesting it may belong to the matrix cancer-associated fibroblast (mCAF) subtype, which is primarily involved in extracellular matrix remodeling. Regarding MPERSRG expression, this subpopulation shows significantly high expression of CEBPB, NUPR1, ATF3, CASP3, and TNFSF10 (Fig. 5C). This indicates that it may regulate endoplasmic reticulum stress (ATF3, NUPR1) and apoptosis (CASP3) pathways, while simultaneously participating in the regulation of macrophage polarization (TNFSF10, CEBPB).

The MATK + fibroblast subpopulation specifically expresses MATK, a gene associated with cell signal transduction and immune regulation, indicating that this subpopulation may represent immunomodulatory cancer-associated fibroblasts (iCAFs). The MPERSRG expression pattern in this subpopulation is similar to that of the MFAP5 + subpopulation, characterized mainly by the high expression of CEBPB, NUPR1, ATF3, and TNFSF10. However, the relatively lower expression level of CASP3 implies that its function in apoptosis regulation might be attenuated.

The HP + fibroblast subpopulation is characterized by the specific high expression of HP, a gene primarily involved in the inflammatory response and hemoglobin clearance, suggesting this subpopulation may consist of inflammatory-associated fibroblasts. In terms of MPERSRG expression, this group predominantly expresses high levels of CEBPB, NUPR1, and ATF3, whereas the expression of CASP3 and TNFSF10 is lower. This demonstrates that while its endoplasmic reticulum stress response may remain relatively active, its capacity for regulating macrophage polarization and inducing apoptosis is comparatively weakened.

Collectively, these results indicate that the three fibroblast subpopulations exhibit a gradient of differences in their MPERSRG expression profiles. The MFAP5 + subpopulation displays the most comprehensive profile, while the HP + subpopulation presents a more restricted one. This heterogeneity suggests that distinct fibroblast subpopulations may exert non-redundant functions within the tumor microenvironment through differential levels of endoplasmic reticulum stress and varying capacities to regulate macrophage polarization.

To investigate pathway-level differences among the three fibroblast subpopulations, we performed GSVA using the Hallmark gene set collection (h.all.v2025.1.Hs.symbols.gmt) on the fibroblast cluster. The resulting heatmap (Fig. 5D) revealed distinct pathway enrichment profiles for each subpopulation. MFAP5 + fibroblasts exhibited significant enrichment (logFC > 0, *p* < 0.05) in pathways including estrogen response (early and late), glycolysis, spermatogenesis, bile acid metabolism, KRAS signaling (upregulated), complement activation, pancreatic beta cell function, coagulation, epithelial-mesenchymal transition, apical surface organization, angiogenesis, myogenesis, Hedgehog signaling, and apical junction assembly. MATK + fibroblasts demonstrated enrichment in E2F targets, allograft rejection, inflammatory response, G2/M checkpoint, KRAS signaling (upregulated), complement activation, and apical junction pathways. HP + fibroblasts showed enrichment in G2/M checkpoint, KRAS signaling (downregulated), estrogen response (early and late), glycolysis, bile acid metabolism, and related pathways.

### Cell communication analysis in lung adenocarcinoma

**Fig. 6:**
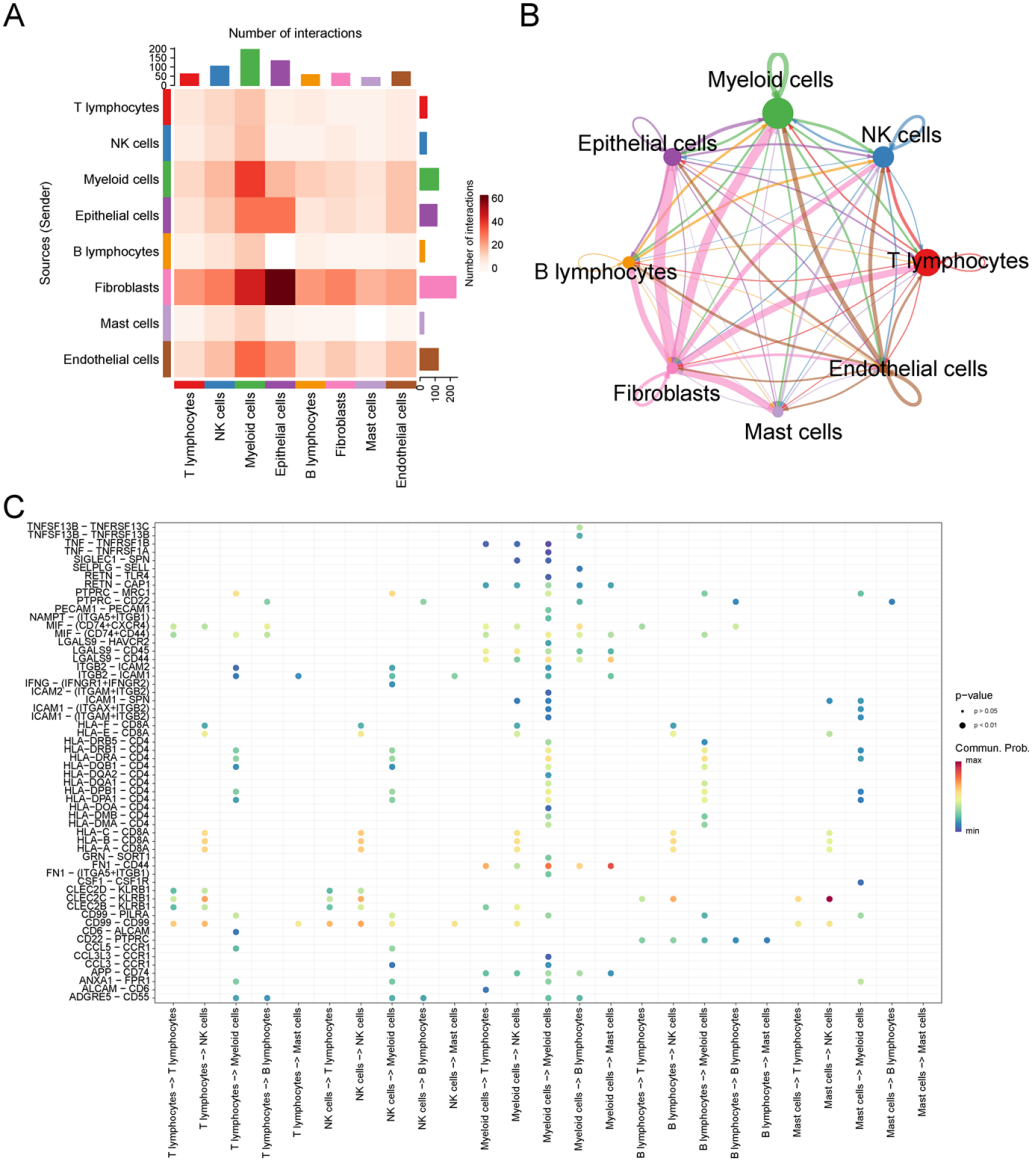
Cell Communication Network Analysis. **A** Heatmap depicting the number of ligand-receptor interaction pairs among the eight cell types. Color intensity (red) represents the abundance of interacting pairs. **B** Circular network plot illustrating intercellular communication strength among cell types. **C** Dot plot of significant ligand-receptor pairs mediating intercellular communication within immune cell populations

The number of interactions (Fig. 6A) and communication strength (Fig. 6B) were visualized using heatmaps and circular plots, respectively. The analysis revealed that fibroblasts and epithelial cells exhibited the most extensive intercellular interactions. We further identified significant ligand-receptor pairs mediating communication within the five immune cell populations (Fig. 6C). Notably, the *CLEC2C*–*KLRB1* ligand-receptor pair demonstrated the highest signaling intensity in communications between mast cells and NK cells.

### inferCNV analysis of cell subpopulations

To identify malignant populations, epithelial cell clusters (clusters 3, 13, 14, 17, 18, 19, and 20) were evaluated using NK cells as a reference baseline. The spatial distribution and computed CNV scores of these subpopulations were visualized using UMAP projections (Figure S4A–D). Comparative analysis revealed that CNV scores across all examined epithelial clusters were significantly elevated compared to the NK cell baseline (predominantly *p* < 0.01; Figure S4E). Consistent with the CNV heatmap (Figure S5), these distinct chromosomal aberrations confirmed that all identified epithelial populations represent malignant tumor cells.

### Construction of regulatory networks

We retrieved miRNAs targeting MPERSRGs from the TarBase database and constructed an mRNA-miRNA regulatory network (Figure S6A). This network comprised 3 MPERSRGs and 45 miRNAs (detailed information in Table S5). Then, we identified potential therapeutic compounds associated with MPERSRGs using the Comparative Toxicogenomics Database (CTD). An mRNA-drug regulatory network was constructed and visualized using Cytoscape software (Figure S6B), encompassing 3 MPERSRGs and 31 drugs (detailed information in Table S6). Third, we identified TFs predicted to regulate MPERSRGs by querying the ChIPBase database. The resulting mRNA-TF regulatory network was visualized using Cytoscape (Figure S6C) and included 4 MPERSRGs and 39 TFs (detailed information in Table S7).

### Drug prediction targeting MPERSRGs

**Fig. 7:**
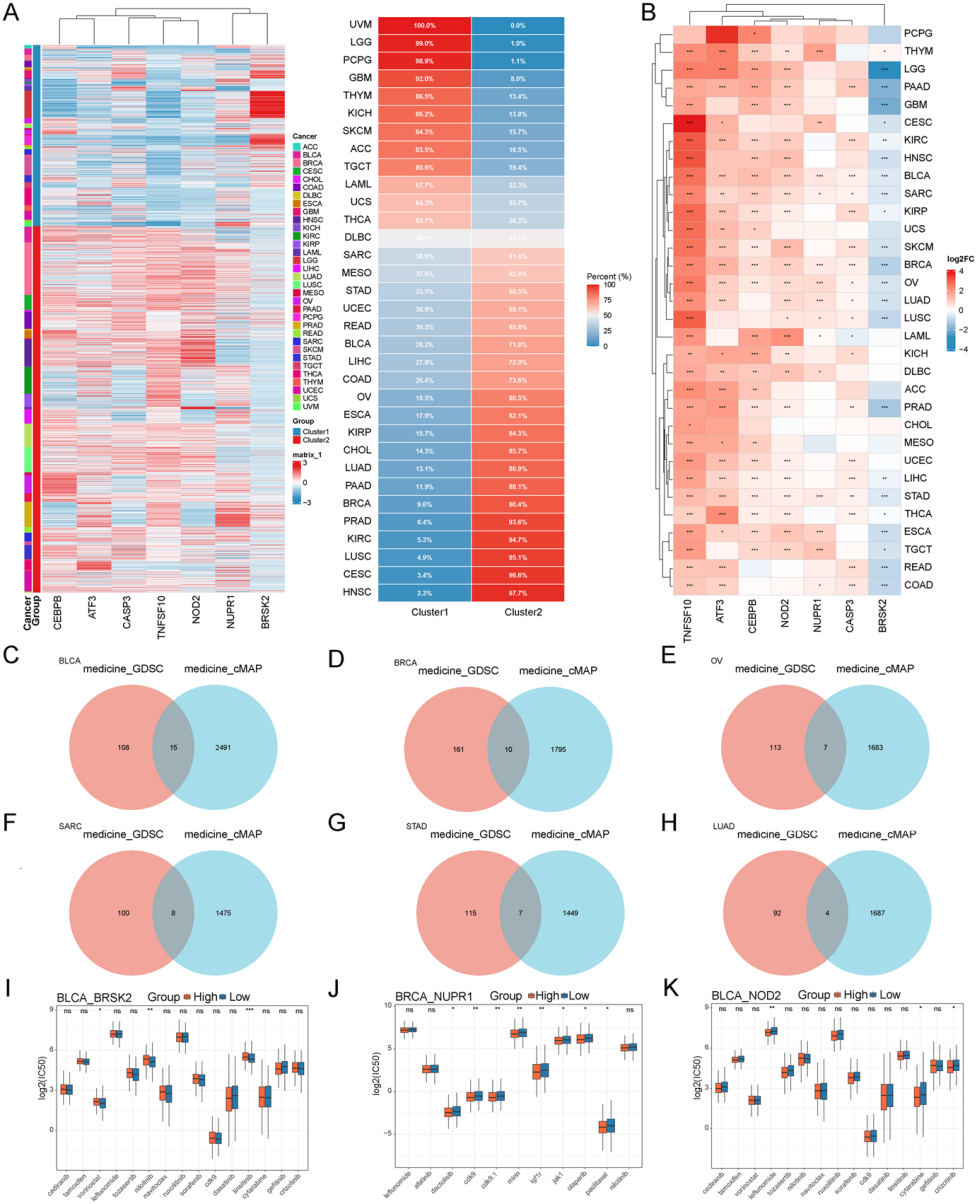
Pan-Cancer Drug Sensitivity Analysis Based on MPERSRGs. **A** Left: Heatmap displaying mRNA expression patterns of MPERSRGs across tumor samples from 33 cancer types in the TCGA dataset. Unsupervised clustering identified two distinct patient subgroups (Cluster 1, blue; Cluster 2, red), indicated by row annotations. **B** Heatmap depicting differential expression (log2 fold change) of MPERSRGs between Cluster 1 and Cluster 2 across 32 TCGA cancer types. **C**–**H** Venn diagrams illustrating overlapping drugs identified by both CMap and GDSC analyses for six cancer types. **I**–**K** Box plots comparing IC50 values of overlapping drugs between patient subgroups stratified by MPERSRG expression levels across the six cancer types. ns: *p* ≥ 0.05; *p < 0.05; **p < 0.01; ***p < 0.001

Unsupervised clustering analysis was performed on the MPERSRGs mRNA expression matrix using K-means clustering algorithm with Pearson correlation distance (clusterAlg = "km", distance = "pearson"), yielding two distinct subgroups (Cluster 1 and Cluster 2; detailed results in Figure S7). A heatmap was generated to visualize MPERSRG expression patterns across cell lines from 33 cancer types (Fig. 7A, left panel), alongside the distribution of clustering subgroups across 26 organ-specific cancer types (Fig. 7A, right panel).

Differential expression analysis of MPERSRGs between Cluster 1 and Cluster 2 was conducted across 33 cancer types using the limma package, with log2FC values displayed in a heatmap to quantify expression alterations (Fig. 7B). Drug sensitivity data for 198 compounds were retrieved from the CMap database, and heatmaps were constructed to display drug sensitivity scores across different clusters (Figure S8) and cancer types (Figure S9).

We employed the "oncoPredict" R package with ridge regression to predict anticancer drug sensitivity in TCGA patient cohorts, using the Genomics of Drug Sensitivity in Cancer (GDSC) database as the training set. The Mann–Whitney U test was applied to assess differential drug sensitivity between Cluster 1 and Cluster 2 for the top 20 significantly associated anticancer drugs across the six cancer types (Figure S10–15).

For each of the six cancer types, we identified overlapping drugs between those showing negative enrichment scores in CMap and those demonstrating significant associations in GDSC, visualized using Venn diagrams (Fig. 7C–H). IC50 values for the overlapping drugs were compared between patient subgroups stratified by high versus low MPERSRG expression, and drugs showing significant differences were identified through comparative analysis (Fig. 7I–K). In BLCA, three distinct gene-based stratifications revealed differential drug sensitivities: patients stratified by BRSK2 expression showed significant differences in response to vorinostat (*p* < 0.05), nilotinib (*p* < 0.05), and linsitinib (*p* < 0.05); NUPR1-based stratification identified eight drugs with differential sensitivity, including dactolisib, CDK9 inhibitors, mirin, IGF1R inhibitors, JAK1 inhibitors, olaparib, and paclitaxel (all *p* < 0.05); and NOD2-based stratification revealed significant differences for leflunomide (*p* < 0.05), cytarabine (*p* < 0.01), and crizotinib (*p* < 0.05).

### Association between MPERSRGs and immune characteristics

We calculated the abundance of 28 immune cell types in tumor samples from 33 cancer types using ssGSEA. Spearman correlation analysis was performed to assess associations between immune infiltration abundance and MPERSRG expression levels, with results visualized as correlation heatmaps (Figure S16A-C).

Given the critical role of immunotherapy in cancer treatment, we evaluated immunotherapy response prediction across 33 cancer types using the TIDE algorithm. TIDE scores were calculated and patients were stratified into high- and low-expression groups based on seven MPERSRGs (CEBPB, NUPR1, ATF3, CASP3, TNFSF10, BRSK2, NOD2). Comparative analyses with statistical testing are presented in supplementary figures (Figure S17–49).

Immunophenotype scores (IPS) for 20 cancer types (BLCA, BRCA, CESC, COAD, GBM, HNSC, KICH, KIRC, KIRP, LIHC, LUAD, LUSC, OV, PAAD, PRAD, READ, SKCM, STAD, THCA, UCEC) were obtained from The Cancer Immunome Atlas (TCIA) database. IPS analysis was performed across four immunotherapy scenarios: CTLA-4 negative/PD-1 negative, CTLA-4 negative/PD-1 positive, CTLA-4 positive/PD-1 negative, and CTLA-4 positive/PD-1 positive. Patients were stratified by MPERSRG expression levels (high vs. low for each of the seven genes), and comparative analyses are provided in supplementary figures (Figure S50–189).

Using the ESTIMATE algorithm, we calculated stromal scores, immune scores, and ESTIMATE scores from expression matrices of the 33 cancer types. Comparative analyses between high- and low-expression groups for each of the seven MPERSRGs are presented in supplementary figures (Figure S190–423).

Based on the correlation analysis between immune infiltration and MPERSRGs across cancer types, we selected TNFSF10 in LUAD for in-depth investigation using TIP analysis. TIP employs predefined immune-related gene sets and ssGSEA to quantify key steps of the anti-tumor immune response cycle, including immune cell infiltration, antigen presentation, and cytotoxic cell activity, thereby providing a comprehensive characterization of tumor immune status.

**Fig. 8:**
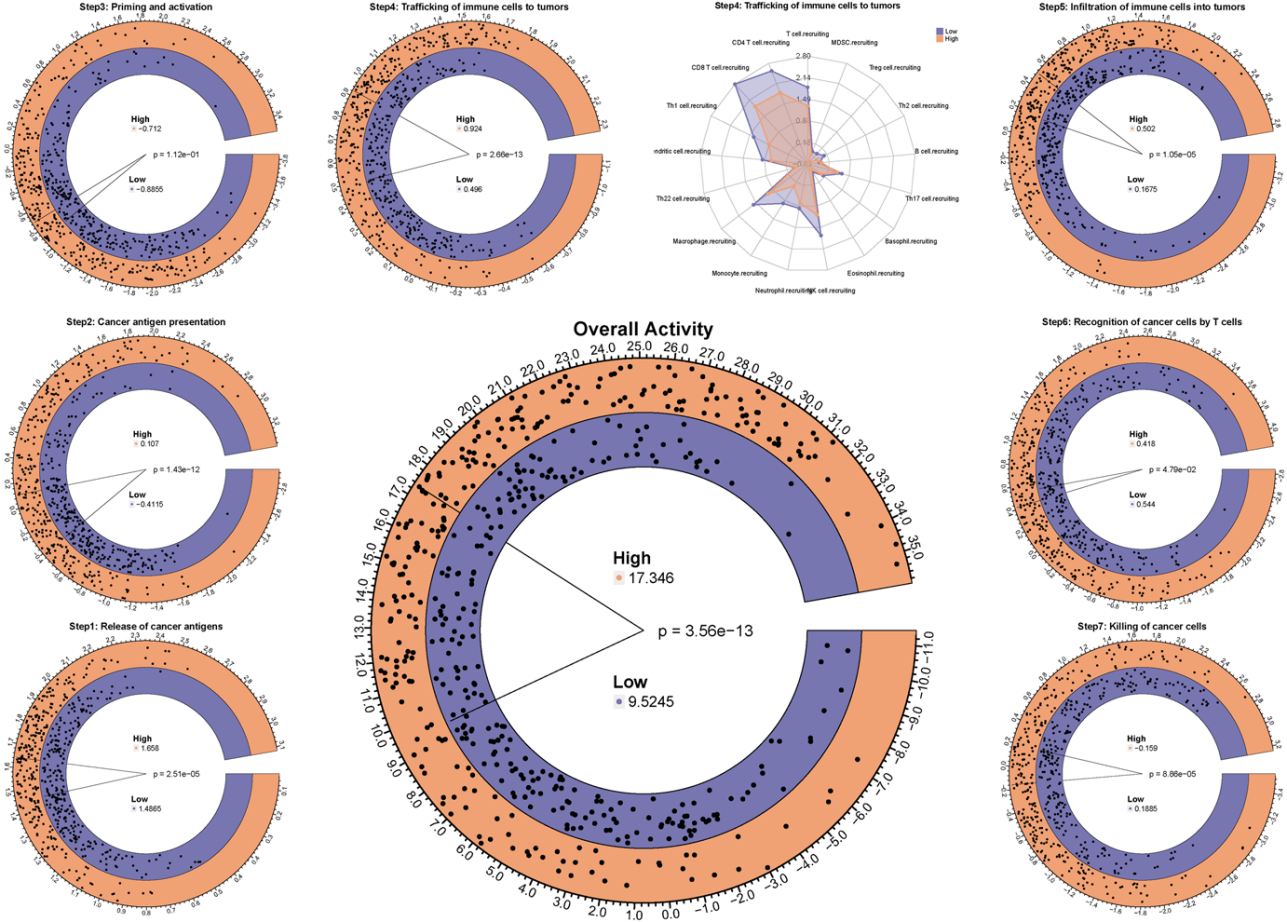
TIP Analysis in LUAD. Radar plot depicting the overall activity of the cancer-immunity cycle in LUAD patients. Samples were stratified by *TNFSF10* expression levels into high-expression (orange) and low-expression (purple) groups. Radial distance from the center indicates immune activity scores. Across steps 1–2 and 4–7, as well as in the aggregate analysis, the high-expression group demonstrated significantly elevated overall immune activity compared to the low-expression group, indicative of a more robust anti-tumor immune microenvironment

TIP analysis of LUAD patients stratified by TNFSF10 expression revealed significant differences in immune scores between high- and low-expression groups across multiple steps (steps 1–2 and 4–7) of the cancer-immunity cycle (Fig. 8). The radar plot visualization demonstrates that TNFSF10 effectively stratifies patients into two immunologically distinct groups. Across most steps, the high-expression group (orange) exhibited scores extending toward the periphery compared to the low-expression group (purple), indicating significantly enhanced overall anti-tumor immune activity. This comprehensive immune enhancement—encompassing improved antigen presentation, immune cell recruitment, activation, and effector functions—suggests that patients in the high-TNFSF10 expression group may be more likely to benefit from immunotherapy.

## Discussion

### Overview of principal findings

This comprehensive study systematically investigated the role of MPERSRGs across 33 cancer types, with particular emphasis on LUAD. Through integrated analysis of bulk transcriptomics, single-cell RNA sequencing, and machine learning approaches, we identified seven key MPERSRGs (CEBPB, NUPR1, ATF3, CASP3, TNFSF10, BRSK2, NOD2) that exhibit significant associations with tumor progression, immune microenvironment remodeling, and clinical outcomes. Our findings revealed distinct molecular subtypes based on MPERSRGs expression patterns, established a robust prognostic model for LUAD with excellent predictive performance (C-index = 0.723), and uncovered critical cellular heterogeneity within the tumor microenvironment, particularly among fibroblast subpopulations. These results provide a comprehensive framework for understanding the interplay between macrophage polarization and ER stress in cancer biology and offer actionable insights for precision medicine.

### Differential expression and molecular subtyping of MPERSRGs

Our pan-cancer analysis revealed widespread differential expression of MPERSRGs across 26 cancer cell lines and 30 tumor types, with distinct clustering into two major subtypes (Cluster1 and Cluster2). These findings align with recent reports highlighting the heterogeneity of ER stress responses and macrophage polarization states in different cancer contexts [32, 33]. The identification of two distinct MPERSRGs-based subtypes suggests fundamental differences in tumor biology that may underlie variations in treatment response and clinical outcomes. Previous studies have shown that molecular subtyping based on immune-related genes can effectively stratify patients for immunotherapy [34]. Our MPERSRGs-based classification extends this concept by integrating both immune regulation (macrophage polarization) and cellular stress responses (ER stress), potentially capturing a more comprehensive picture of tumor biology.

The differential expression patterns of MPERSRGs in tumor versus normal tissues provide valuable insights into their functional roles in carcinogenesis. For instance, the upregulation of NUPR1 (Nuclear Protein 1) in LUAD, as confirmed by proteomic data, is consistent with its known role in promoting tumor cell survival under ER stress conditions [34]. Similarly, elevated expression of TNFSF10 (TRAIL) has been associated with enhanced anti-tumor immunity, though its role can be context-dependent [35]. These findings suggest that MPERSRGs could serve as early detection biomarkers or targets for preventive interventions, particularly in high-risk populations.

### Pathway enrichment and mechanistic insights

Our GSEA revealed that individual MPERSRGs are associated with distinct biological pathways, reflecting their diverse functions in cancer biology. ATF3 (Activating Transcription Factor 3), a key component of the ER stress response, showed enrichment in GABA synthesis and metabolic pathways, consistent with its role in regulating cellular metabolism under stress [36]. BRSK2 (BR Serine/Threonine Kinase 2) was associated with complement cascade activation, suggesting a potential link to innate immunity and inflammation [35]. CASP3 (Caspase 3), the executioner caspase in apoptosis, showed enrichment in liver cancer-related pathways, highlighting its role in tissue-specific tumor suppression [37].

Notably, several MPERSRGs demonstrated significant correlations with hallmark inflammatory response, interferon-gamma response, and TNF-α signaling pathways. This finding underscores the intimate connection between ER stress and immune activation in the tumor microenvironment [38, 39]. The TNF-α pathway, in particular, plays a crucial role in macrophage polarization, creating a potential feedback loop where ER stress influences macrophage phenotype, which in turn modulates the inflammatory milieu [40]. This crosstalk may explain why MPERSRGs-based subtypes show differential responses to immunotherapy.

The association between MPERSRGs expression and ESTIMATE scores across multiple cancer types further validates their role in shaping the tumor microenvironment. High MPERSRGs expression was generally correlated with increased immune and stromal cell infiltration, suggesting an "inflamed" tumor phenotype that might be more amenable to immune checkpoint blockade therapy [41]. However, this relationship was not uniform across all cancer types, indicating tissue-specific factors that modulate the MPERSRGs-immune microenvironment axis. Understanding these context-dependent mechanisms will be crucial for developing personalized treatment strategies.

### MPERSRGs and tumor immune microenvironment

Our comprehensive immune infiltration analysis using ssGSEA revealed complex associations between MPERSRGs expression and 28 immune cell types across 33 cancers. The finding that high TNFSF10 expression in LUAD correlates with enhanced overall immune activity, as demonstrated by TIP analysis, is particularly noteworthy. TNFSF10 (TRAIL) is known to induce apoptosis in tumor cells while sparing normal cells, and its expression by immune cells can enhance anti-tumor immunity [42]. Recent studies have shown that TRAIL-expressing immune cells are associated with favorable prognosis in several cancers [43]. Our results suggest that TNFSF10 could serve as a biomarker to identify patients with an active immune microenvironment who are more likely to benefit from immunotherapy.

The correlation between MPERSRGs and various T cell subsets, including activated CD8 + T cells and regulatory T cells, highlights their potential role in modulating adaptive immunity. ER stress in tumor cells can affect antigen presentation and immunogenicity, thereby influencing T cell recognition and response [44, 45]. Moreover, macrophage polarization directly impacts T cell function through cytokine secretion and checkpoint molecule expression [46]. Our findings suggest that MPERSRGs-based stratification could complement existing biomarkers such as PD-L1 expression and tumor mutational burden in predicting immunotherapy response.

The TIDE and IPS analyses across multiple cancer types revealed that MPERSRGs expression levels significantly influence predicted immunotherapy response. Patients with high expression of certain MPERSRGs showed lower TIDE scores, suggesting reduced immune dysfunction and exclusion [47]. This observation aligns with the concept that an active ER stress response and balanced macrophage polarization can enhance tumor immunogenicity. However, the relationship varied across different MPERSRGs and cancer types, emphasizing the need for context-specific interpretation and validation in clinical cohorts.

### Prognostic model and clinical translation

The prognostic model we developed for LUAD, incorporating five MPERSRGs-correlated genes (FAM83A, RHOV, CPS1, STRIP2, SLC2A1), demonstrated robust predictive performance with a C-index of 0.723. This performance is comparable to or exceeds many published prognostic signatures for LUAD [48, 49]. The integration of 117 machine learning algorithm combinations enabled comprehensive evaluation and selection of the optimal model, minimizing overfitting and ensuring generalizability. The superior performance of the StepCox[forward] + RSF combination highlights the value of ensemble approaches in biomarker discovery.

Each gene in the prognostic model has biological relevance to cancer progression. FAM83A (Family with sequence similarity 83 member A) is an oncogene involved in EGFR signaling and has been associated with poor prognosis in multiple cancers [50]. RHOV (Ras Homolog Family Member V) regulates cytoskeletal dynamics and cell migration, contributing to metastasis [51]. CPS1 (Carbamoyl-Phosphate Synthase 1) is a key enzyme in ammonia detoxification and has emerging roles in cancer metabolism. SLC2A1 (GLUT1) is a glucose transporter essential for the Warburg effect, and its overexpression is associated with aggressive tumor behavior and resistance to therapy.

The time-dependent ROC curves and decision curve analysis confirmed that our model provides optimal clinical utility for 3-year survival prediction. This finding is particularly valuable for clinical decision-making, as 3-year survival is a critical milestone in LUAD management. The calibration curves demonstrated excellent agreement between predicted and observed outcomes, supporting the model's reliability. Future studies should validate this model in independent cohorts and explore its utility in guiding treatment decisions, such as the intensity of adjuvant therapy or eligibility for clinical trials.

### Single-cell insights into cellular heterogeneity

Our single-cell RNA sequencing analysis of LUAD samples revealed that fibroblasts exhibited the highest MPERSRGs expression scores among eight major cell types. Cancer-associated fibroblasts (CAFs) are increasingly recognized as key orchestrators of the tumor microenvironment, promoting tumor growth, angiogenesis, immune suppression, and therapy resistance through diverse mechanisms [52, 53]. The identification of three fibroblast subpopulations (MFAP5+, MATK+, and HP + fibroblasts) with distinct MPERSRGs expression and pathway enrichment patterns underscores the functional heterogeneity within the CAF compartment.

MFAP5 + fibroblasts showed enrichment in estrogen response, glycolysis, and epithelial-mesenchymal transition (EMT) pathways, suggesting a role in metabolic reprogramming and promoting tumor invasiveness. MATK + fibroblasts were associated with inflammatory response and allograft rejection pathways, indicating a more immunoactive phenotype. HP + fibroblasts displayed enrichment in cell cycle pathways, suggesting a proliferative state. These functional distinctions align with recent reports of CAF heterogeneity and their differential impacts on tumor progression and therapy response [54, 55].

The CellChat analysis revealed extensive communication between fibroblasts and epithelial cells, with the CLEC2C-KLRB1 ligand-receptor pair showing the strongest interaction among immune cells. CLEC2C is a C-type lectin receptor expressed on myeloid cells, while KLRB1 (CD161) is found on NK cells and T cell subsets, playing roles in immune regulation [56]. This finding suggests that targeting specific cell–cell communication pathways could disrupt the immunosuppressive microenvironment. Moreover, the inferCNV analysis confirmed the malignant nature of epithelial cell clusters, providing confidence in our cellular annotations and highlighting the genomic instability characteristic of cancer cells.

### Multilayer regulatory architecture of MPERSRGs: biological interpretation of the mRNA-miRNA, mRNA-Drug, and mRNA-TF Networks

The three-tiered regulatory network constructed in this study (Fig. 10) reveals a hierarchical landscape governing MPERSRG expression that offers mechanistically grounded insights into tumor biology and therapeutic intervention. At the post-transcriptional level, the mRNA-miRNA network comprising 3 MPERSRGs and 45 miRNAs reflects stringent cooperative regulatory control: miRNAs targeting ATF3 include members that modulate ER stress-induced apoptosis through the PERK-eIF2α-ATF4-ATF3-CHOP axis, wherein ATF3 transactivates death receptor DR5 to sensitize tumor cells to apoptotic stimuli—a mechanism whose disruption by oncogenic miRNAs may confer ER stress resistance [57]. miRNAs regulating CEBPB govern macrophage differentiation and cytokine production through NF-κB-dependent programs, such that their dysregulation rewires macrophage polarization toward an immunosuppressive M2-like phenotype that sustains tumor progression, highlighting these miRNAs as both diagnostic biomarkers and candidate therapeutic targets [58]. At the pharmacological level, the mRNA-drug network of 31 compounds converges on MPERSRG-related pathways through shared NF-κB and MAPK effectors; cross-validation between CTD-derived interactions and GDSC/CMap-based sensitivity predictions (Fig. 11I–K) provides convergent evidence strengthening confidence in the therapeutic relevance of identified compounds. At the transcriptional level, the mRNA-TF network of 4 MPERSRGs and 39 TFs defines the upstream regulatory layer coordinating MPERSRG expression in response to microenvironmental cues. Stress-responsive TFs including ATF4 and XBP1 function as molecular bridges linking ER stress to macrophage polarization—PERK–ATF4 stabilizes M2-like metabolic reprogramming while IRE1α–XBP1s sustains immunosuppressive myeloid programming via STAT3 activation—and STAT and NF-κB family members further reinforce this coupling by governing context-dependent macrophage activation programs [59, 60]. Collectively, these three regulatory layers define the full MPERSRG regulatory landscape and identify multiple actionable nodes for simultaneously modulating ER stress adaptation and macrophage polarization in the tumor microenvironment.

### Mechanistic basis for differential drug sensitivity across MPERSRG-stratified subgroups

Our integrated drug prediction approach combining CMap and GDSC databases identified several promising therapeutic compounds with differential efficacy based on MPERSRGs expression, and the observed sensitivities are grounded in converging molecular mechanisms at the intersection of ER stress, DNA damage response, and tumor immunity. The markedly lower IC50 values for vorinostat in BRSK2-high BLCA tumors reflect HDAC inhibition's dual capacity to activate pro-apoptotic UPR branches—via PERK dissociation and IRE1/XBP1/ATF4 signaling—while simultaneously reversing immunosuppression by restoring antigen processing machinery and T cell recognition, providing strong rationale for combining vorinostat with immune checkpoint blockade [61, 62]. NUPR1-associated olaparib sensitivity is mechanistically rooted in NUPR1's role as a stress-responsive coregulator that sustains pro-survival UPR branches at the cost of homologous recombination fidelity, creating a functional HRD-like vulnerability exploitable through PARP inhibitor-induced synthetic lethality—a mechanism extending beyond canonical BRCA-mutated contexts [63, 64]. The additional sensitivities to dactolisib and paclitaxel in NUPR1-high tumors align with NUPR1-dependent mTORC1 translational programs and paclitaxel-triggered ER stress-dependent immunogenic cell death, respectively. NOD2-high tumor sensitivity to leflunomide, cytarabine, and crizotinib is consistent with NOD2's activation of the AMPK–LKB1 autophagy axis, which amplifies genotoxic chemosensitivity in multiple cancer contexts [65].

The mechanistic connections between identified compounds and MPERSRG-associated signaling pathways provide biological coherence for the observed drug sensitivities. Vorinostat's enhanced efficacy in BRSK2-high BLCA tumors reflects its convergent suppression of ERK/NF-κB inflammatory signaling—a shared downstream effector of BRSK2-driven ER stress—and its reversal of M2 macrophage polarization through blockade of the HDAC6/IL-10 immunosuppressive axis, collectively dismantling the tumor-promoting microenvironment sustained by MPERSRG dysregulation [66, 67]. NUPR1-associated olaparib sensitivity is mechanistically rooted in NUPR1's dual role in maintaining pro-survival UPR branches while impairing homologous recombination fidelity, generating a functional HRD-like state that renders NUPR1-high tumors acutely dependent on PARP-mediated repair and vulnerable to synthetic lethality [63]. Nilotinib's differential efficacy in BRSK2-high subgroups is consistent with its broad kinase inhibitory profile encompassing CSF-1R, KIT, and p38 MAPK, which converges on the NF-κB/MAPK axis governing both ER stress survival programs and macrophage polarization states relevant to MPERSRG biology [68]. These mechanistic linkages reinforce the biological rationale for MPERSRG expression-guided therapeutic stratification.

### An MPERSRGs-guided precision medicine framework

The convergent findings from drug sensitivity profiling and regulatory network analysis support an MPERSRGs-guided precision oncology framework in which NUPR1, BRSK2, and NOD2 expression profiles serve as first-tier molecular classifiers stratifying patients by ER stress adaptation status, DNA repair capacity, and innate immune signaling trajectory. This stratification enables mechanistically rationalized treatment selection: HDAC inhibitors for BRSK2-high immunosuppressive tumors, PARP inhibitors for NUPR1-high tumors with replicative stress, and autophagy-modulating or kinase inhibitor regimens for NOD2-high tumors—complementing rather than replacing existing biomarkers such as PD-L1 expression or tumor mutational burden [69]. The integration of MPERSRG-defined molecular subtypes with drug sensitivity predictions could improve response rates while reducing empirical treatment toxicity, and prospective validation in biomarker-enriched cohorts will be essential to establish the clinical utility of this framework [70].

### Limitations and future directions

Despite the comprehensive nature of our study, several limitations should be acknowledged. First, our analyses were primarily based on publicly available datasets and bioinformatics approaches, lacking experimental validation in laboratory settings. While computational predictions provide valuable hypotheses, functional studies are essential to confirm the mechanistic roles of MPERSRGs in macrophage polarization, ER stress, and their effects on tumor progression. Future research should include in vitro and in vivo experiments to validate key findings, such as the effects of modulating individual MPERSRGs on tumor growth, immune infiltration, and therapy response.

Furthermore, it is important to acknowledge the potential impact of batch effects on our cross-dataset validation. In this study, we opted to apply the established predictive model directly to independent validation cohorts rather than merging datasets for a joint analysis; consequently, computational batch effect correction methods (such as ComBat) were not applied. While this approach tests the model's robustness against technical variations, the assumption that underlying biological signals outweigh uncorrected technical shifts may not be entirely rigorous, potentially influencing the precise assessment of the model's generalization capability. Future multicenter validation studies should incorporate strict batch correction algorithms or rely on large-scale, prospective cohorts sequenced on a unified platform to further solidify these findings. The consistency of clinical covariates across the analyzed datasets was not systematically evaluated in this study. Due to missing or heterogeneous clinical annotations in the GEO validation cohorts (GSE19188 and GSE31210) compared to the TCGA training cohort, we were unable to perform rigorous clinical covariate matching or correct for potential confounding effects. Consequently, underlying disparities in baseline clinical characteristics—such as age, gender, and tumor stage—might have influenced the distribution of risk scores and the observed prognostic associations. The lack of covariate-adjusted analysis implies that the predictive efficacy of our model within specific clinical subpopulations remains undetermined. Future prospective studies incorporating comprehensive, detailed, and standardized clinical data are essential to fully ascertain the stability and clinical applicability of this prognostic signature.

Third, while our prognostic model showed strong performance in LUAD, its generalizability to other cancer types requires further investigation. Each tumor type has unique biological characteristics, and molecular signatures may have differential prognostic value across histologies. Extending this analysis to other cancers and validating the models in large, independent cohorts would enhance their clinical applicability.

Fourth, single-cell RNA sequencing, while powerful for characterizing cellular heterogeneity, is limited by the number of cells and samples analyzed. Our study included 22 LUAD samples, which may not fully capture the diversity of tumor microenvironments across different disease stages and patient populations. Spatial transcriptomics approaches would provide additional insights into the spatial organization of MPERSRGs-expressing cells and their interactions with neighboring cells, further elucidating their functional roles.

Finally, the drug sensitivity predictions based on cell line data may not fully recapitulate responses in patients due to differences in tumor complexity, microenvironment, and pharmacokinetics. Clinical validation through biomarker-driven trials is necessary to establish the utility of MPERSRGs expression for guiding therapy selection. Nonetheless, our findings provide a strong rationale for such studies and highlight promising therapeutic avenues.

## Conclusions

In conclusion, this comprehensive pan-cancer study provides a systematic characterization of MPERSRGs across multiple levels of biological organization. We have demonstrated that MPERSRGs exhibit widespread differential expression across cancer types, define distinct molecular subtypes, correlate with clinical outcomes, shape the immune microenvironment, and influence drug sensitivity. Our findings bridge critical knowledge gaps at the intersection of ER stress biology and tumor immunology, offering new perspectives on cancer progression and therapeutic resistance.

The robust prognostic model developed for LUAD, combined with insights from single-cell analysis and drug prediction, provides a framework for precision oncology. By integrating bulk and single-cell transcriptomics with machine learning and pharmacogenomics, we have identified actionable biomarkers and therapeutic targets. The cellular heterogeneity revealed in our study, particularly among CAF subpopulations, highlights opportunities for microenvironment-directed therapies that could complement conventional treatments and immunotherapies.

Future research should focus on experimental validation of our computational findings, clinical translation through biomarker-driven trials, and integration with emerging technologies such as spatial transcriptomics and longitudinal single-cell profiling. As our understanding of the complex interplay between macrophage polarization, ER stress, and the tumor microenvironment deepens, MPERSRGs-targeted strategies may offer new hope for improving cancer patient outcomes. This study lays a foundation for such efforts and underscores the power of integrative multi-omics approaches in advancing precision cancer medicine.

## Supplementary Information

Additional file1 (PDF 70 KB)

Additional file2 (PDF 27884 KB)

Additional file3 (PDF 57226 KB)

Additional file4 (PDF 3357 KB)

Additional file5 (PDF 3141 KB)

Additional file6 (PDF 318 KB)

Additional file7 (ZIP 110 KB)

Additional file8 (PDF 148 KB)

Additional file9 (PDF 147 KB)

Additional file10 (ZIP 1469 KB)

Additional file11 (PDF 2684 KB)

Additional file12 (ZIP 3652 KB)

Additional file13 (DOCX 18 KB)

Additional file14 (XLSX 10 KB)

Additional file15 (CSV 1 KB)

Additional file16 (CSV 1 KB)

Additional file17 (CSV 2 KB)

Additional file18 (CSV 1 KB)

Additional file19 (CSV 1 KB)

Additional file20 (CSV 1 KB)

## Data Availability

All data generated or analyzed during this study are included in this published article and its supplementary information files. The Supplementary Figure S17–49, Figure S50–189, Figure S190–423 are available at NutStore (https://www.jianguoyun.com/p/DUV_g9QQ887bChj16JoGIAA, https://www.jianguoyun.com/p/DZxcBukQ887bChjz6JoGIAA and https://www.jianguoyun.com/p/DTe0-sUQ887bChj06JoGIAA). The datasets supporting the conclusions of this article are available in the following public repositories: TCGA: https://portal.gdc.cancer.gov/. GTEx: https://gtexportal.org/ and https://xena.ucsc.edu/. GEO datasets GSE19188, GSE31210, and GSE131907: https://www.ncbi.nlm.nih.gov/geo/. CCLE: https://sites.broadinstitute.org/ccle/. HPA: https://www.proteinatlas.org/. CPTAC data via UALCAN: http://ualcan.path.uab.edu/. MSigDB gene sets: https://www.gsea-msigdb.org/gsea/msigdb/. CTD: https://ctdbase.org/. ChIPBase: http://rna.sysu.edu.cn/chipbase/. CMap: https://clue.io/. GDSC: https://www.cancerrxgene.org/. TIDE: http://tide.dfci.harvard.edu/.

## Abbreviations

ER: Endoplasmic reticulum
MPERSRGs: ER stress-related genes
TCGA: The cancer genome atlas
GTEx: Genotype-tissue expression
CCLE: Cancer cell line encyclopedia
LUAD: Lung adenocarcinoma
TME: Tumor microenvironment
UPR: Unfolded protein response
TPM: Transcripts per million
MP: Macrophage polarization
ERS: Endoplasmic reticulum stress
CPTAC: Clinical proteomic tumor analysis consortium
GSEA: Gene set enrichment analysis
MSigDB: Molecular signatures database
BH: Benjamini–Hochberg
GSVA: Gene set variation analysis
RSF: Random survival forest
LASSO: Least absolute shrinkage and selection operator
GBM: Gradient boosting machine
survival-SVM: Survival support vector machine
SuperPC: Supervised principal component
plsRcox: Partial least squares regression for Cox models
OS: Overall survival
KM: Kaplan–Meier
ROC: Receiver operating characteristic
AUC: Area under the curve
scRNA-seq: Single-cell RNA sequencing
PCA: Principal component analysis
UMAP: Uniform manifold approximation and projection
CNVs: Copy number variations
miRNAs: MicroRNAs
TFs: Transcription factors
CMap: Connectivity map
