# Supplementary figures and images for: Integration of 117 machine learning algorithms and single-cell transcriptomics identifies macrophage polarization and ER stress signatures for cancer prognosis and precision therapy (part 1 of 2)
Source: Discov Oncol. 2026 Apr 30;17:917. doi: 10.1007/s12672-026-05126-6 (PMC13275951; doi:10.1007/s12672-026-05126-6)

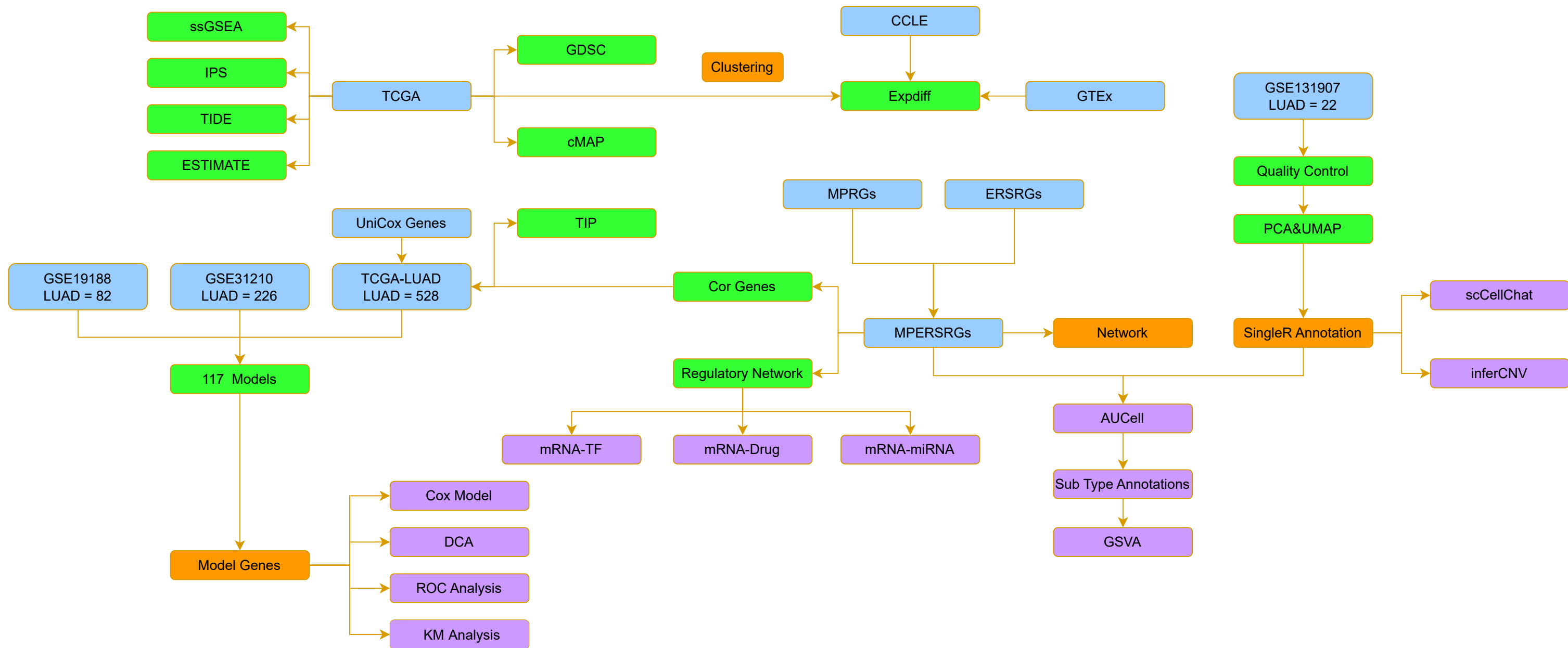

Supplement: Supplementary file 1 — Additional file1 (PDF 70 KB) [file 12672_2026_5126_MOESM1_ESM.pdf]

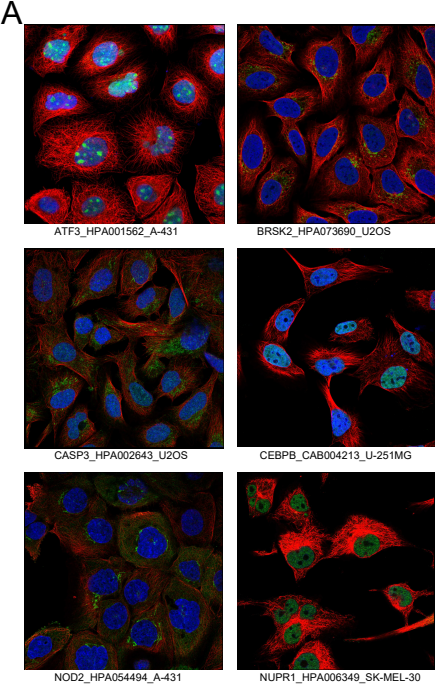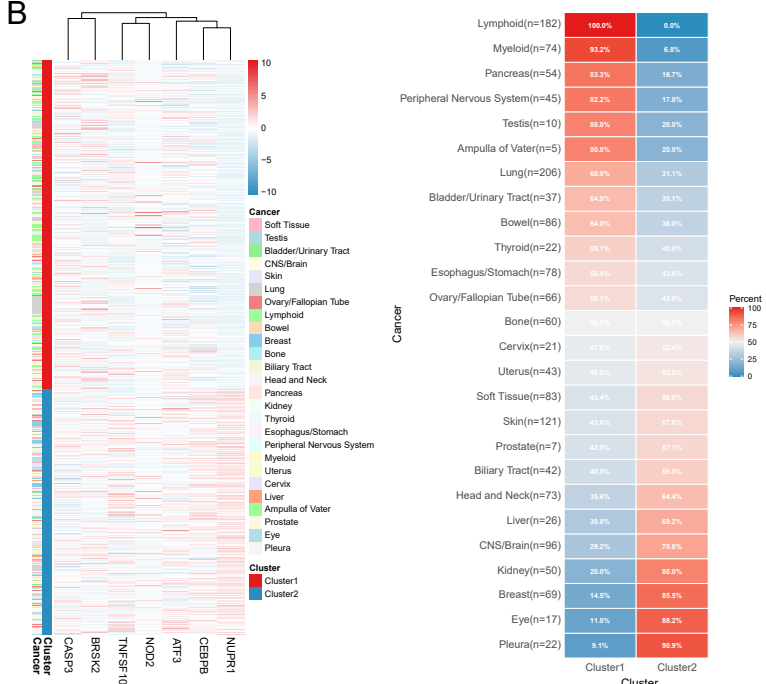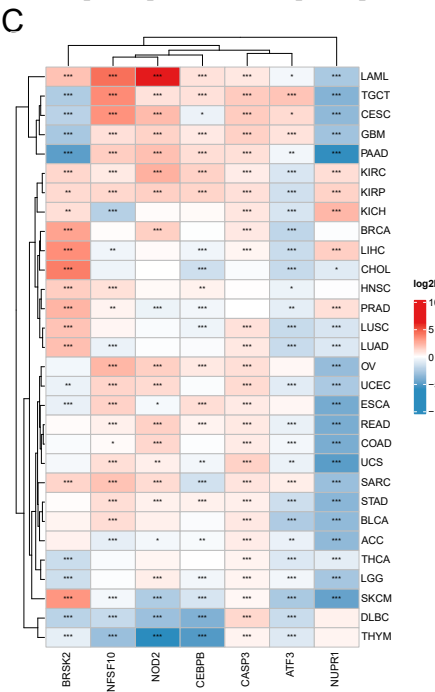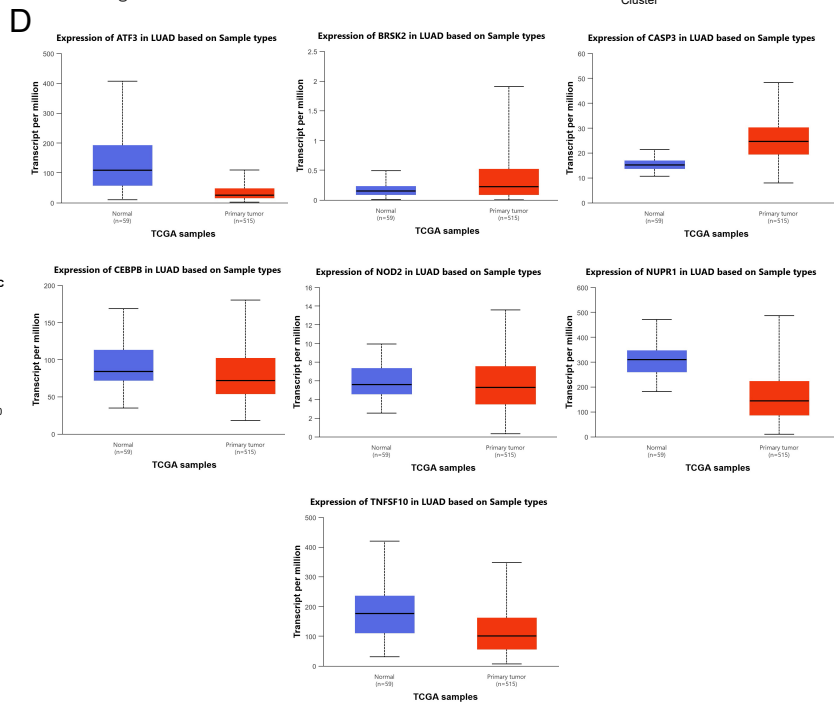

Supplement: Supplementary file 2 — Additional file2 (PDF 27884 KB) [file 12672_2026_5126_MOESM2_ESM.pdf]

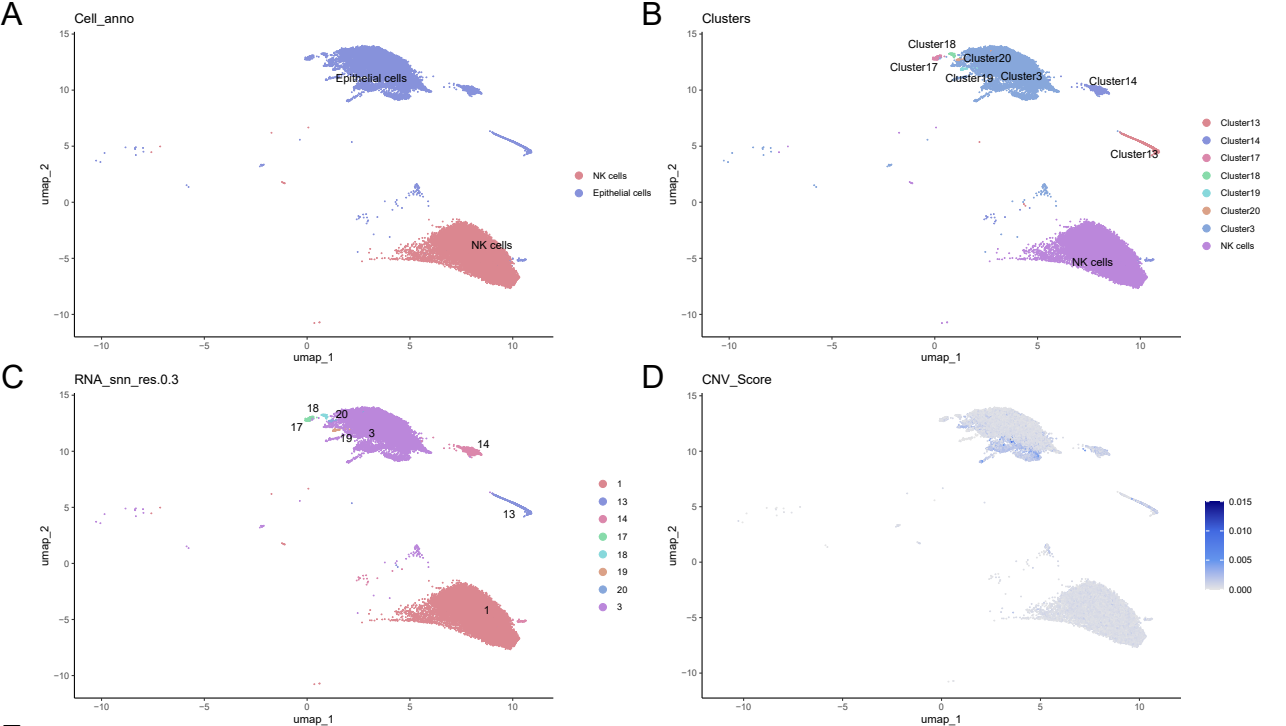

**E**

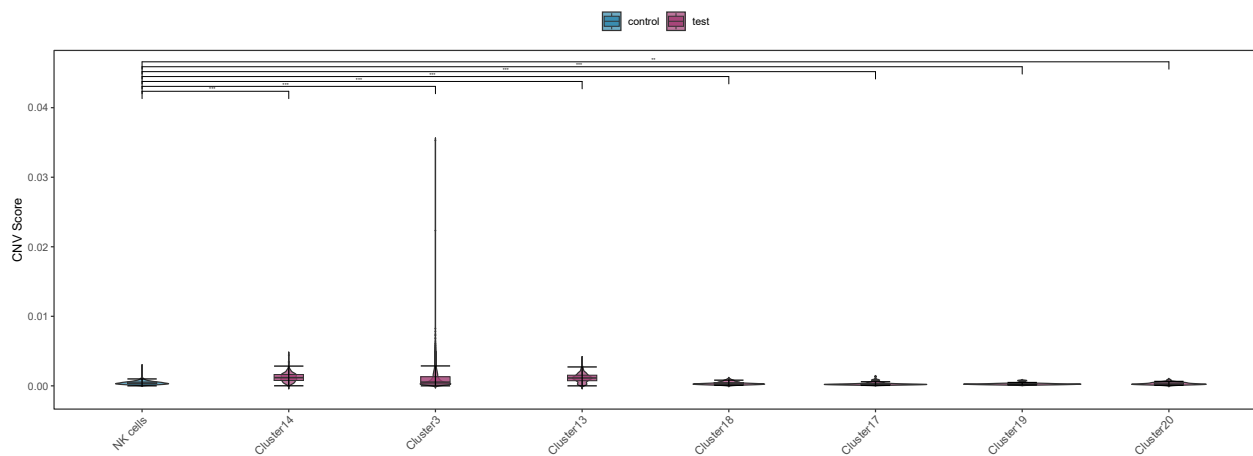

Supplement: Supplementary file 4 — Additional file4 (PDF 3357 KB) [file 12672_2026_5126_MOESM4_ESM.pdf]

inferCNV

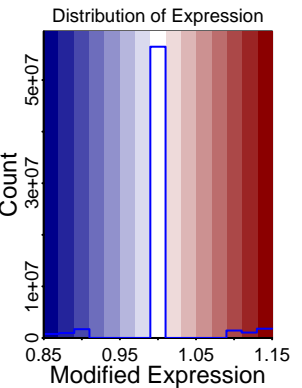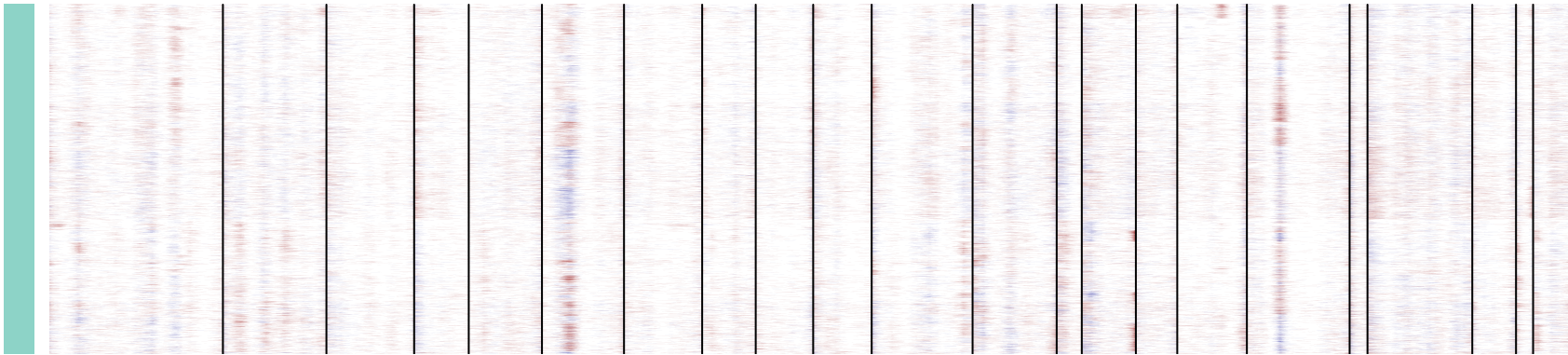

References (Cells)

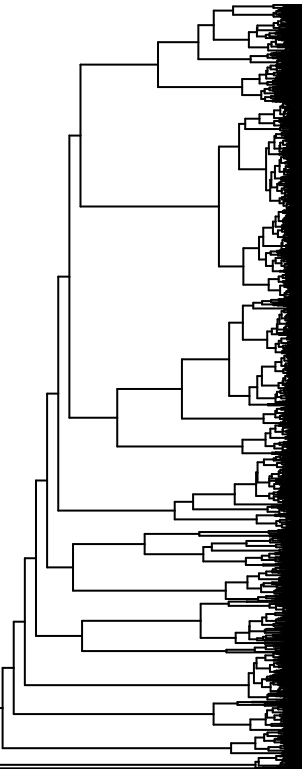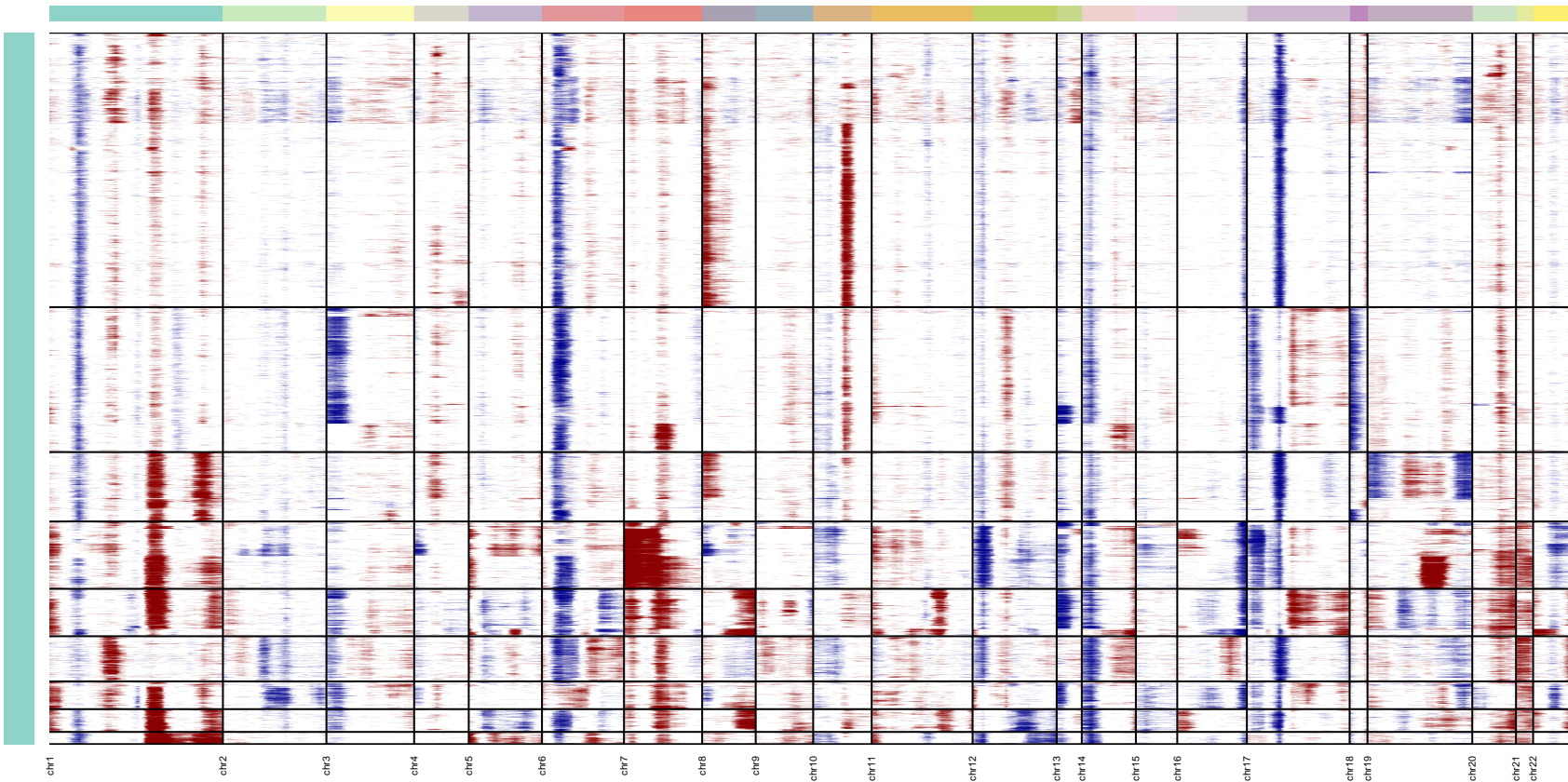

Observations (Cells)

Genomic Region

NK cells

Epithelial cells

Supplement: Supplementary file 5 — Additional file5 (PDF 3141 KB) [file 12672_2026_5126_MOESM5_ESM.pdf]

A

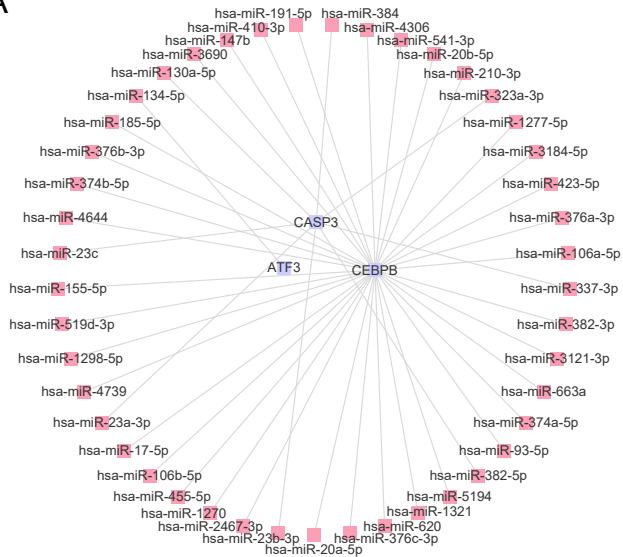

B

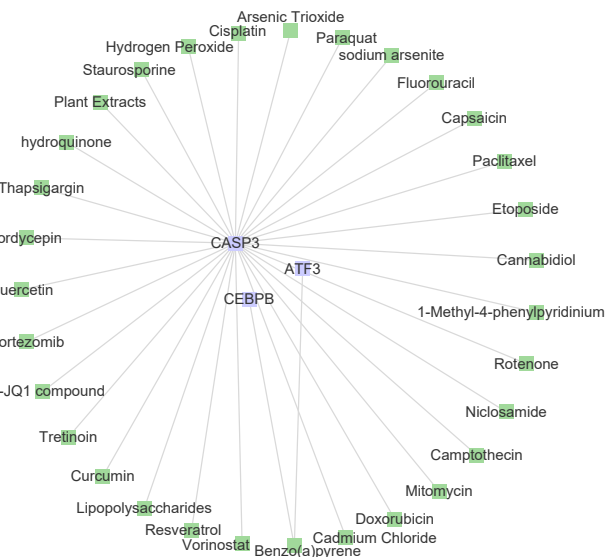

C

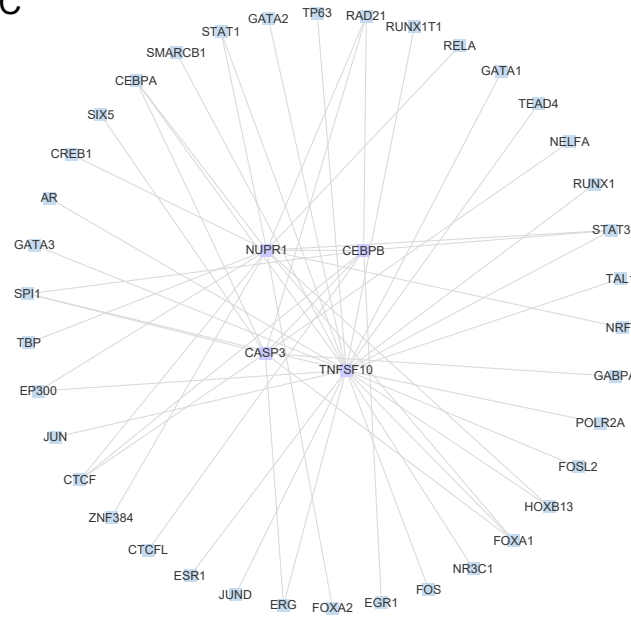

Supplement: Supplementary file 6 — Additional file6 (PDF 318 KB) [file 12672_2026_5126_MOESM6_ESM.pdf]

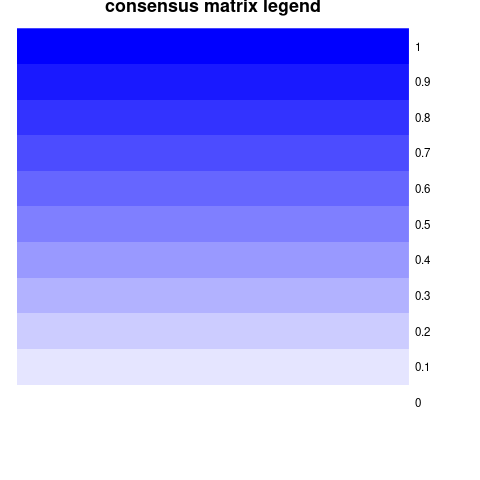

Supplement: Supplementary file 7 — Additional file7 (ZIP 110 KB) [file 12672_2026_5126_MOESM7_ESM.zip › Figure S7 consensus001.png]

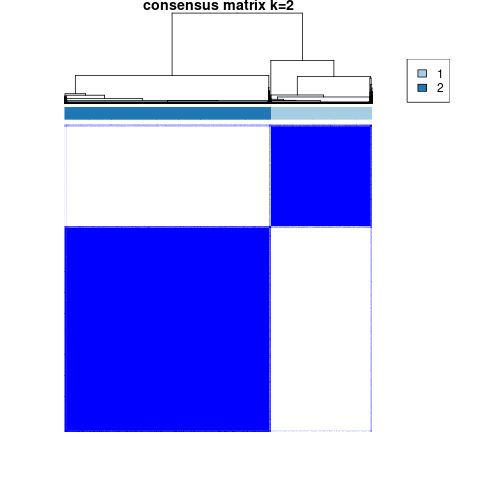

Supplement: Supplementary file 7 — Additional file7 (ZIP 110 KB) [file 12672_2026_5126_MOESM7_ESM.zip › Figure S7 consensus002.png]

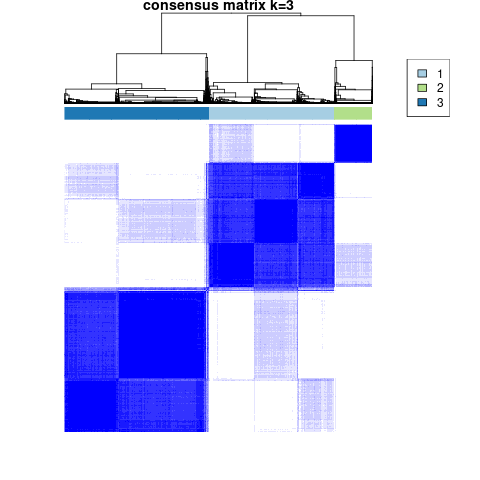

Supplement: Supplementary file 7 — Additional file7 (ZIP 110 KB) [file 12672_2026_5126_MOESM7_ESM.zip › Figure S7 consensus003.png]

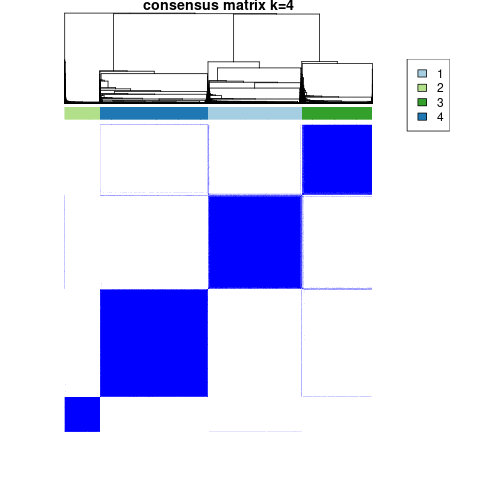

Supplement: Supplementary file 7 — Additional file7 (ZIP 110 KB) [file 12672_2026_5126_MOESM7_ESM.zip › Figure S7 consensus004.png]

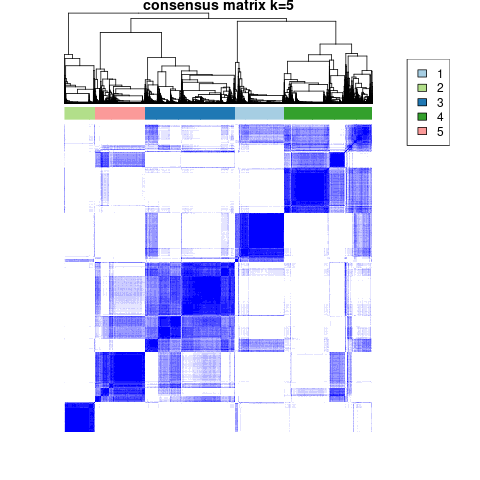

Supplement: Supplementary file 7 — Additional file7 (ZIP 110 KB) [file 12672_2026_5126_MOESM7_ESM.zip › Figure S7 consensus005.png]

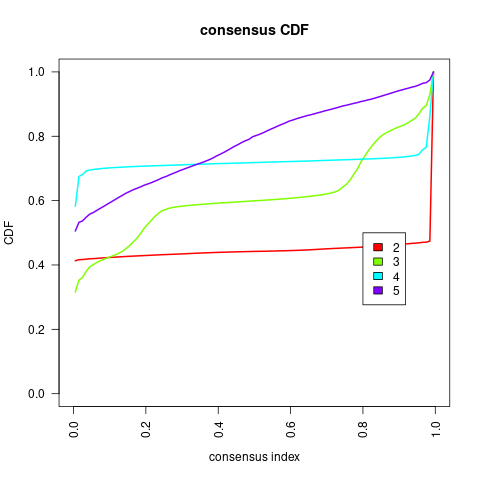

Supplement: Supplementary file 7 — Additional file7 (ZIP 110 KB) [file 12672_2026_5126_MOESM7_ESM.zip › Figure S7 consensus006.png]

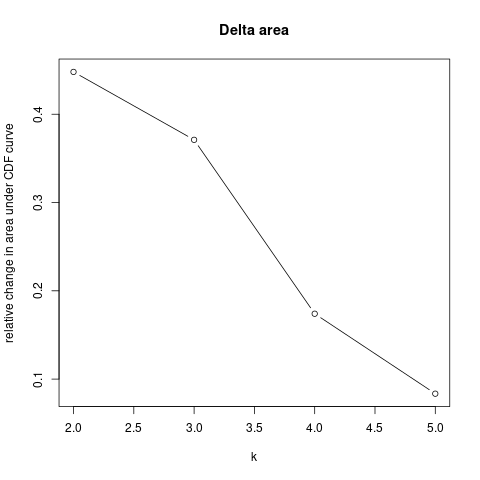

Supplement: Supplementary file 7 — Additional file7 (ZIP 110 KB) [file 12672_2026_5126_MOESM7_ESM.zip › Figure S7 consensus007.png]

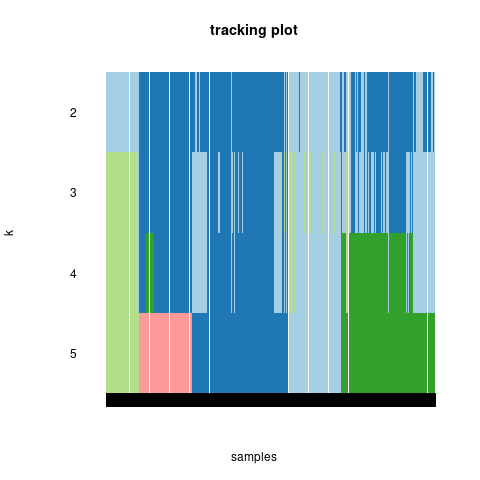

Supplement: Supplementary file 7 — Additional file7 (ZIP 110 KB) [file 12672_2026_5126_MOESM7_ESM.zip › Figure S7 consensus008.png]

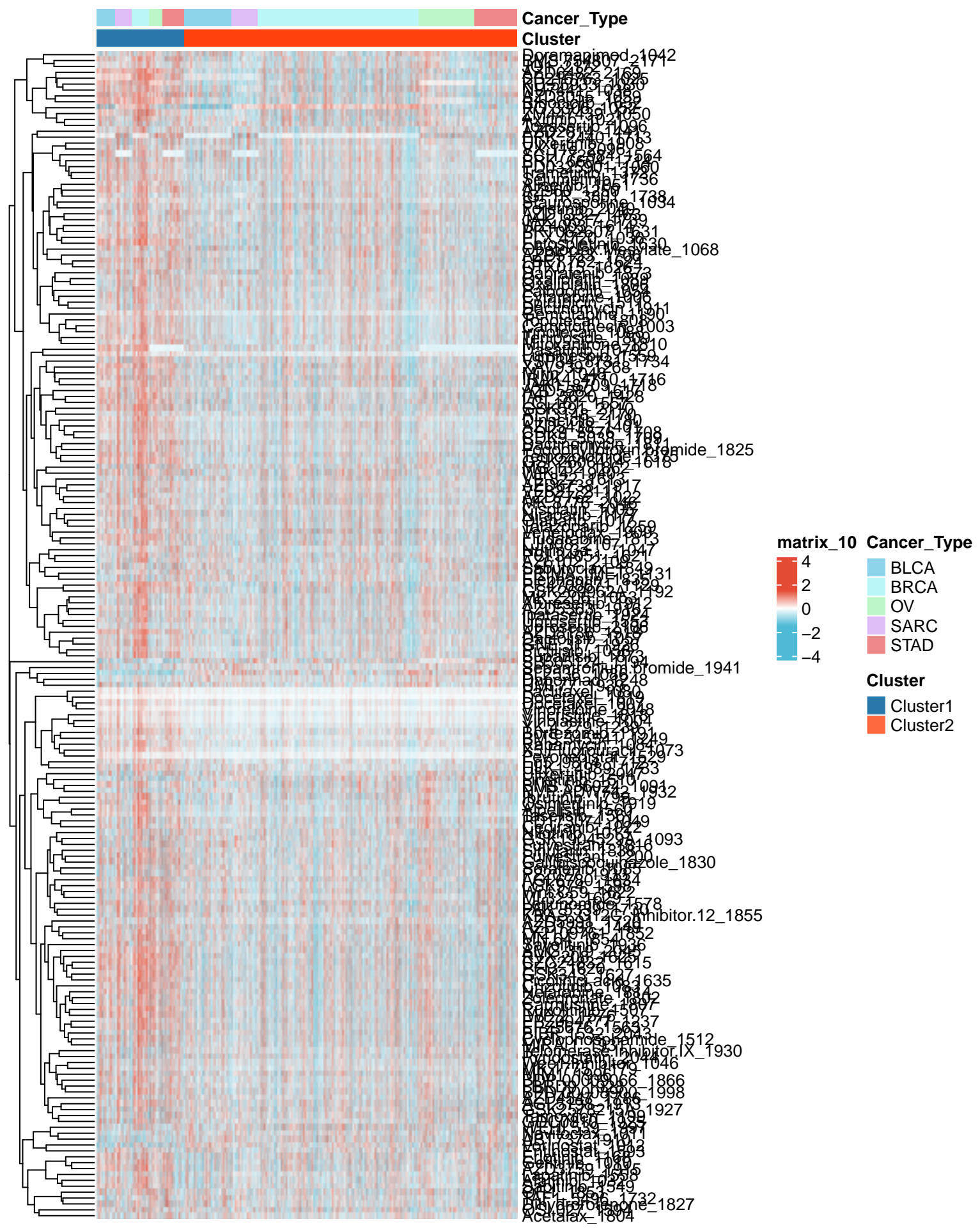

Supplement: Supplementary file 8 — Additional file8 (PDF 148 KB) [file 12672_2026_5126_MOESM8_ESM.pdf]

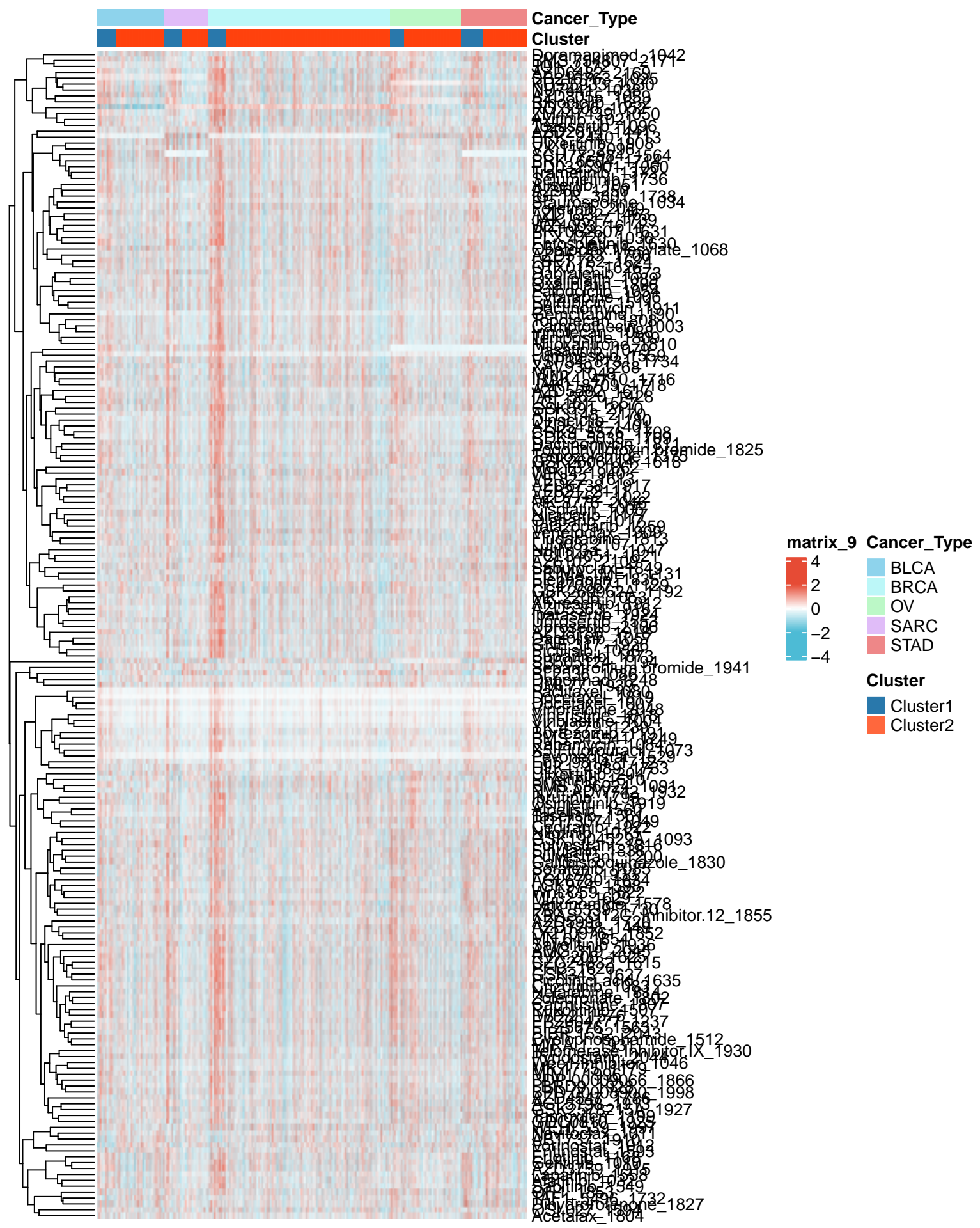

Supplement: Supplementary file 9 — Additional file9 (PDF 147 KB) [file 12672_2026_5126_MOESM9_ESM.pdf]

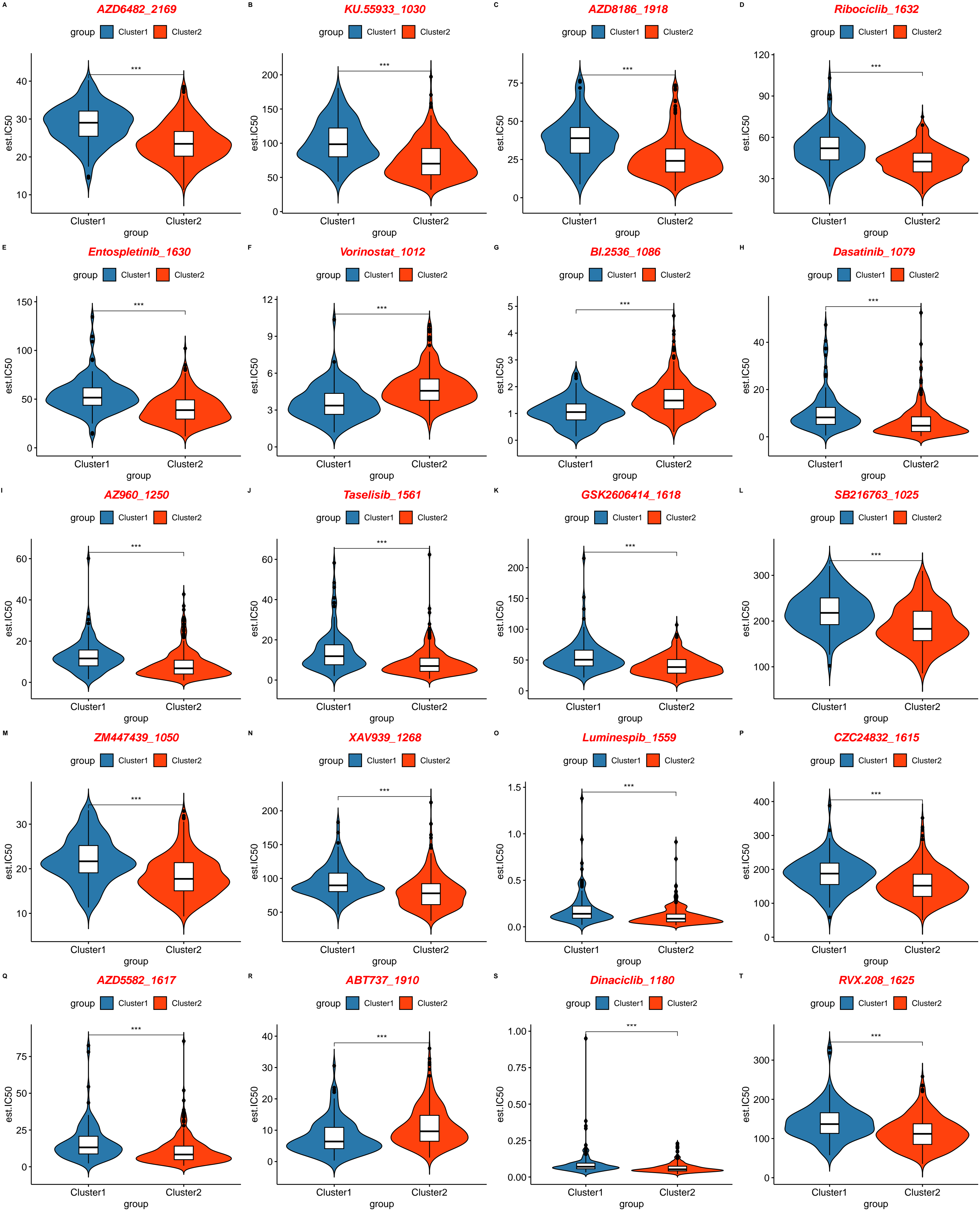

Supplement: Supplementary file 10 — Additional file10 (ZIP 1469 KB) [file 12672_2026_5126_MOESM10_ESM.zip › Figure S10-15 BLCA_oncoPredict_top20.pdf]

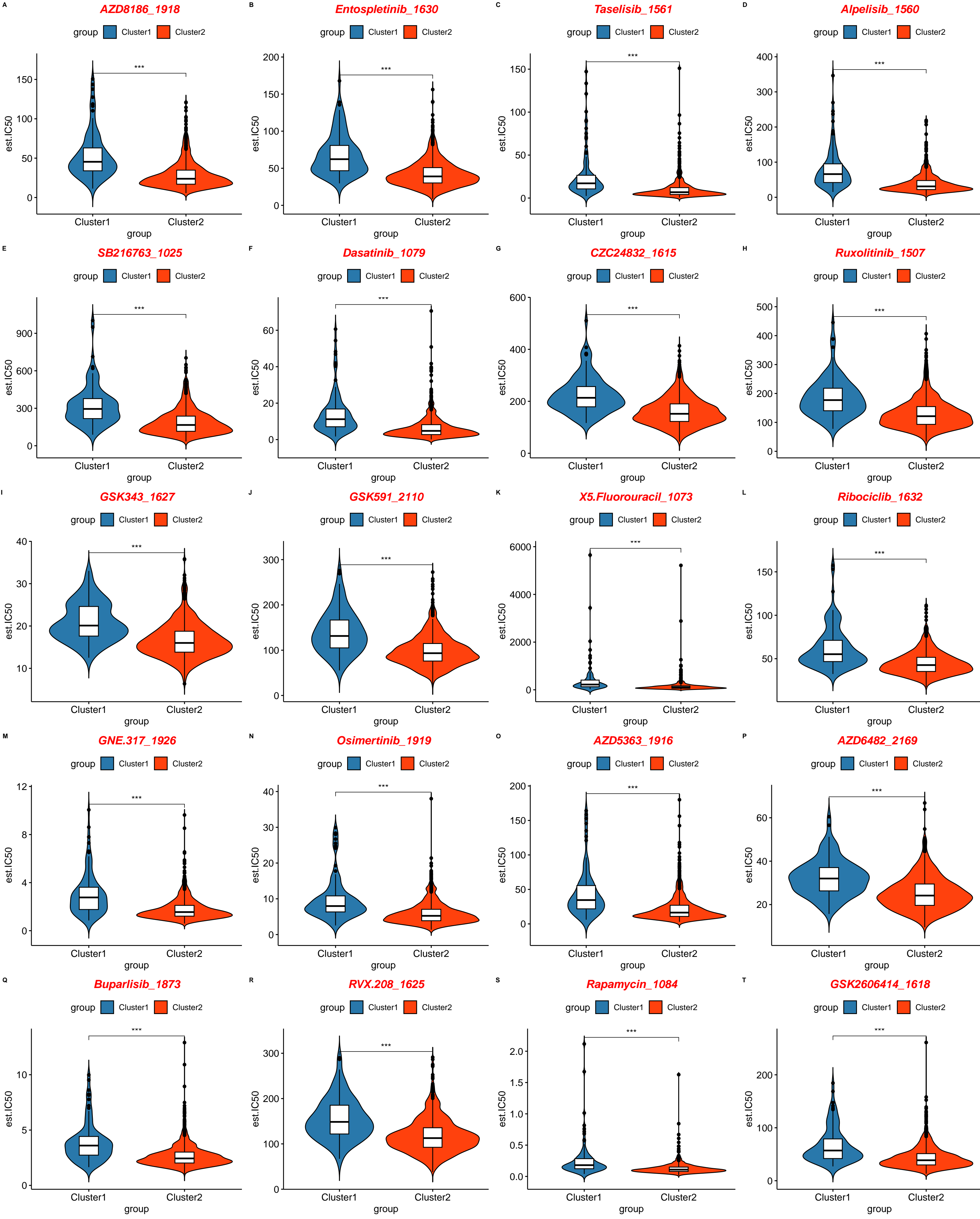

Supplement: Supplementary file 10 — Additional file10 (ZIP 1469 KB) [file 12672_2026_5126_MOESM10_ESM.zip › Figure S10-15 BRCA_oncoPredict_top20.pdf]

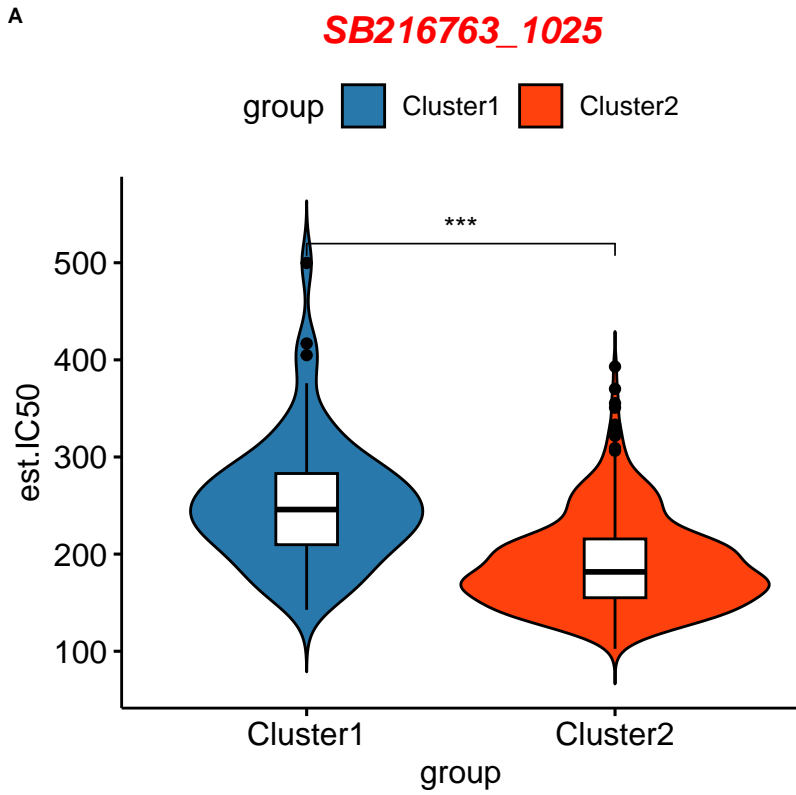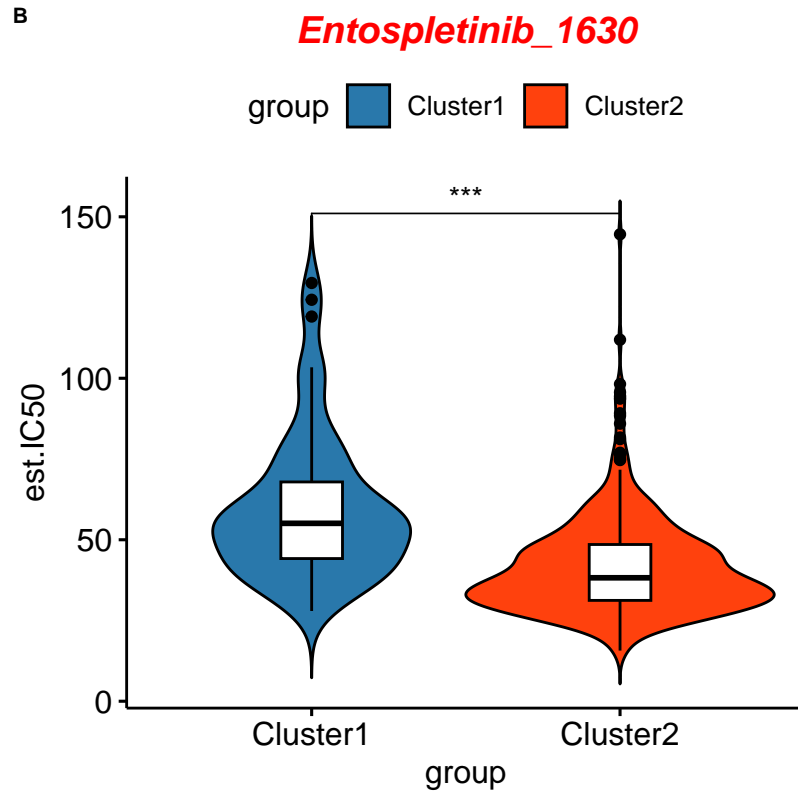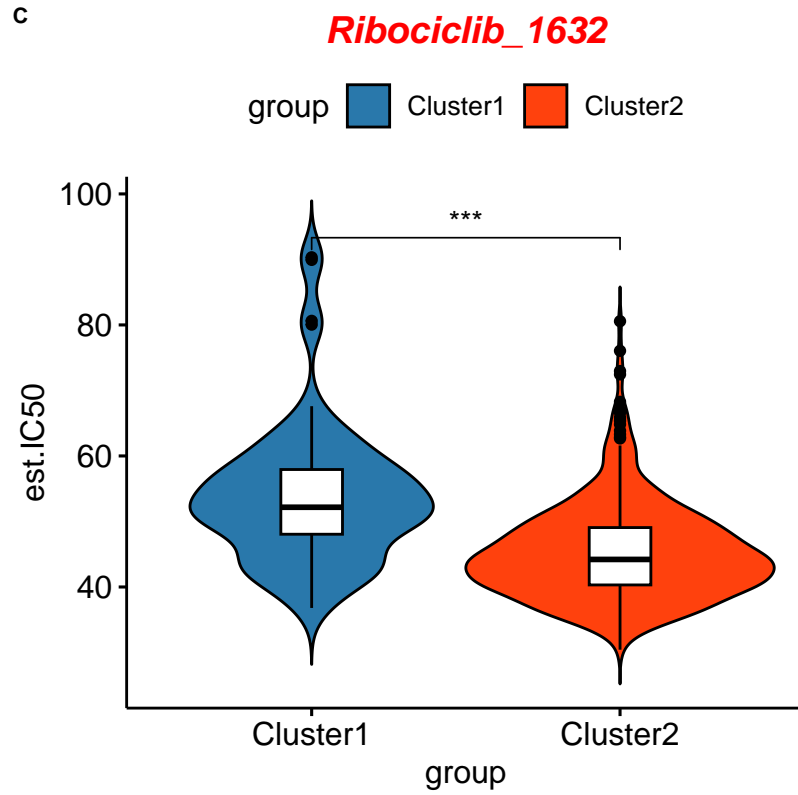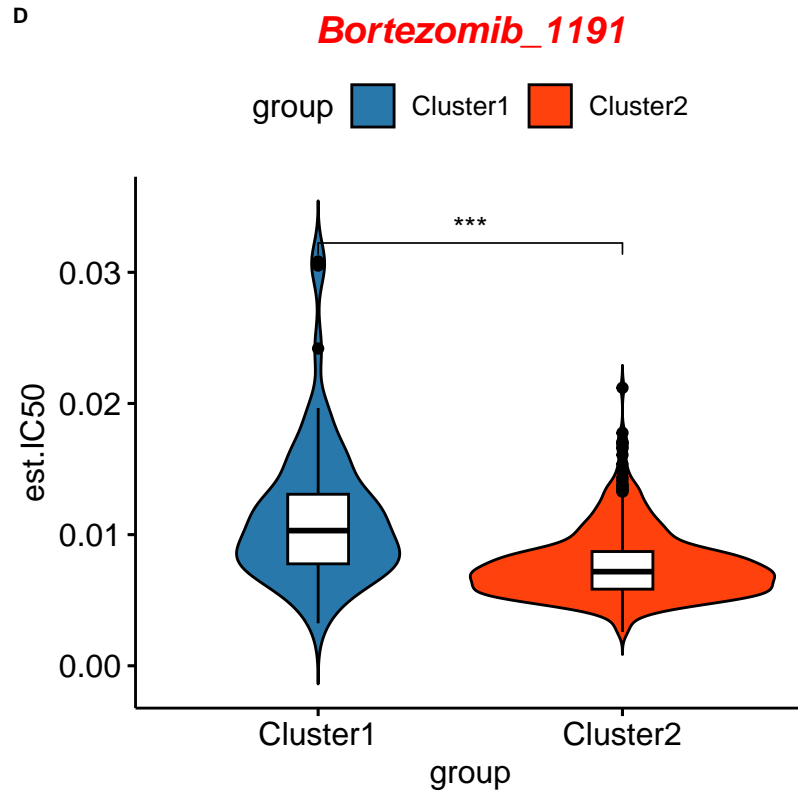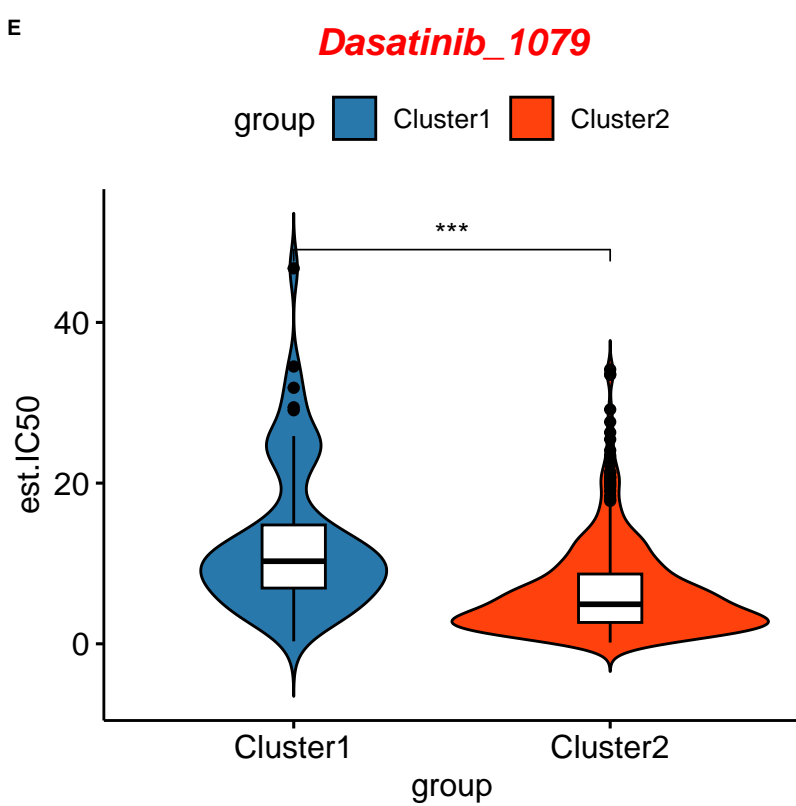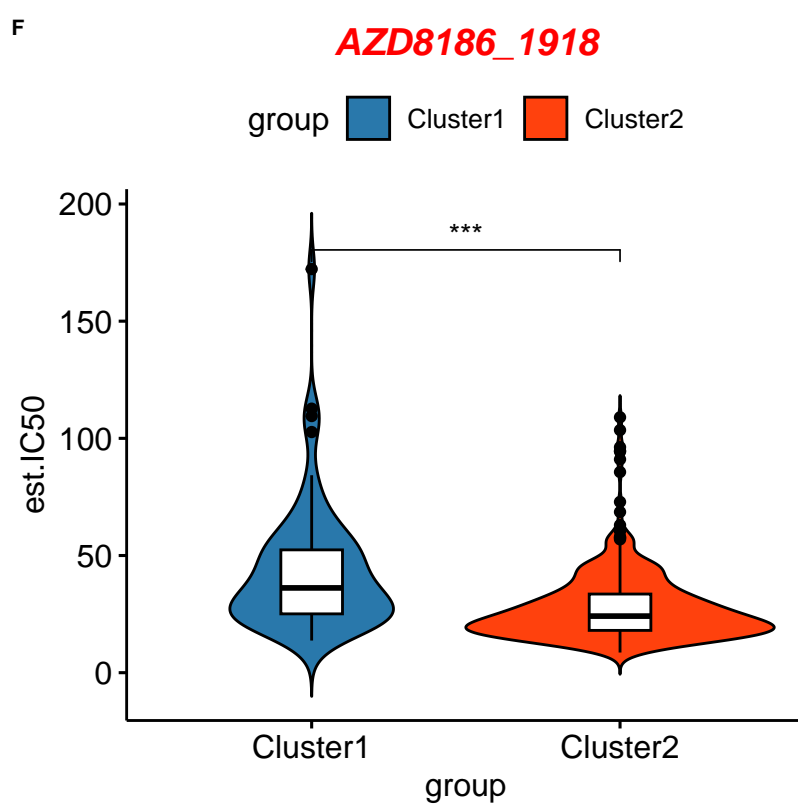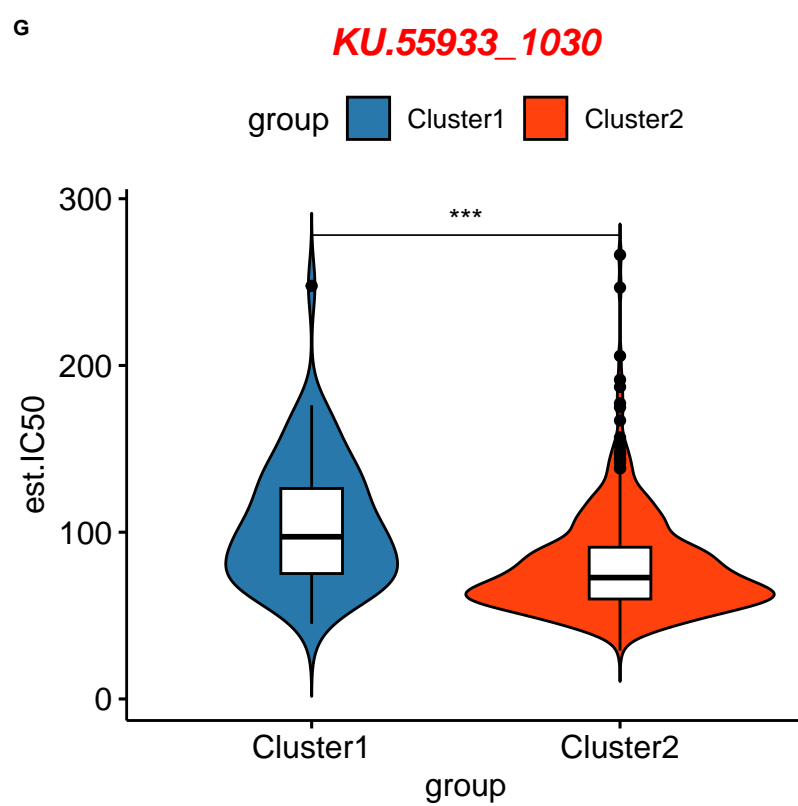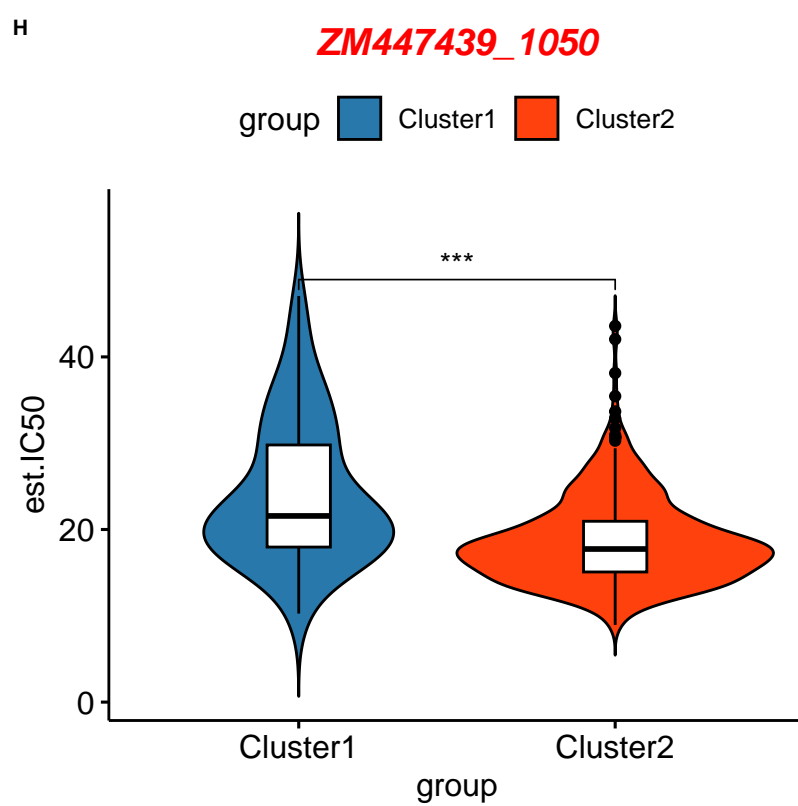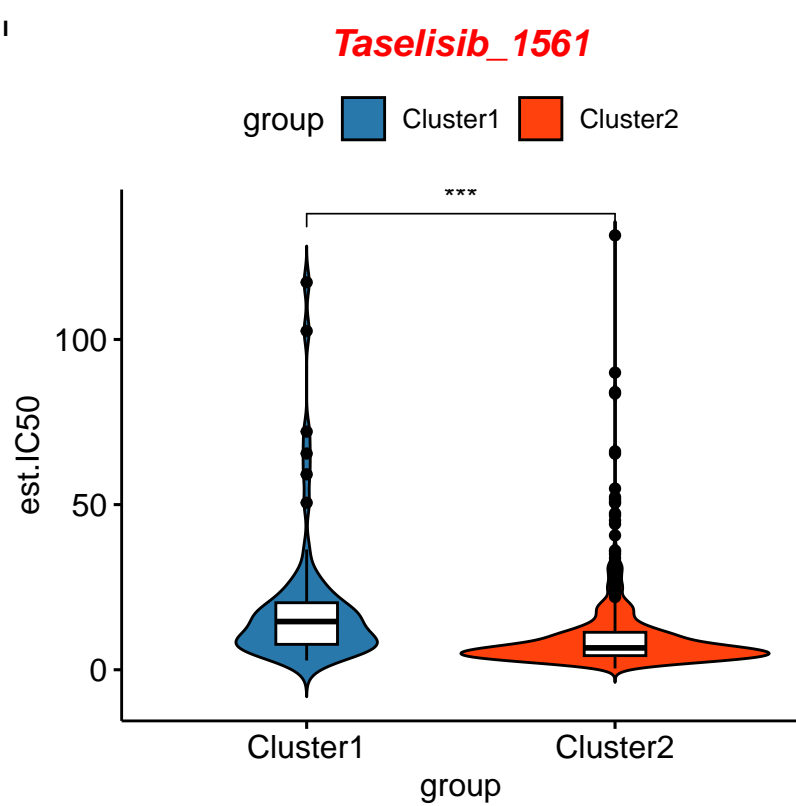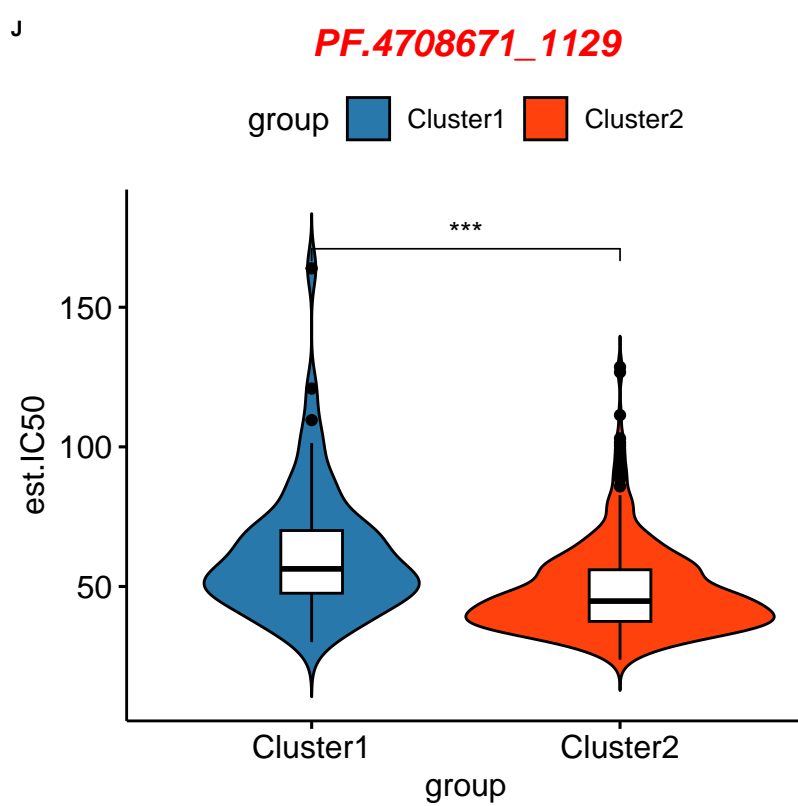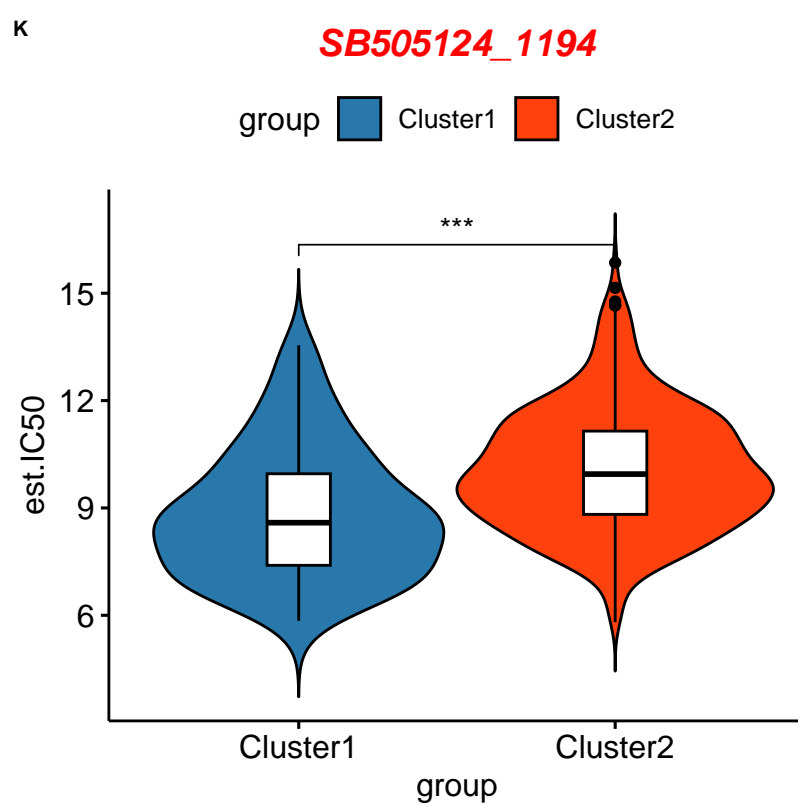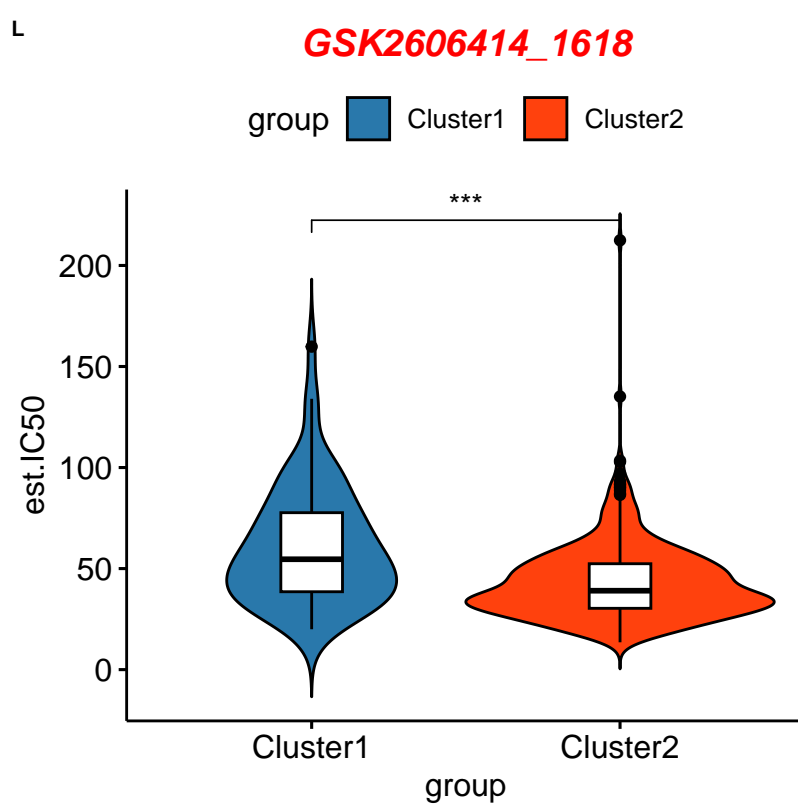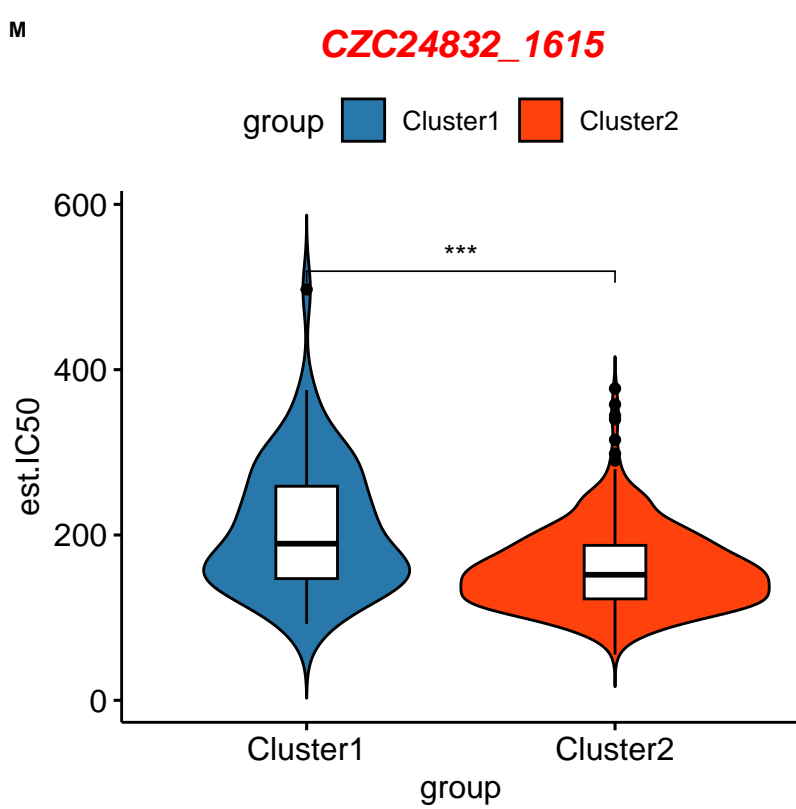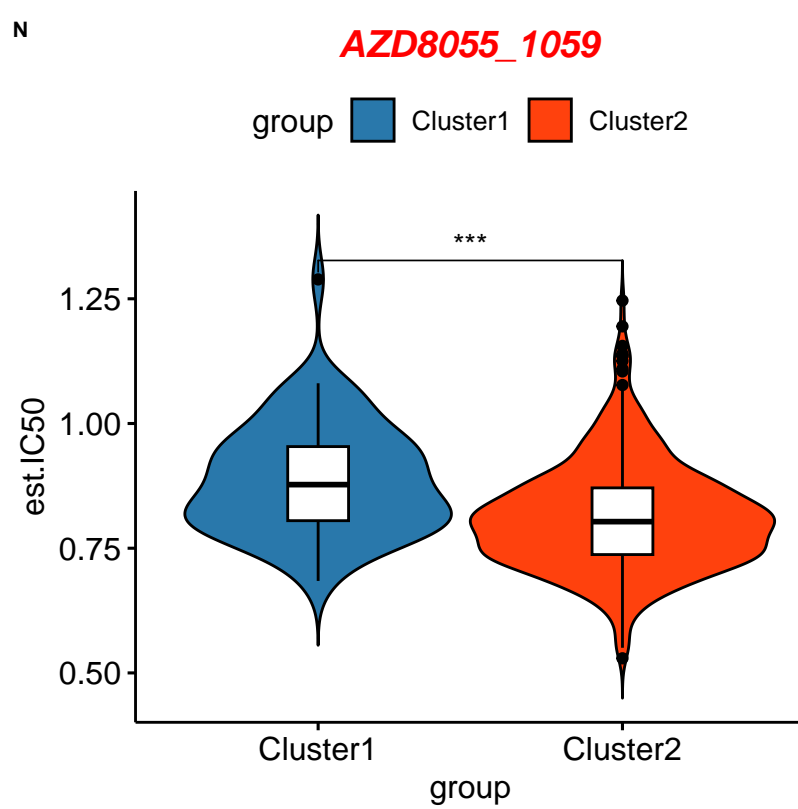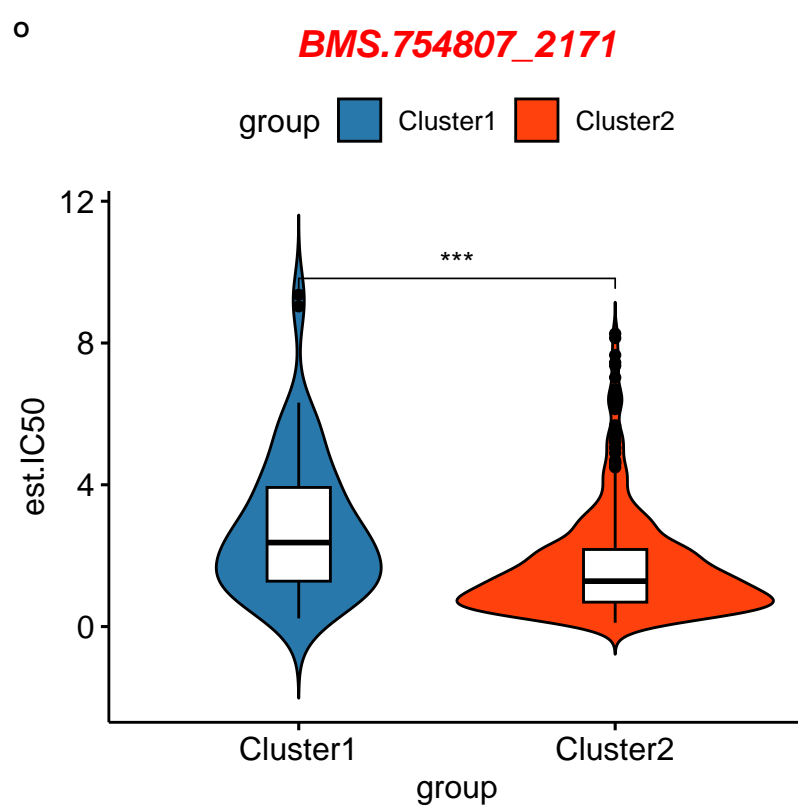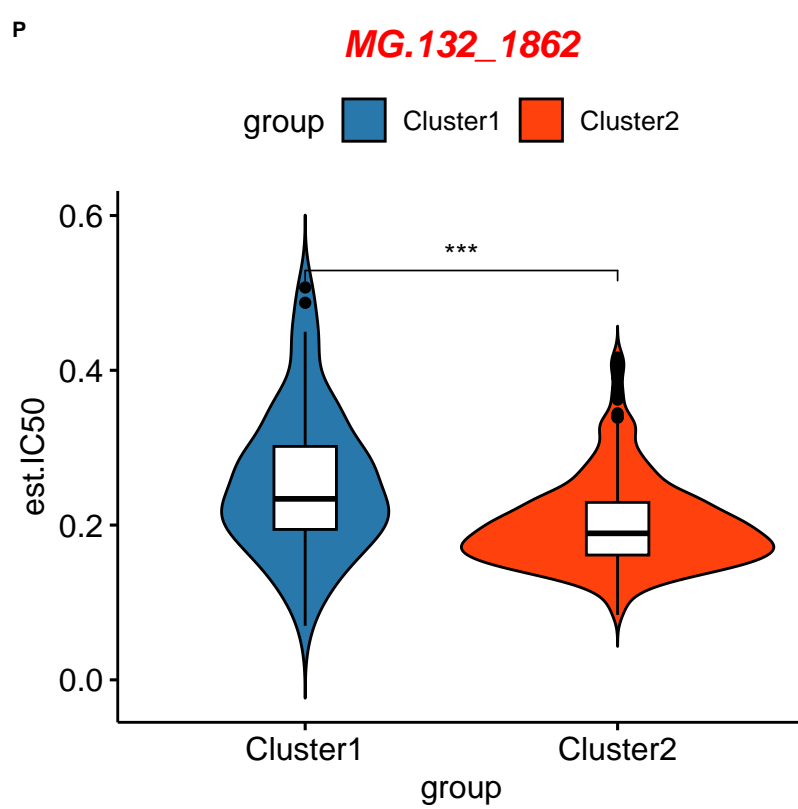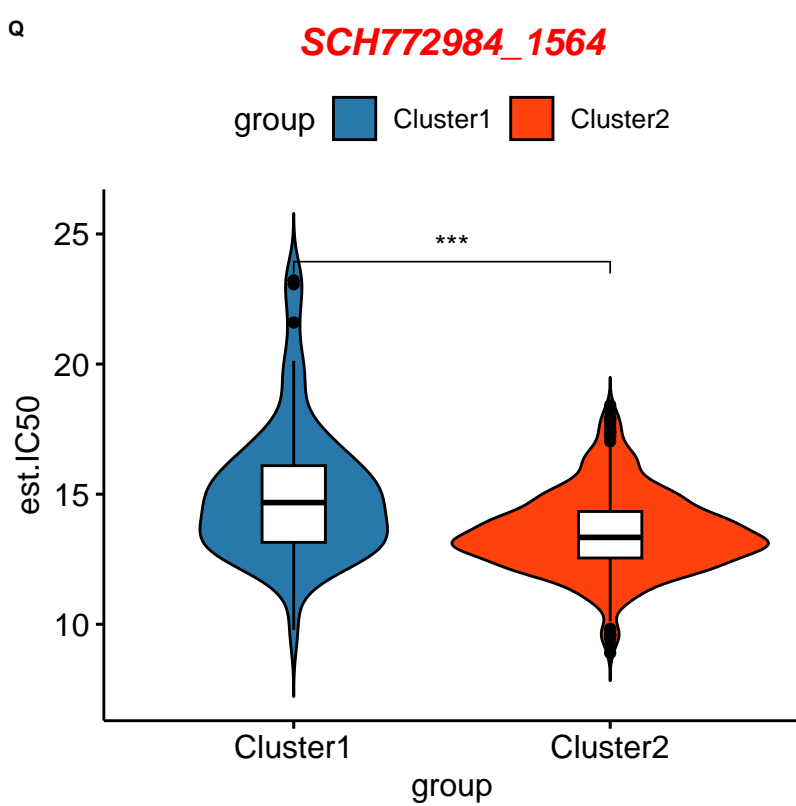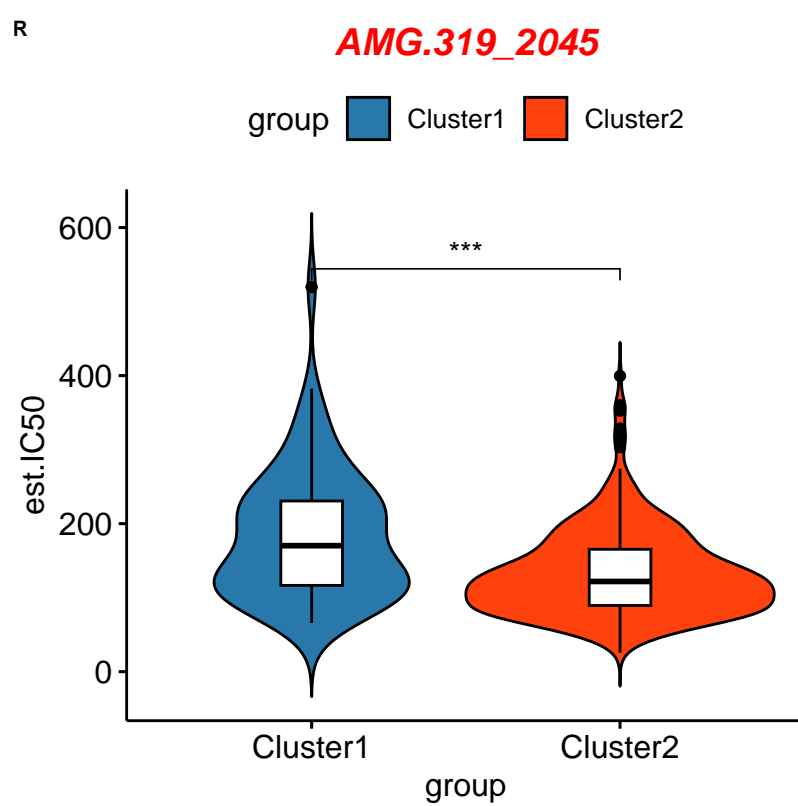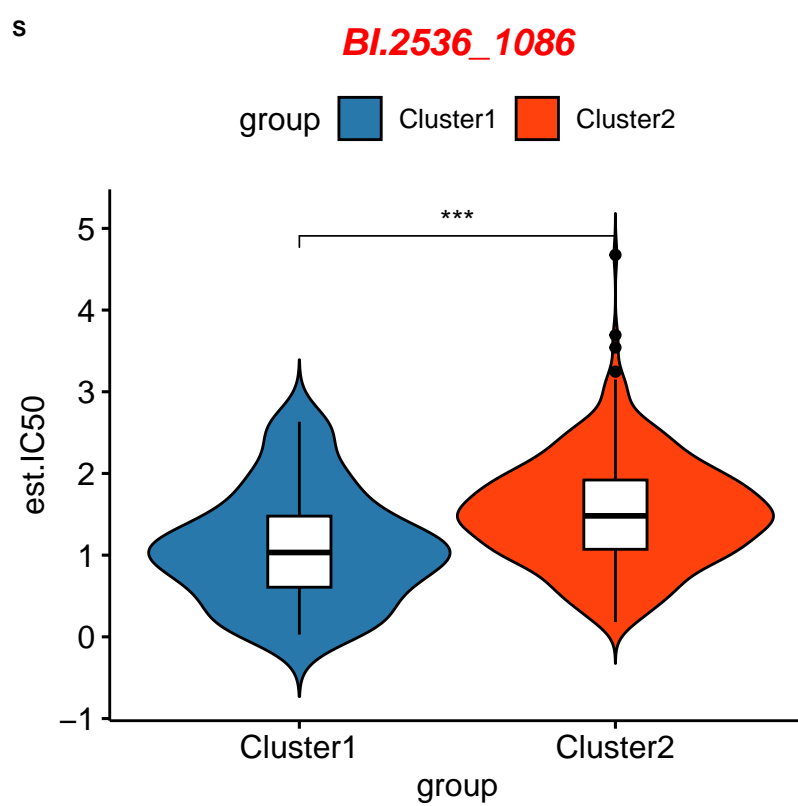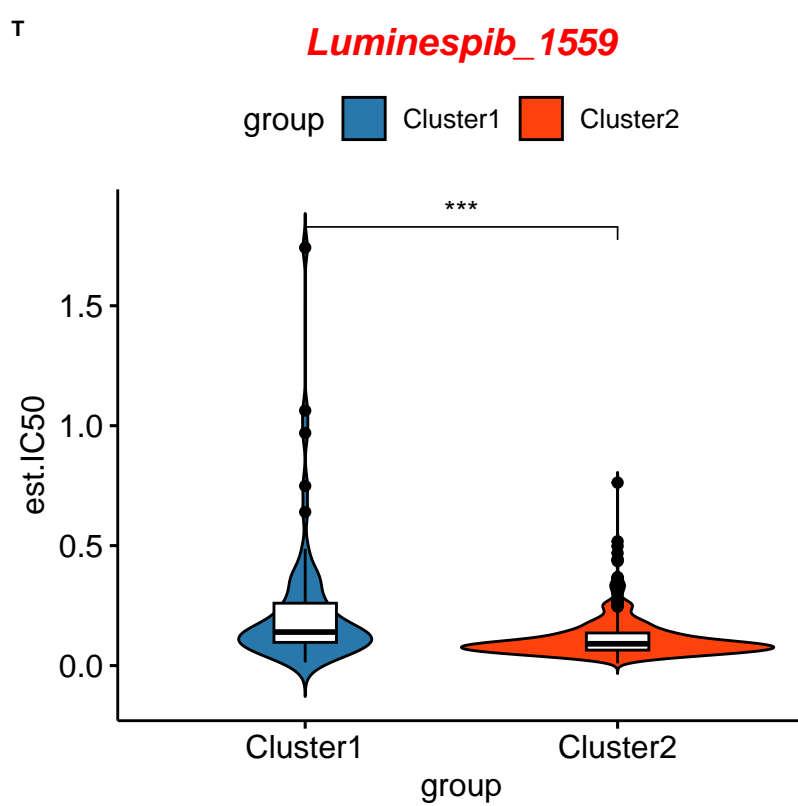

Supplement: Supplementary file 10 — Additional file10 (ZIP 1469 KB) [file 12672_2026_5126_MOESM10_ESM.zip › Figure S10-15 LUAD_oncoPredict_top20.pdf]

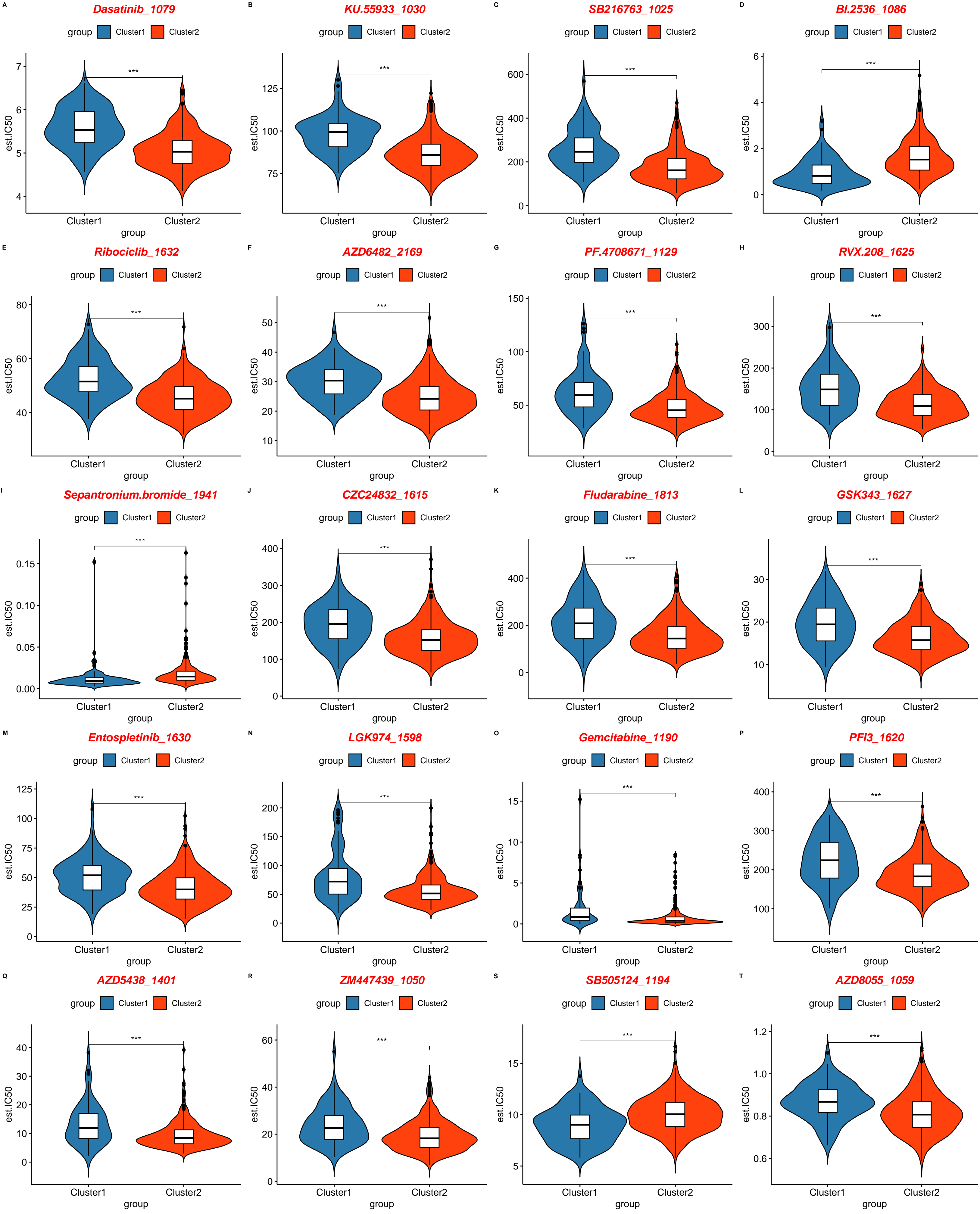

Supplement: Supplementary file 10 — Additional file10 (ZIP 1469 KB) [file 12672_2026_5126_MOESM10_ESM.zip › Figure S10-15 OV_oncoPredict_top20.pdf]

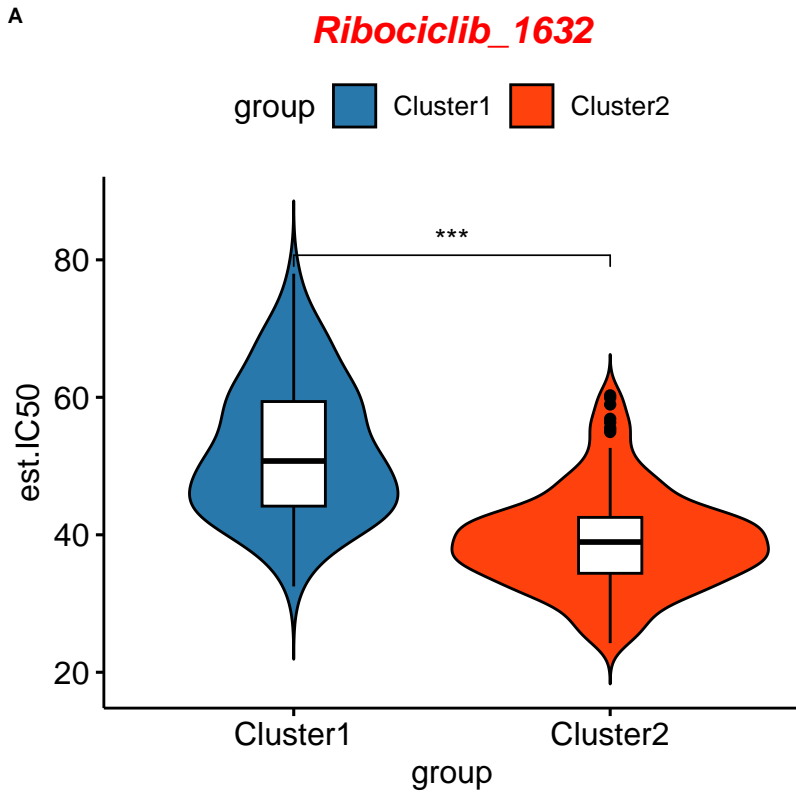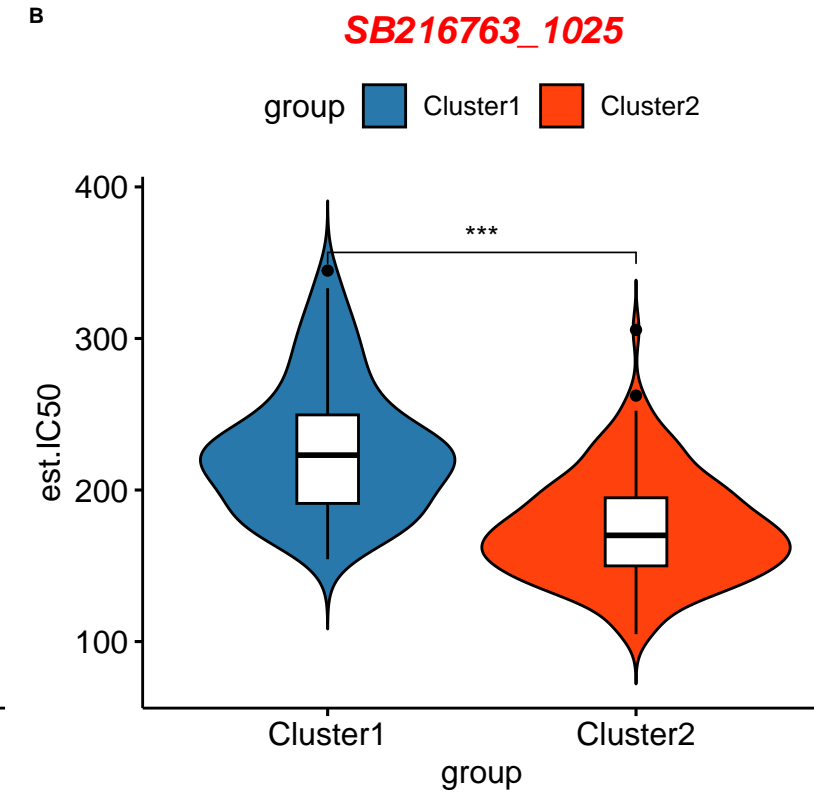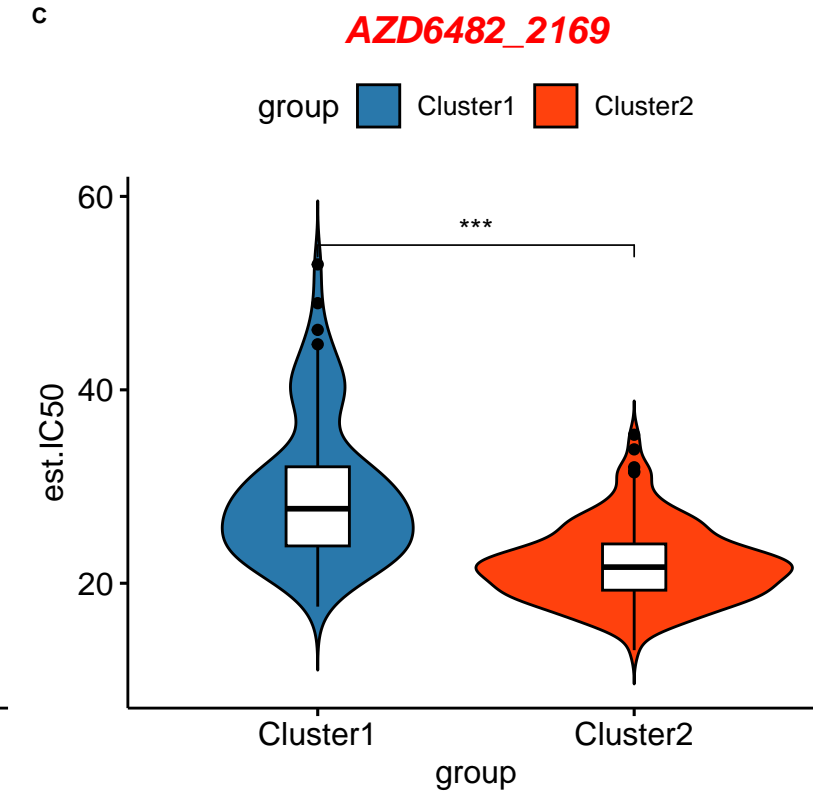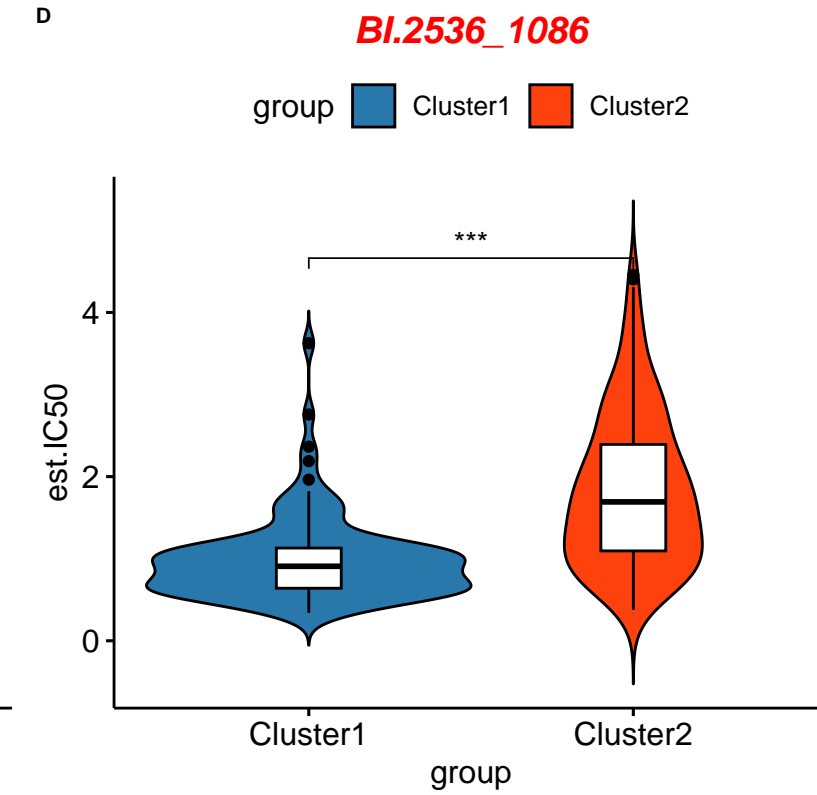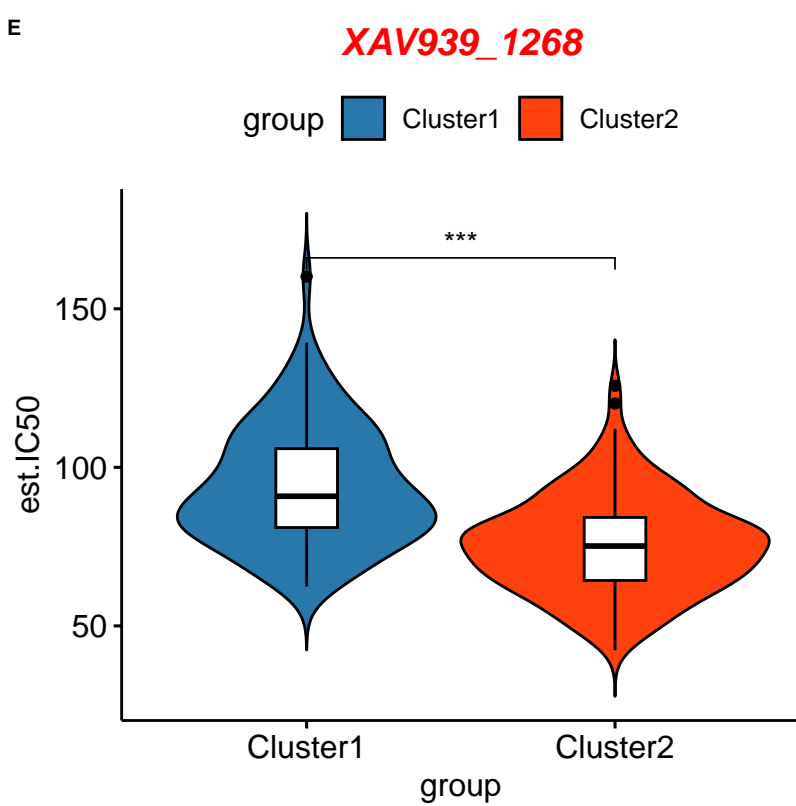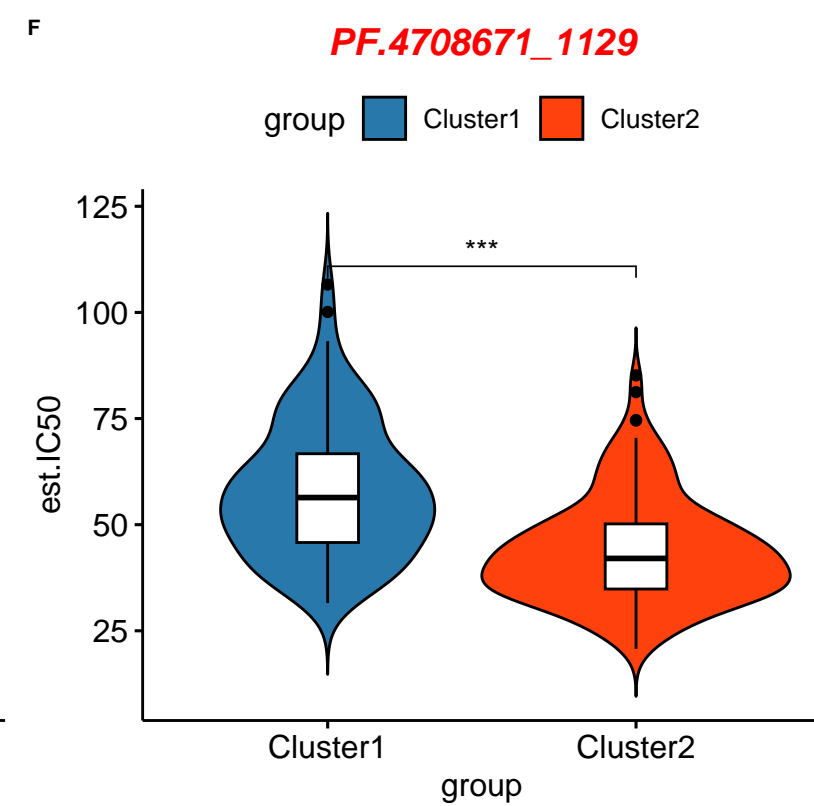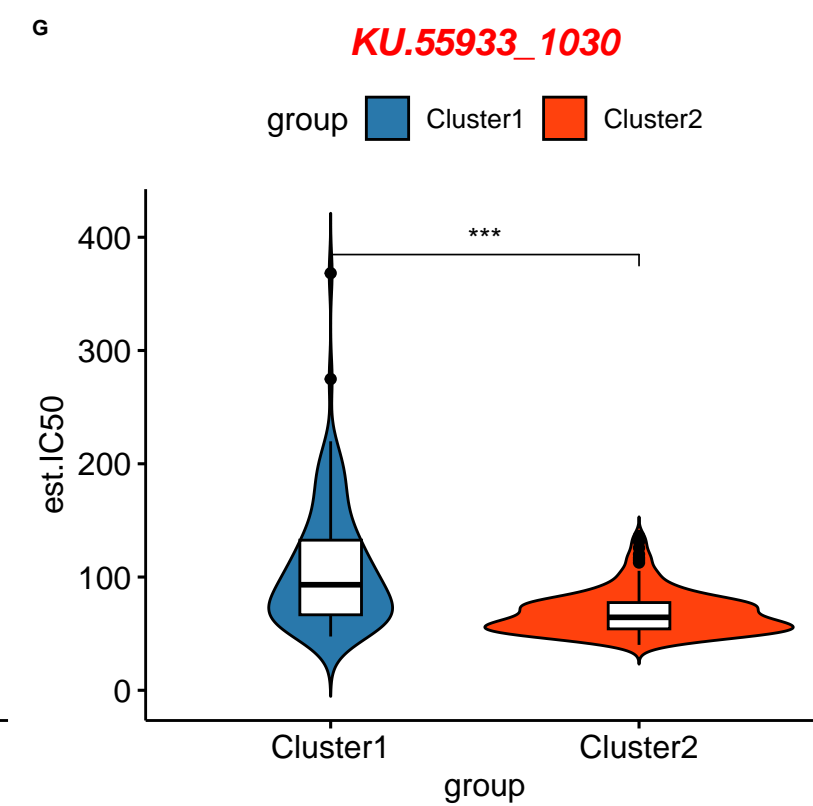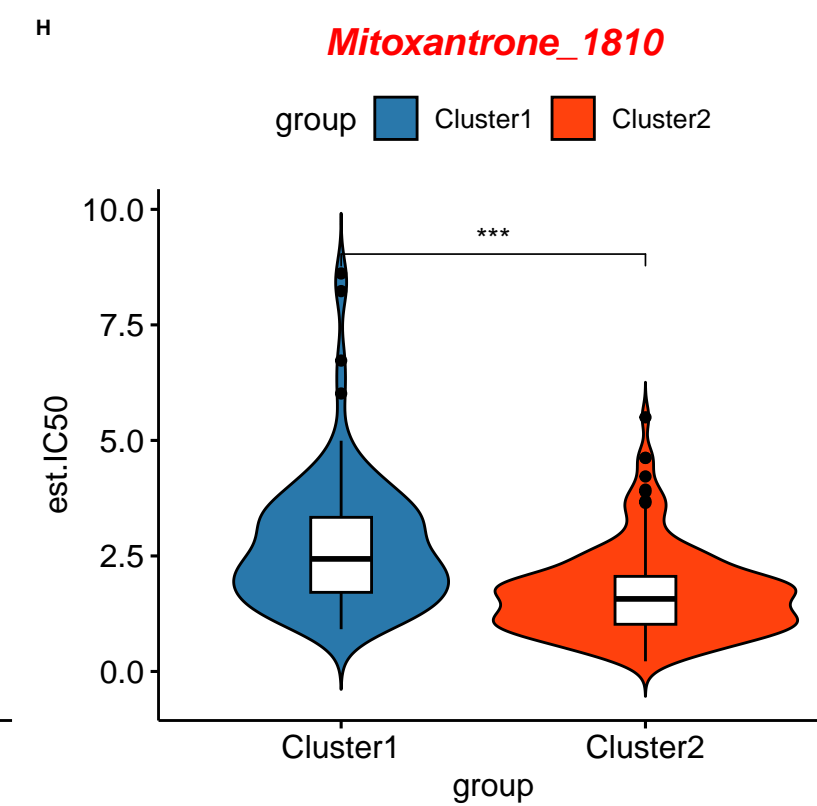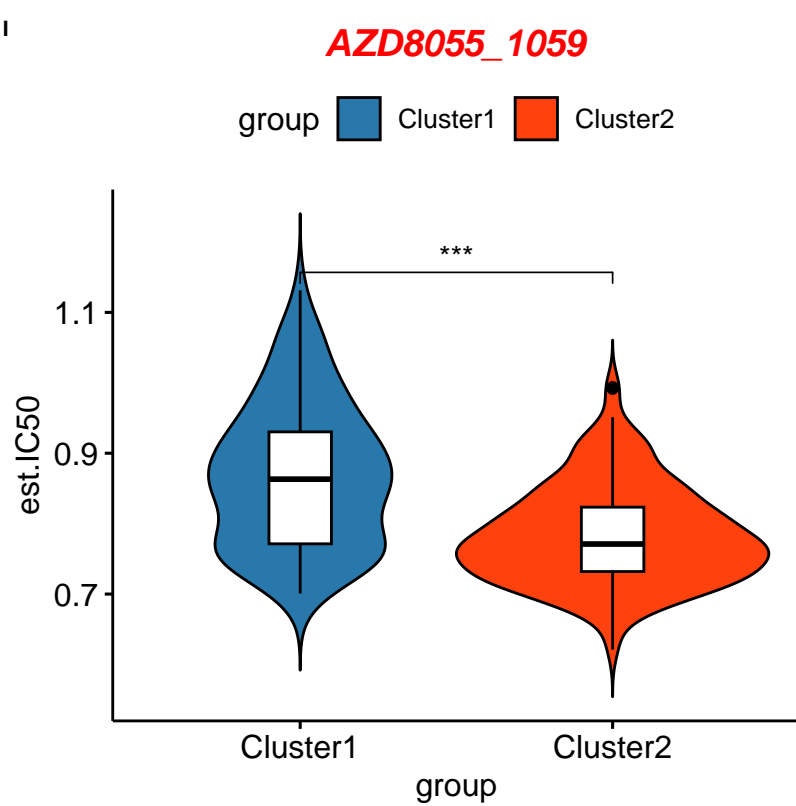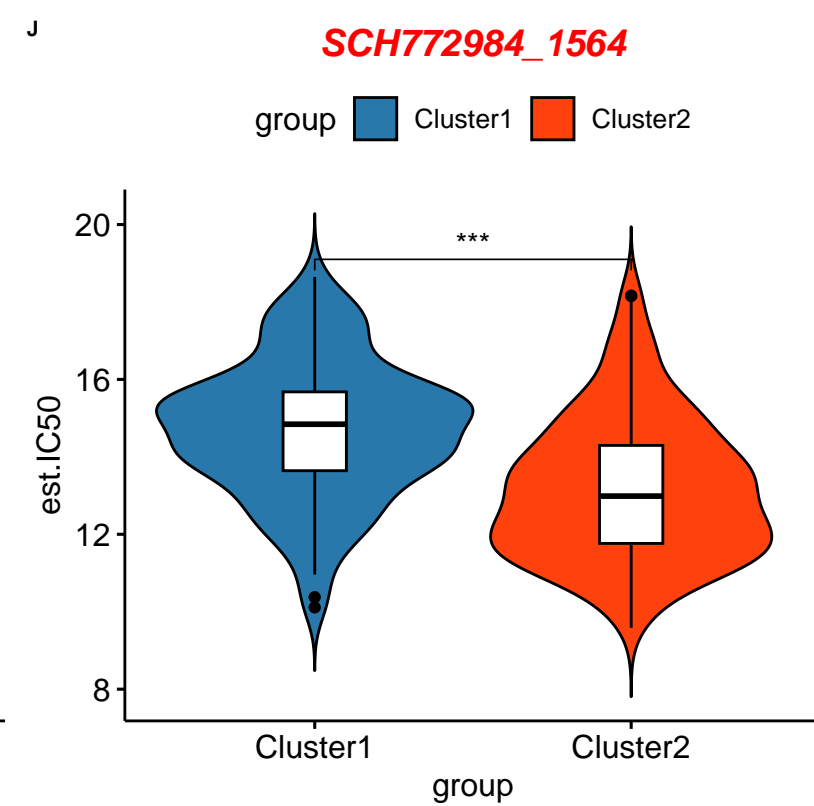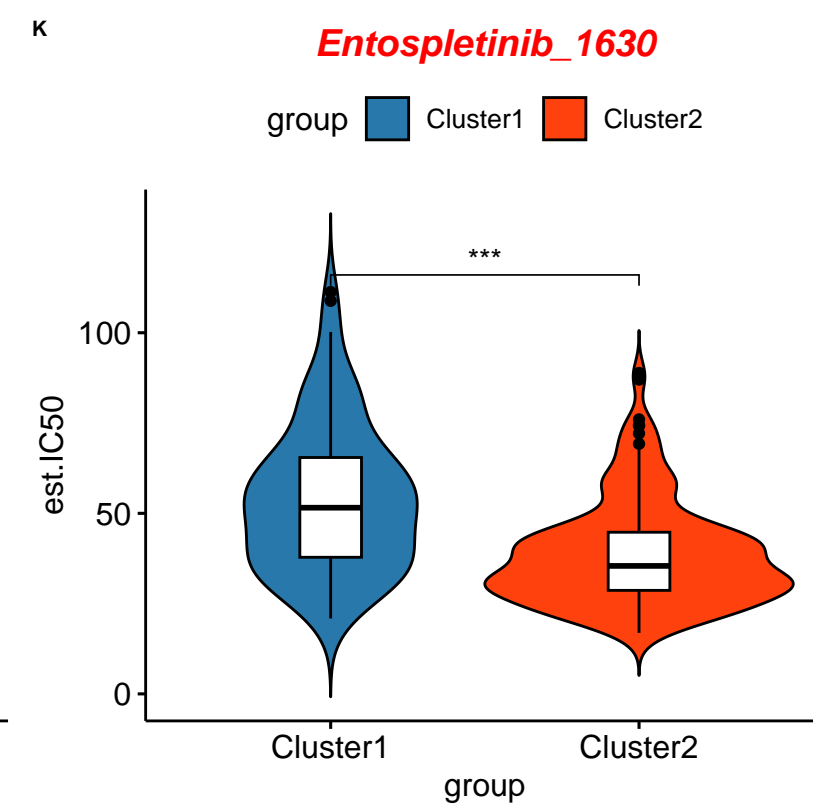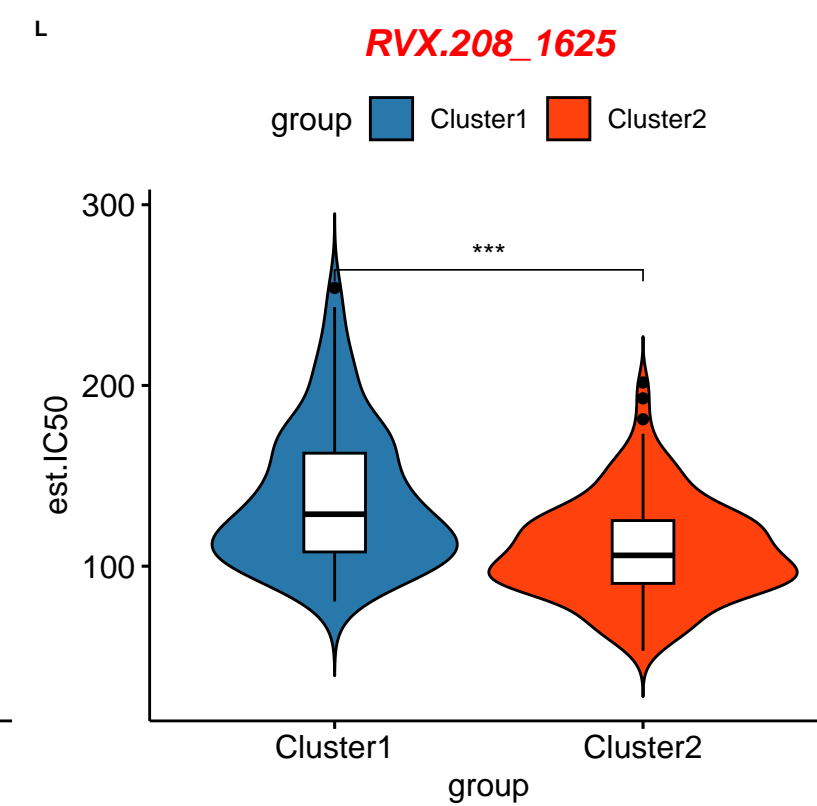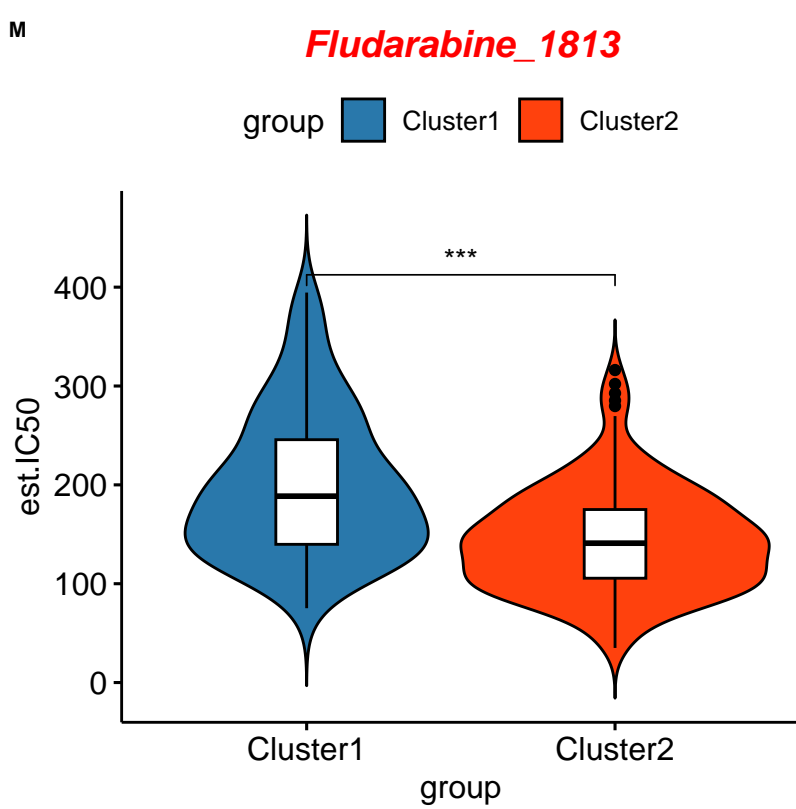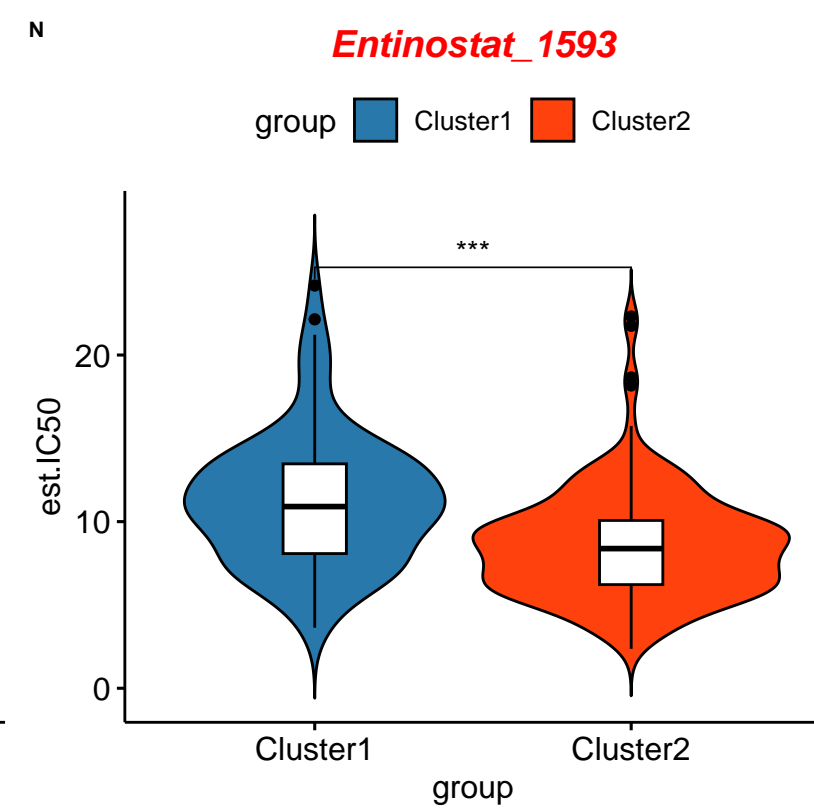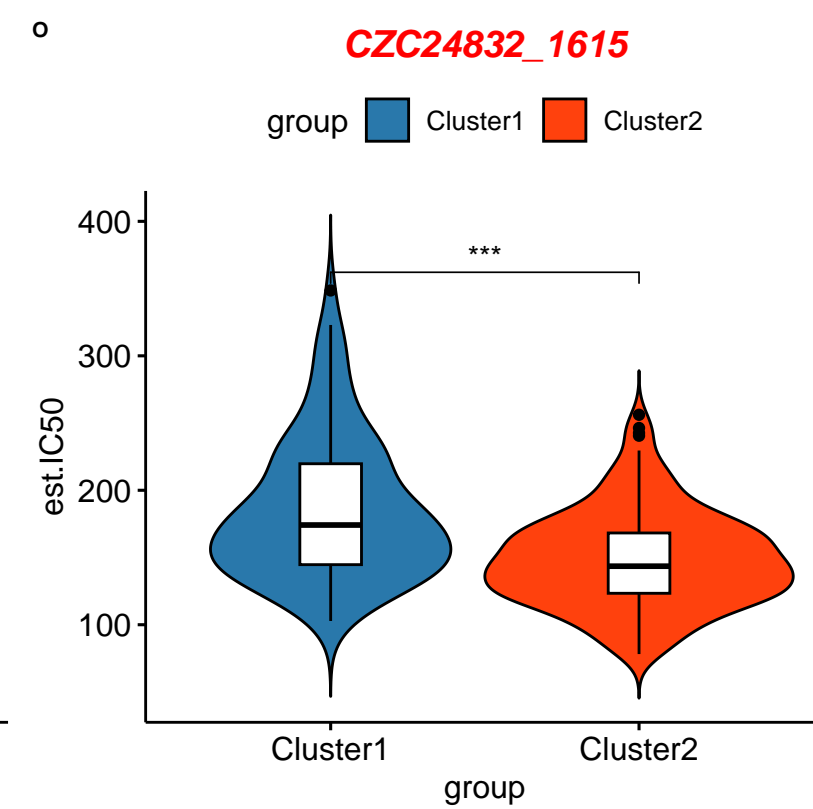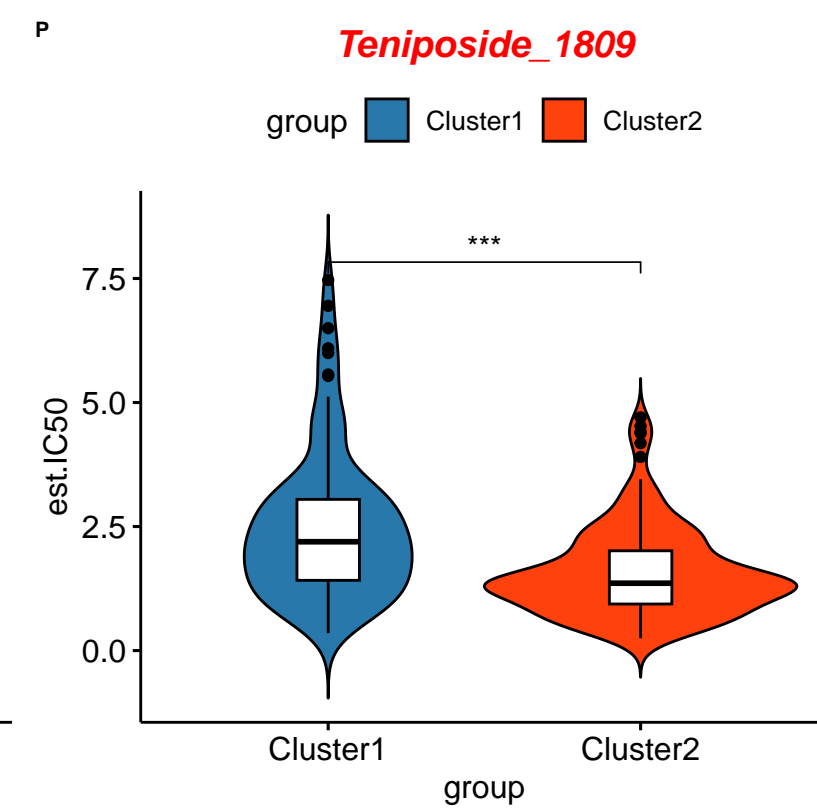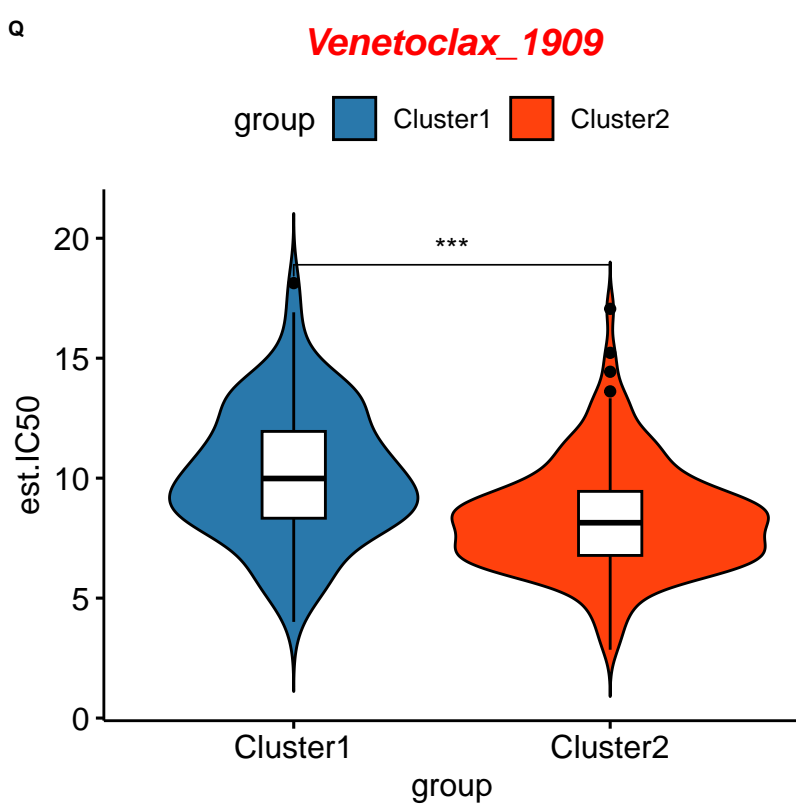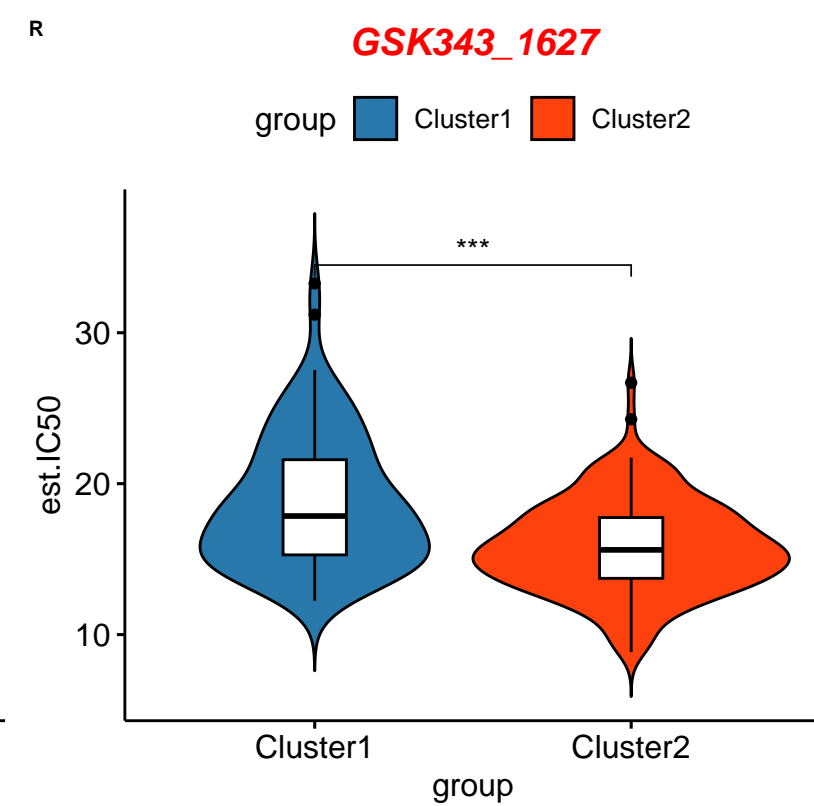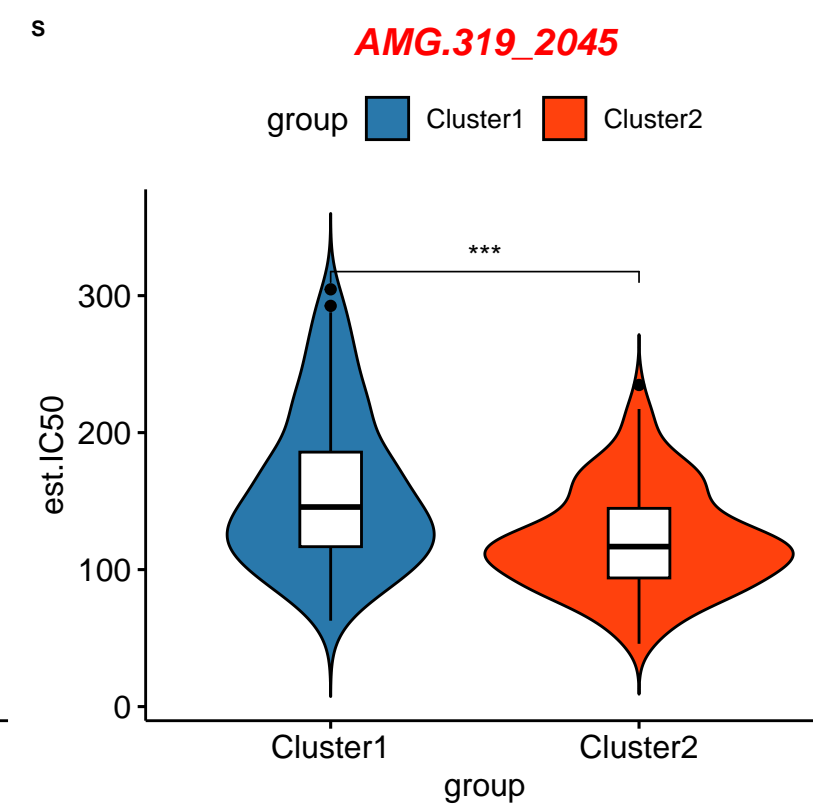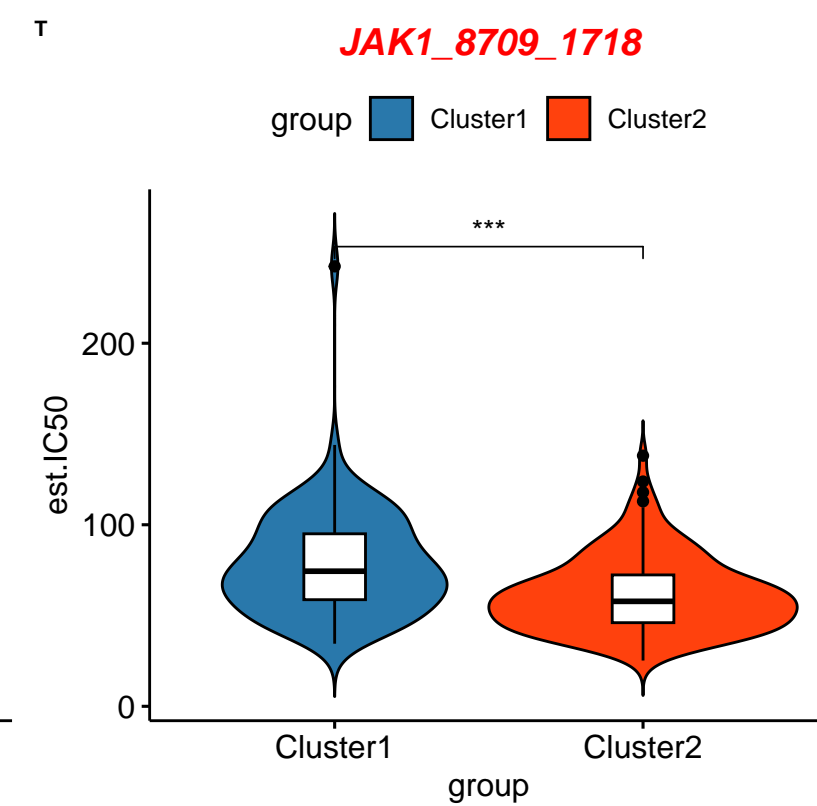

Supplement: Supplementary file 10 — Additional file10 (ZIP 1469 KB) [file 12672_2026_5126_MOESM10_ESM.zip › Figure S10-15 SARC_oncoPredict_top20.pdf]

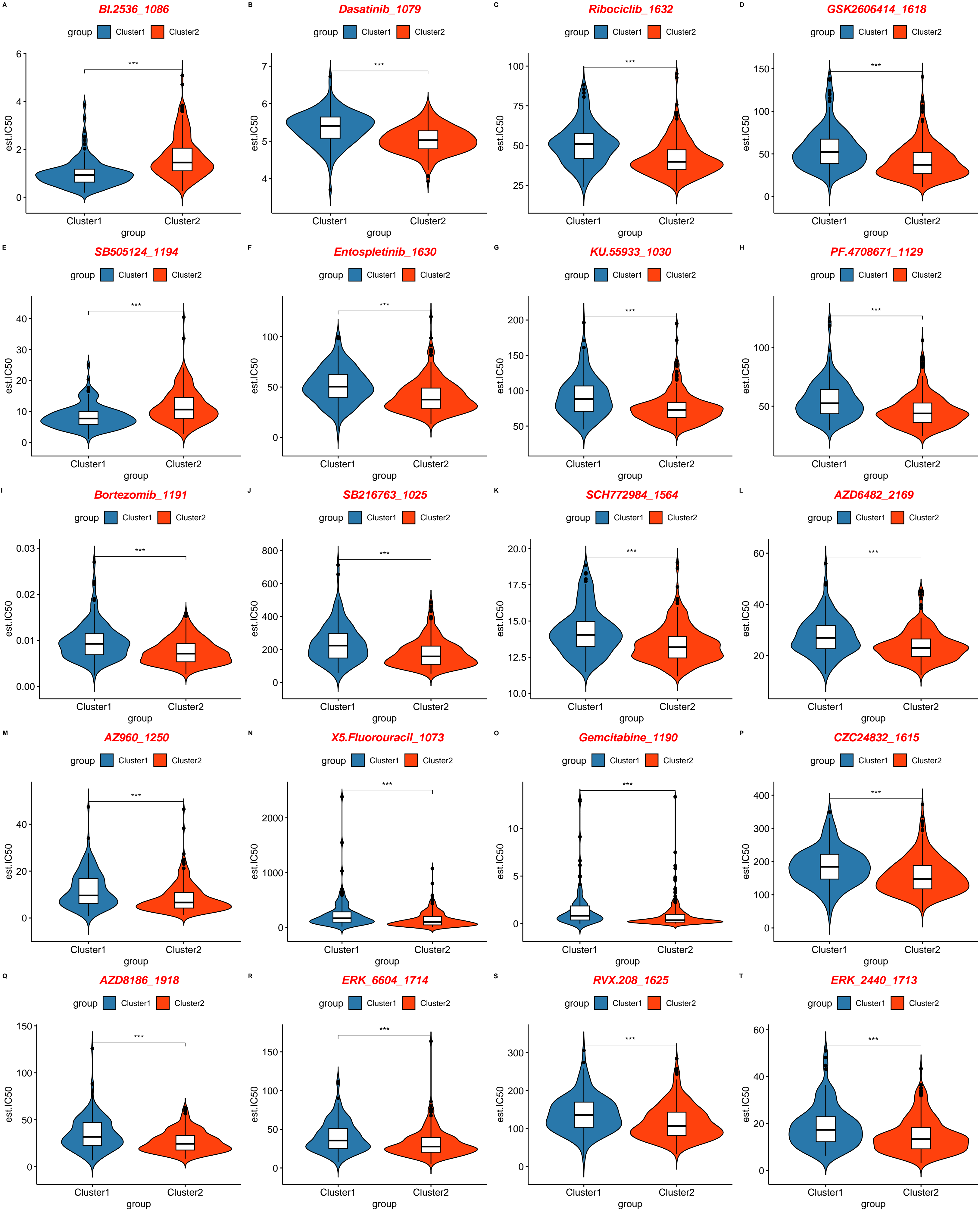

Supplement: Supplementary file 10 — Additional file10 (ZIP 1469 KB) [file 12672_2026_5126_MOESM10_ESM.zip › Figure S10-15 STAD_oncoPredict_top20.pdf]

Cancer Type: ACC

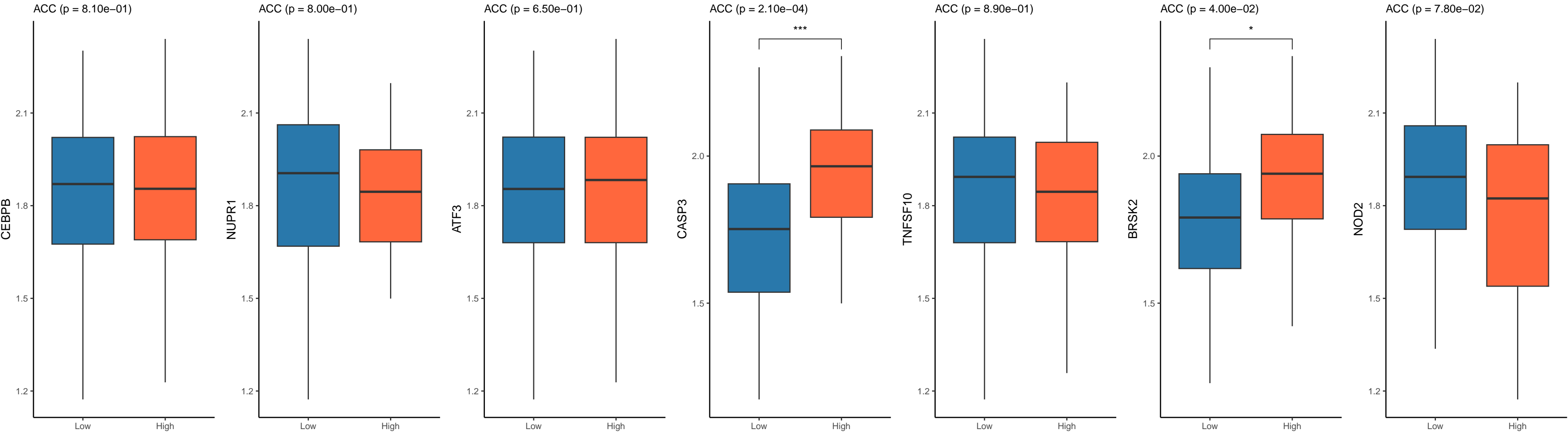

Supplement: Supplementary file 12 — Additional file12 (ZIP 3652 KB) [file 12672_2026_5126_MOESM12_ESM.zip › ACC_combined.pdf]

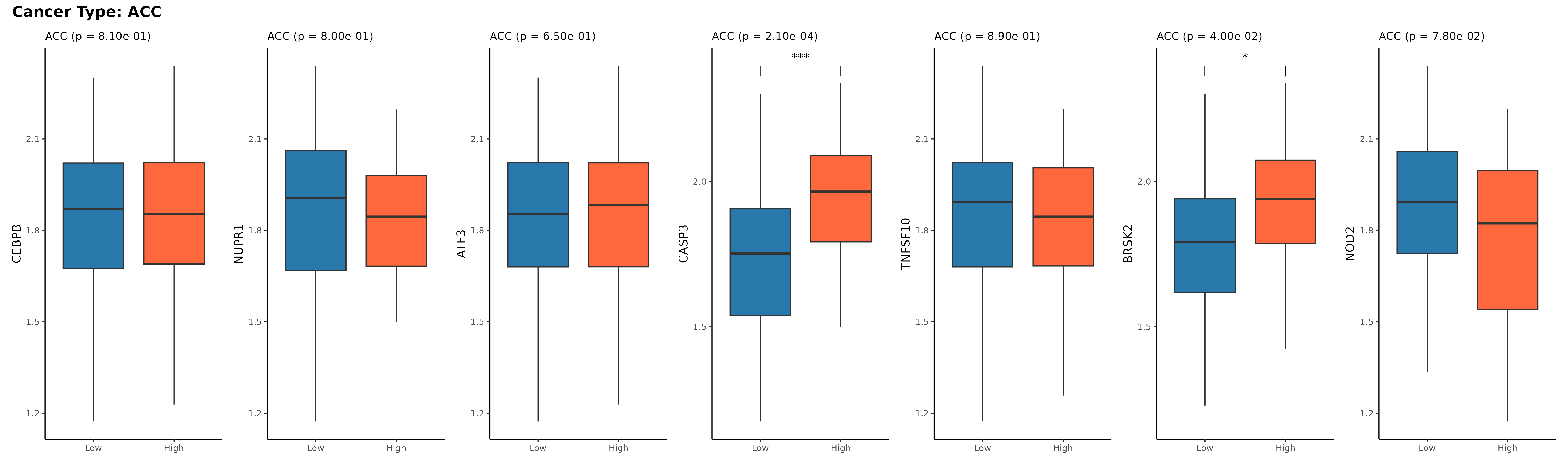

Supplement: Supplementary file 12 — Additional file12 (ZIP 3652 KB) [file 12672_2026_5126_MOESM12_ESM.zip › ACC_combined.png]

Cancer Type: BLCA

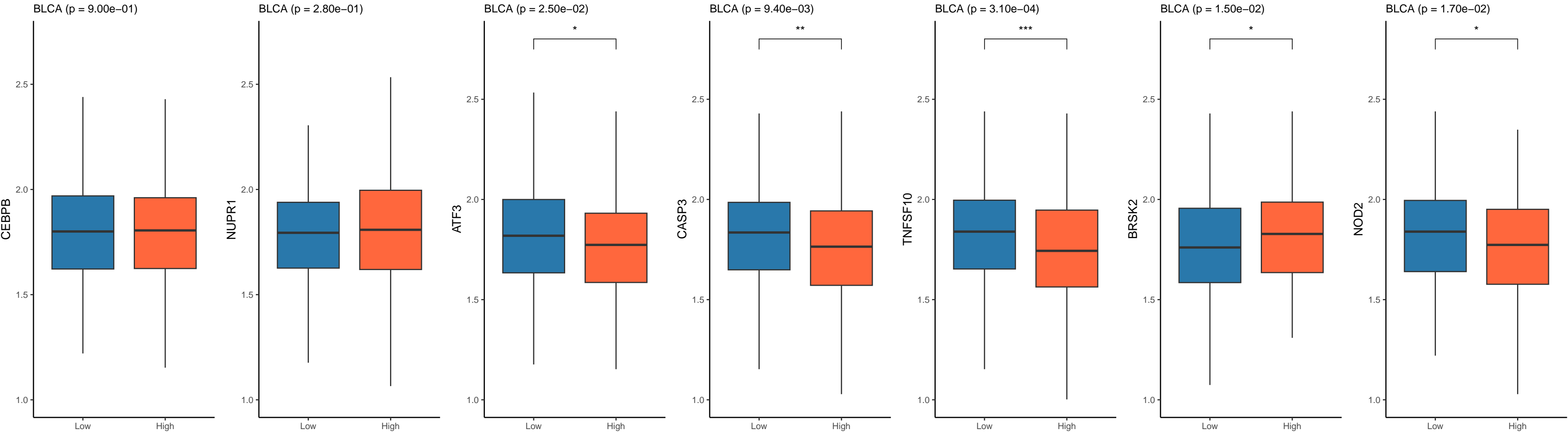

Supplement: Supplementary file 12 — Additional file12 (ZIP 3652 KB) [file 12672_2026_5126_MOESM12_ESM.zip › BLCA_combined.pdf]

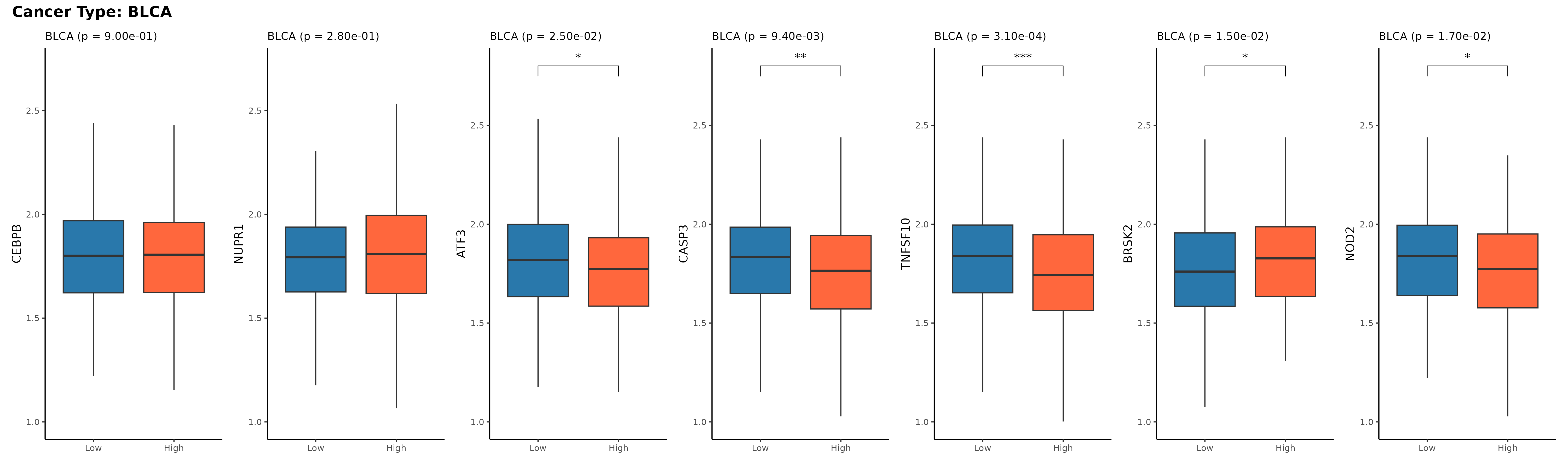

Supplement: Supplementary file 12 — Additional file12 (ZIP 3652 KB) [file 12672_2026_5126_MOESM12_ESM.zip › BLCA_combined.png]

Cancer Type: BRCA

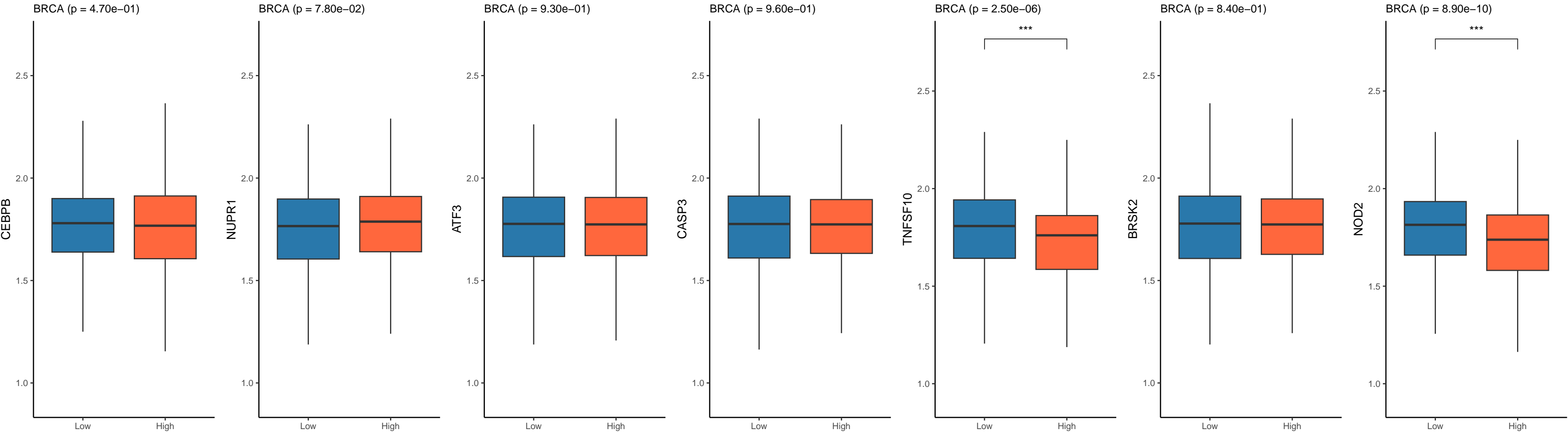

Supplement: Supplementary file 12 — Additional file12 (ZIP 3652 KB) [file 12672_2026_5126_MOESM12_ESM.zip › BRCA_combined.pdf]

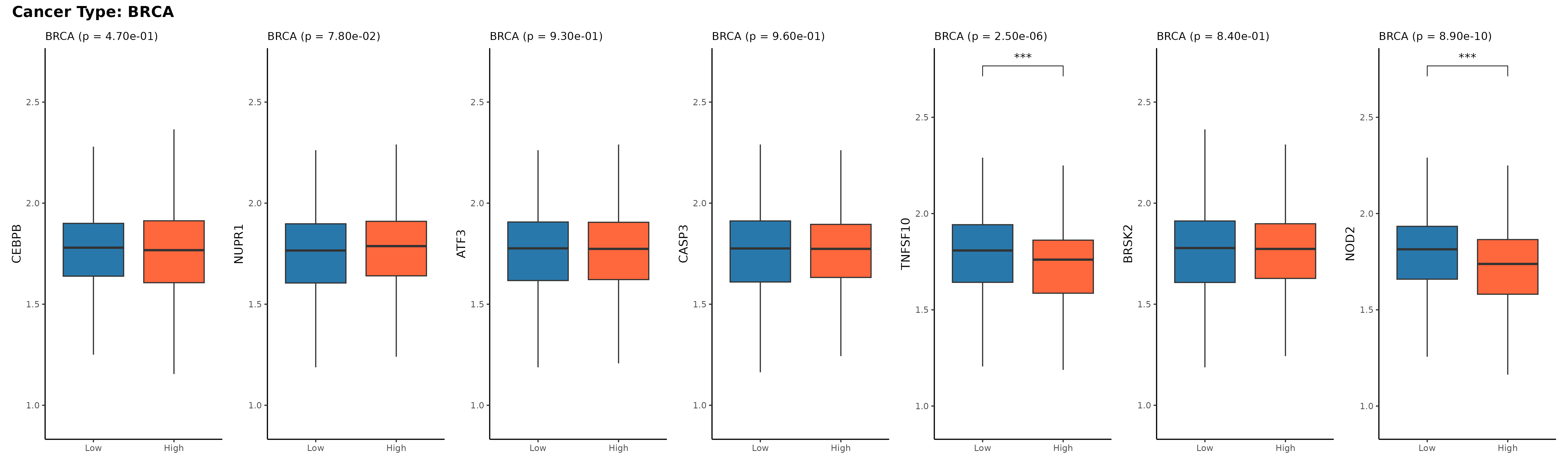

Supplement: Supplementary file 12 — Additional file12 (ZIP 3652 KB) [file 12672_2026_5126_MOESM12_ESM.zip › BRCA_combined.png]

Cancer Type: CESC

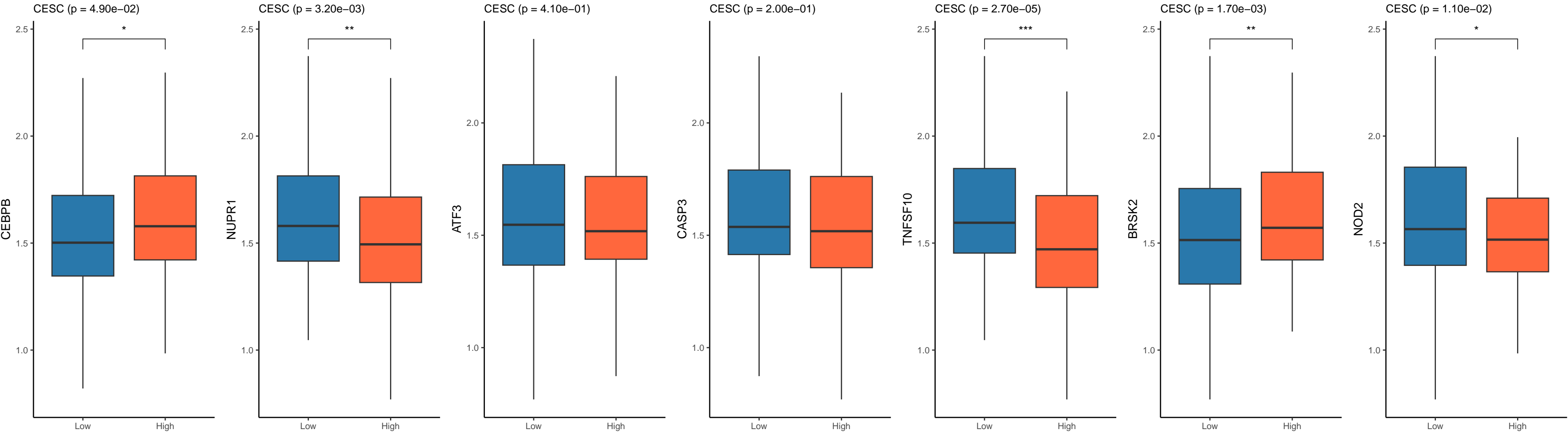

Supplement: Supplementary file 12 — Additional file12 (ZIP 3652 KB) [file 12672_2026_5126_MOESM12_ESM.zip › CESC_combined.pdf]

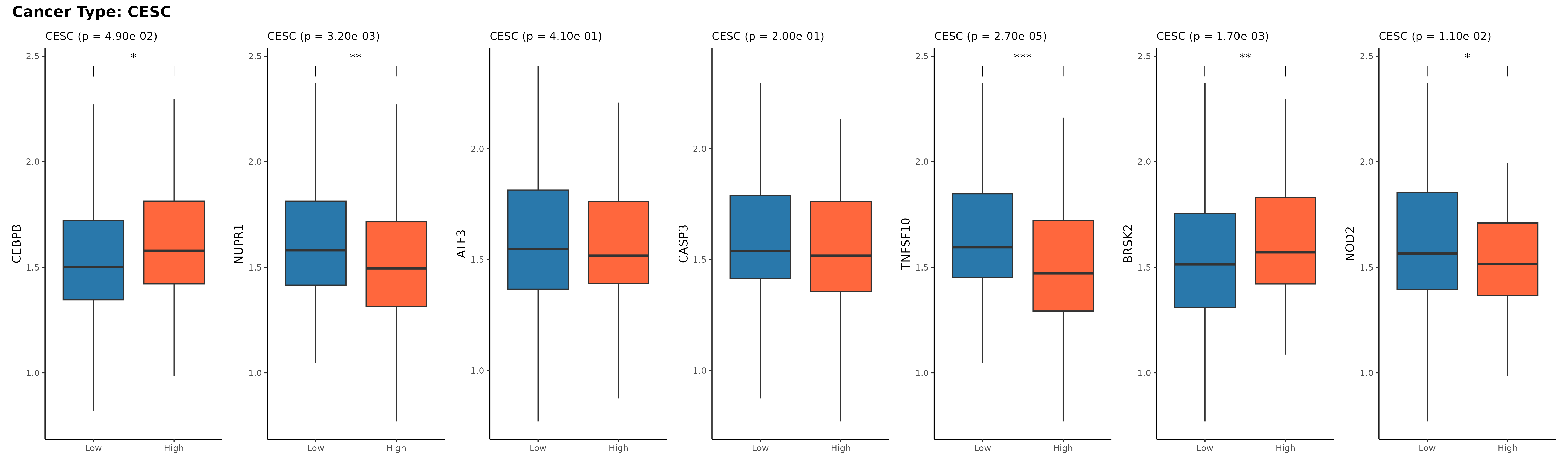

Supplement: Supplementary file 12 — Additional file12 (ZIP 3652 KB) [file 12672_2026_5126_MOESM12_ESM.zip › CESC_combined.png]

Cancer Type: CHOL

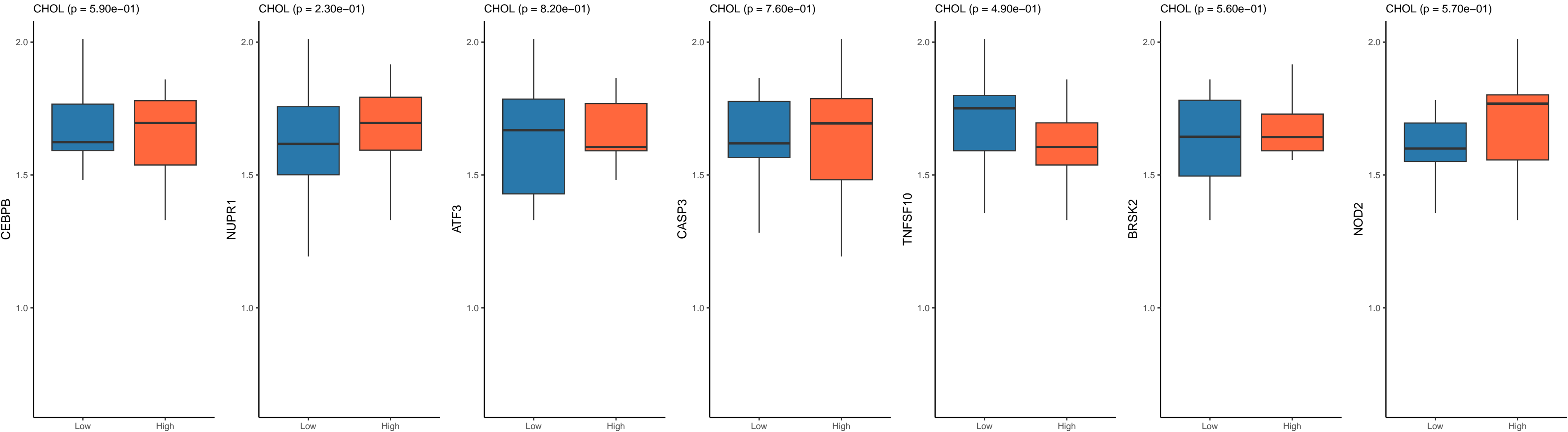

Supplement: Supplementary file 12 — Additional file12 (ZIP 3652 KB) [file 12672_2026_5126_MOESM12_ESM.zip › CHOL_combined.pdf]

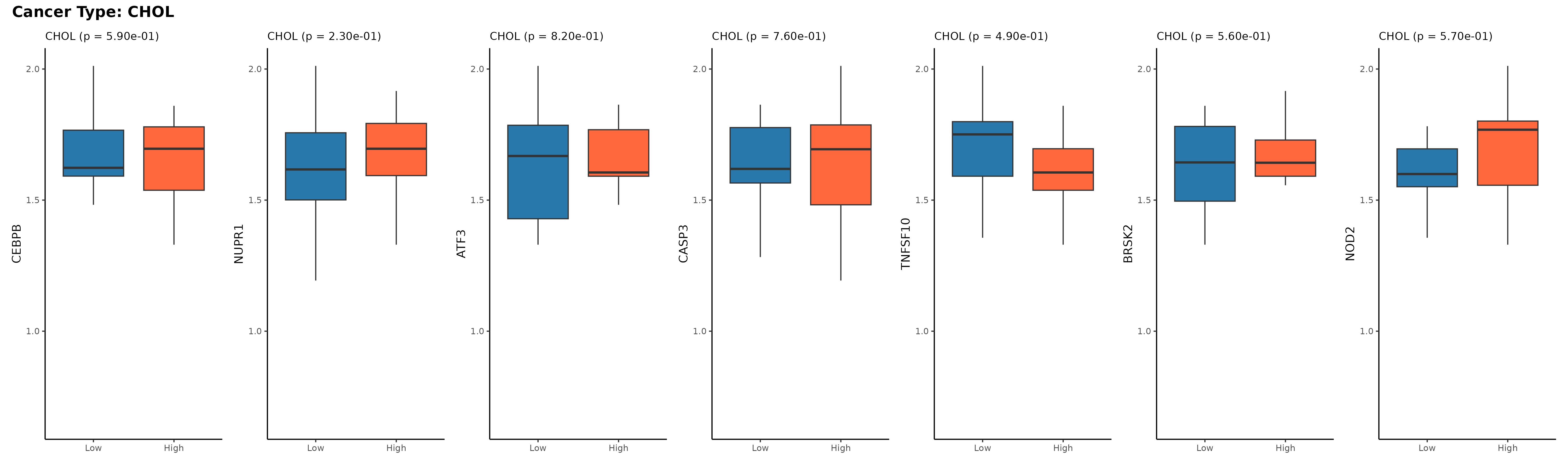

Supplement: Supplementary file 12 — Additional file12 (ZIP 3652 KB) [file 12672_2026_5126_MOESM12_ESM.zip › CHOL_combined.png]

Cancer Type: COAD

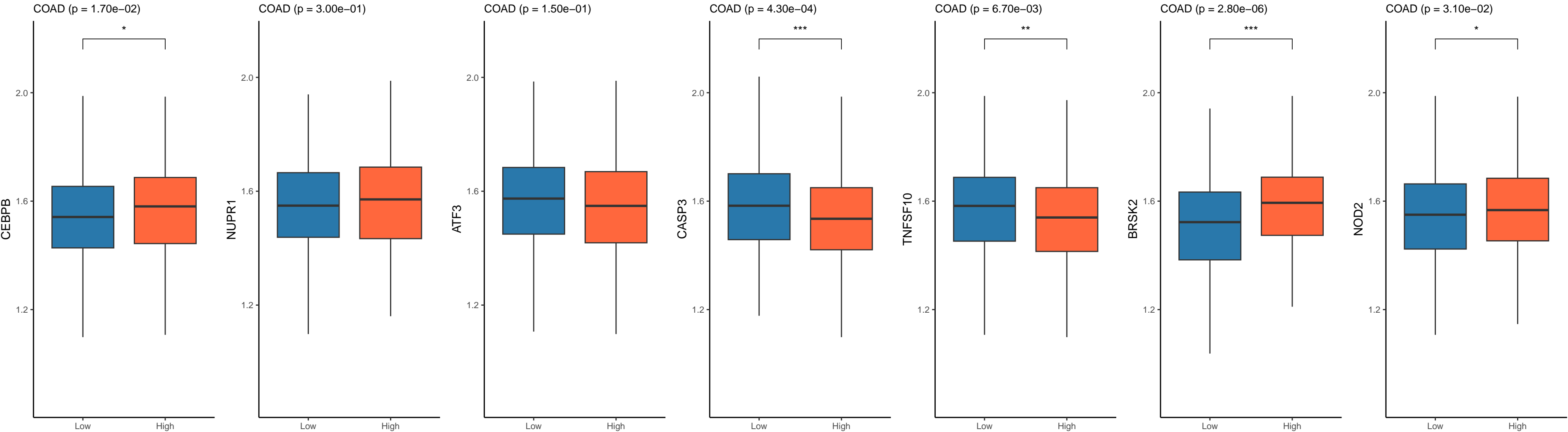

Supplement: Supplementary file 12 — Additional file12 (ZIP 3652 KB) [file 12672_2026_5126_MOESM12_ESM.zip › COAD_combined.pdf]

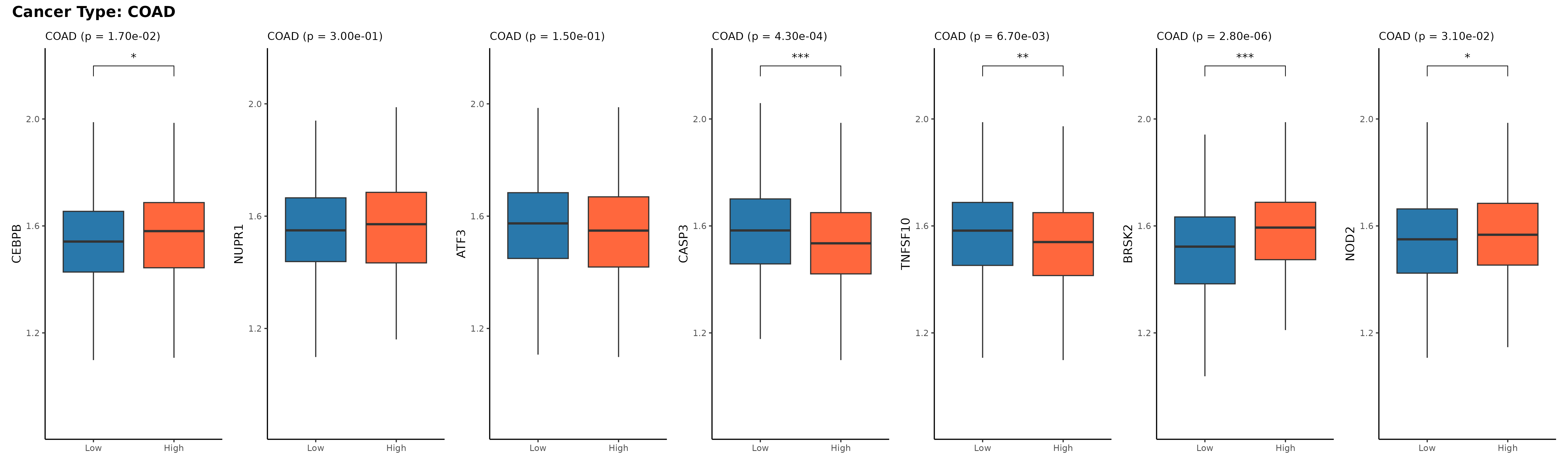

Supplement: Supplementary file 12 — Additional file12 (ZIP 3652 KB) [file 12672_2026_5126_MOESM12_ESM.zip › COAD_combined.png]

Cancer Type: DLBC

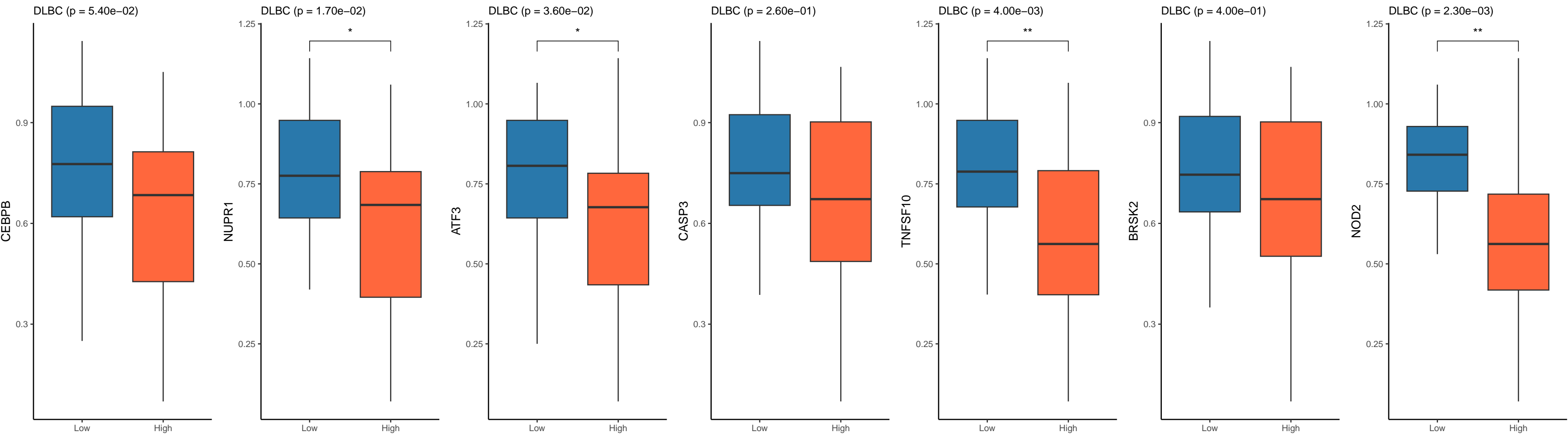

Supplement: Supplementary file 12 — Additional file12 (ZIP 3652 KB) [file 12672_2026_5126_MOESM12_ESM.zip › DLBC_combined.pdf]

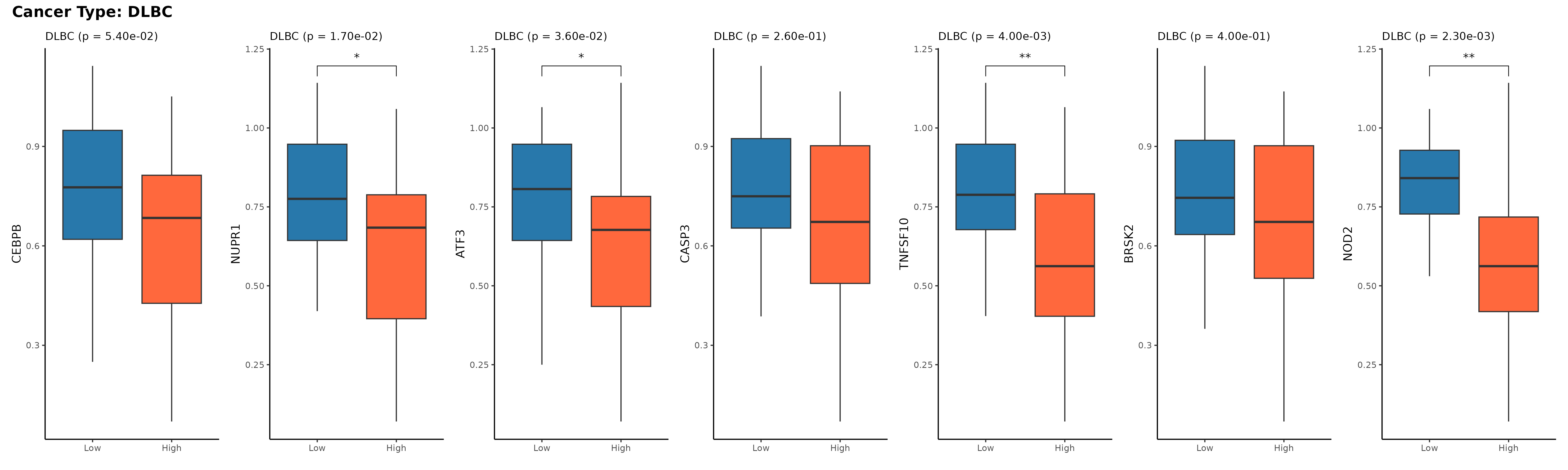

Supplement: Supplementary file 12 — Additional file12 (ZIP 3652 KB) [file 12672_2026_5126_MOESM12_ESM.zip › DLBC_combined.png]

Cancer Type: ESCA

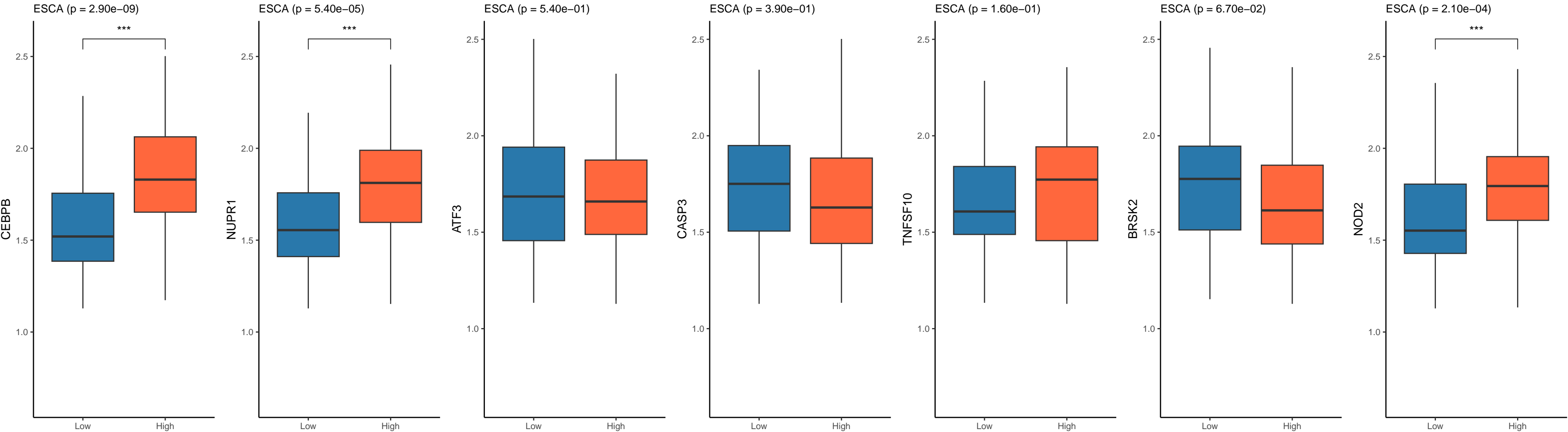

Supplement: Supplementary file 12 — Additional file12 (ZIP 3652 KB) [file 12672_2026_5126_MOESM12_ESM.zip › ESCA_combined.pdf]

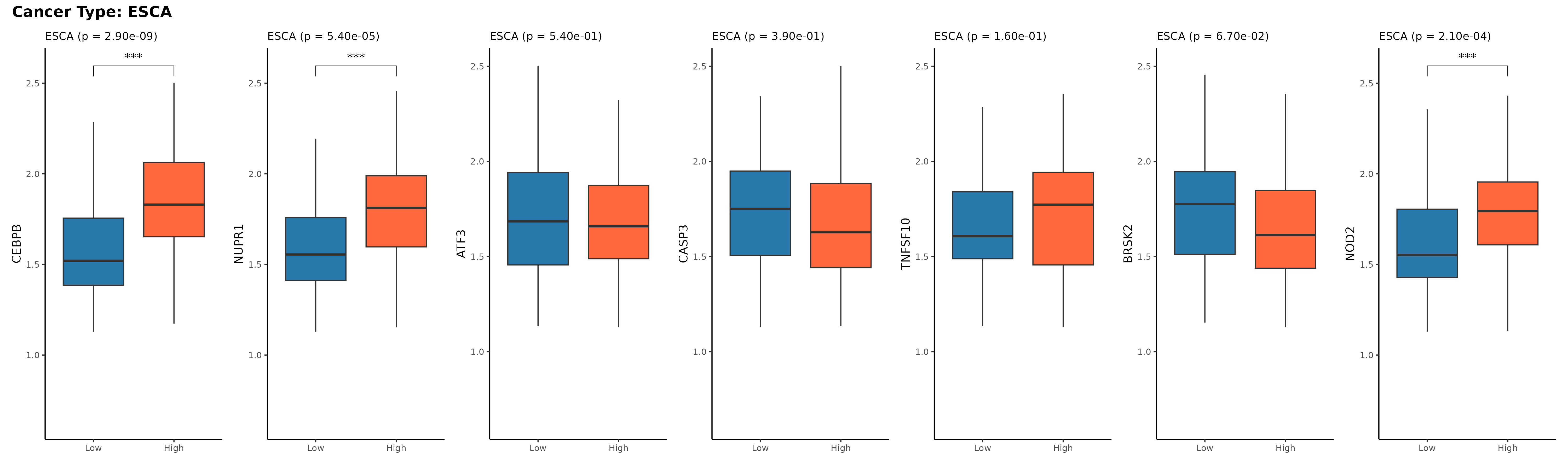

Supplement: Supplementary file 12 — Additional file12 (ZIP 3652 KB) [file 12672_2026_5126_MOESM12_ESM.zip › ESCA_combined.png]

Cancer Type: GBM

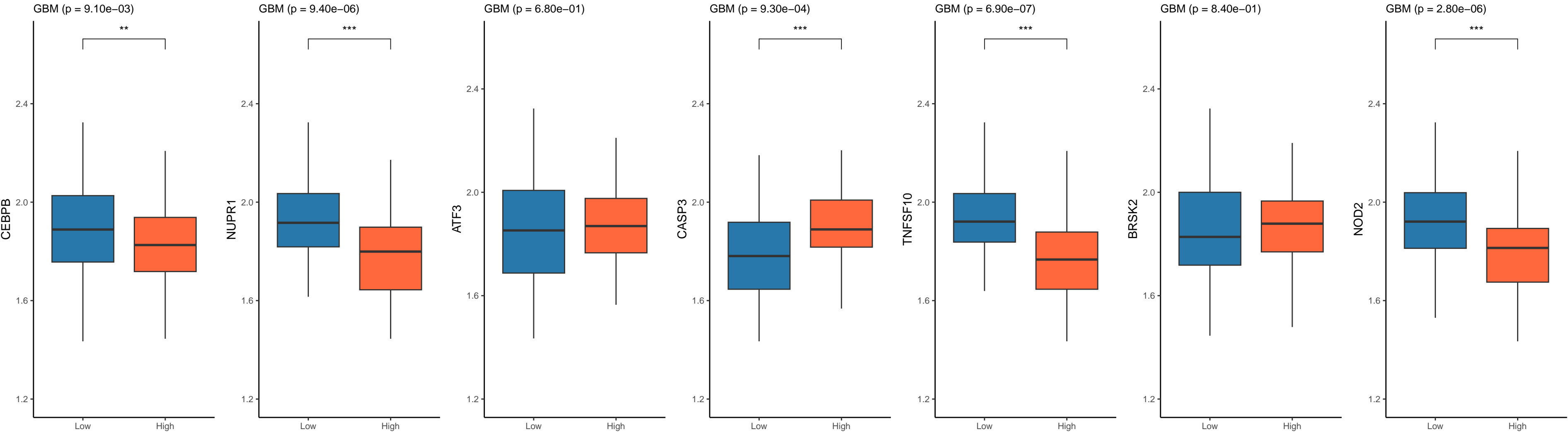

Supplement: Supplementary file 12 — Additional file12 (ZIP 3652 KB) [file 12672_2026_5126_MOESM12_ESM.zip › GBM_combined.pdf]

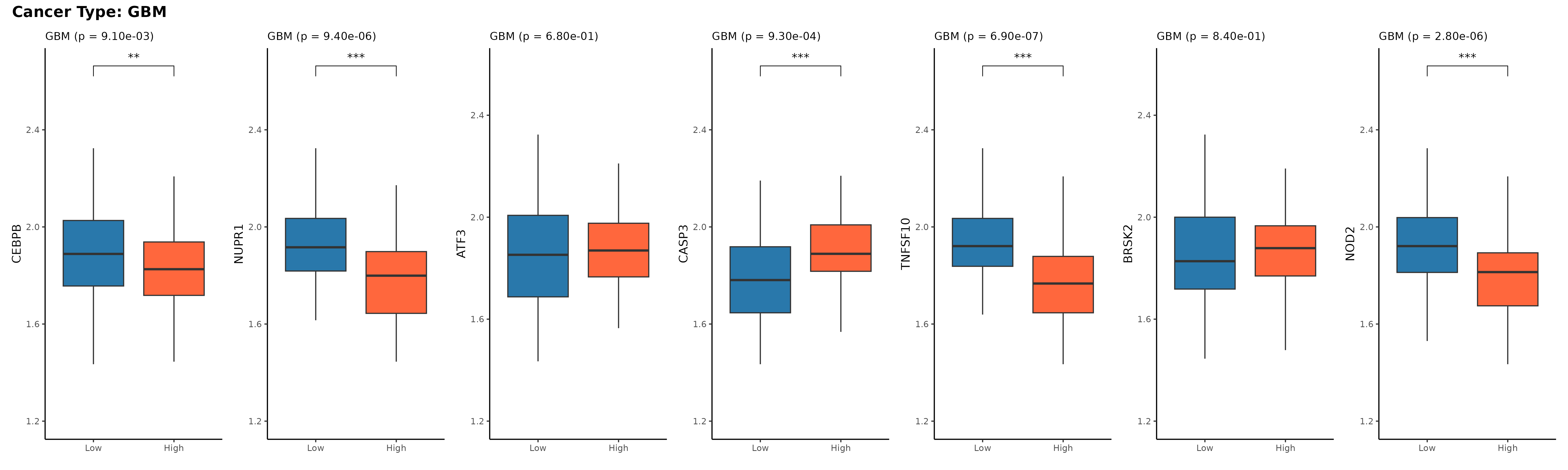

Supplement: Supplementary file 12 — Additional file12 (ZIP 3652 KB) [file 12672_2026_5126_MOESM12_ESM.zip › GBM_combined.png]

Cancer Type: HNSC

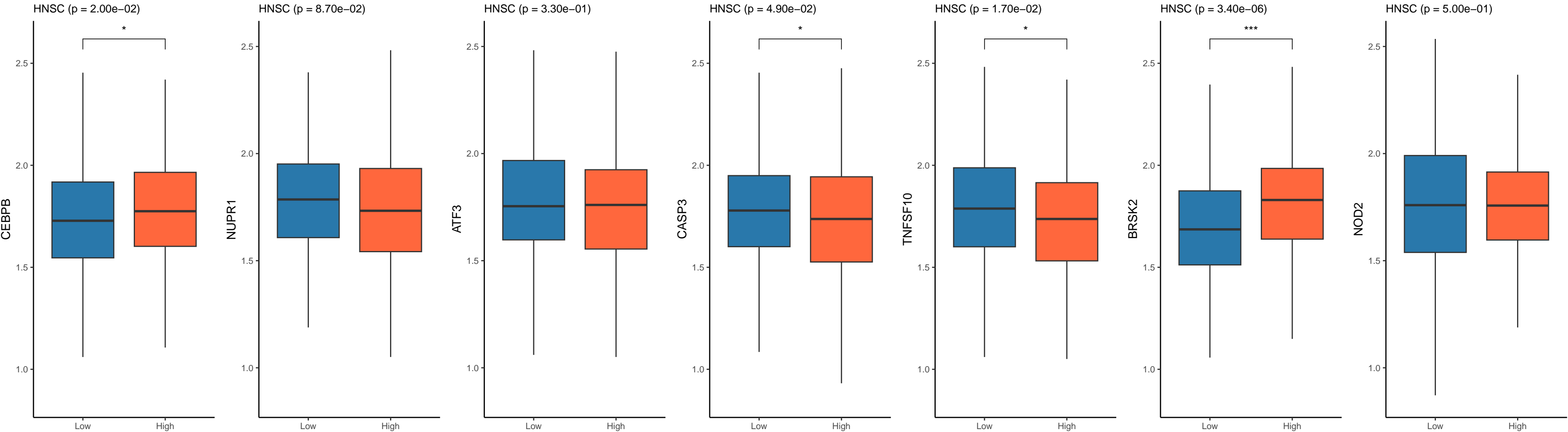

Supplement: Supplementary file 12 — Additional file12 (ZIP 3652 KB) [file 12672_2026_5126_MOESM12_ESM.zip › HNSC_combined.pdf]

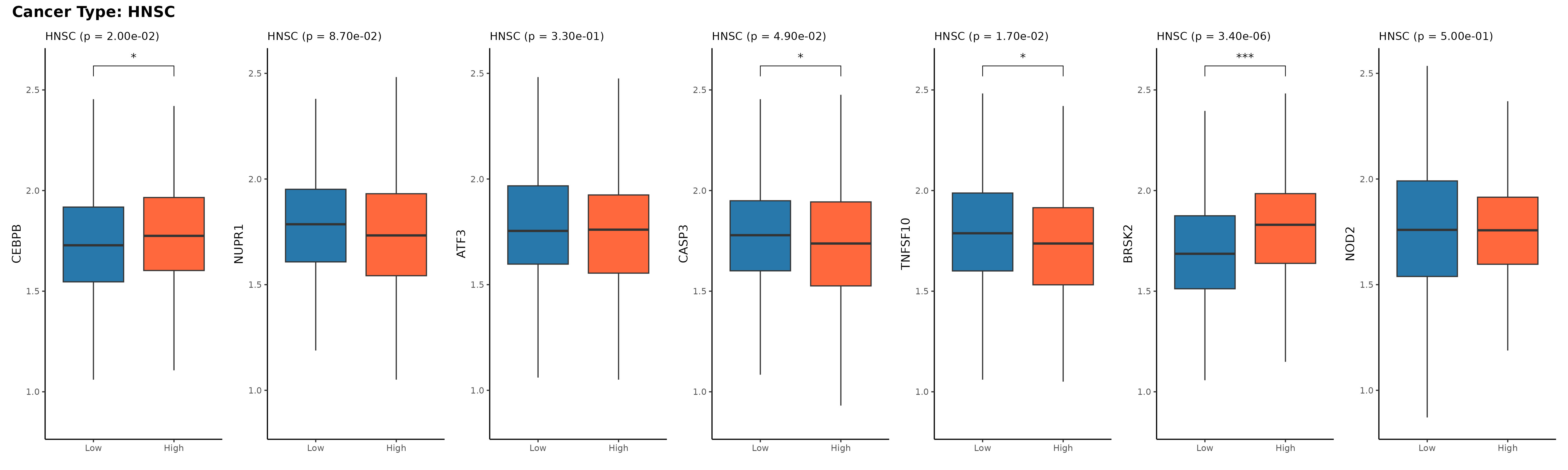

Supplement: Supplementary file 12 — Additional file12 (ZIP 3652 KB) [file 12672_2026_5126_MOESM12_ESM.zip › HNSC_combined.png]

Cancer Type: KICH

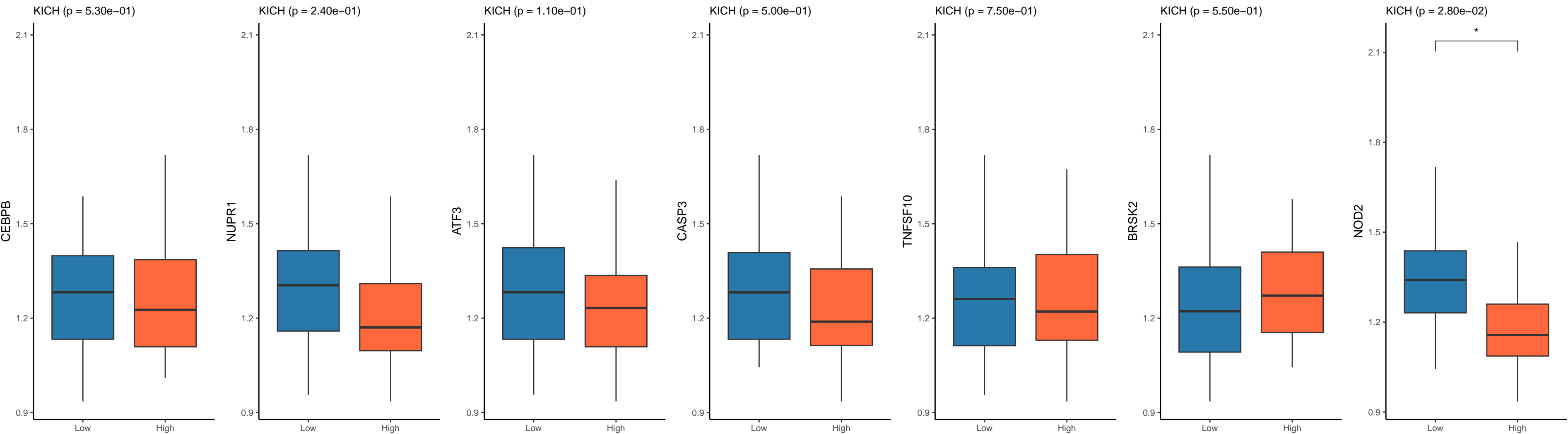

Supplement: Supplementary file 12 — Additional file12 (ZIP 3652 KB) [file 12672_2026_5126_MOESM12_ESM.zip › KICH_combined.pdf]

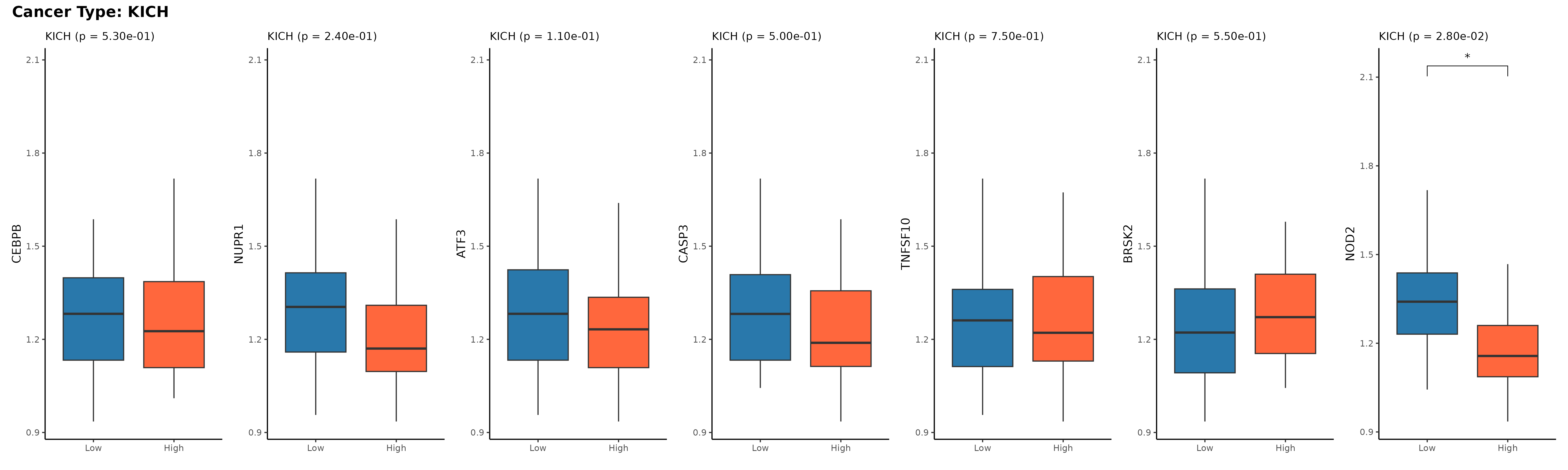

Supplement: Supplementary file 12 — Additional file12 (ZIP 3652 KB) [file 12672_2026_5126_MOESM12_ESM.zip › KICH_combined.png]

Cancer Type: KIRC

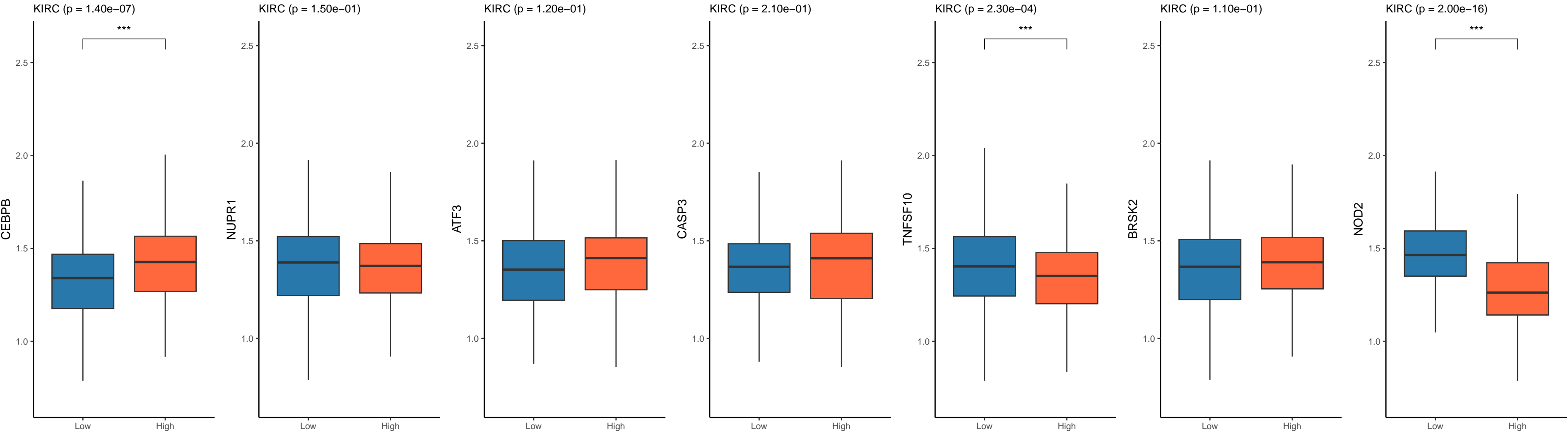

Supplement: Supplementary file 12 — Additional file12 (ZIP 3652 KB) [file 12672_2026_5126_MOESM12_ESM.zip › KIRC_combined.pdf]

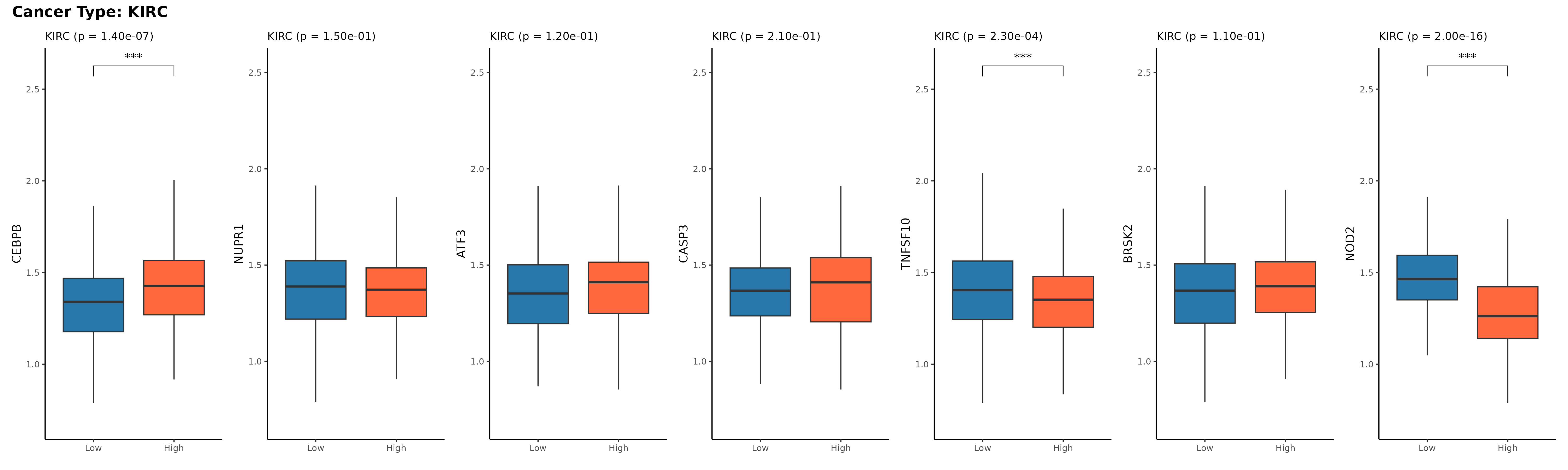

Supplement: Supplementary file 12 — Additional file12 (ZIP 3652 KB) [file 12672_2026_5126_MOESM12_ESM.zip › KIRC_combined.png]

Cancer Type: KIRP

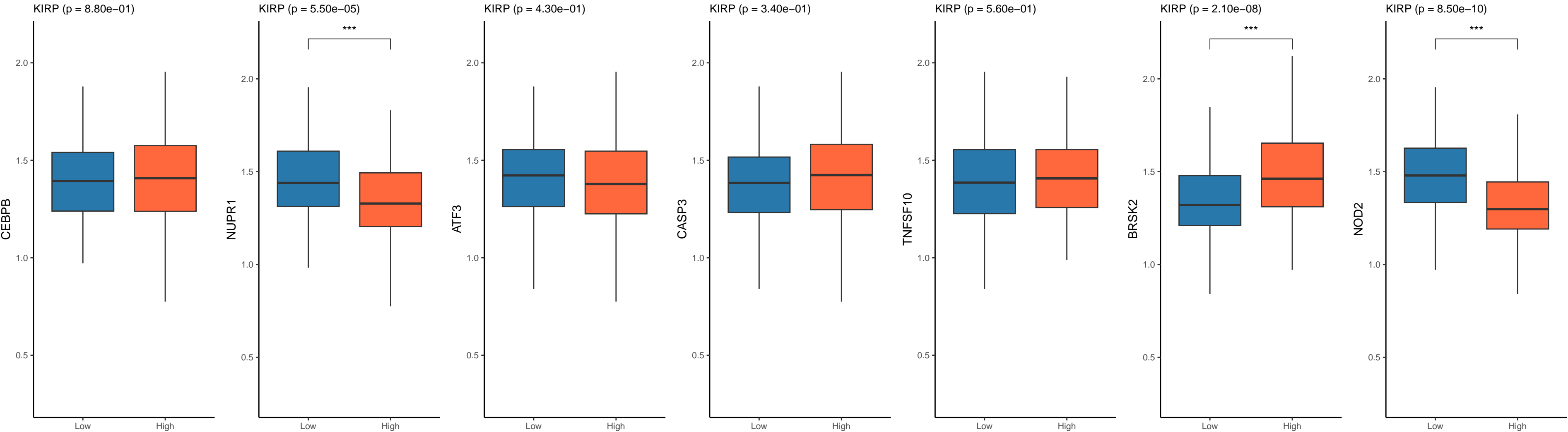

Supplement: Supplementary file 12 — Additional file12 (ZIP 3652 KB) [file 12672_2026_5126_MOESM12_ESM.zip › KIRP_combined.pdf]

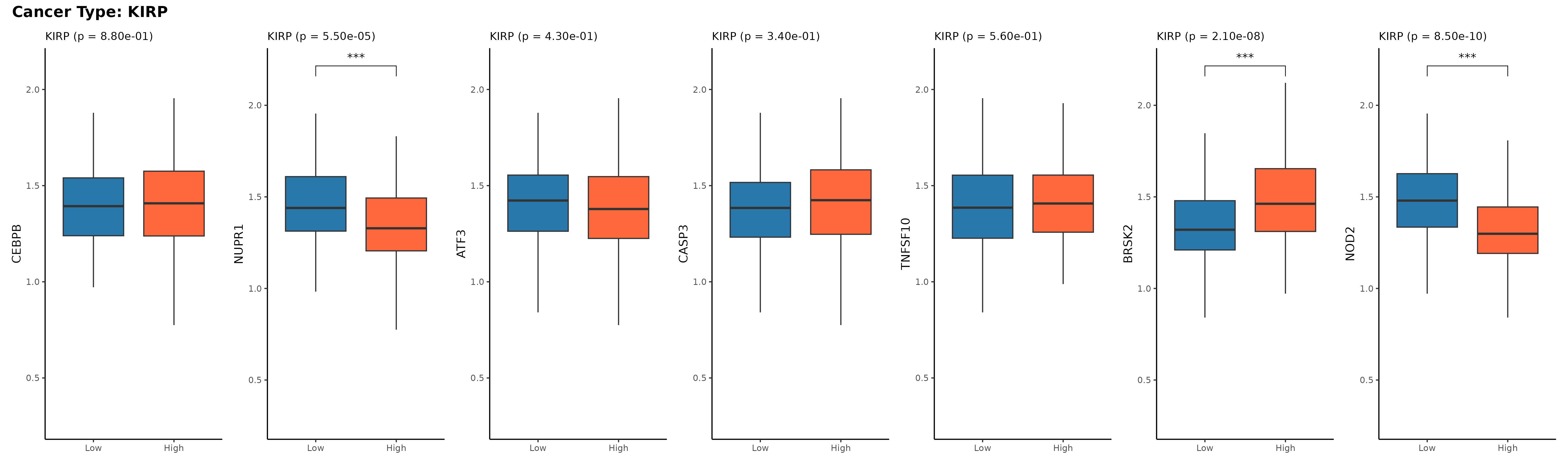

Supplement: Supplementary file 12 — Additional file12 (ZIP 3652 KB) [file 12672_2026_5126_MOESM12_ESM.zip › KIRP_combined.png]

Cancer Type: LAML

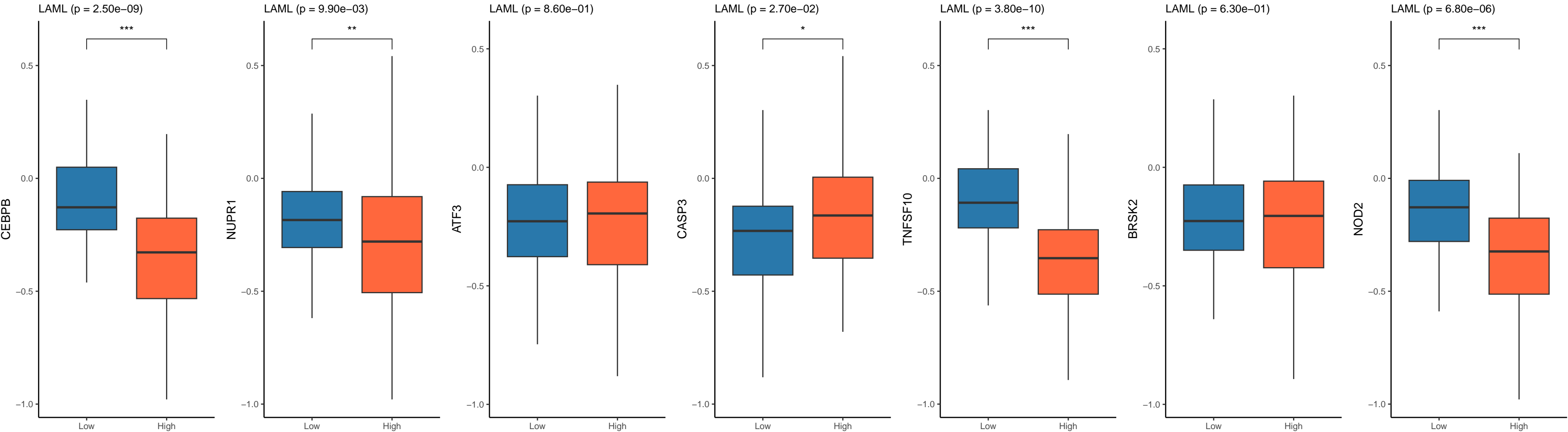

Supplement: Supplementary file 12 — Additional file12 (ZIP 3652 KB) [file 12672_2026_5126_MOESM12_ESM.zip › LAML_combined.pdf]

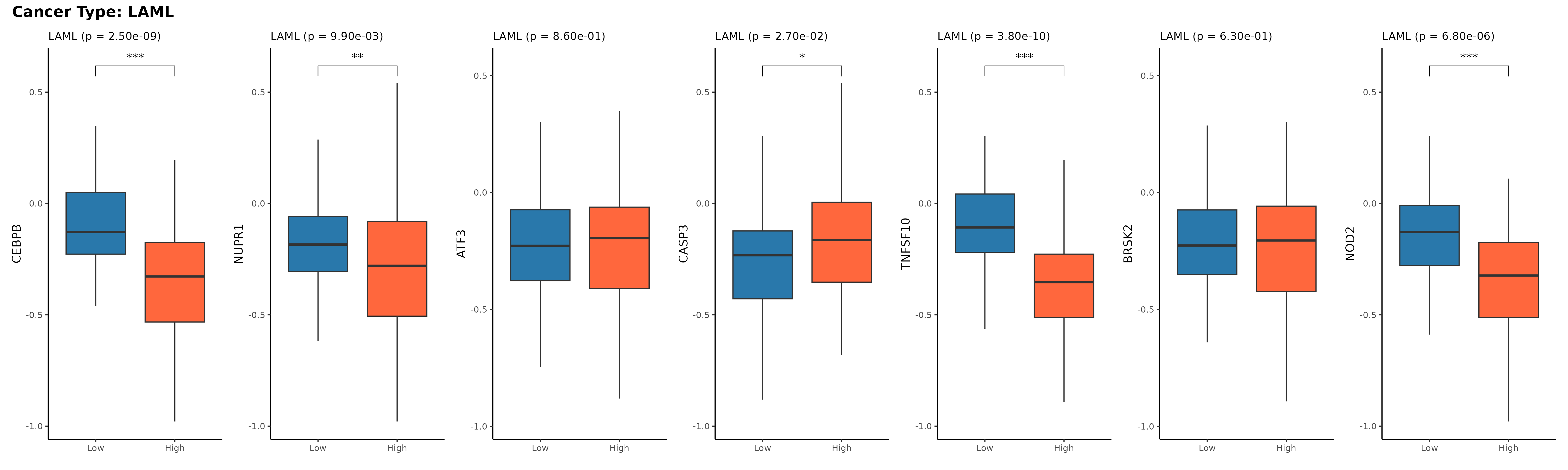

Supplement: Supplementary file 12 — Additional file12 (ZIP 3652 KB) [file 12672_2026_5126_MOESM12_ESM.zip › LAML_combined.png]

**Cancer Type: LGG**

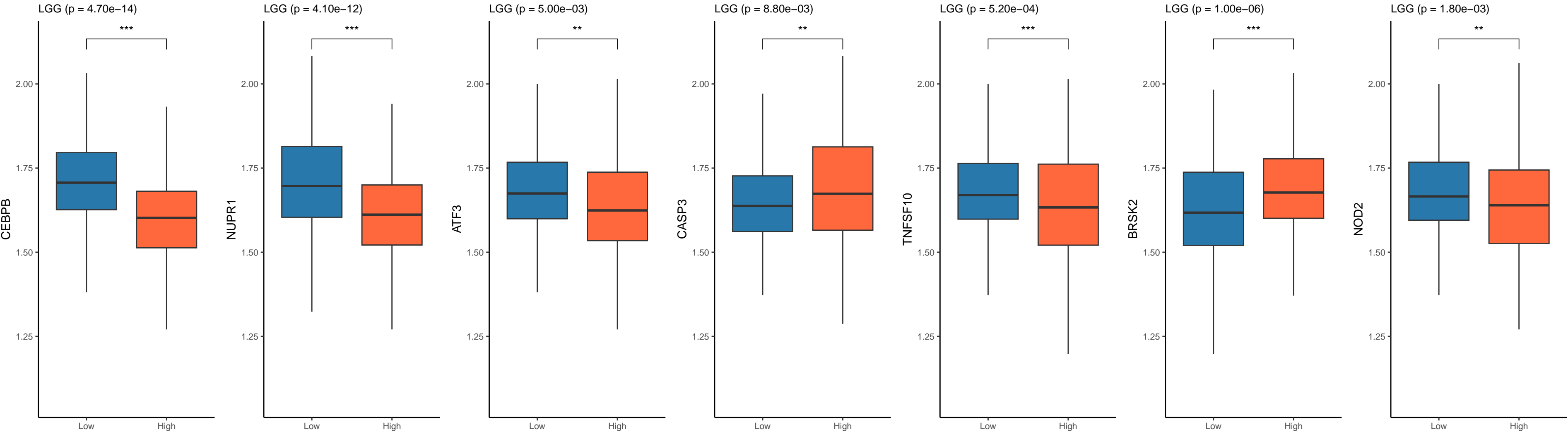

Supplement: Supplementary file 12 — Additional file12 (ZIP 3652 KB) [file 12672_2026_5126_MOESM12_ESM.zip › LGG_combined.pdf]

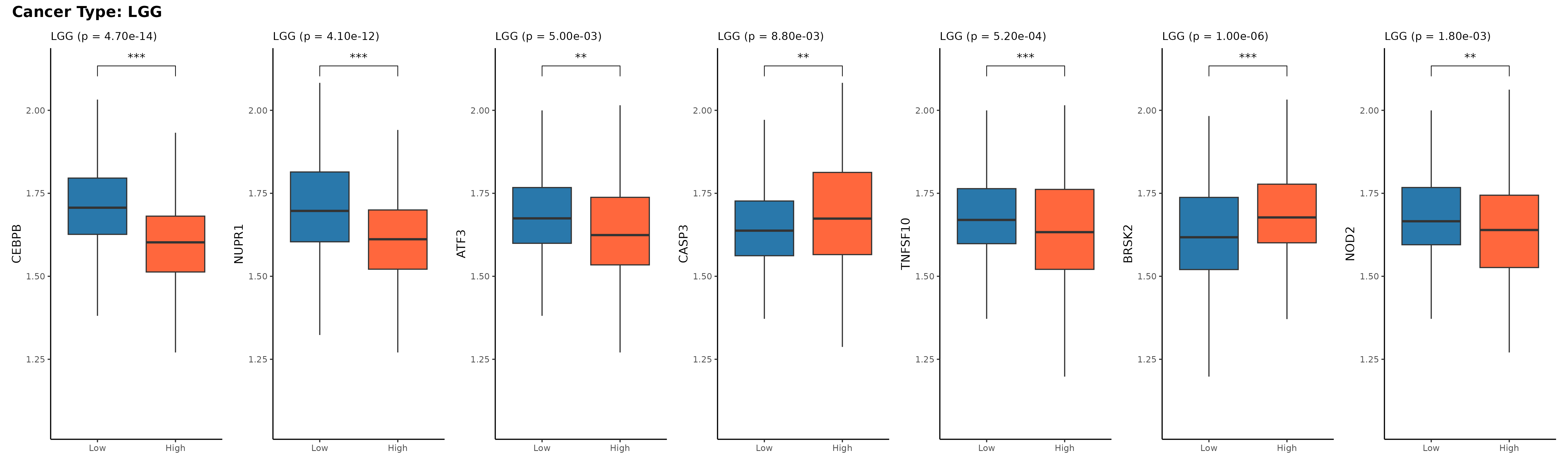

Supplement: Supplementary file 12 — Additional file12 (ZIP 3652 KB) [file 12672_2026_5126_MOESM12_ESM.zip › LGG_combined.png]

Cancer Type: LIHC

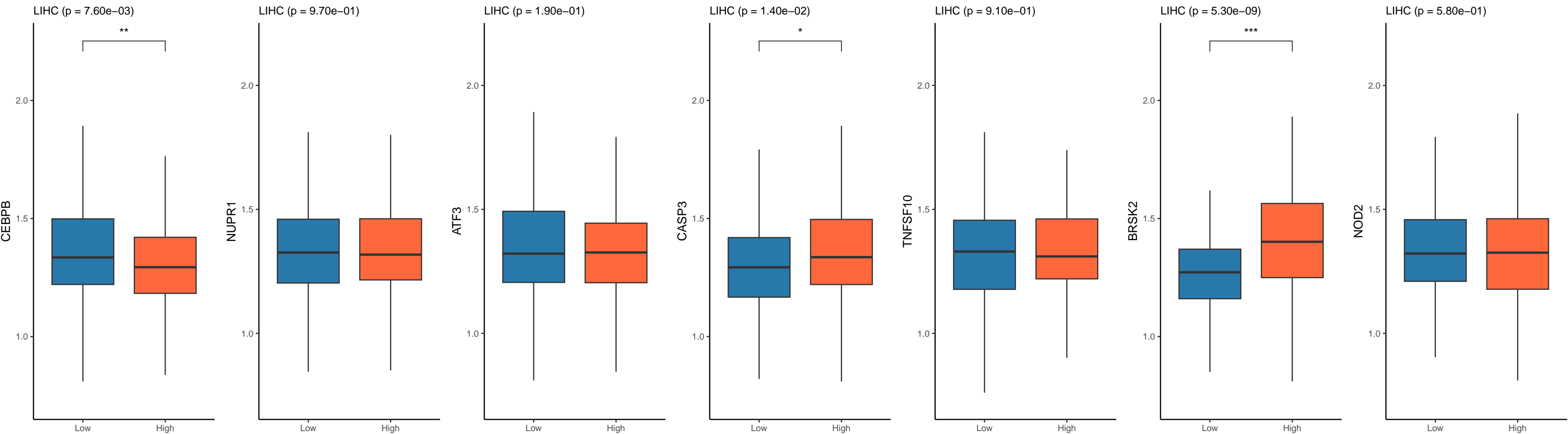

Supplement: Supplementary file 12 — Additional file12 (ZIP 3652 KB) [file 12672_2026_5126_MOESM12_ESM.zip › LIHC_combined.pdf]

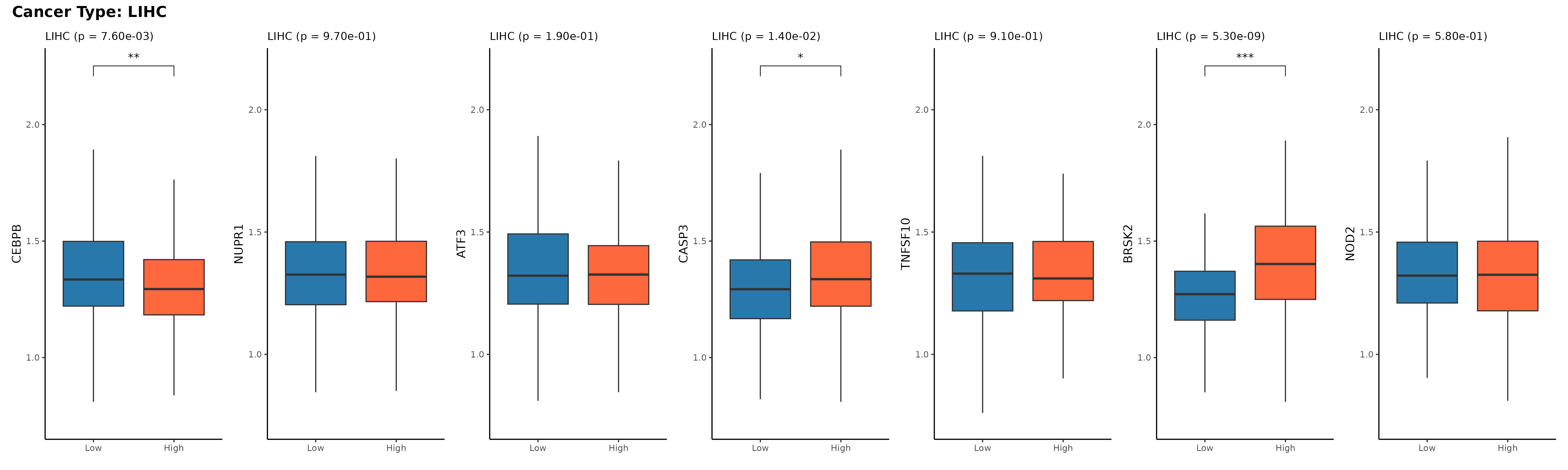

Supplement: Supplementary file 12 — Additional file12 (ZIP 3652 KB) [file 12672_2026_5126_MOESM12_ESM.zip › LIHC_combined.png]

Cancer Type: LUAD

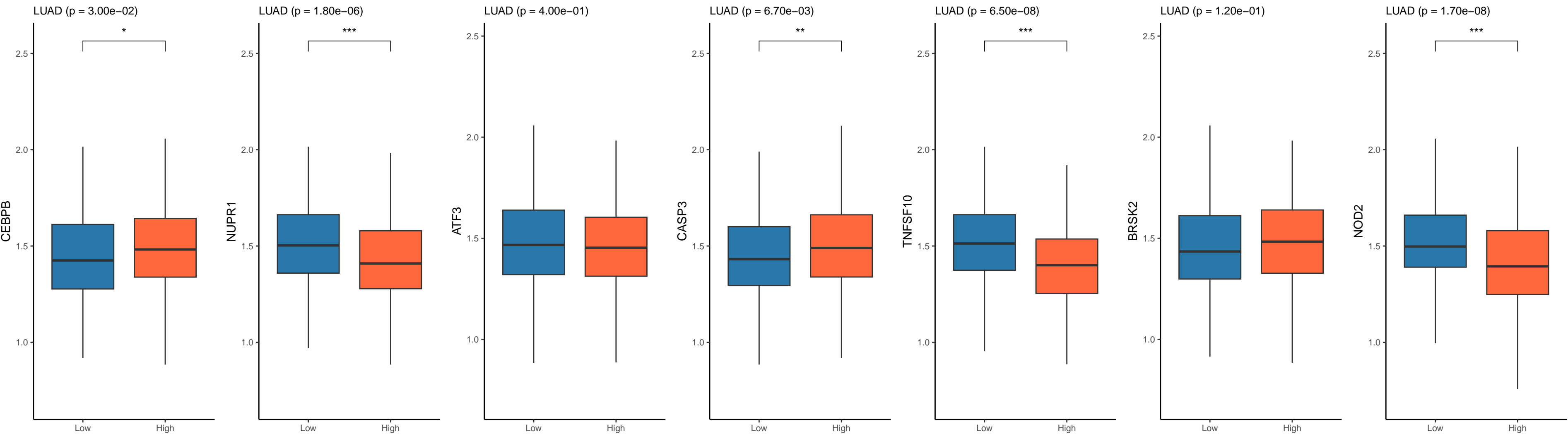

Supplement: Supplementary file 12 — Additional file12 (ZIP 3652 KB) [file 12672_2026_5126_MOESM12_ESM.zip › LUAD_combined.pdf]

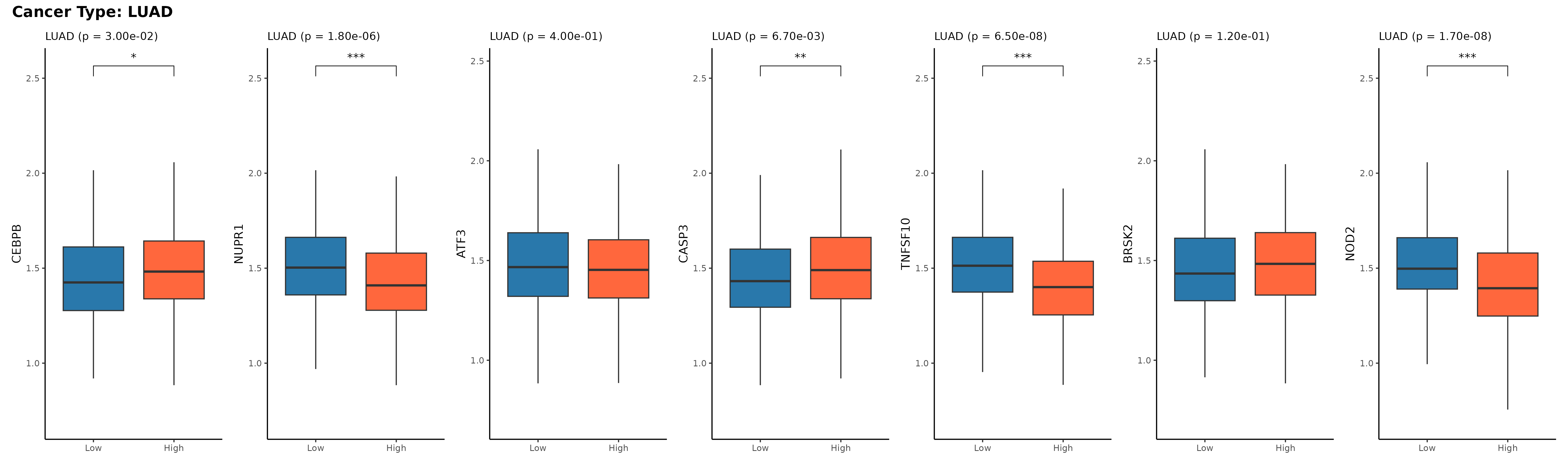

Supplement: Supplementary file 12 — Additional file12 (ZIP 3652 KB) [file 12672_2026_5126_MOESM12_ESM.zip › LUAD_combined.png]

Cancer Type: LUSC

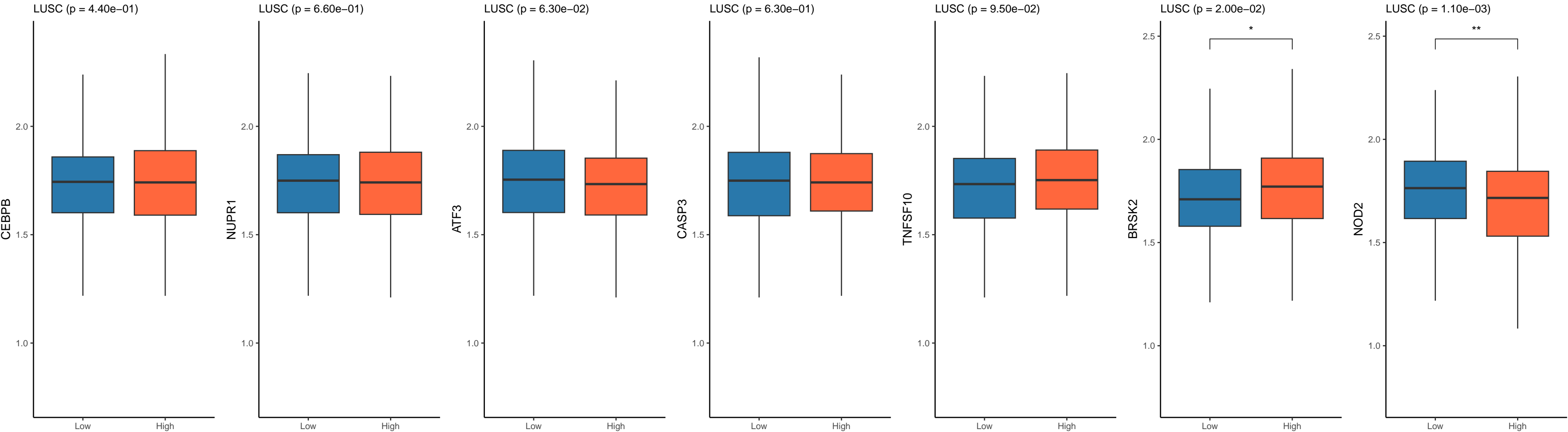

Supplement: Supplementary file 12 — Additional file12 (ZIP 3652 KB) [file 12672_2026_5126_MOESM12_ESM.zip › LUSC_combined.pdf]

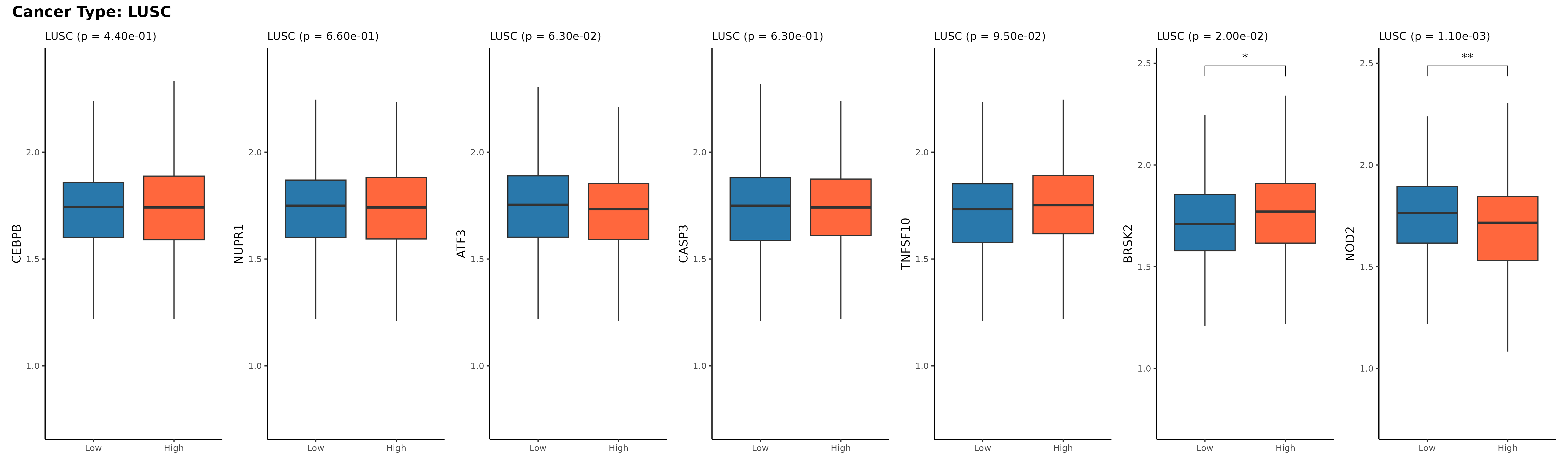

Supplement: Supplementary file 12 — Additional file12 (ZIP 3652 KB) [file 12672_2026_5126_MOESM12_ESM.zip › LUSC_combined.png]

Cancer Type: MESO

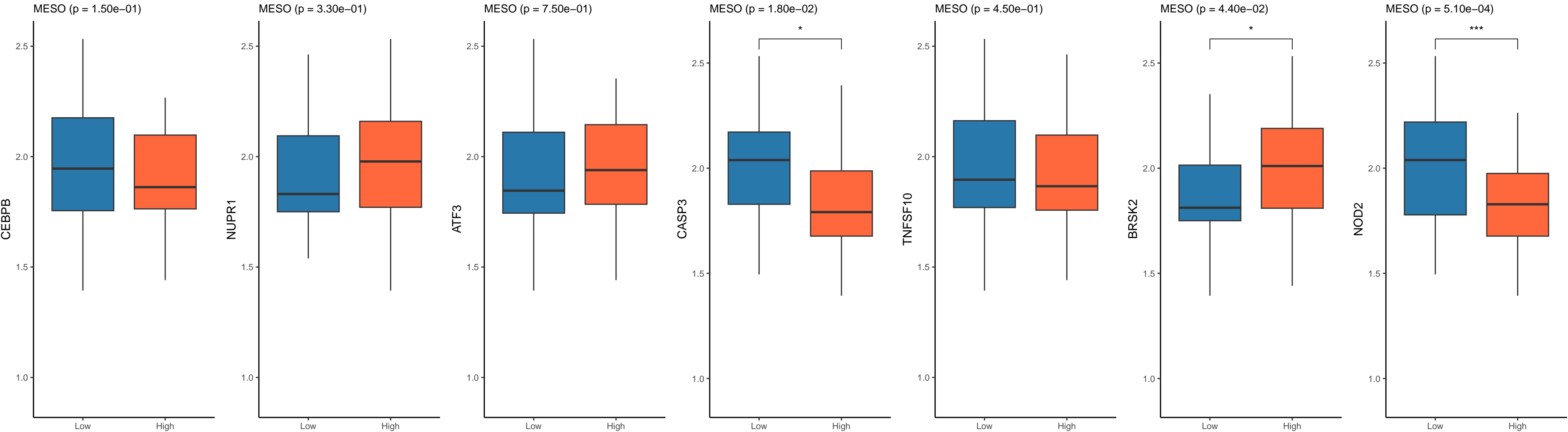

Supplement: Supplementary file 12 — Additional file12 (ZIP 3652 KB) [file 12672_2026_5126_MOESM12_ESM.zip › MESO_combined.pdf]

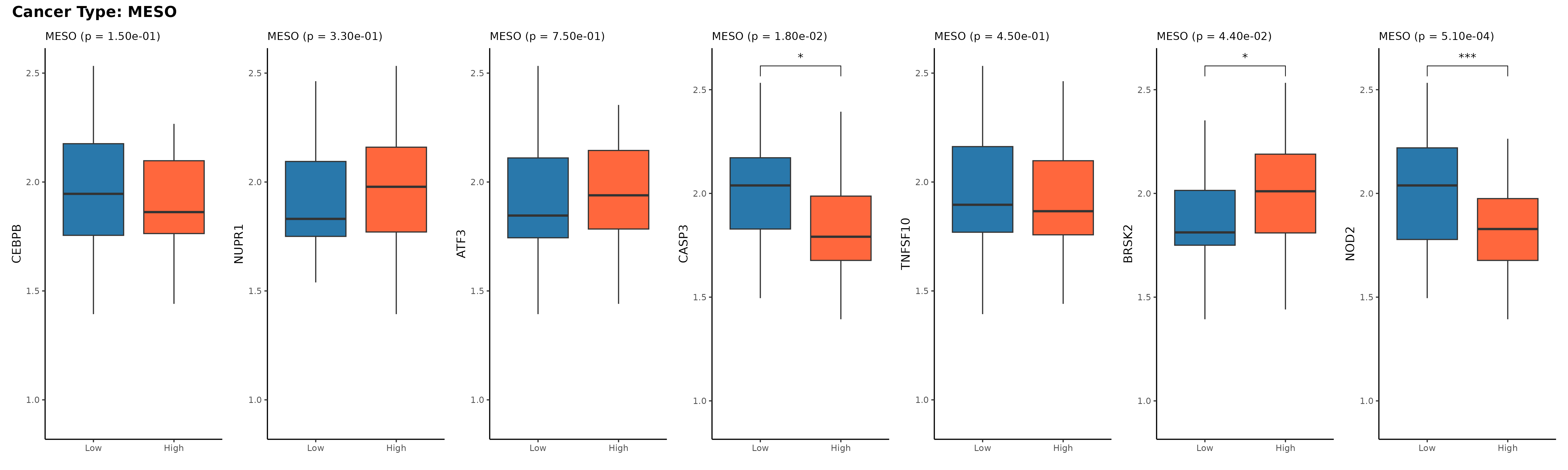

Supplement: Supplementary file 12 — Additional file12 (ZIP 3652 KB) [file 12672_2026_5126_MOESM12_ESM.zip › MESO_combined.png]

Cancer Type: OV

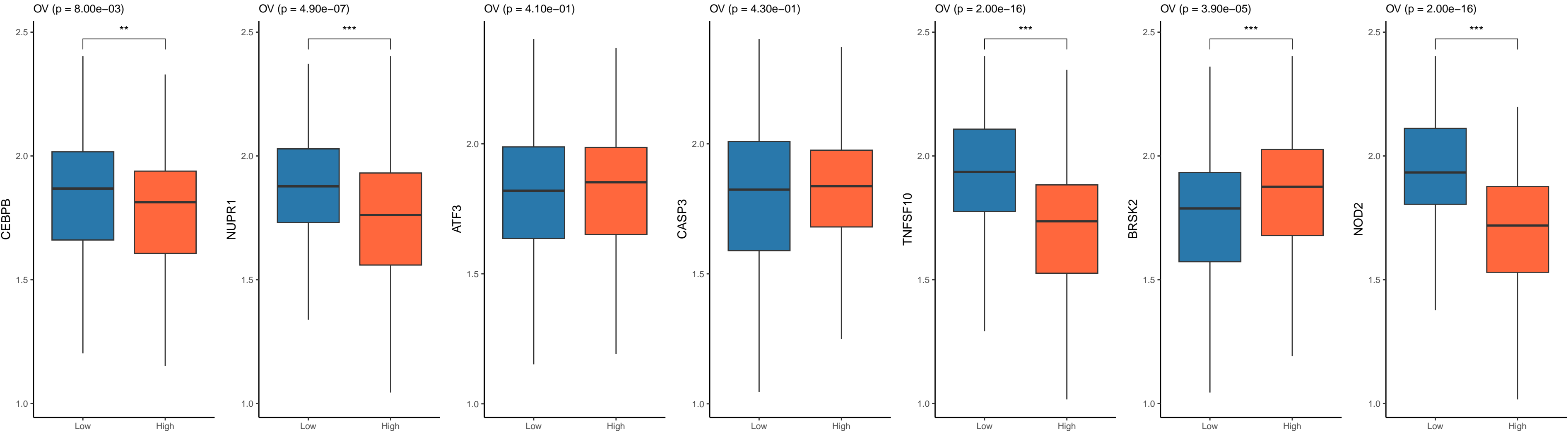

Supplement: Supplementary file 12 — Additional file12 (ZIP 3652 KB) [file 12672_2026_5126_MOESM12_ESM.zip › OV_combined.pdf]

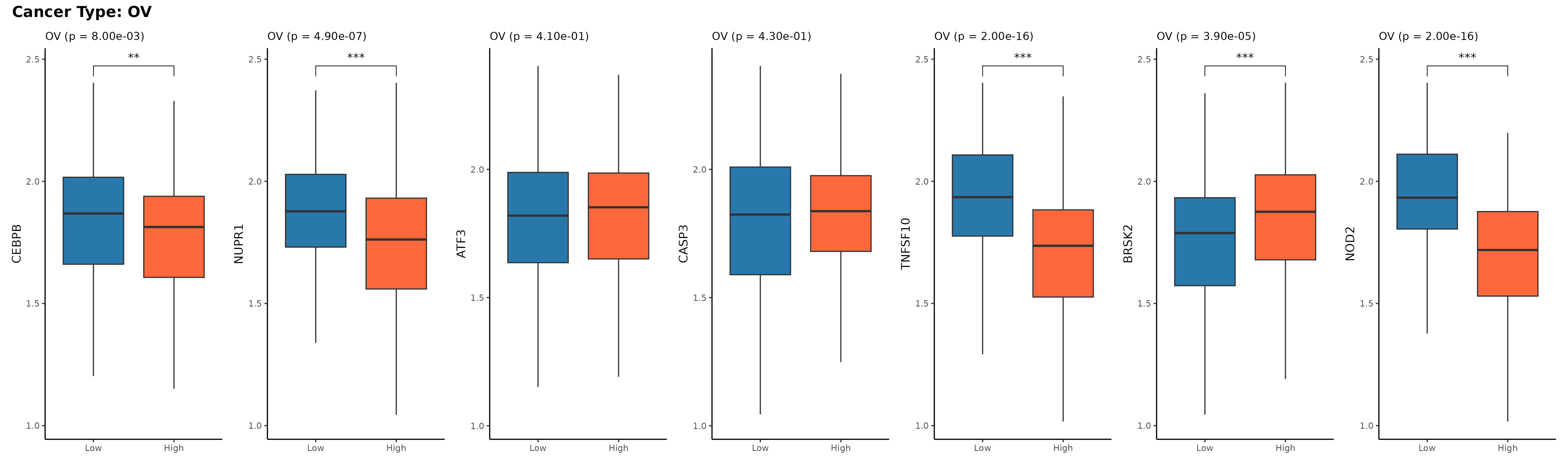

Supplement: Supplementary file 12 — Additional file12 (ZIP 3652 KB) [file 12672_2026_5126_MOESM12_ESM.zip › OV_combined.png]

Cancer Type: PAAD

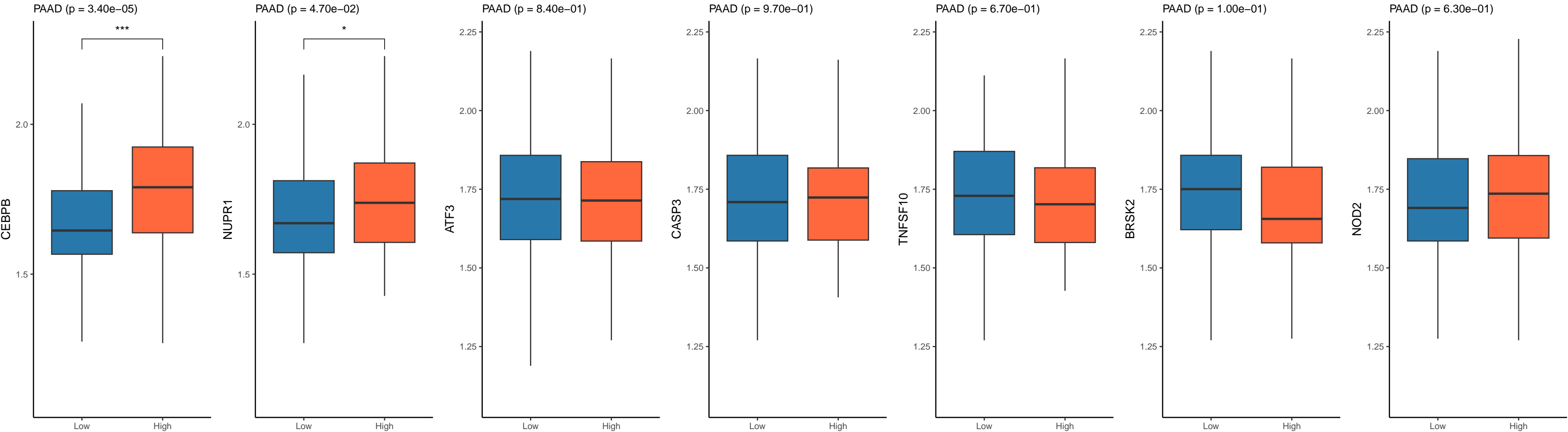

Supplement: Supplementary file 12 — Additional file12 (ZIP 3652 KB) [file 12672_2026_5126_MOESM12_ESM.zip › PAAD_combined.pdf]

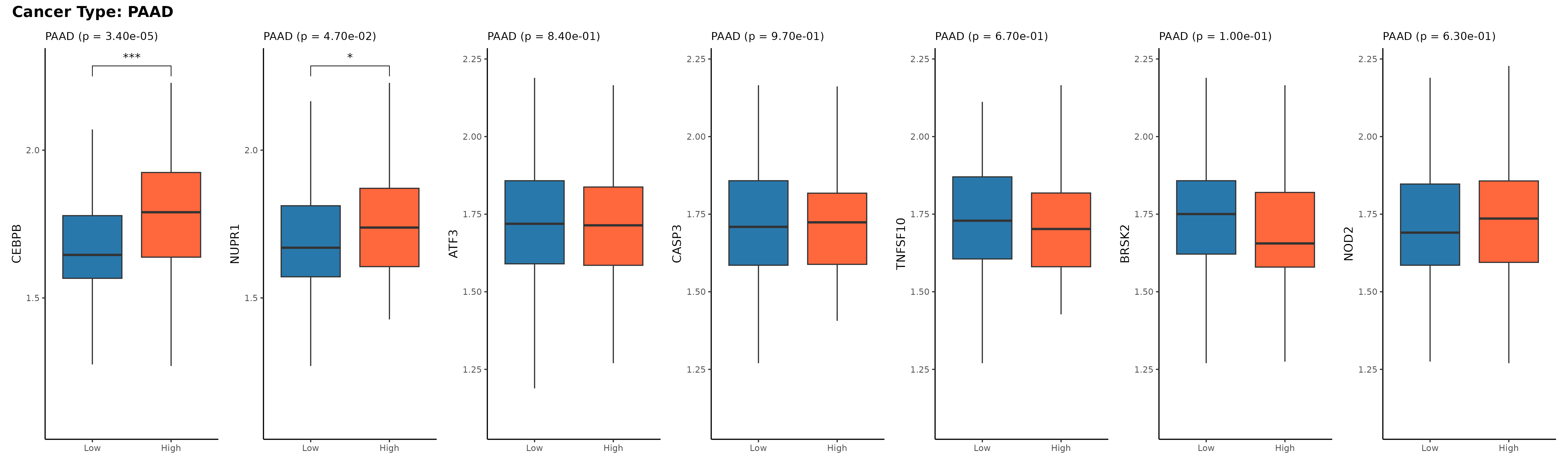

Supplement: Supplementary file 12 — Additional file12 (ZIP 3652 KB) [file 12672_2026_5126_MOESM12_ESM.zip › PAAD_combined.png]

Cancer Type: PCPG

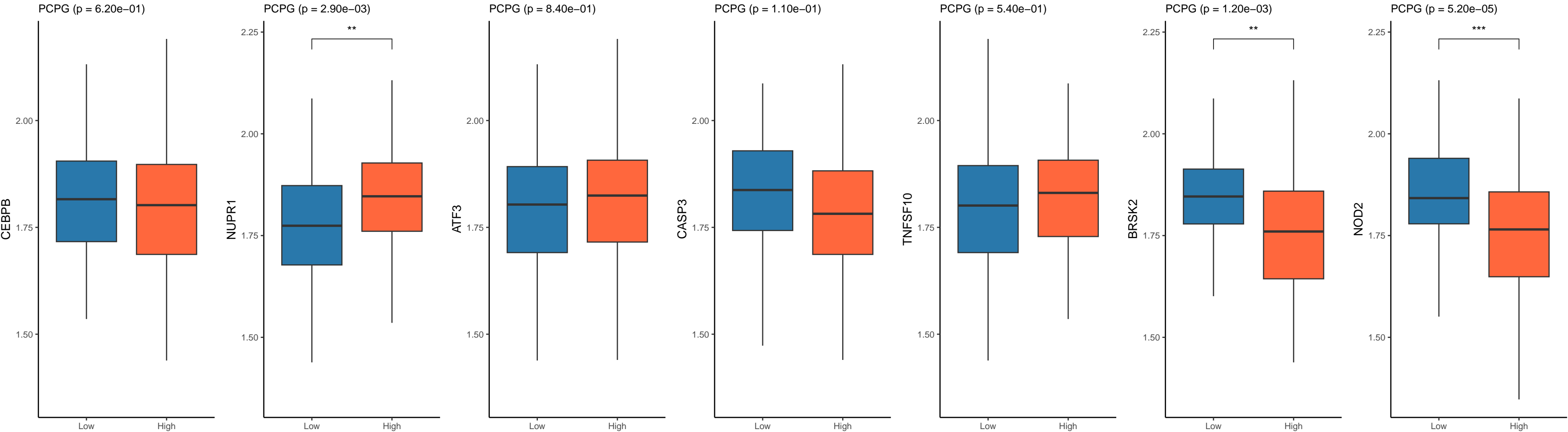

Supplement: Supplementary file 12 — Additional file12 (ZIP 3652 KB) [file 12672_2026_5126_MOESM12_ESM.zip › PCPG_combined.pdf]

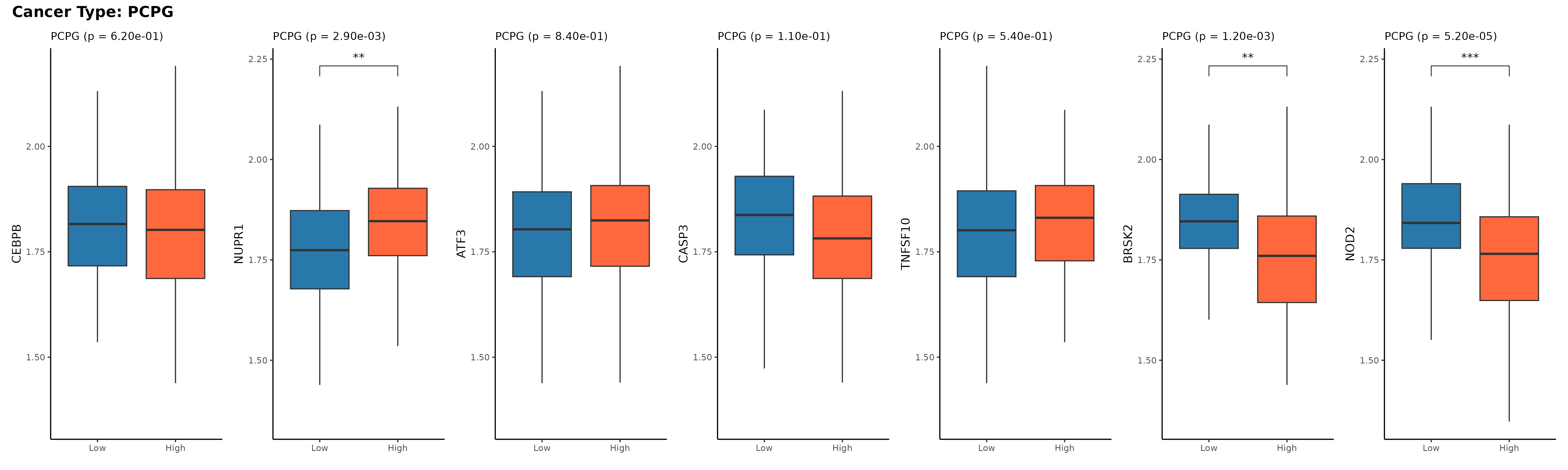

Supplement: Supplementary file 12 — Additional file12 (ZIP 3652 KB) [file 12672_2026_5126_MOESM12_ESM.zip › PCPG_combined.png]

Cancer Type: PRAD

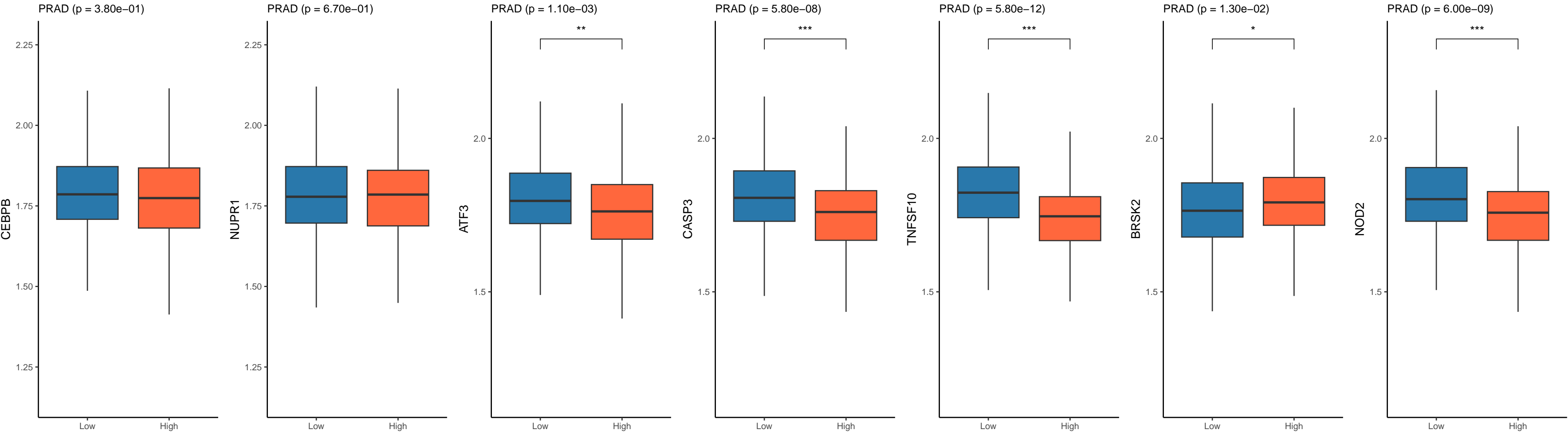

Supplement: Supplementary file 12 — Additional file12 (ZIP 3652 KB) [file 12672_2026_5126_MOESM12_ESM.zip › PRAD_combined.pdf]

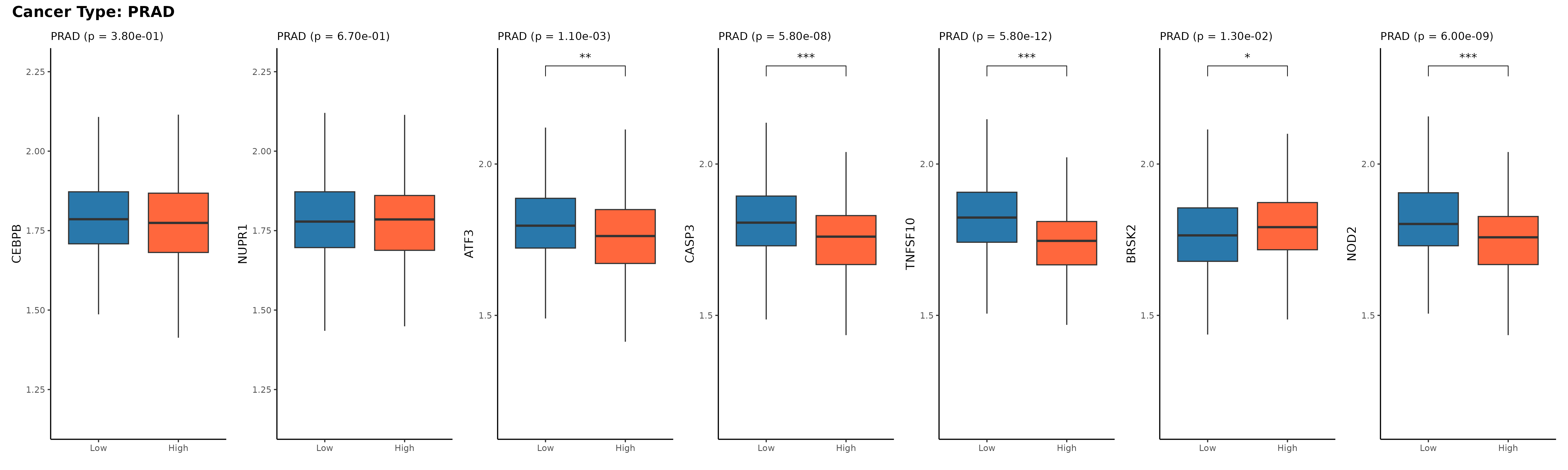

Supplement: Supplementary file 12 — Additional file12 (ZIP 3652 KB) [file 12672_2026_5126_MOESM12_ESM.zip › PRAD_combined.png]

Cancer Type: READ

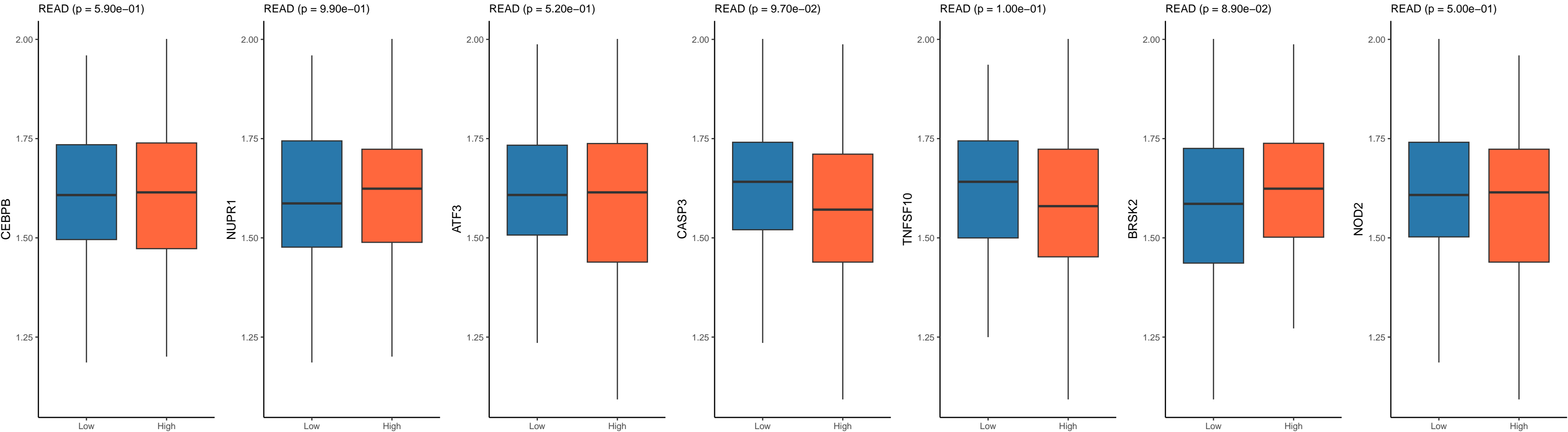

Supplement: Supplementary file 12 — Additional file12 (ZIP 3652 KB) [file 12672_2026_5126_MOESM12_ESM.zip › READ_combined.pdf]

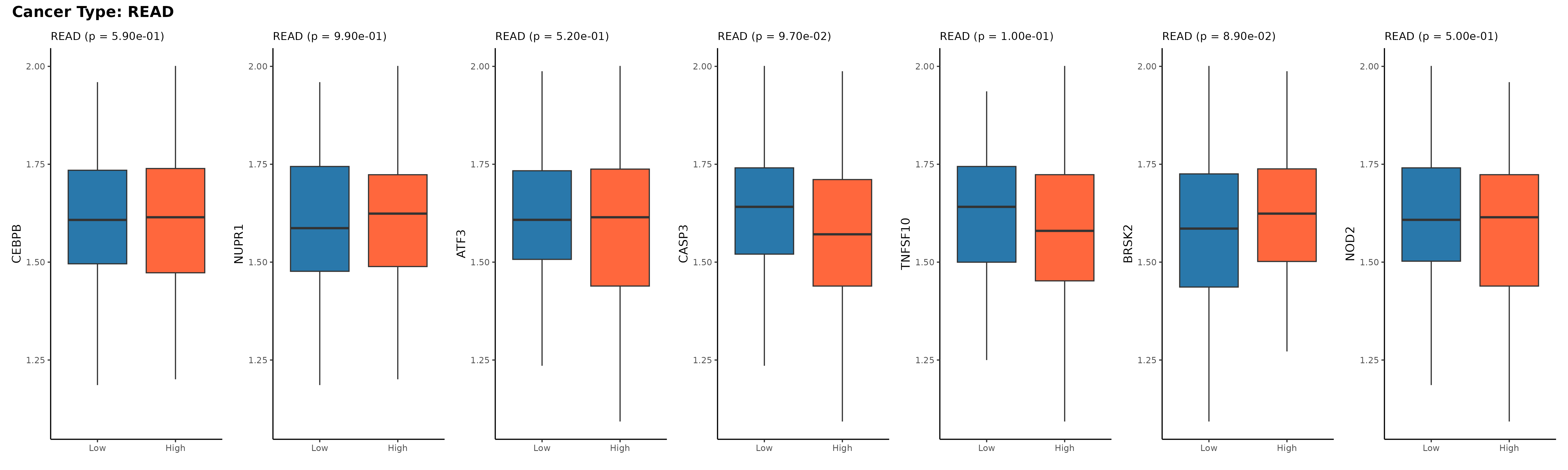

Supplement: Supplementary file 12 — Additional file12 (ZIP 3652 KB) [file 12672_2026_5126_MOESM12_ESM.zip › READ_combined.png]

Cancer Type: SARC

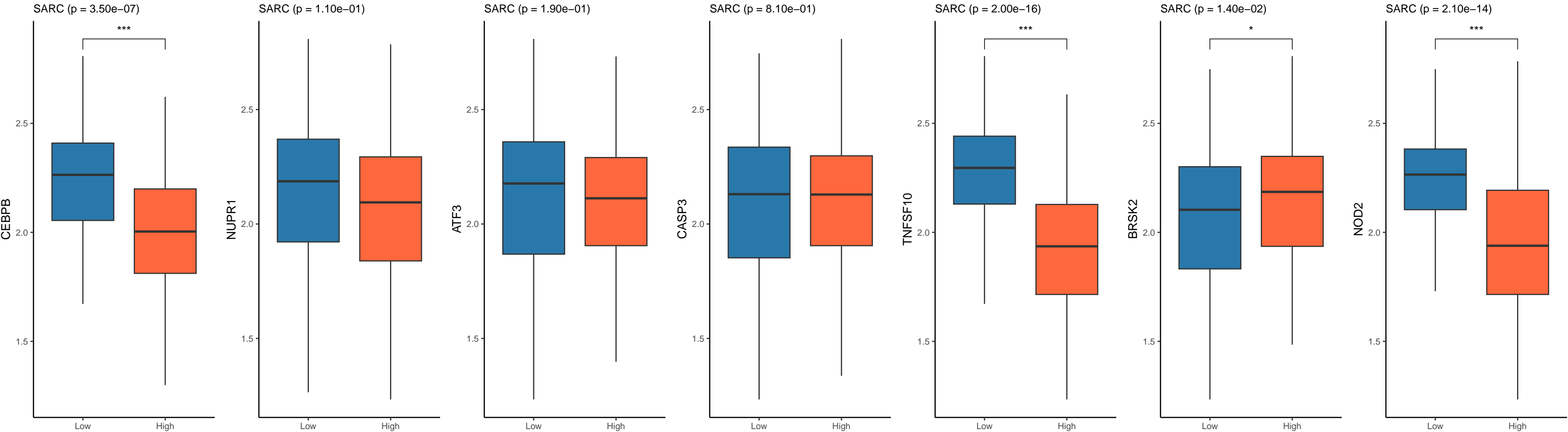

Supplement: Supplementary file 12 — Additional file12 (ZIP 3652 KB) [file 12672_2026_5126_MOESM12_ESM.zip › SARC_combined.pdf]

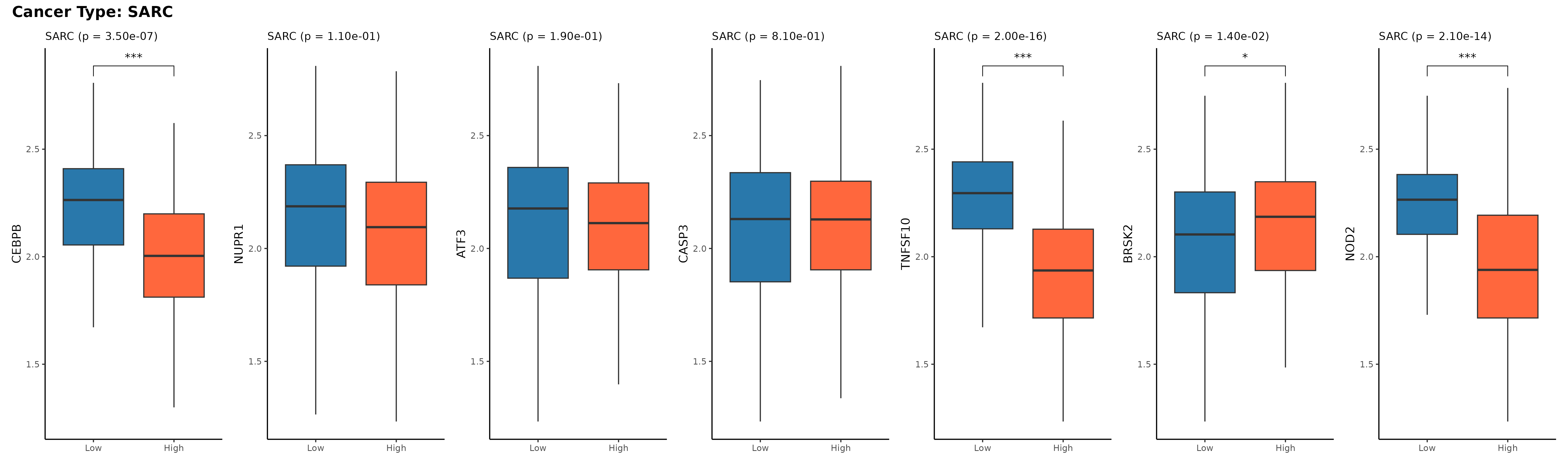

Supplement: Supplementary file 12 — Additional file12 (ZIP 3652 KB) [file 12672_2026_5126_MOESM12_ESM.zip › SARC_combined.png]

# Significant Results (Page 1/11)

Plots: 1–12 (Total: 121)

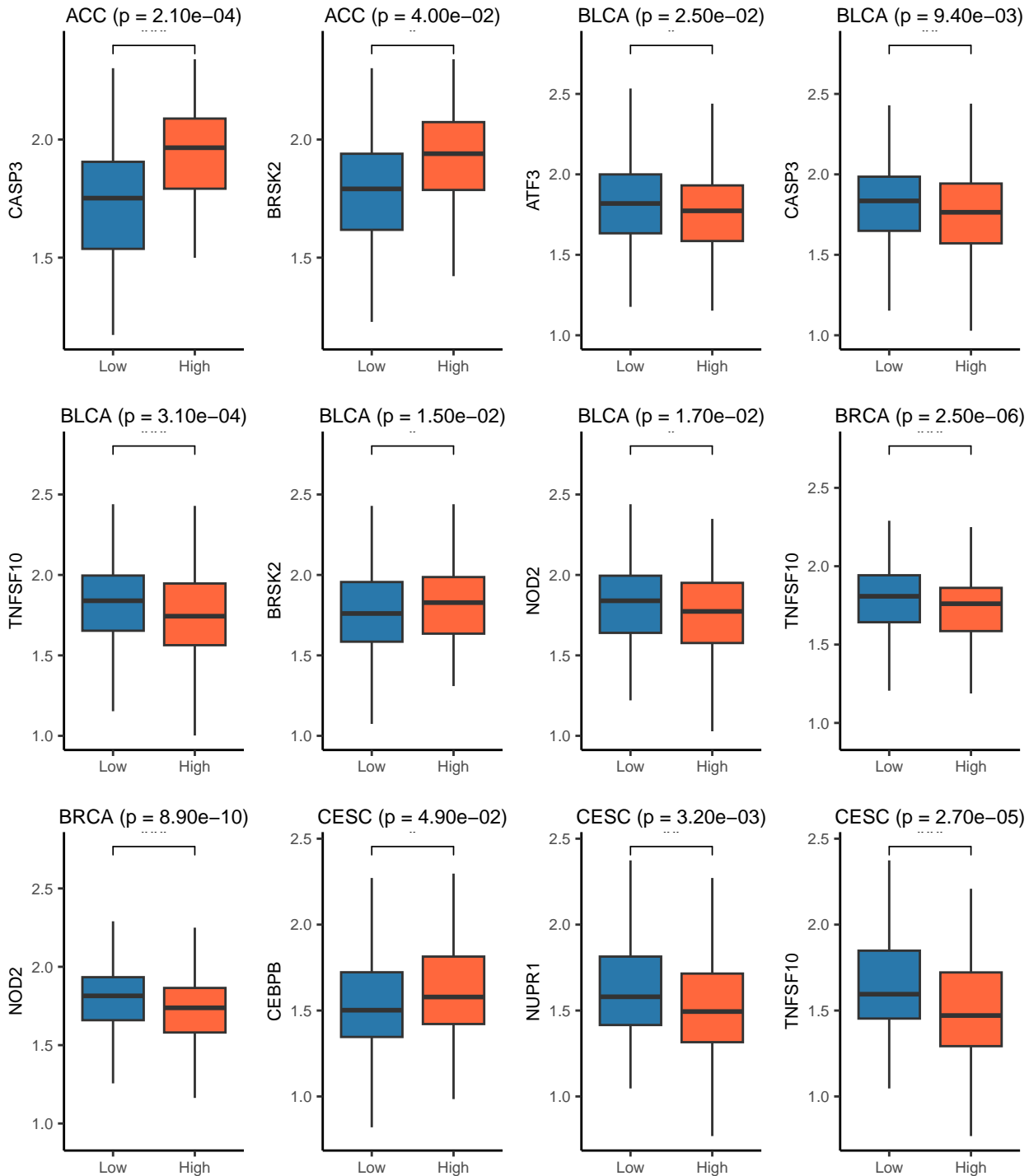

Supplement: Supplementary file 12 — Additional file12 (ZIP 3652 KB) [file 12672_2026_5126_MOESM12_ESM.zip › significant_results_page1.pdf]

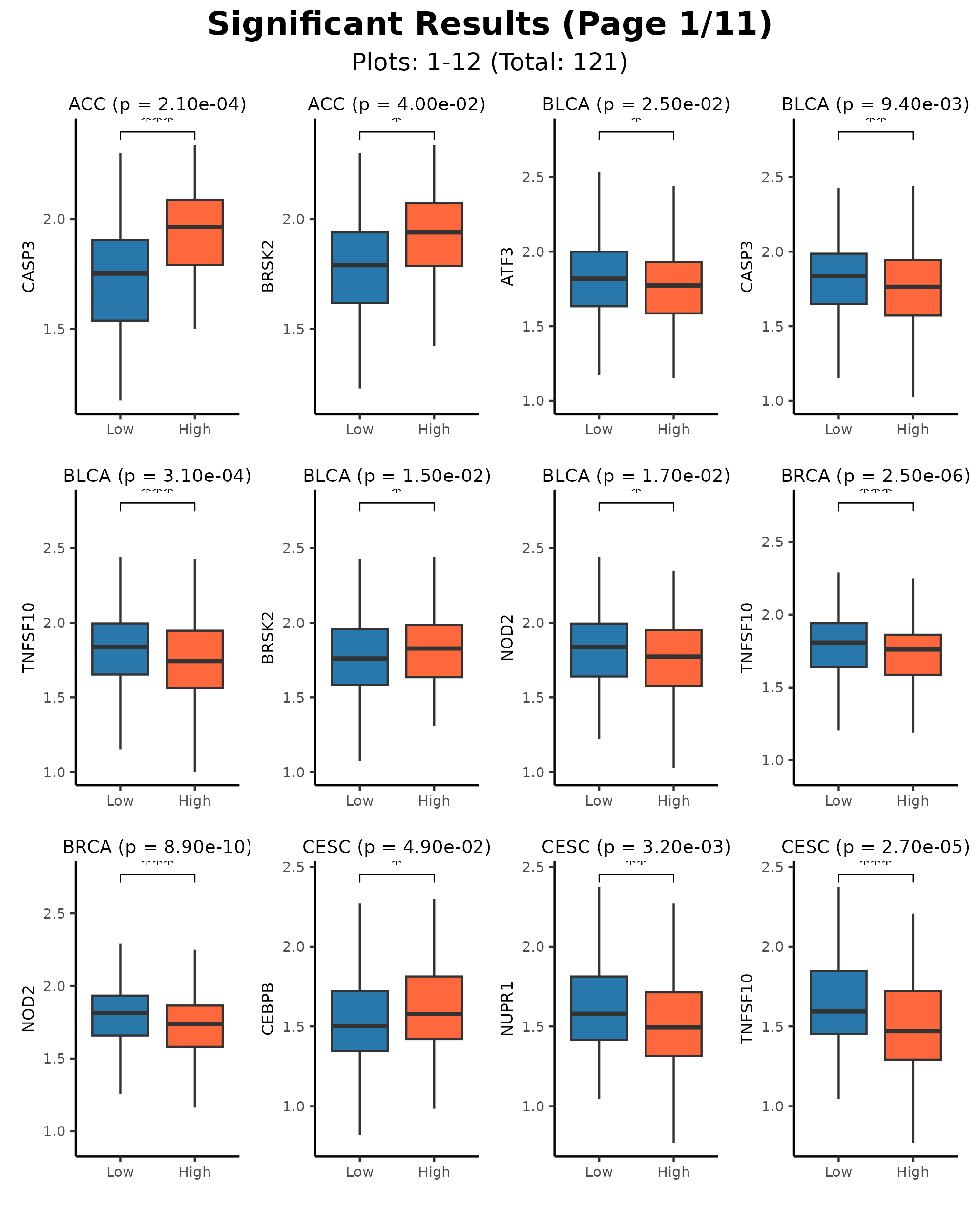

Supplement: Supplementary file 12 — Additional file12 (ZIP 3652 KB) [file 12672_2026_5126_MOESM12_ESM.zip › significant_results_page1.png]

# Significant Results (Page 10/11)

Plots: 109–120 (Total: 121)

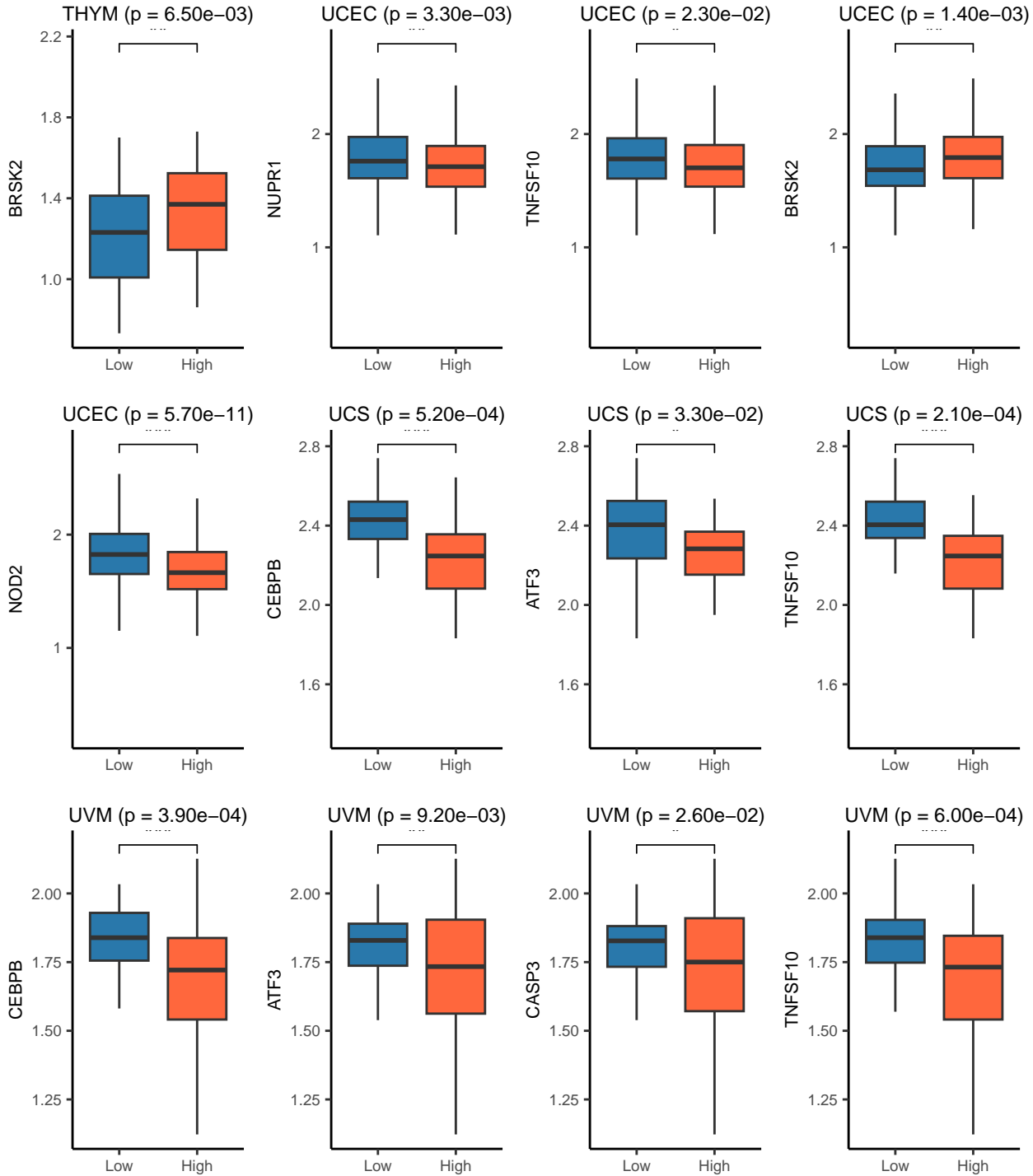

Supplement: Supplementary file 12 — Additional file12 (ZIP 3652 KB) [file 12672_2026_5126_MOESM12_ESM.zip › significant_results_page10.pdf]

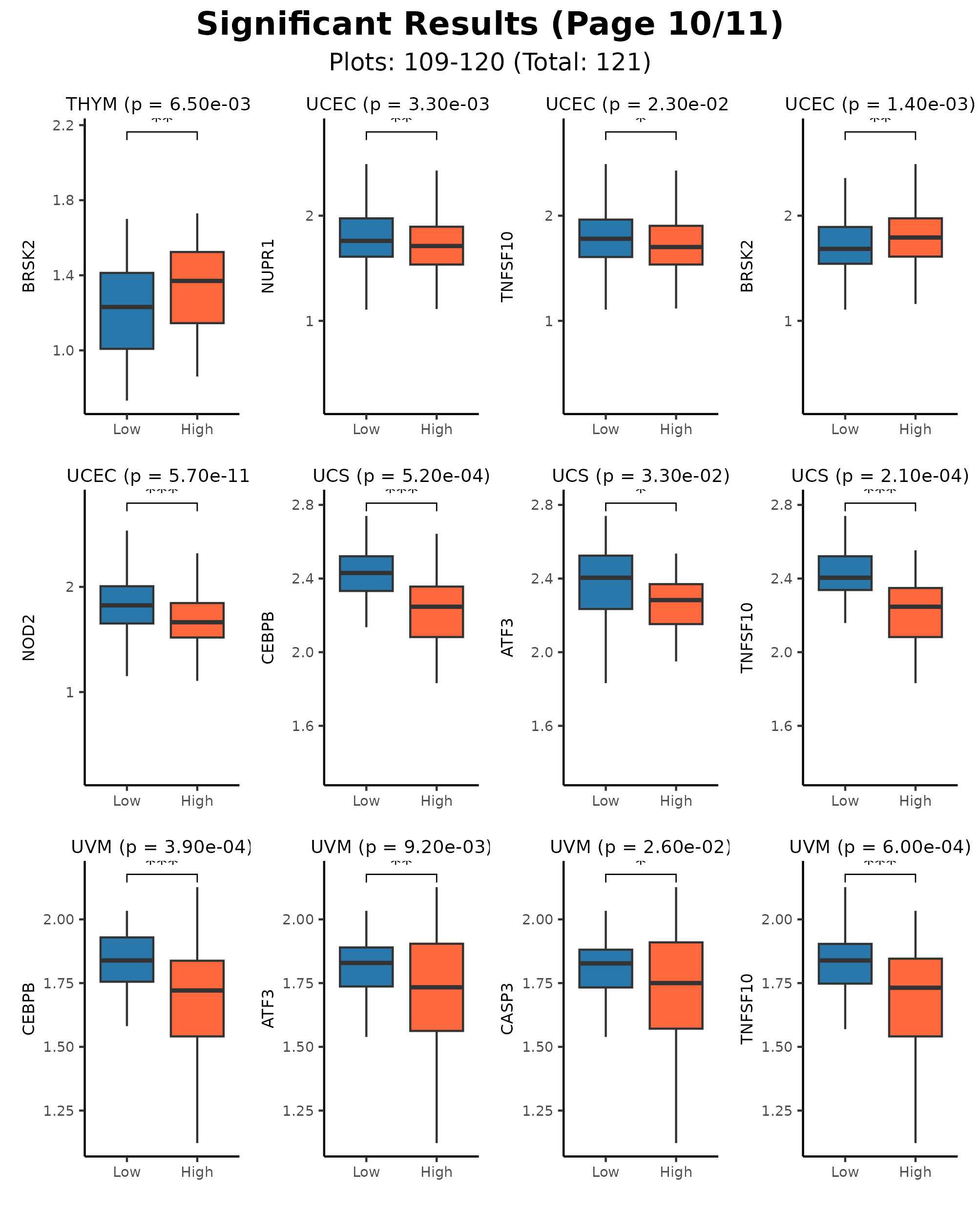

Supplement: Supplementary file 12 — Additional file12 (ZIP 3652 KB) [file 12672_2026_5126_MOESM12_ESM.zip › significant_results_page10.png]

# Significant Results (Page 11/11)

Plots: 121–121 (Total: 121)

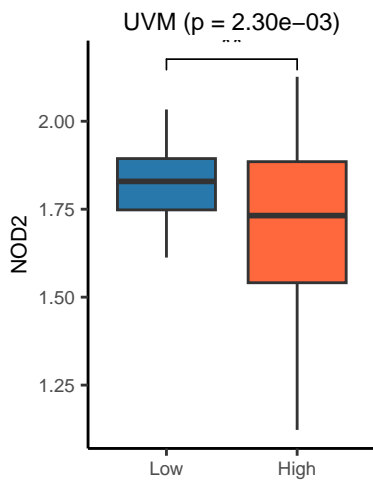

Supplement: Supplementary file 12 — Additional file12 (ZIP 3652 KB) [file 12672_2026_5126_MOESM12_ESM.zip › significant_results_page11.pdf]

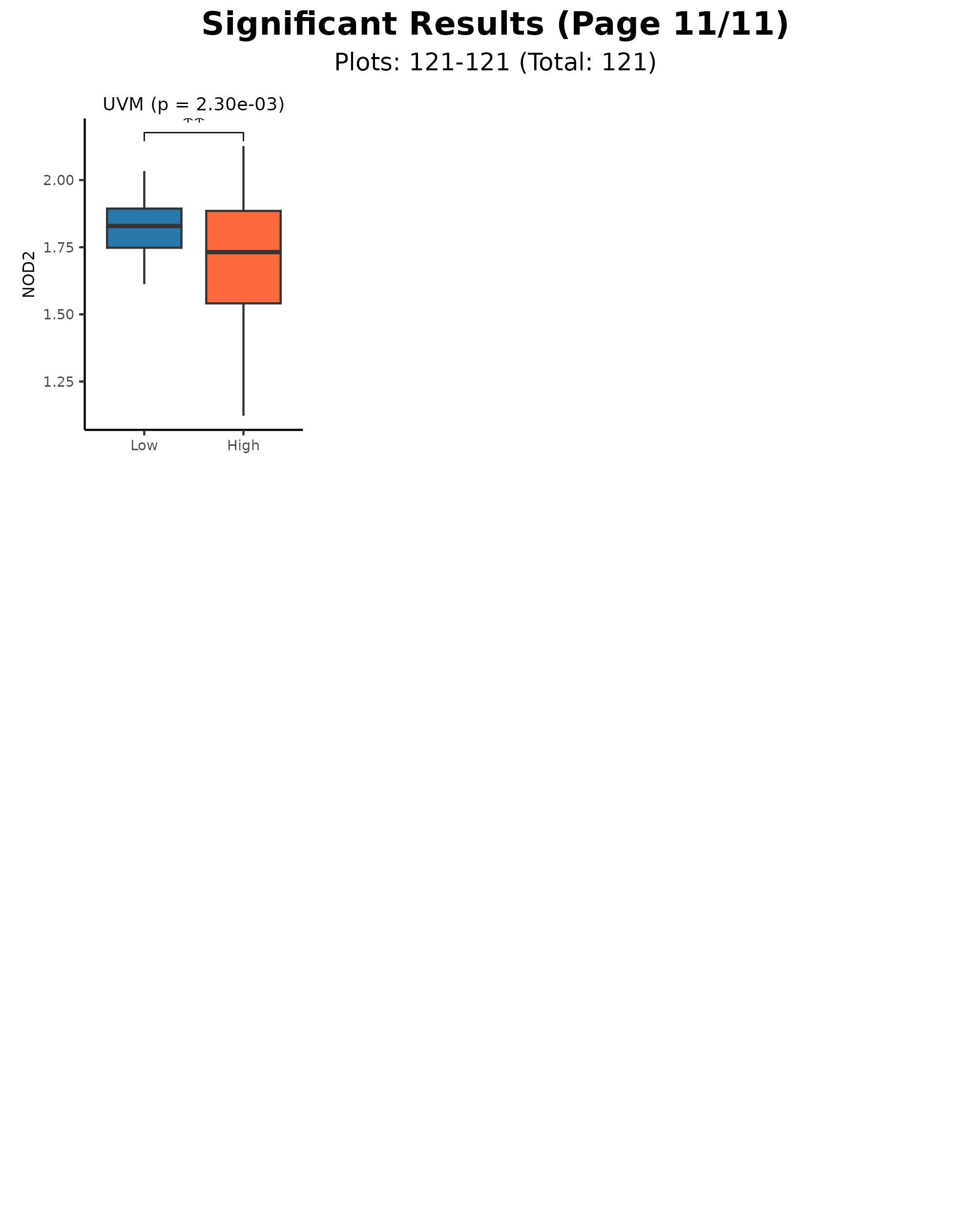

Supplement: Supplementary file 12 — Additional file12 (ZIP 3652 KB) [file 12672_2026_5126_MOESM12_ESM.zip › significant_results_page11.png]

# Significant Results (Page 2/11)

Plots: 13–24 (Total: 121)

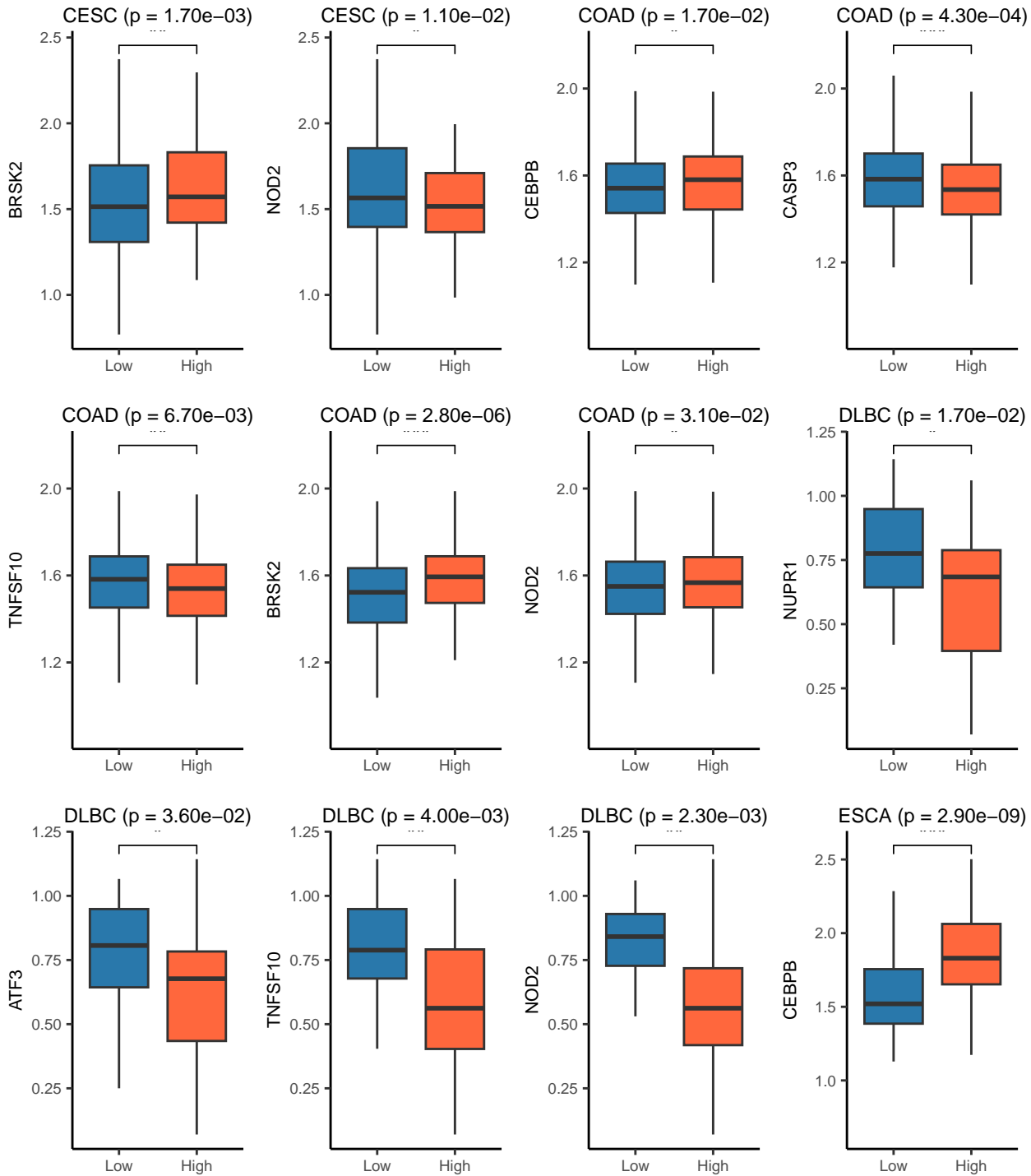

Supplement: Supplementary file 12 — Additional file12 (ZIP 3652 KB) [file 12672_2026_5126_MOESM12_ESM.zip › significant_results_page2.pdf]

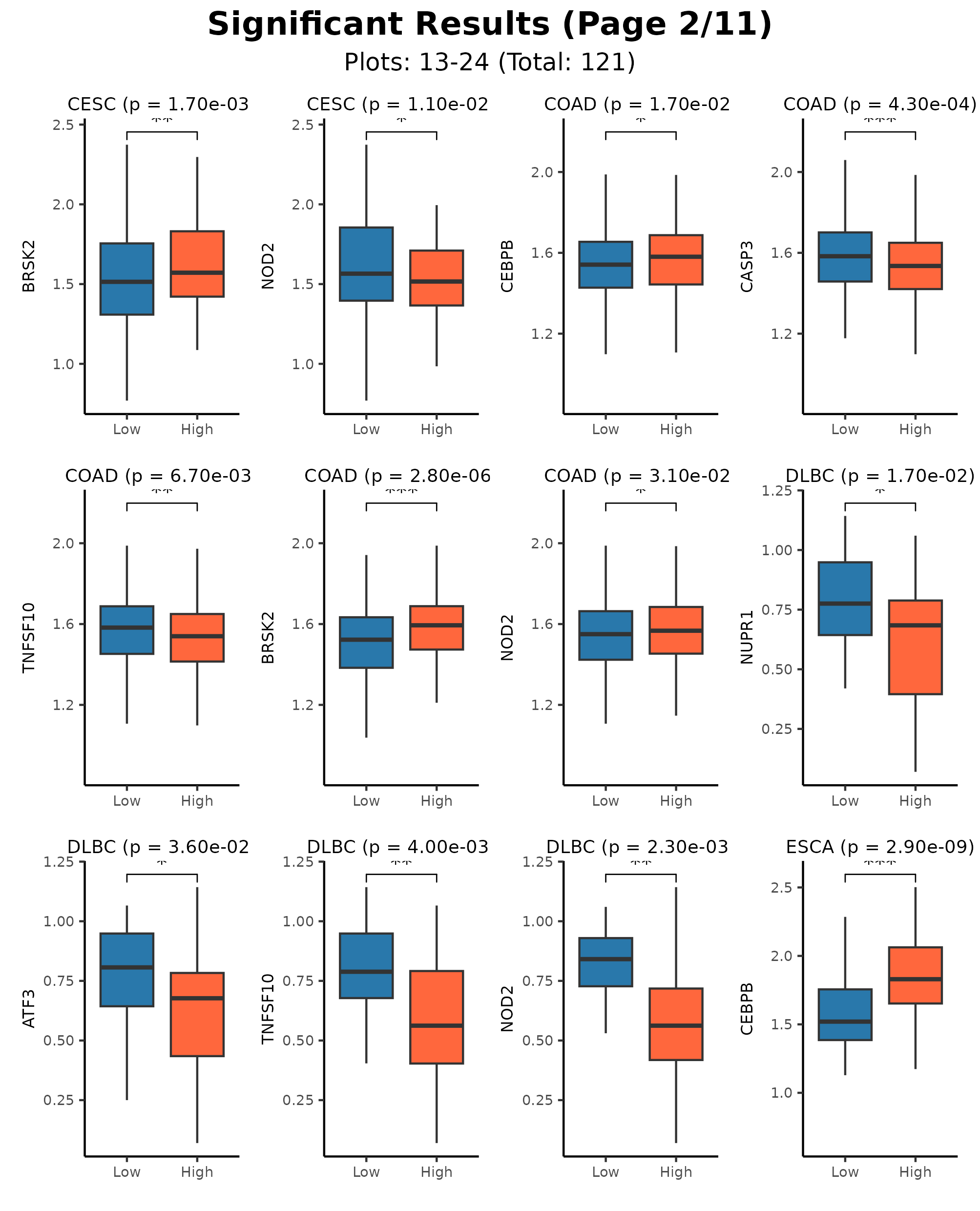

Supplement: Supplementary file 12 — Additional file12 (ZIP 3652 KB) [file 12672_2026_5126_MOESM12_ESM.zip › significant_results_page2.png]

# Significant Results (Page 3/11)

Plots: 25–36 (Total: 121)

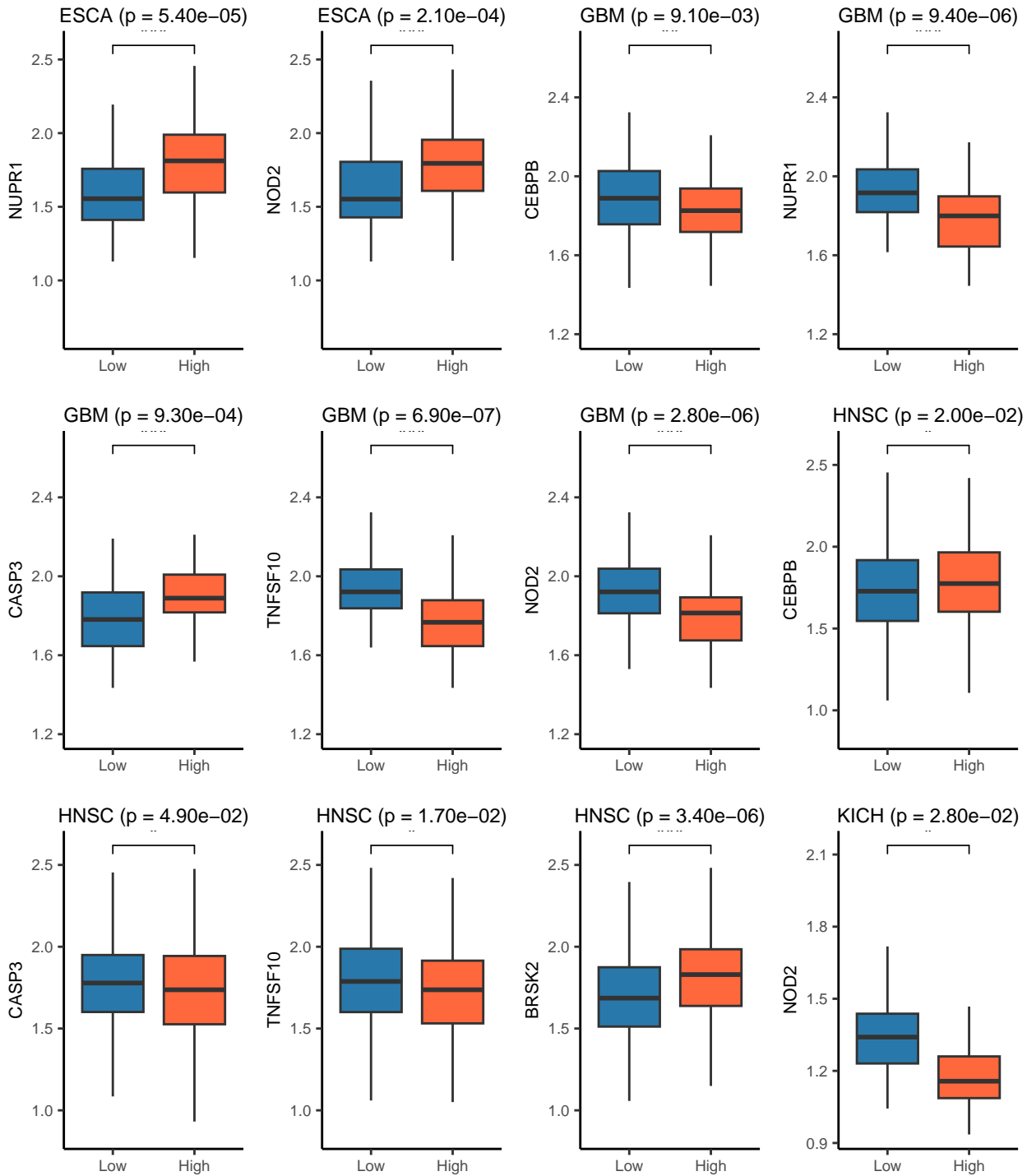

Supplement: Supplementary file 12 — Additional file12 (ZIP 3652 KB) [file 12672_2026_5126_MOESM12_ESM.zip › significant_results_page3.pdf]

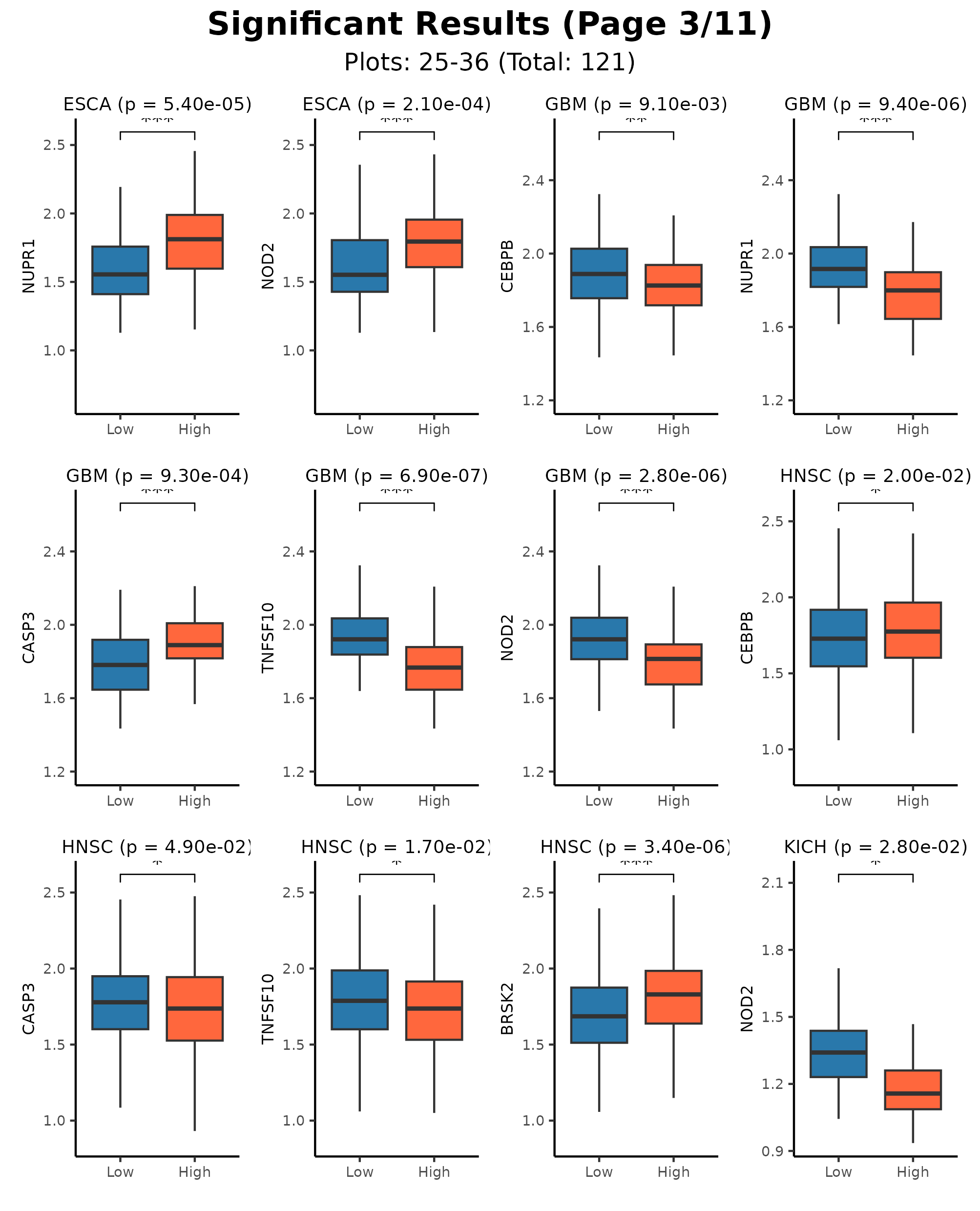

Supplement: Supplementary file 12 — Additional file12 (ZIP 3652 KB) [file 12672_2026_5126_MOESM12_ESM.zip › significant_results_page3.png]

# Significant Results (Page 4/11)

Plots: 37–48 (Total: 121)

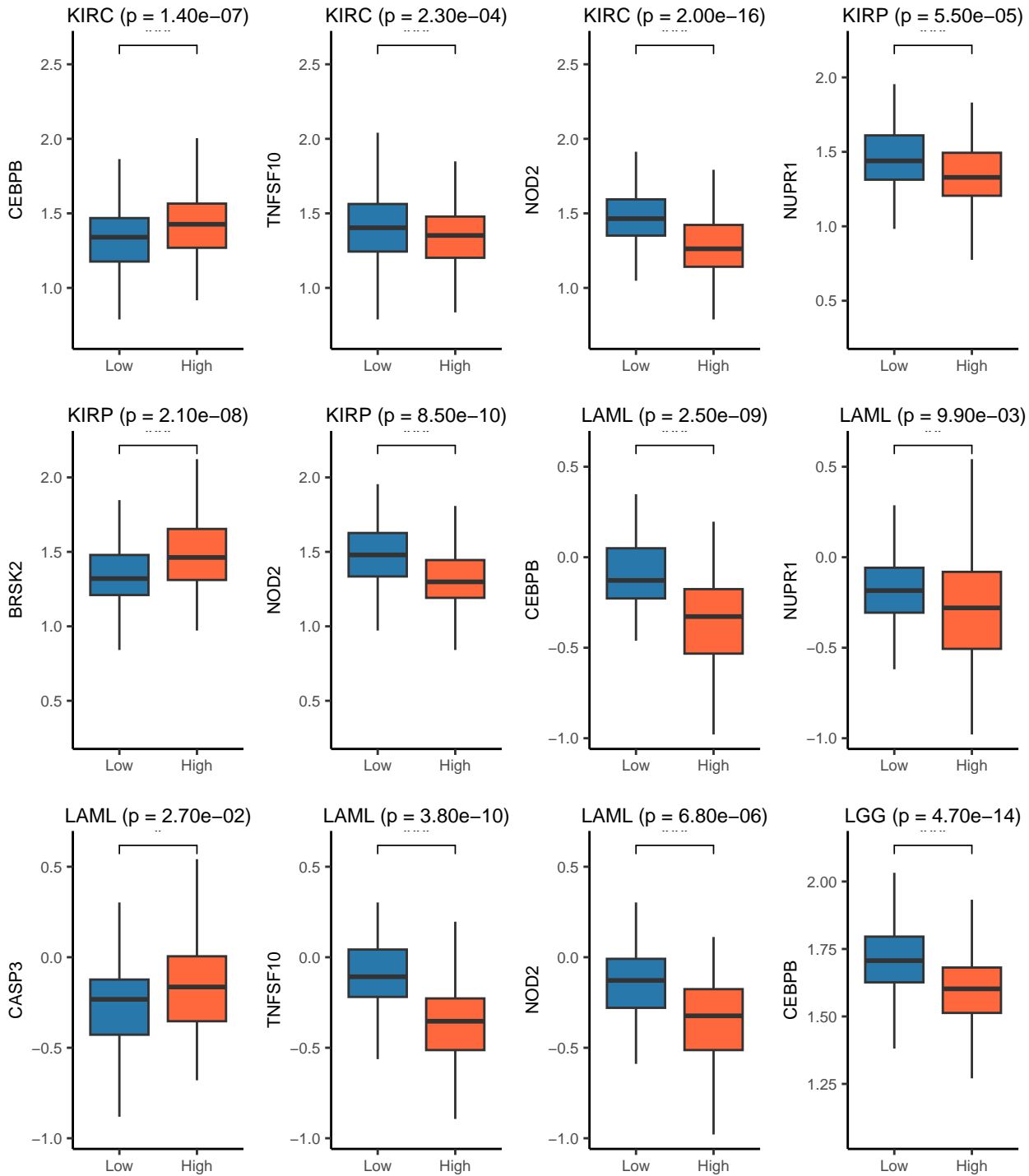

Supplement: Supplementary file 12 — Additional file12 (ZIP 3652 KB) [file 12672_2026_5126_MOESM12_ESM.zip › significant_results_page4.pdf]

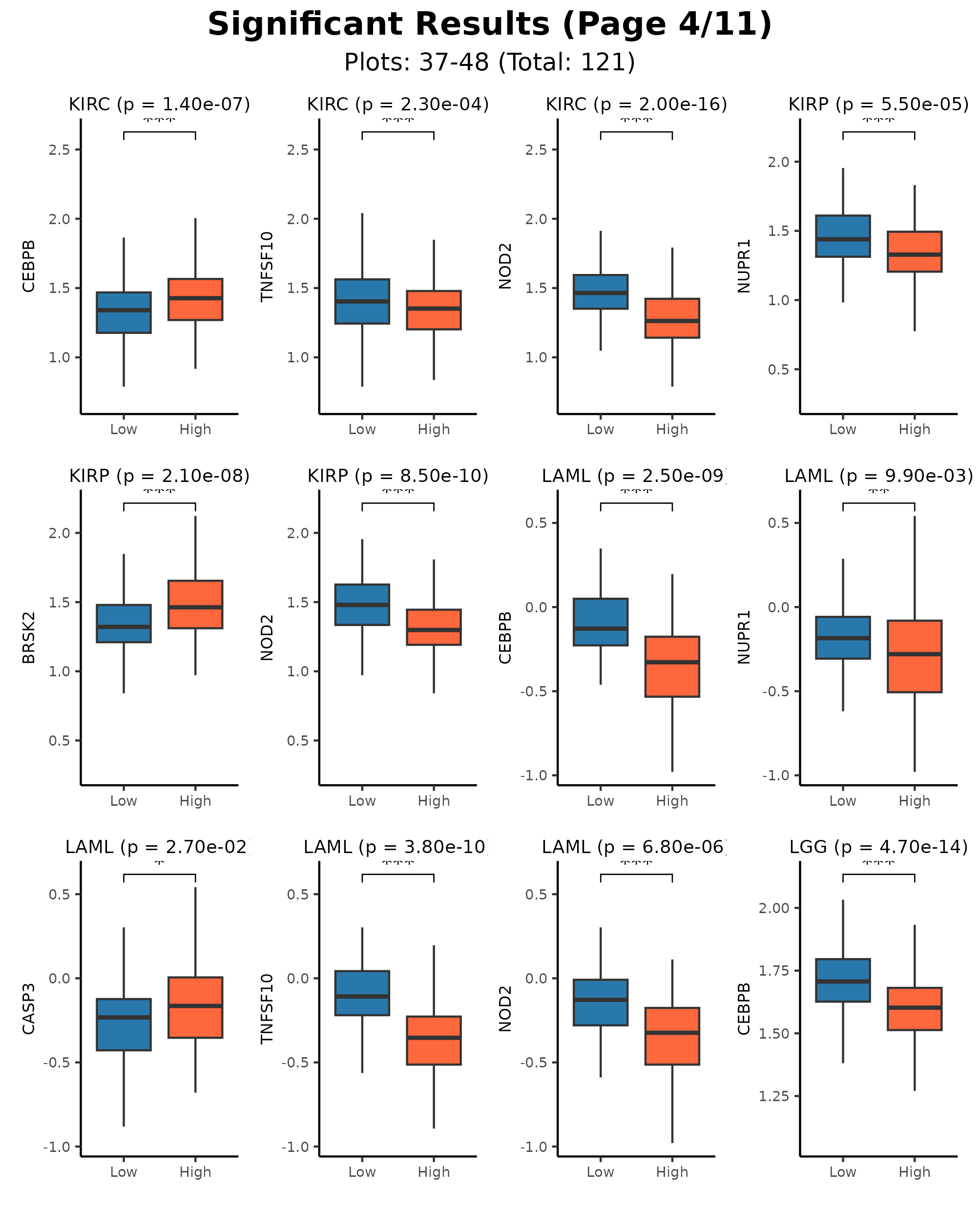

Supplement: Supplementary file 12 — Additional file12 (ZIP 3652 KB) [file 12672_2026_5126_MOESM12_ESM.zip › significant_results_page4.png]

# Significant Results (Page 5/11)

Plots: 49–60 (Total: 121)

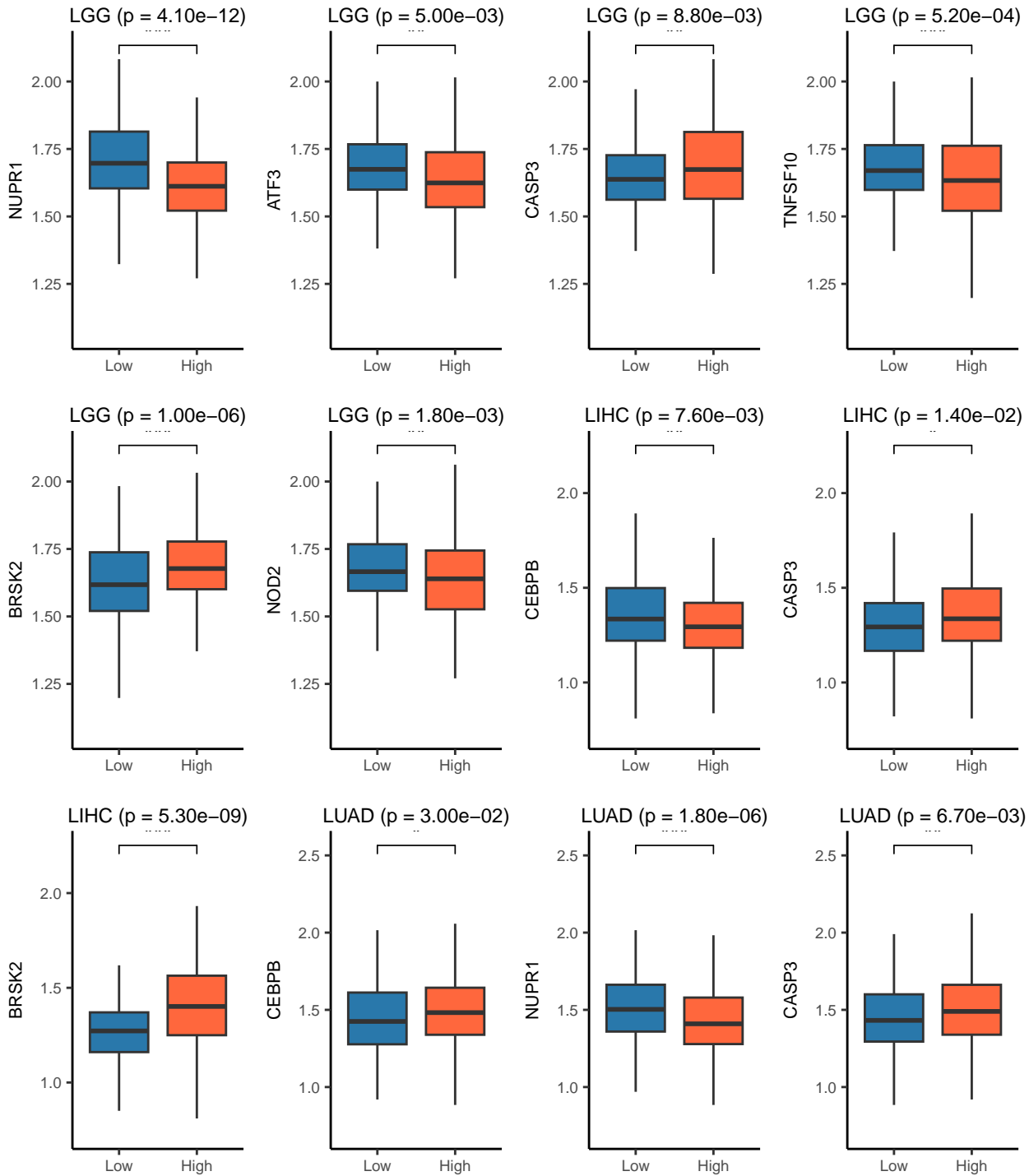

Supplement: Supplementary file 12 — Additional file12 (ZIP 3652 KB) [file 12672_2026_5126_MOESM12_ESM.zip › significant_results_page5.pdf]

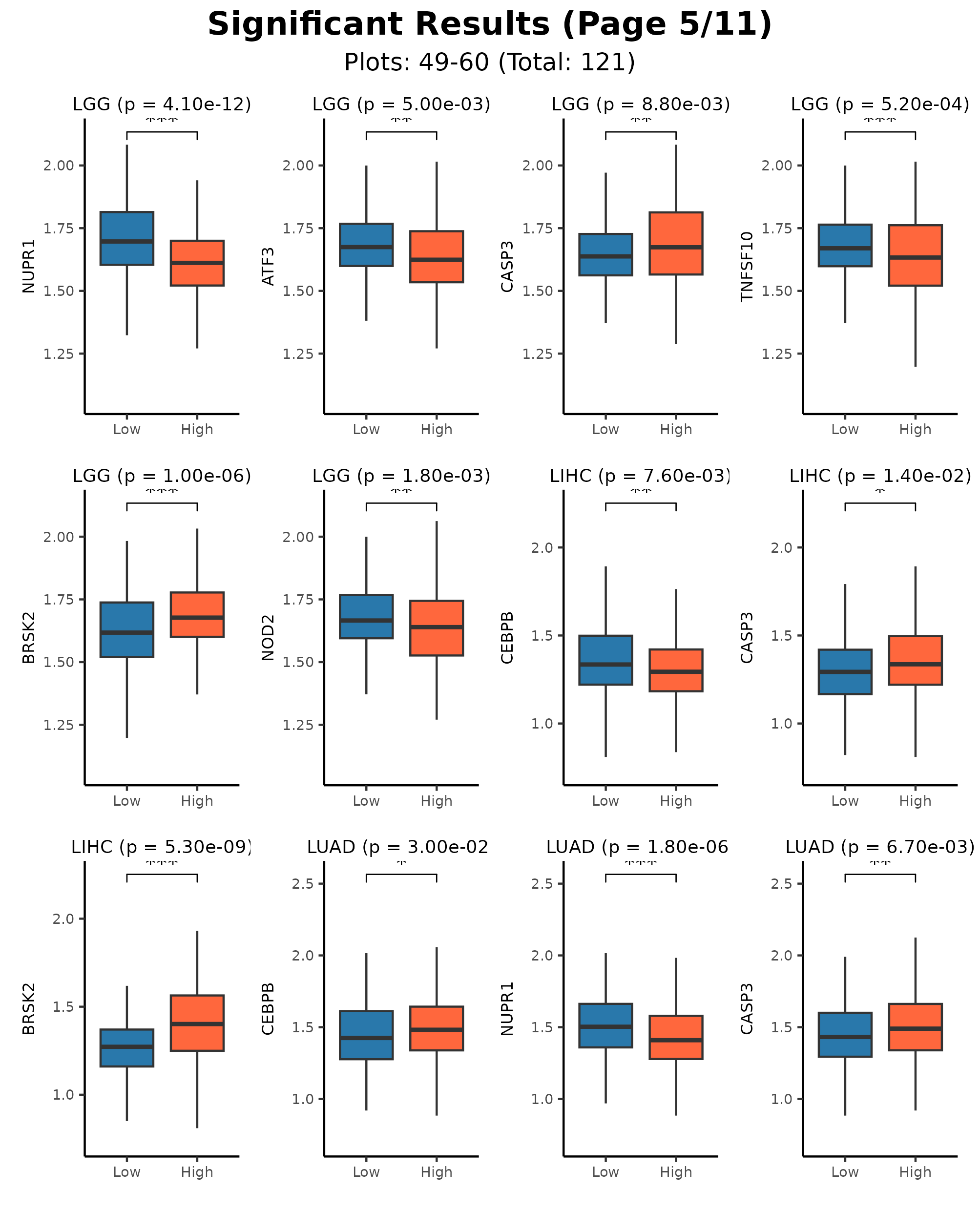

Supplement: Supplementary file 12 — Additional file12 (ZIP 3652 KB) [file 12672_2026_5126_MOESM12_ESM.zip › significant_results_page5.png]

# Significant Results (Page 6/11)

Plots: 61–72 (Total: 121)

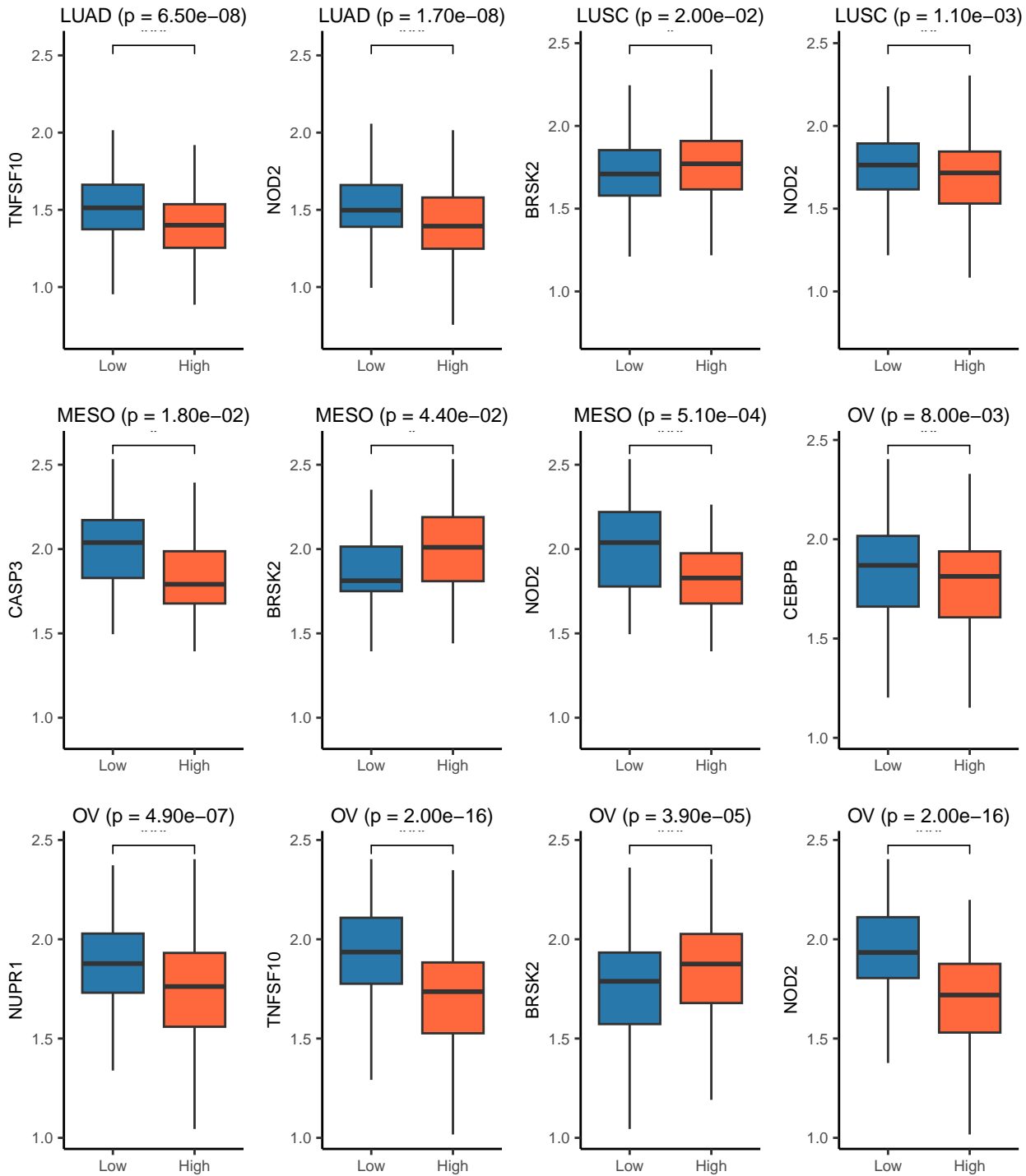

Supplement: Supplementary file 12 — Additional file12 (ZIP 3652 KB) [file 12672_2026_5126_MOESM12_ESM.zip › significant_results_page6.pdf]

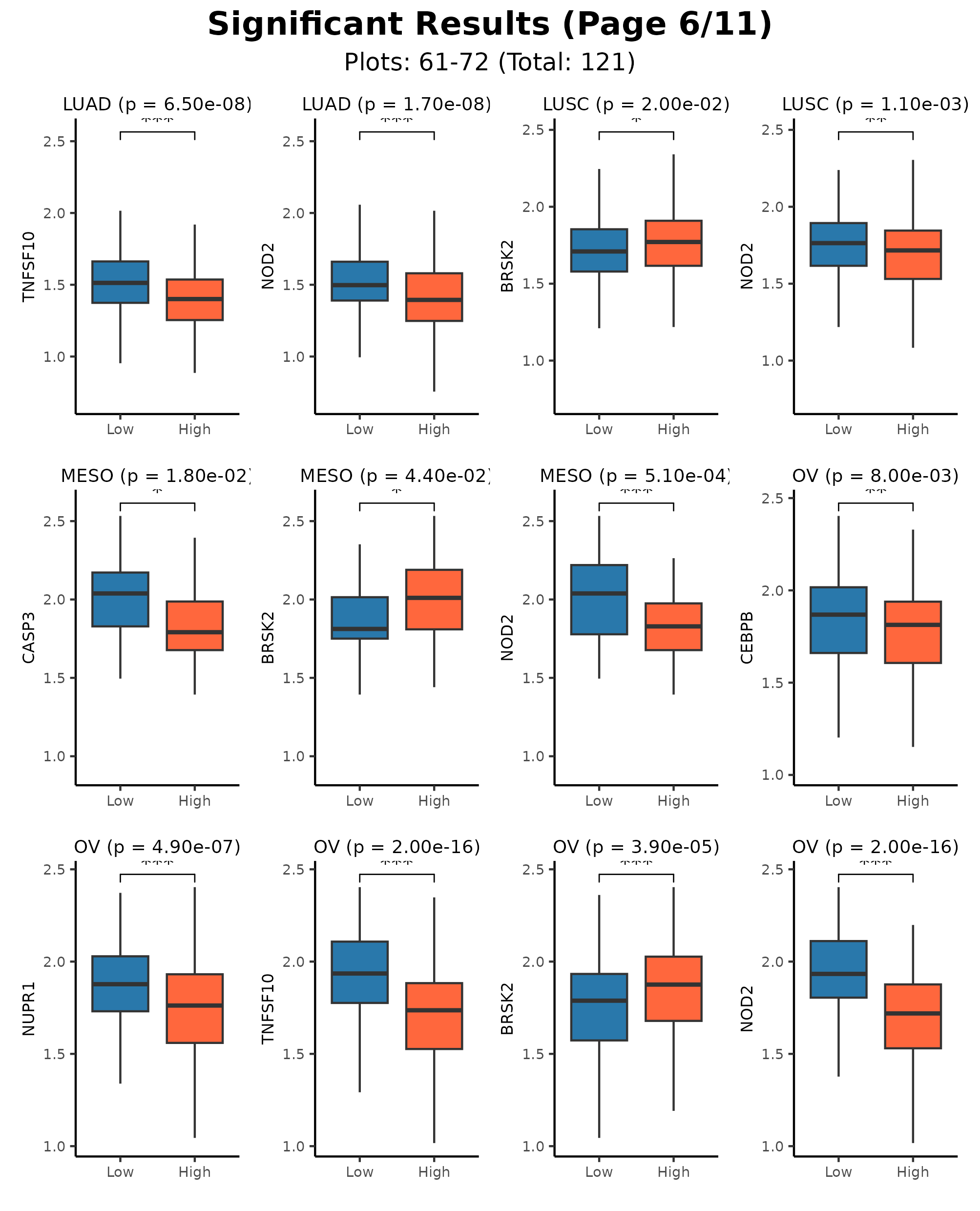

Supplement: Supplementary file 12 — Additional file12 (ZIP 3652 KB) [file 12672_2026_5126_MOESM12_ESM.zip › significant_results_page6.png]

# Significant Results (Page 7/11)

Plots: 73–84 (Total: 121)

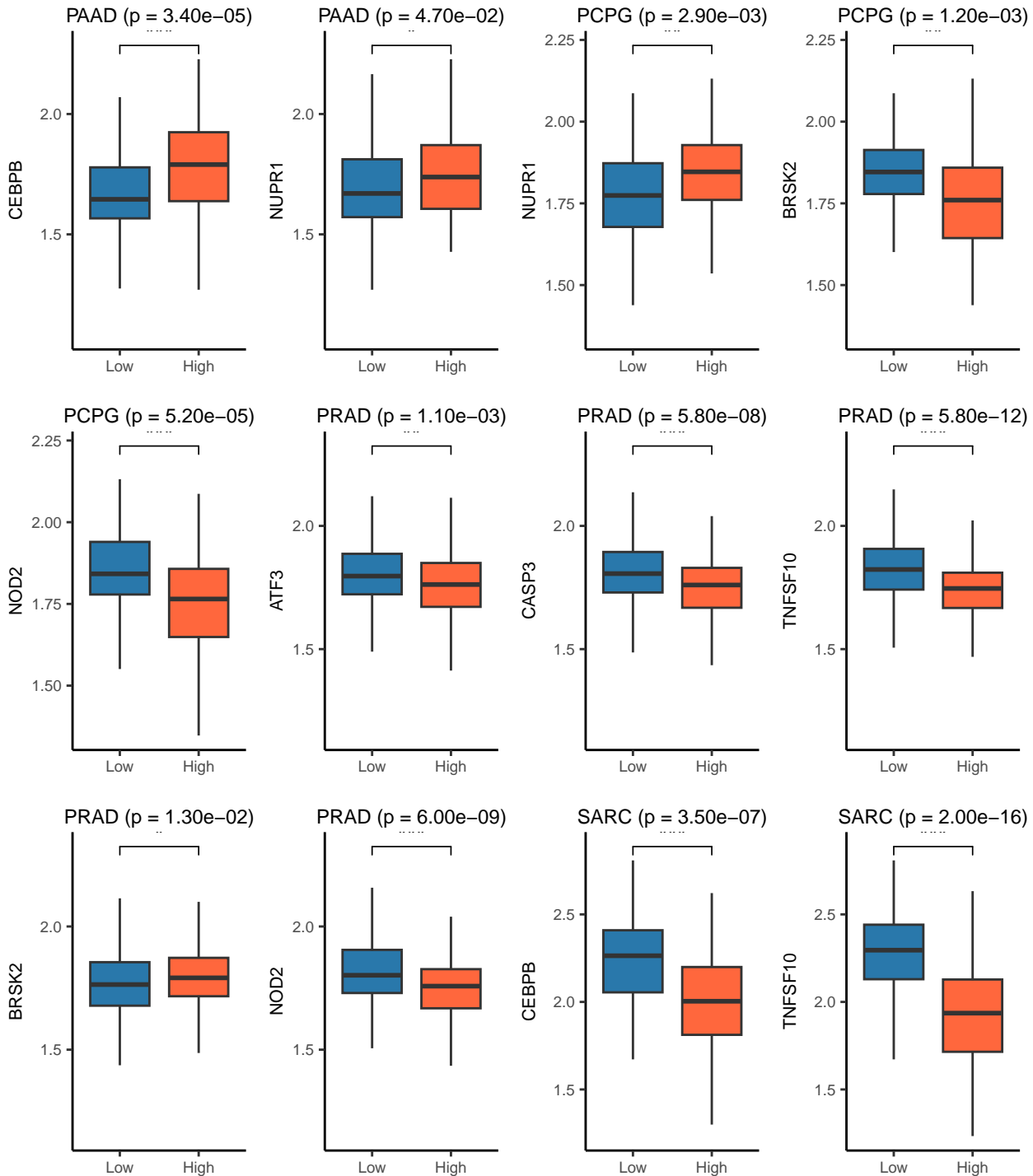

Supplement: Supplementary file 12 — Additional file12 (ZIP 3652 KB) [file 12672_2026_5126_MOESM12_ESM.zip › significant_results_page7.pdf]

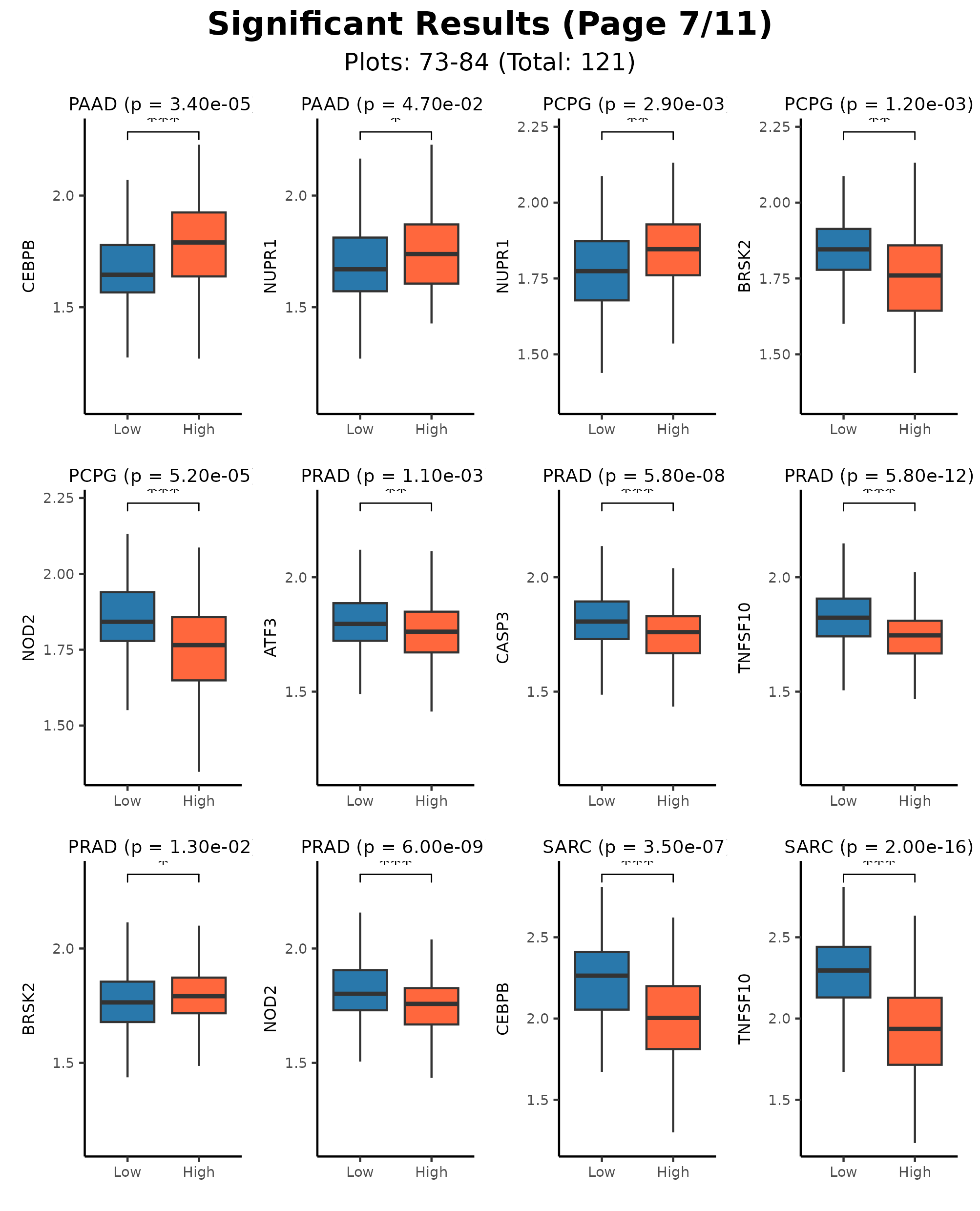

Supplement: Supplementary file 12 — Additional file12 (ZIP 3652 KB) [file 12672_2026_5126_MOESM12_ESM.zip › significant_results_page7.png]

# Significant Results (Page 8/11)

Plots: 85–96 (Total: 121)

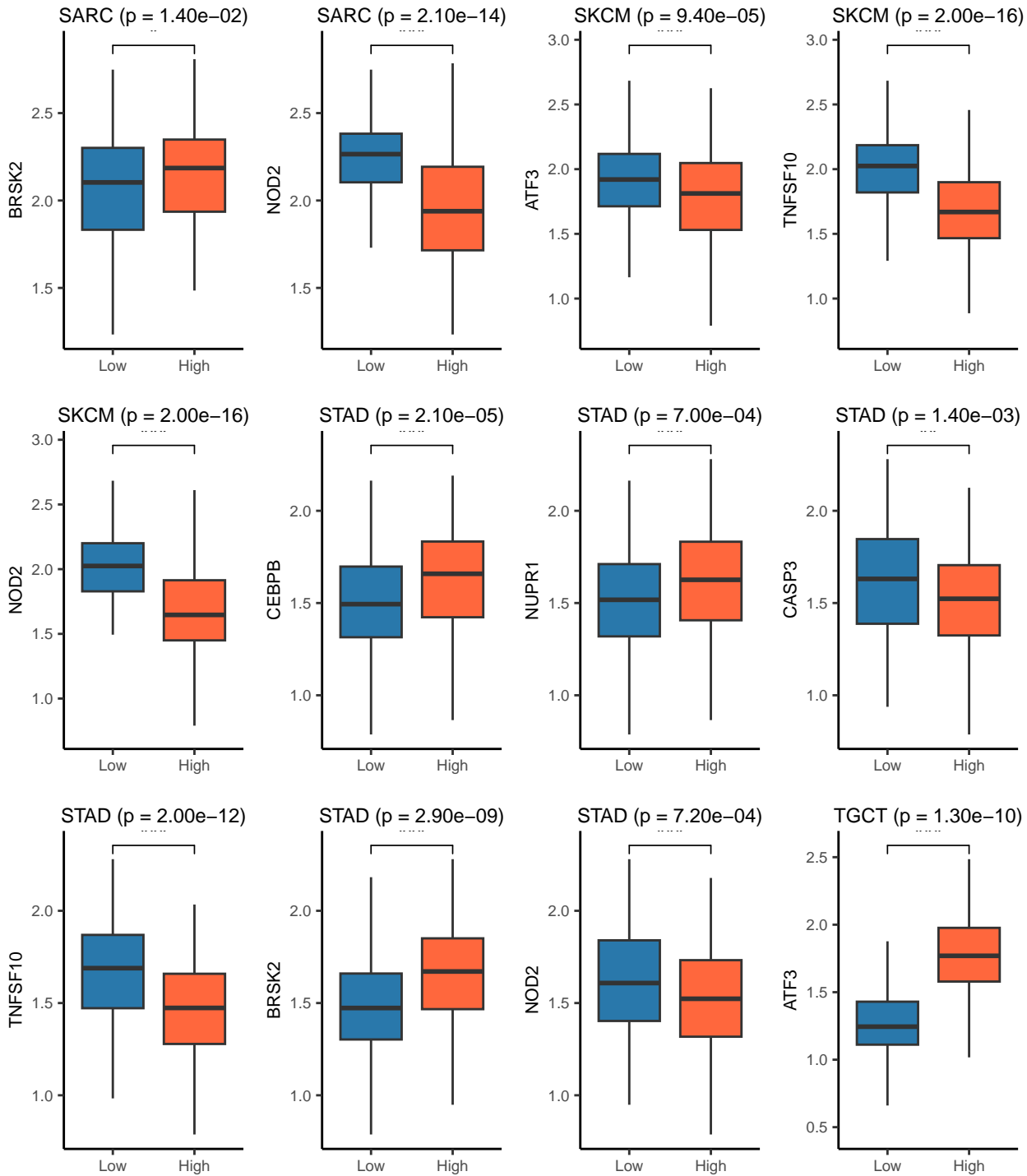

Supplement: Supplementary file 12 — Additional file12 (ZIP 3652 KB) [file 12672_2026_5126_MOESM12_ESM.zip › significant_results_page8.pdf]

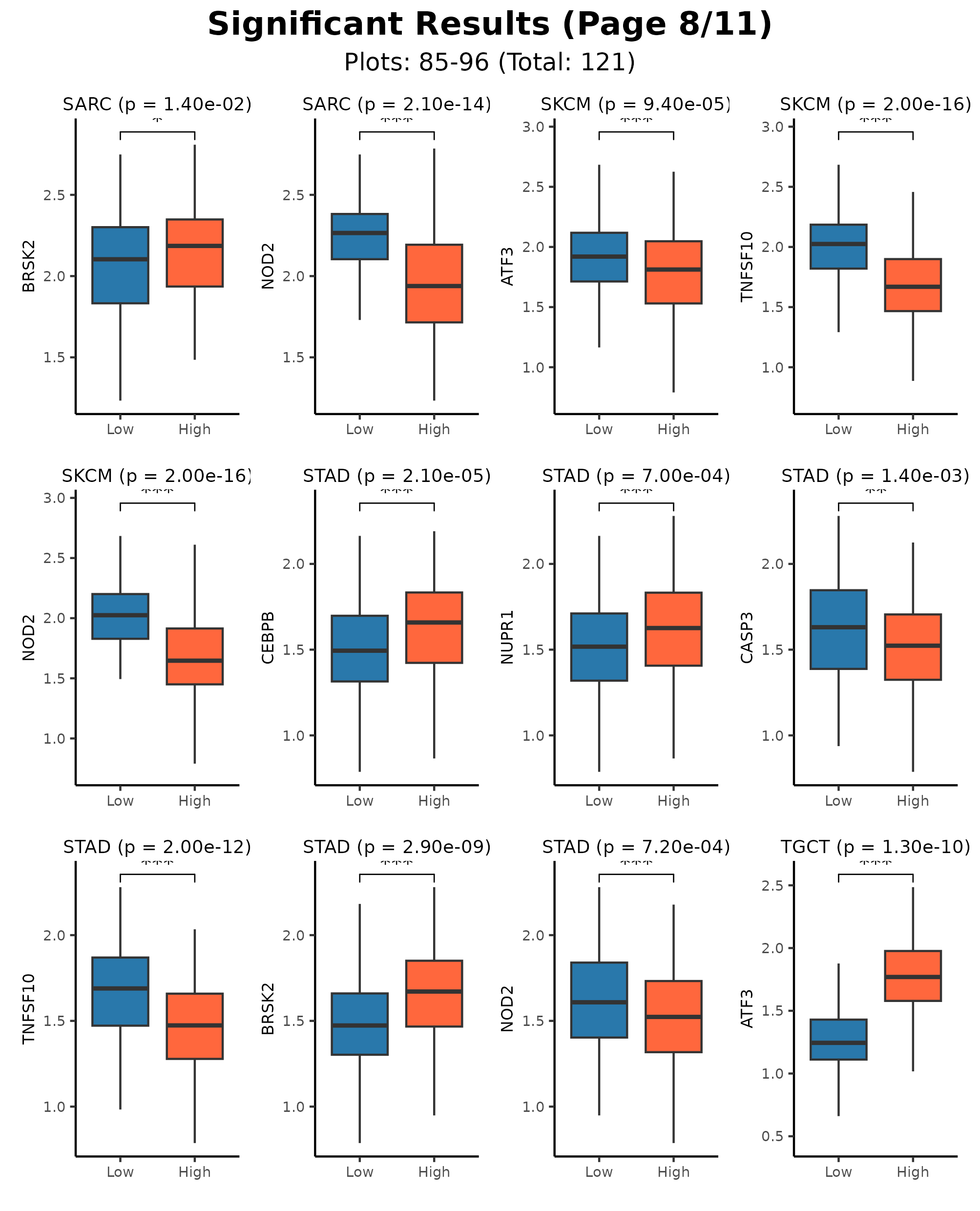

Supplement: Supplementary file 12 — Additional file12 (ZIP 3652 KB) [file 12672_2026_5126_MOESM12_ESM.zip › significant_results_page8.png]

# Significant Results (Page 9/11)

Plots: 97–108 (Total: 121)

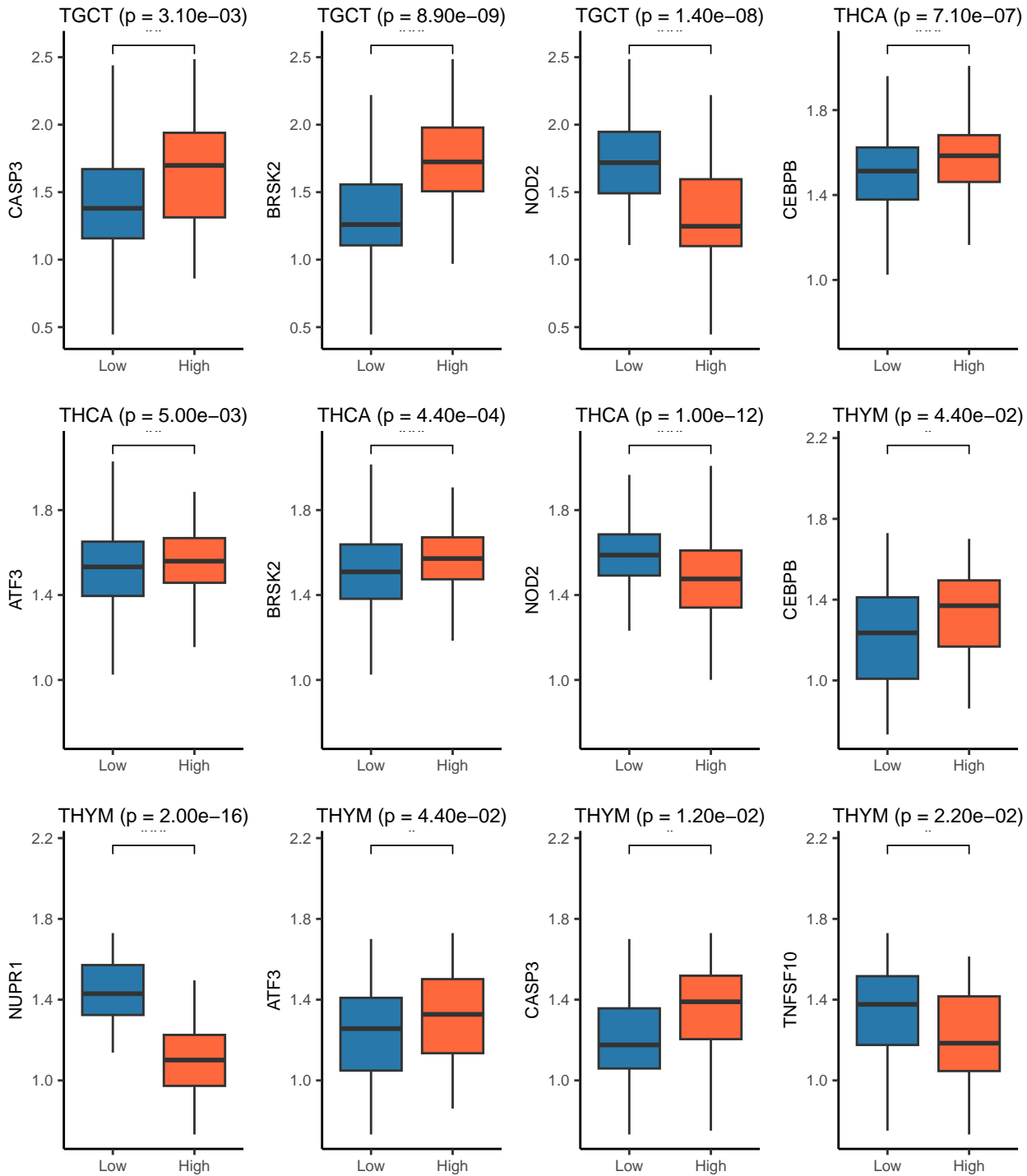

Supplement: Supplementary file 12 — Additional file12 (ZIP 3652 KB) [file 12672_2026_5126_MOESM12_ESM.zip › significant_results_page9.pdf]

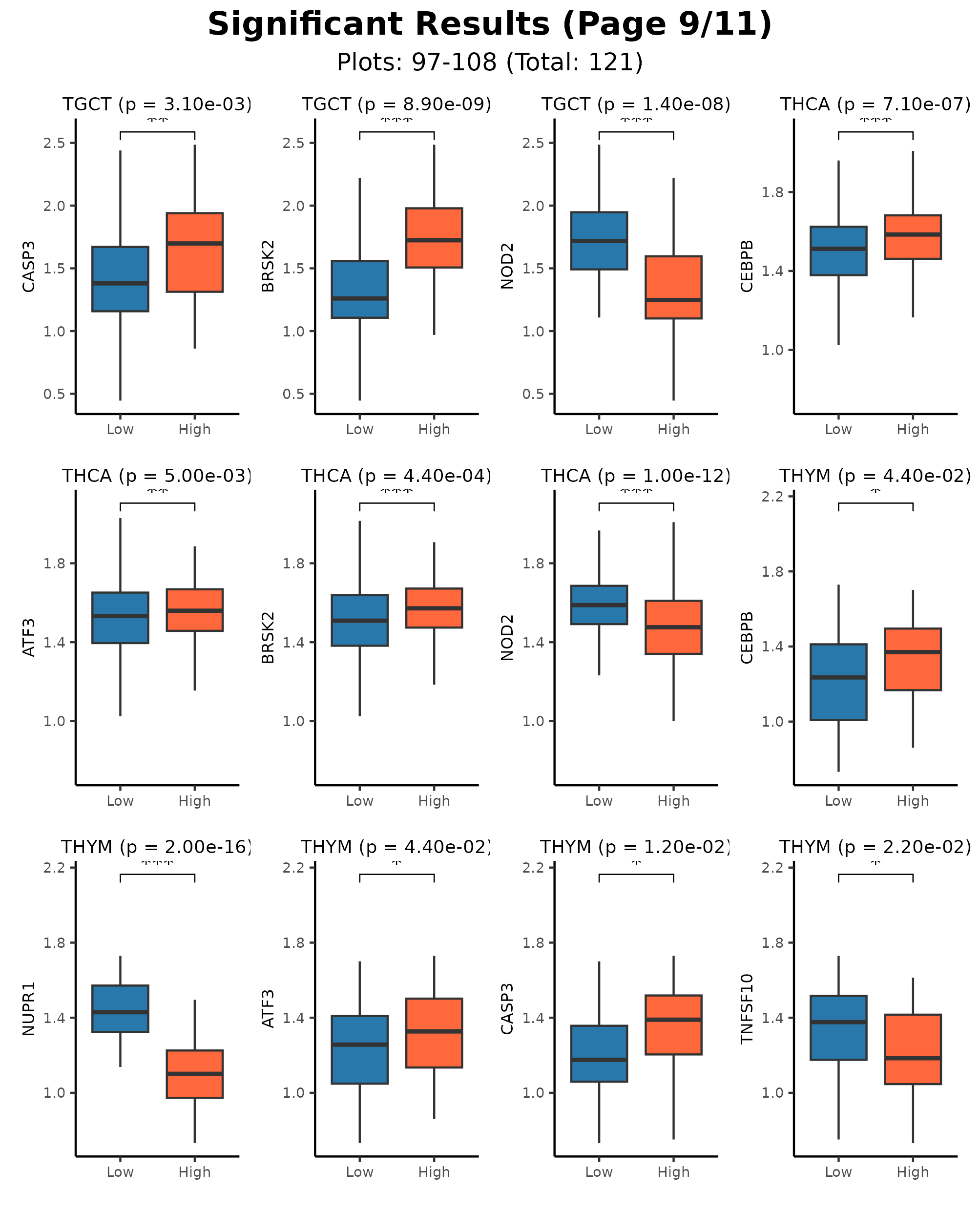

Supplement: Supplementary file 12 — Additional file12 (ZIP 3652 KB) [file 12672_2026_5126_MOESM12_ESM.zip › significant_results_page9.png]

Cancer Type: SKCM

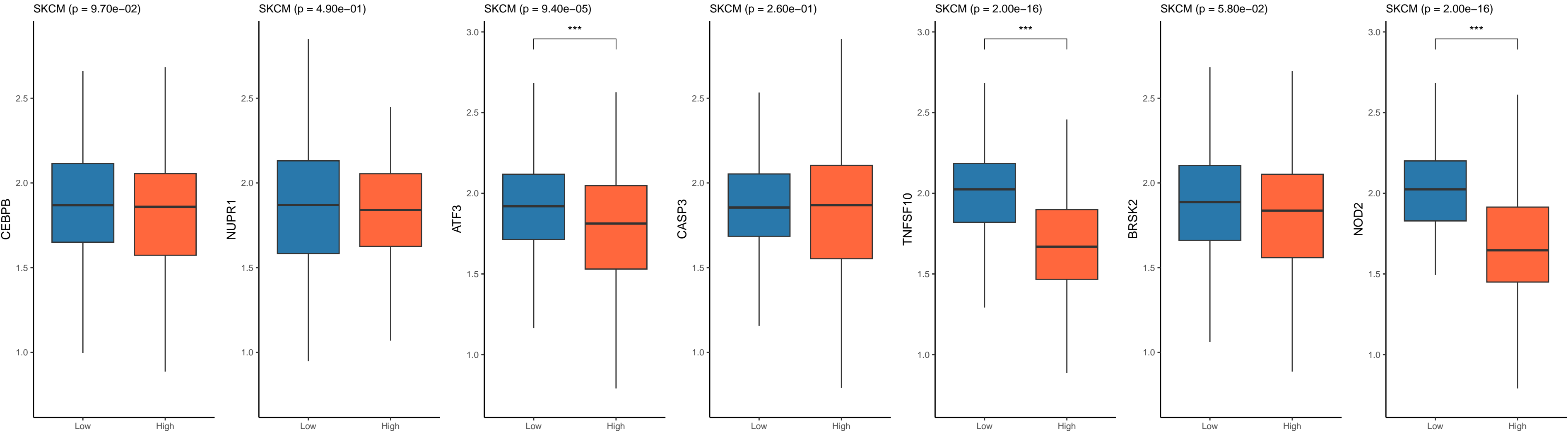

Supplement: Supplementary file 12 — Additional file12 (ZIP 3652 KB) [file 12672_2026_5126_MOESM12_ESM.zip › SKCM_combined.pdf]

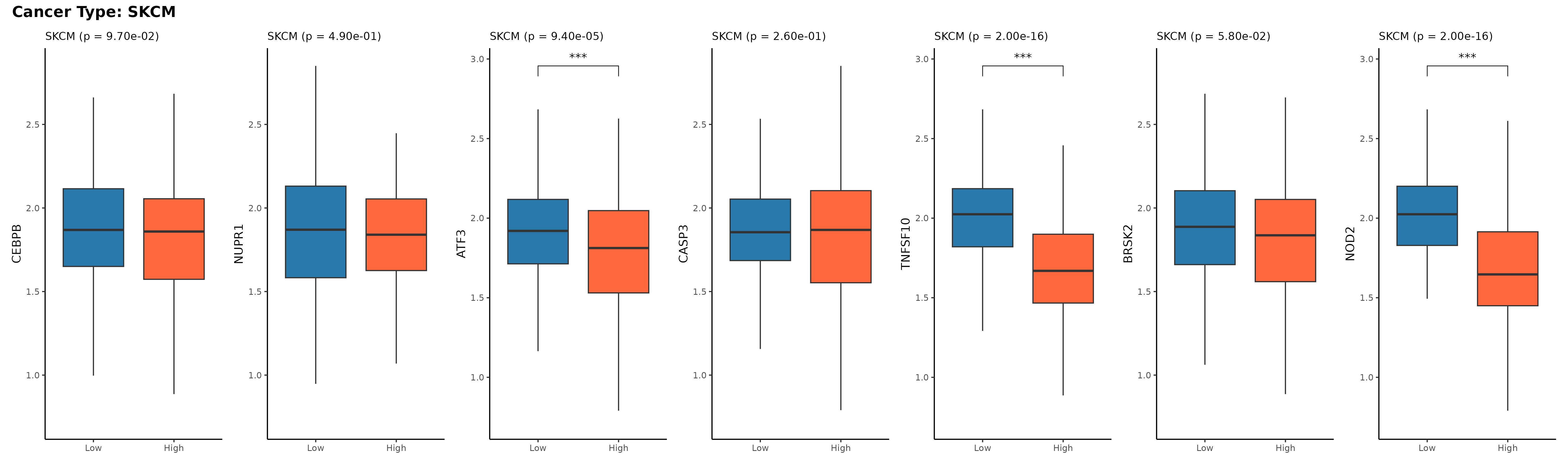

Supplement: Supplementary file 12 — Additional file12 (ZIP 3652 KB) [file 12672_2026_5126_MOESM12_ESM.zip › SKCM_combined.png]

**Cancer Type: STAD**

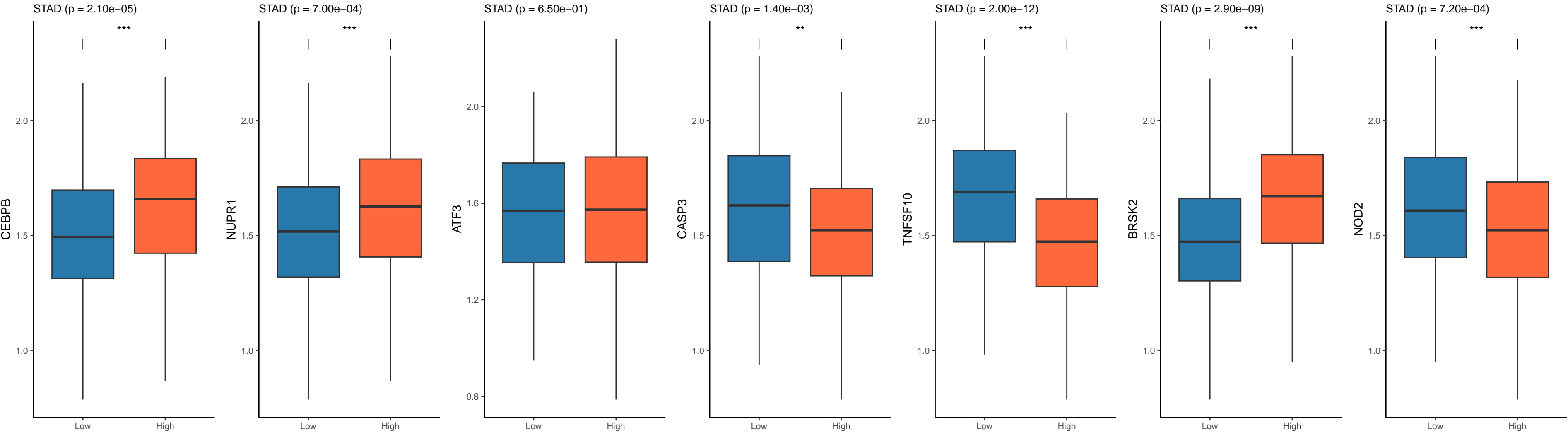

Supplement: Supplementary file 12 — Additional file12 (ZIP 3652 KB) [file 12672_2026_5126_MOESM12_ESM.zip › STAD_combined.pdf]

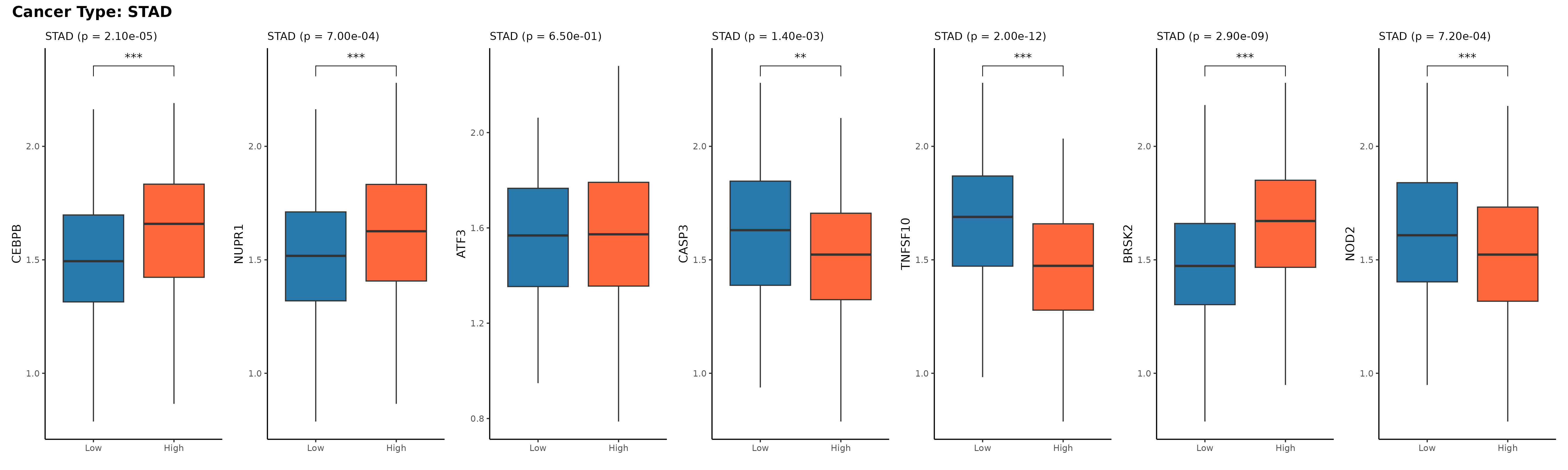

Supplement: Supplementary file 12 — Additional file12 (ZIP 3652 KB) [file 12672_2026_5126_MOESM12_ESM.zip › STAD_combined.png]

Cancer Type: TGCT

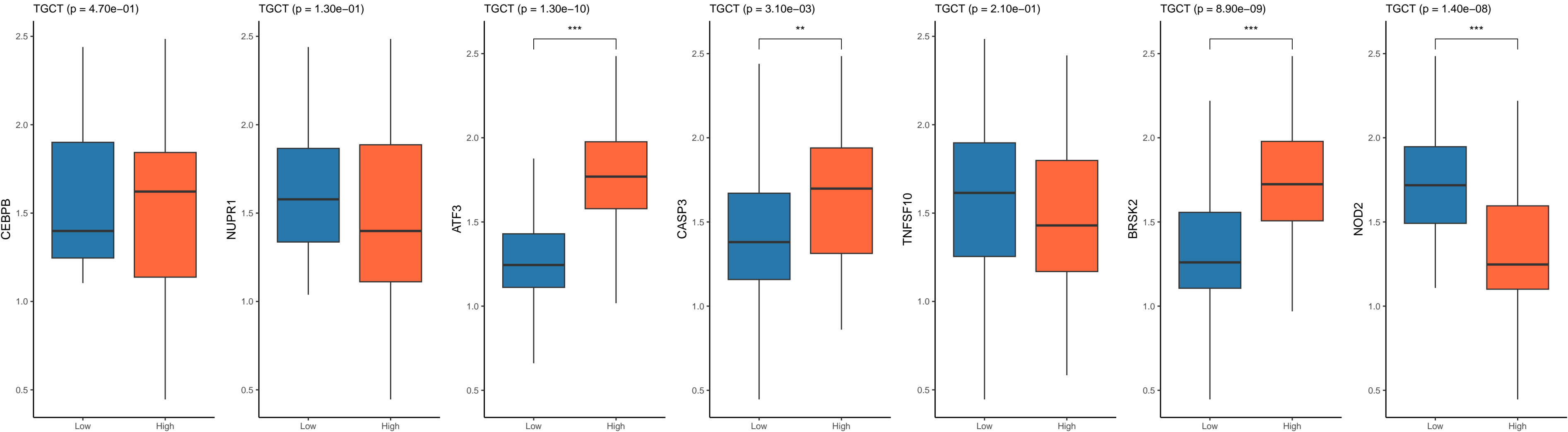

Supplement: Supplementary file 12 — Additional file12 (ZIP 3652 KB) [file 12672_2026_5126_MOESM12_ESM.zip › TGCT_combined.pdf]

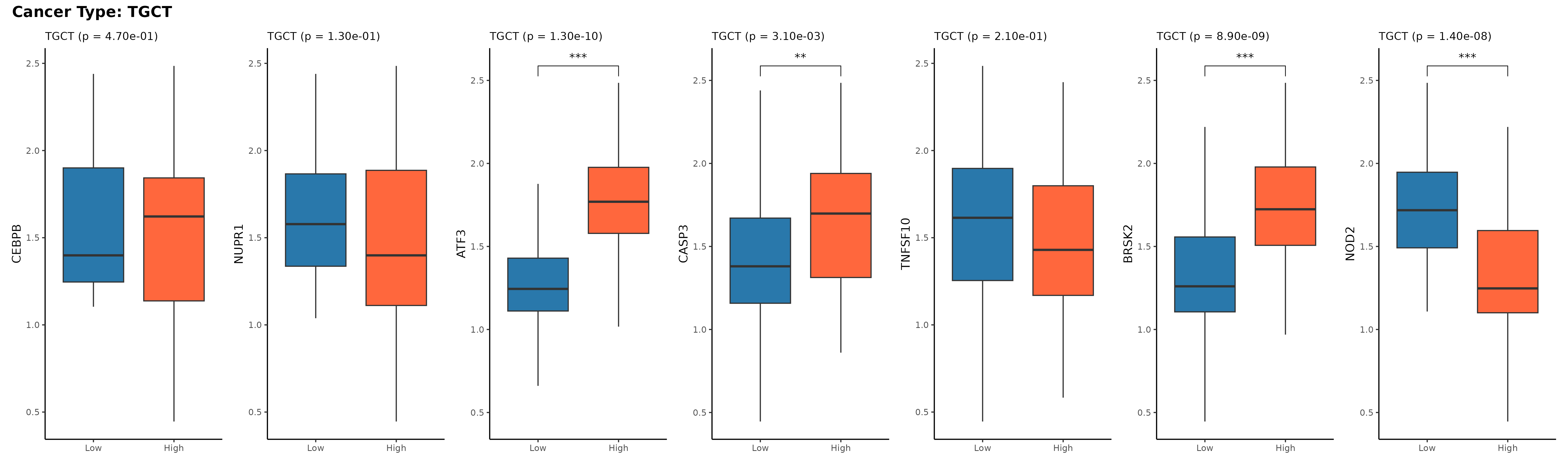

Supplement: Supplementary file 12 — Additional file12 (ZIP 3652 KB) [file 12672_2026_5126_MOESM12_ESM.zip › TGCT_combined.png]

Cancer Type: THCA

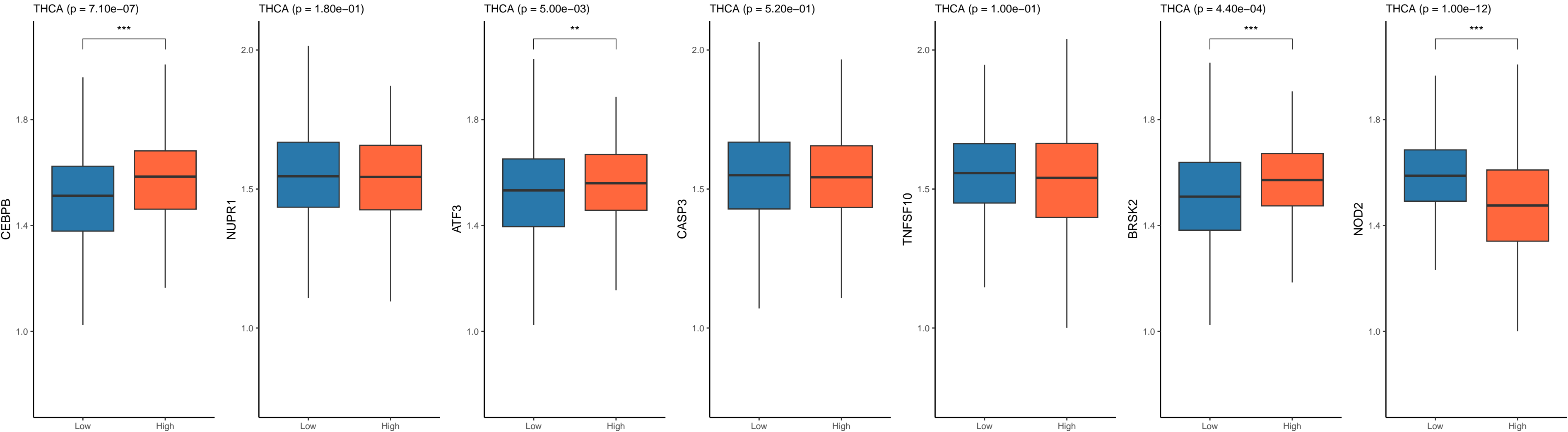

Supplement: Supplementary file 12 — Additional file12 (ZIP 3652 KB) [file 12672_2026_5126_MOESM12_ESM.zip › THCA_combined.pdf]
