# Supplementary material for: Integration of 117 machine learning algorithms and single-cell transcriptomics identifies macrophage polarization and ER stress signatures for cancer prognosis and precision therapy
Source: Discov Oncol. 2026 Apr 30;17:917. doi: 10.1007/s12672-026-05126-6 (PMC13275951; doi:10.1007/s12672-026-05126-6)

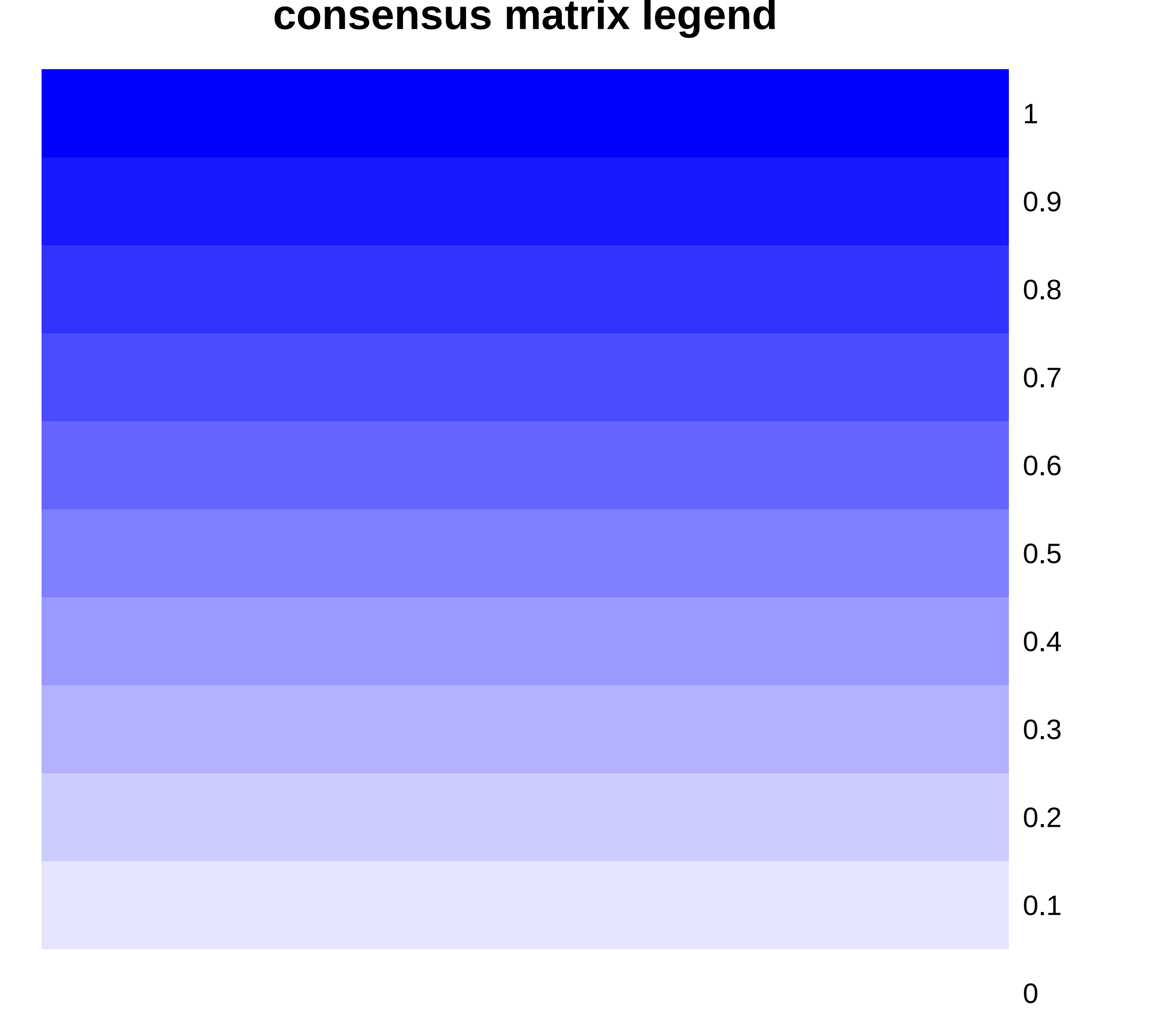

consensus matrix legend
1
0.9
0.8
0.7
0.6
0.5
0.4
0.3
0.2
0.1
0

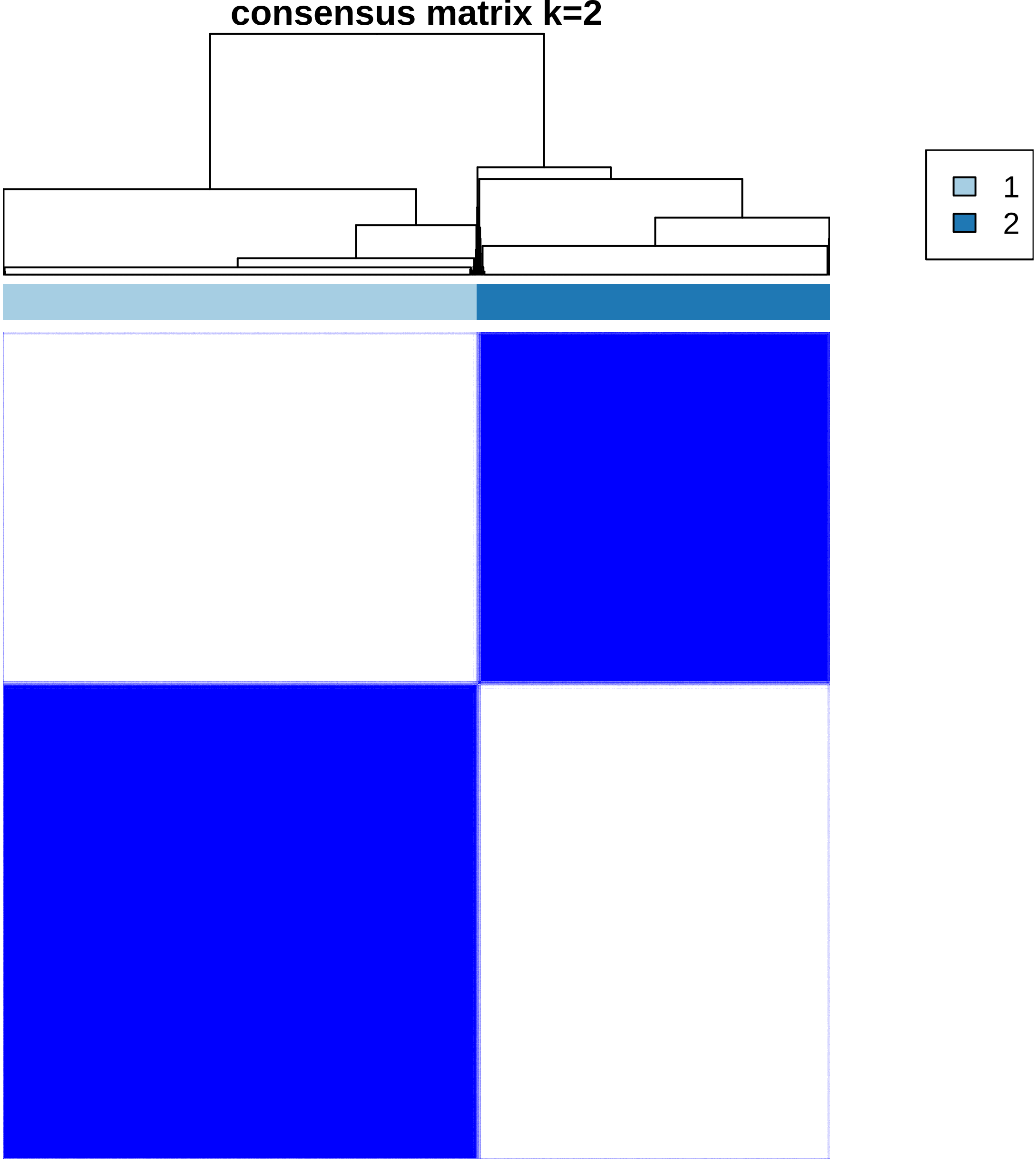
consensus matrix k=2
1
2

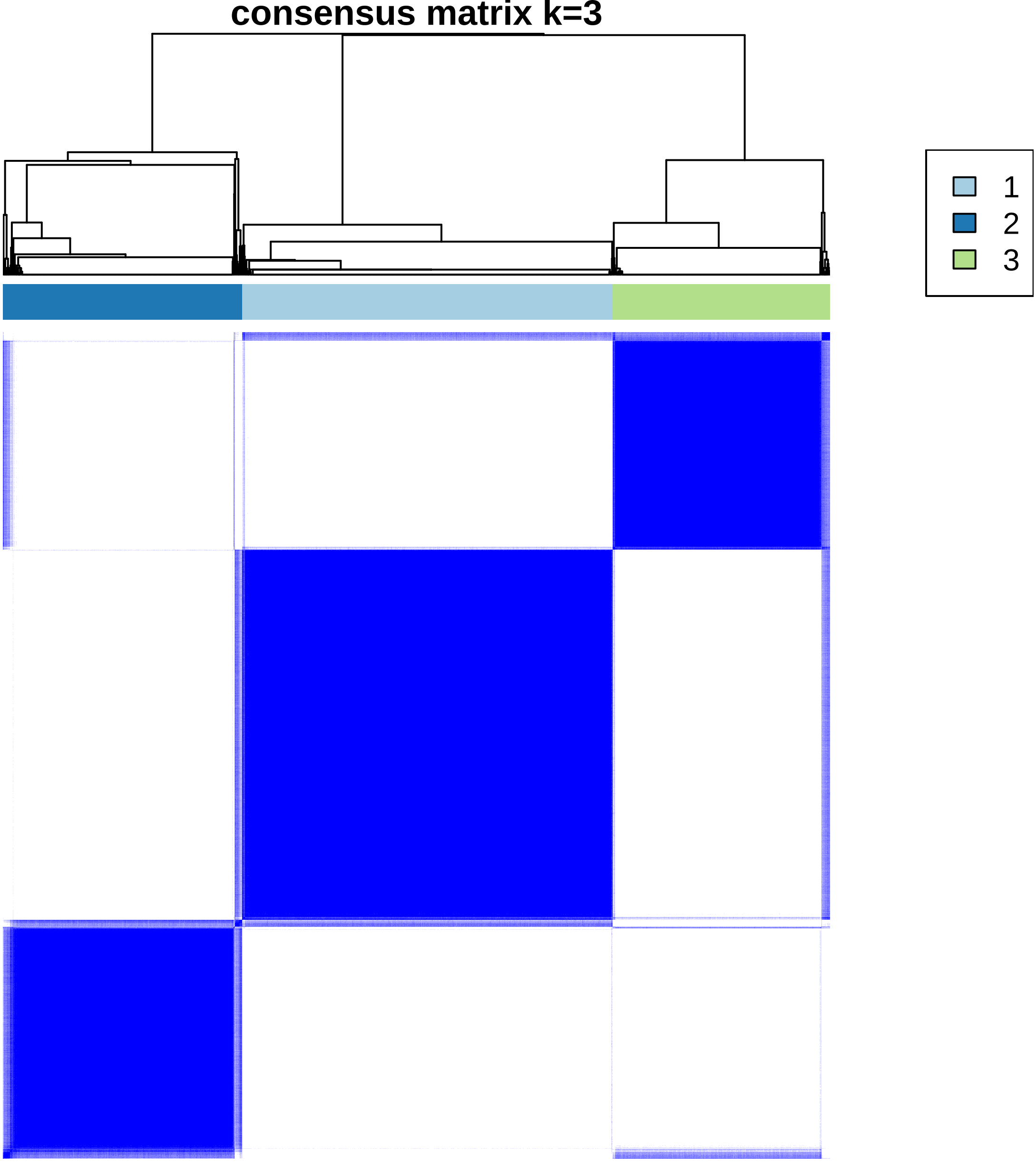
consensus matrix k=3
1
2
3

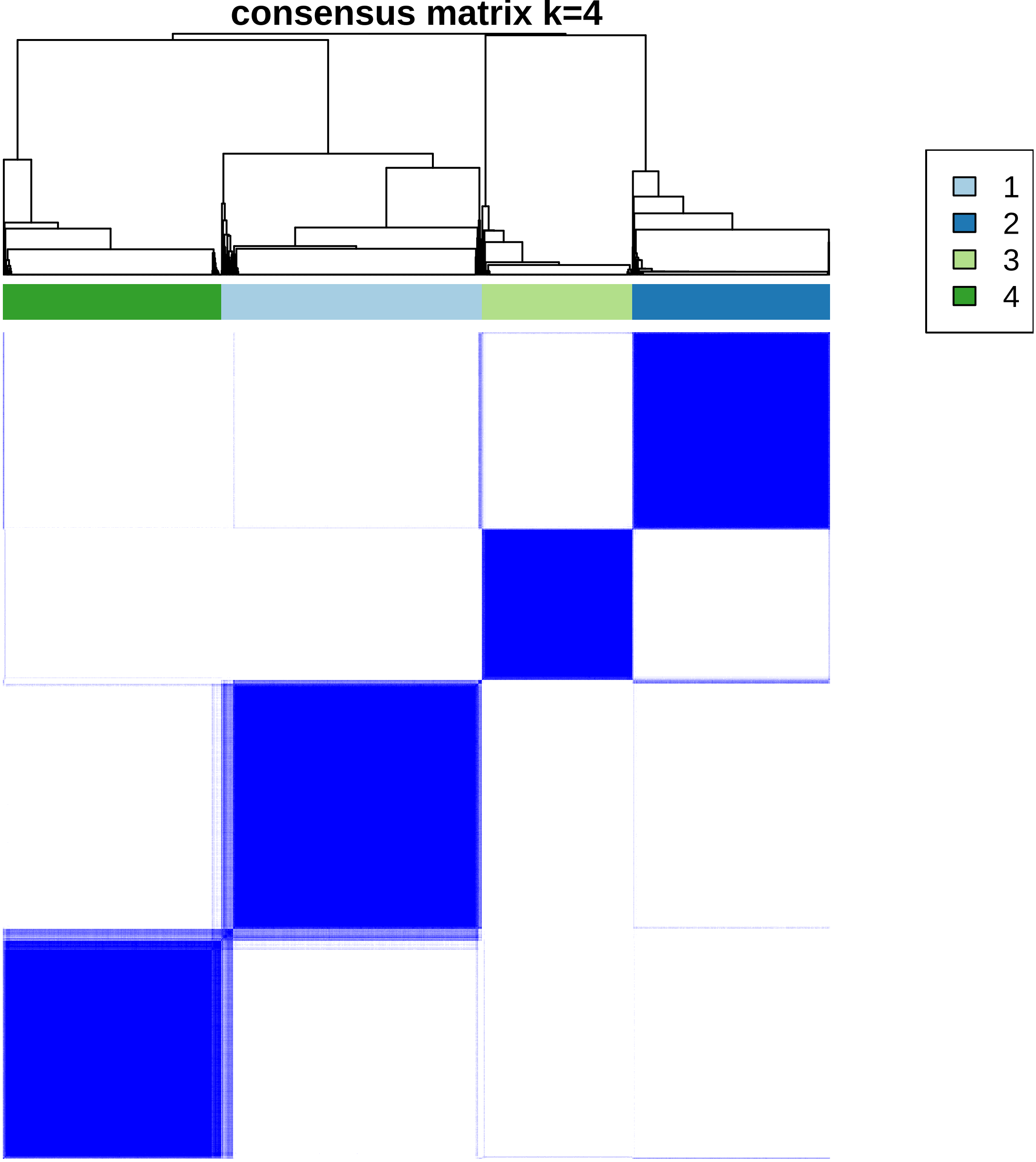
consensus matrix k=4
1
2
3
4

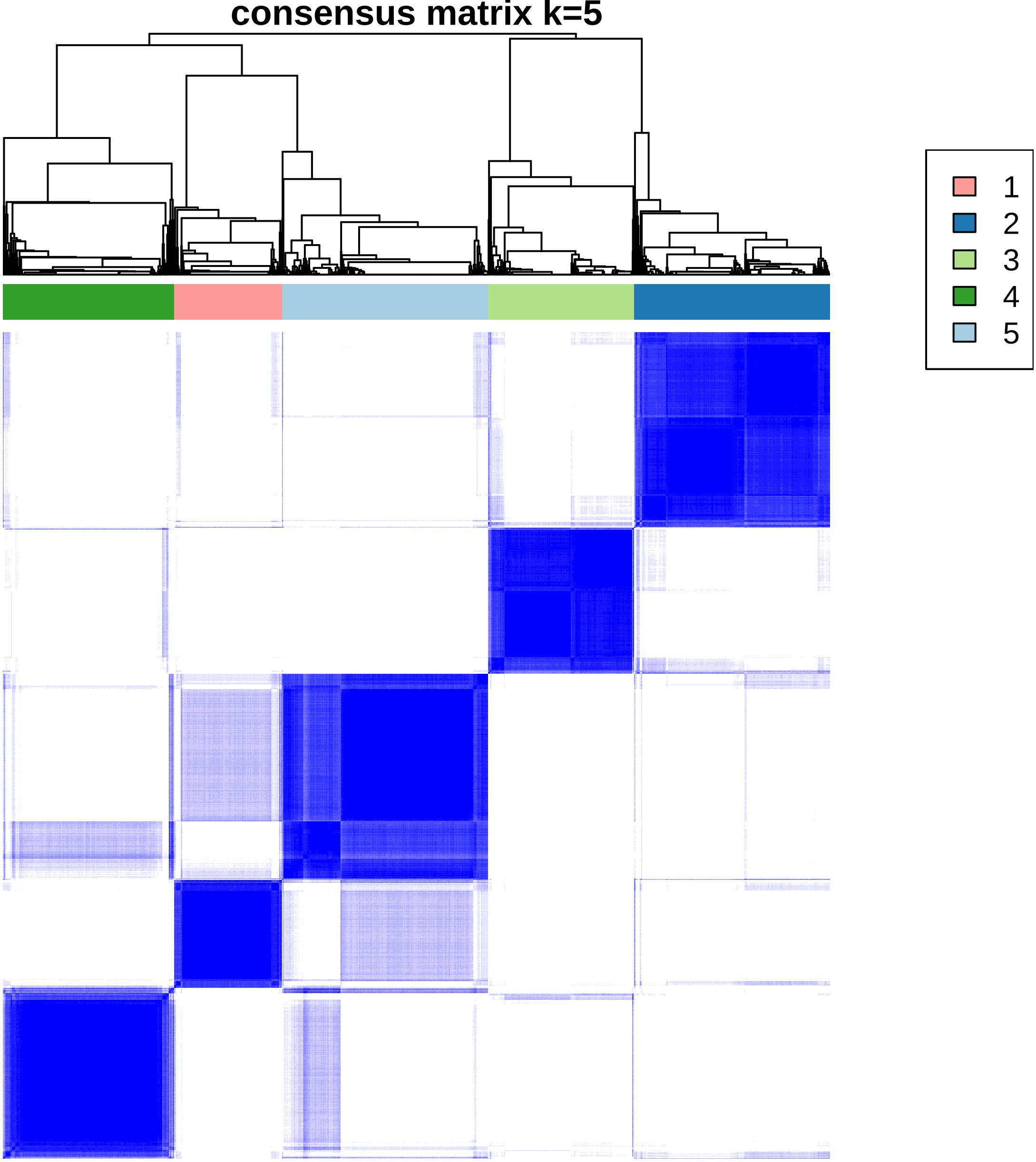
consensus matrix k=5
1
2
3
4
5

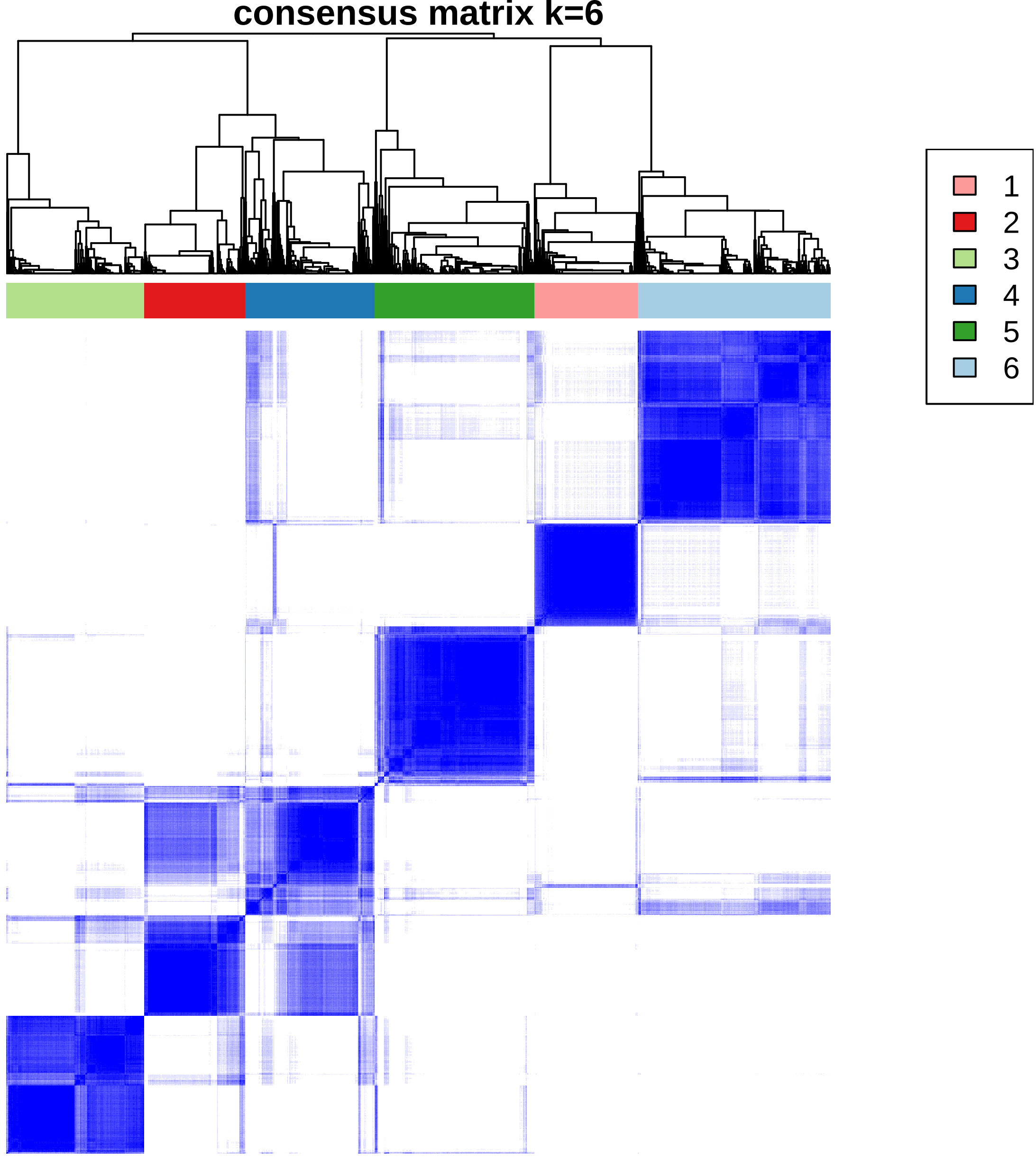
consensus matrix k=6
1
2
3
4
5
6

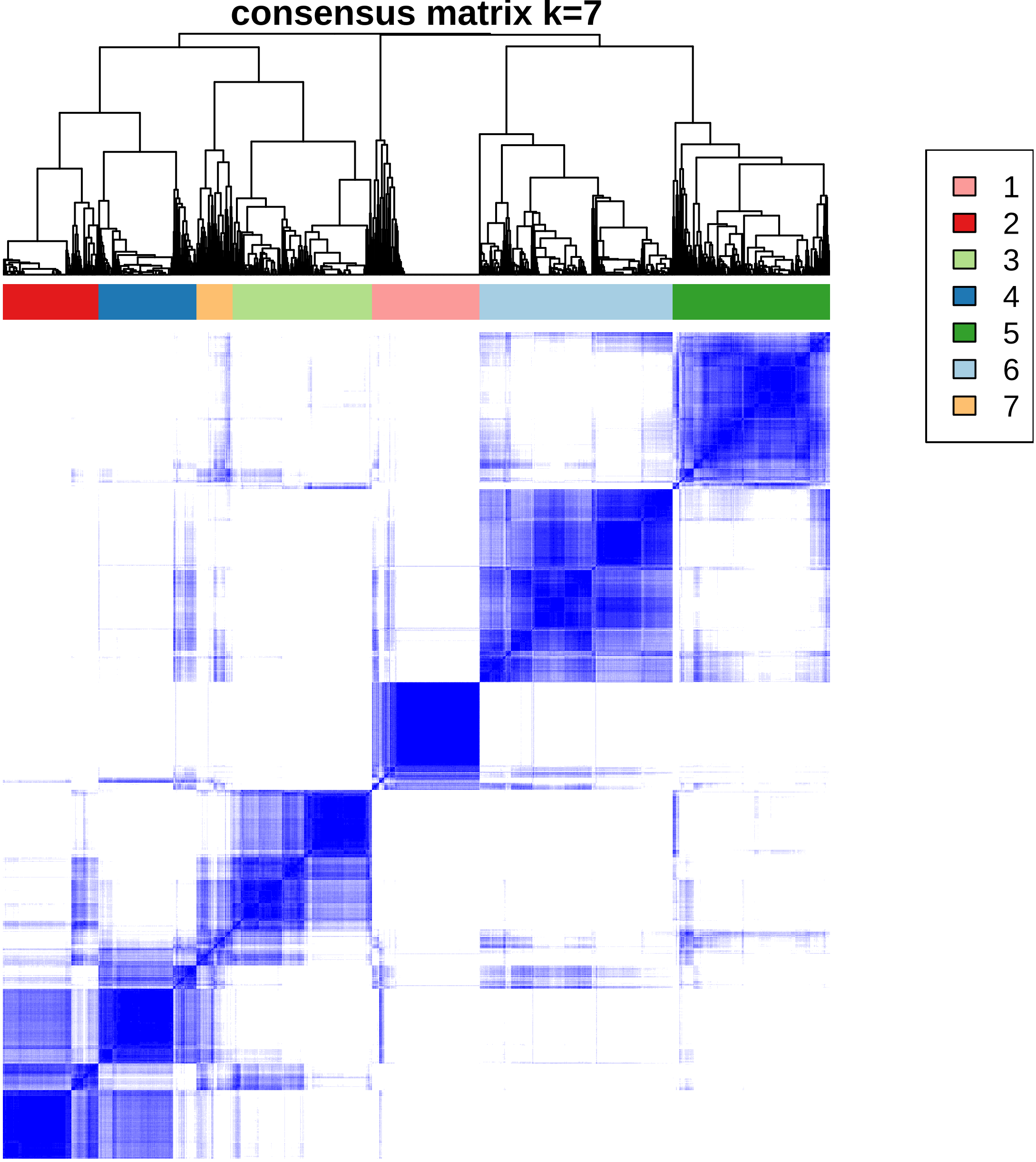
consensus matrix k=7
1
2
3
4
5
6
7

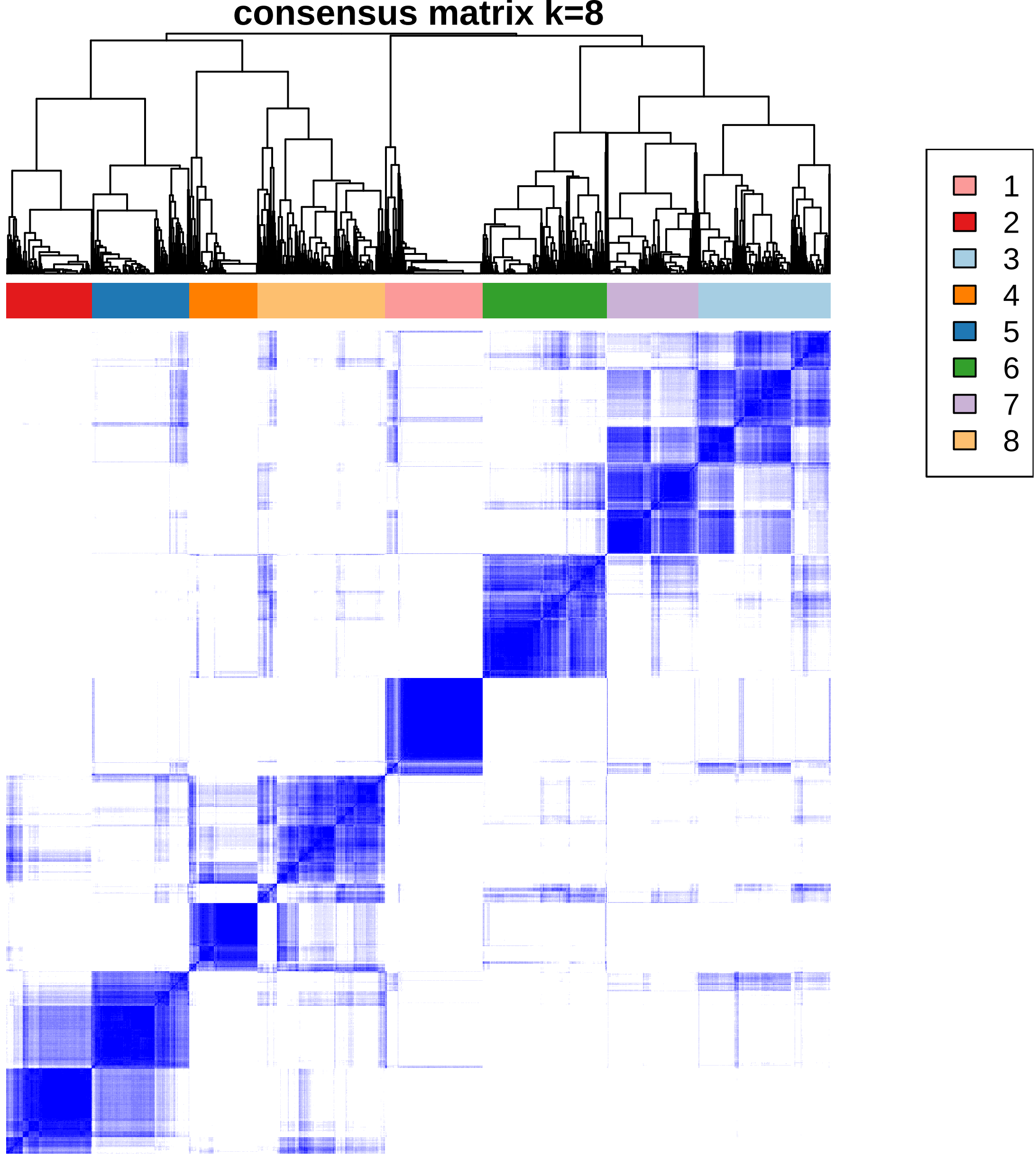
consensus matrix k=8
1
2
3
4
5
6
7
8

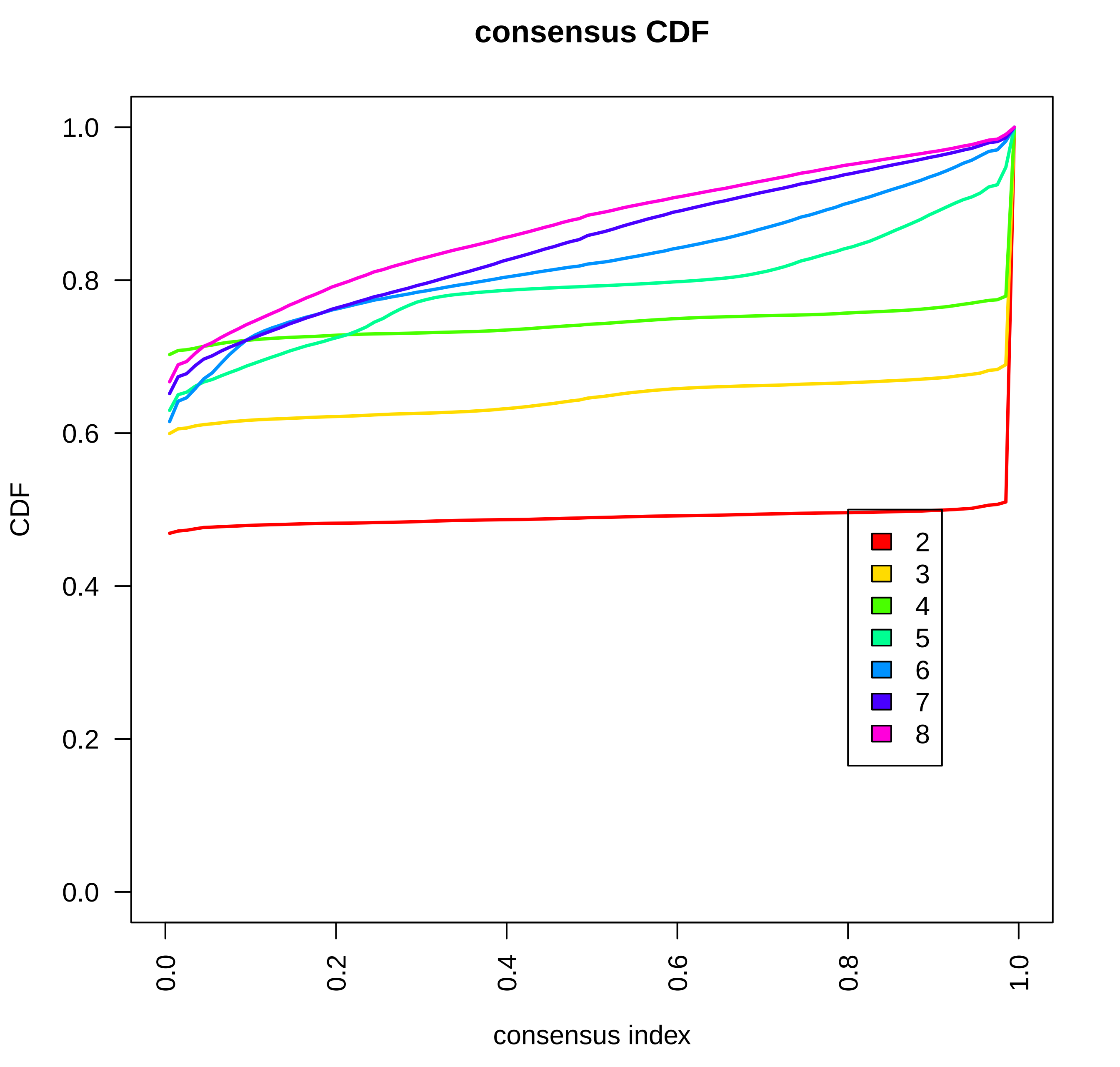
consensus CDF
CDF
consensus index
1.0
0.8
0.6
0.4
0.2
0.0
2
3
4
5
6
7
8

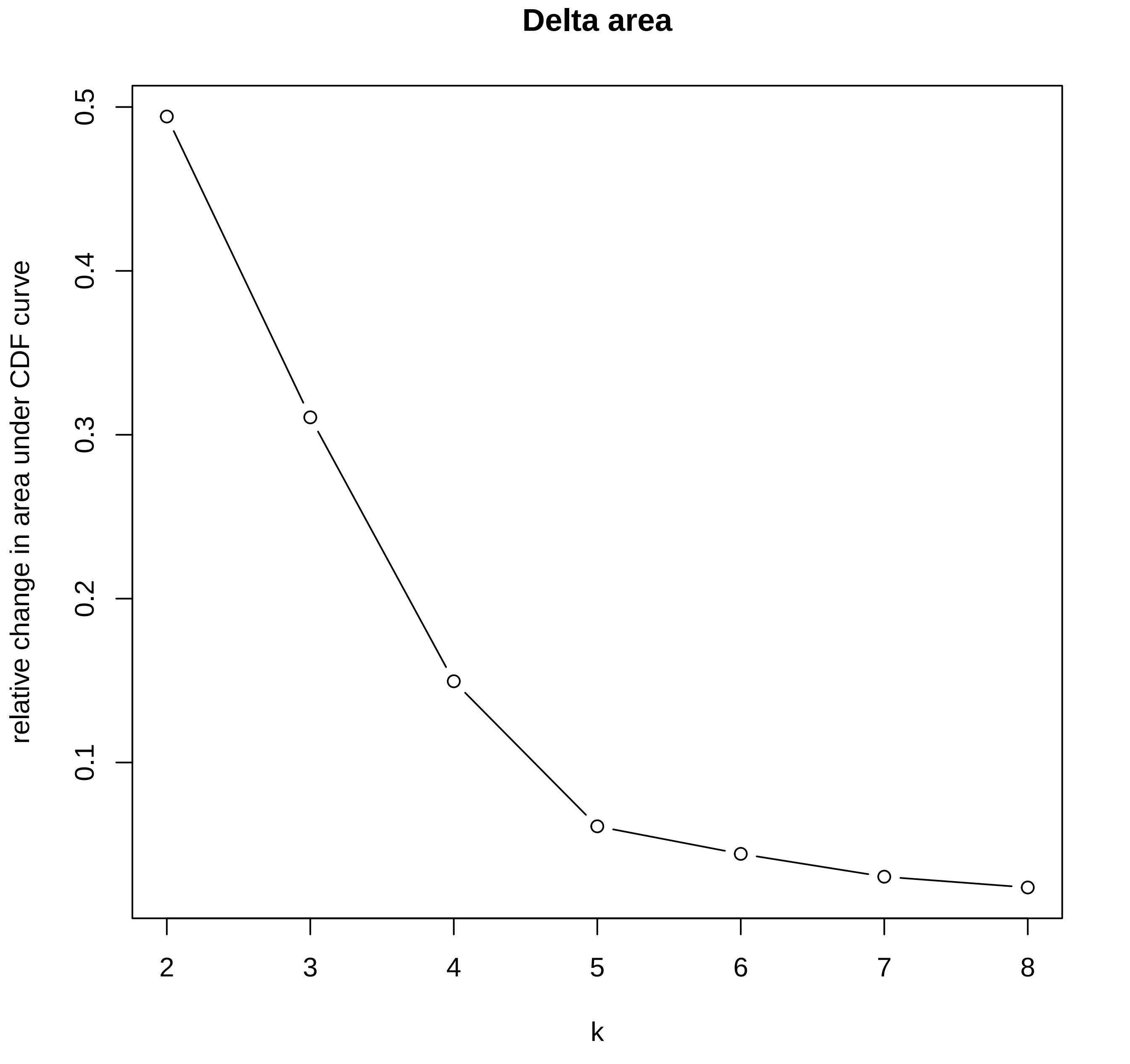
Delta area
relative change in area under CDF curve
0.1
0.2
0.3
0.4
0.5
k
2
3
4
5
6
7
8

tracking plot

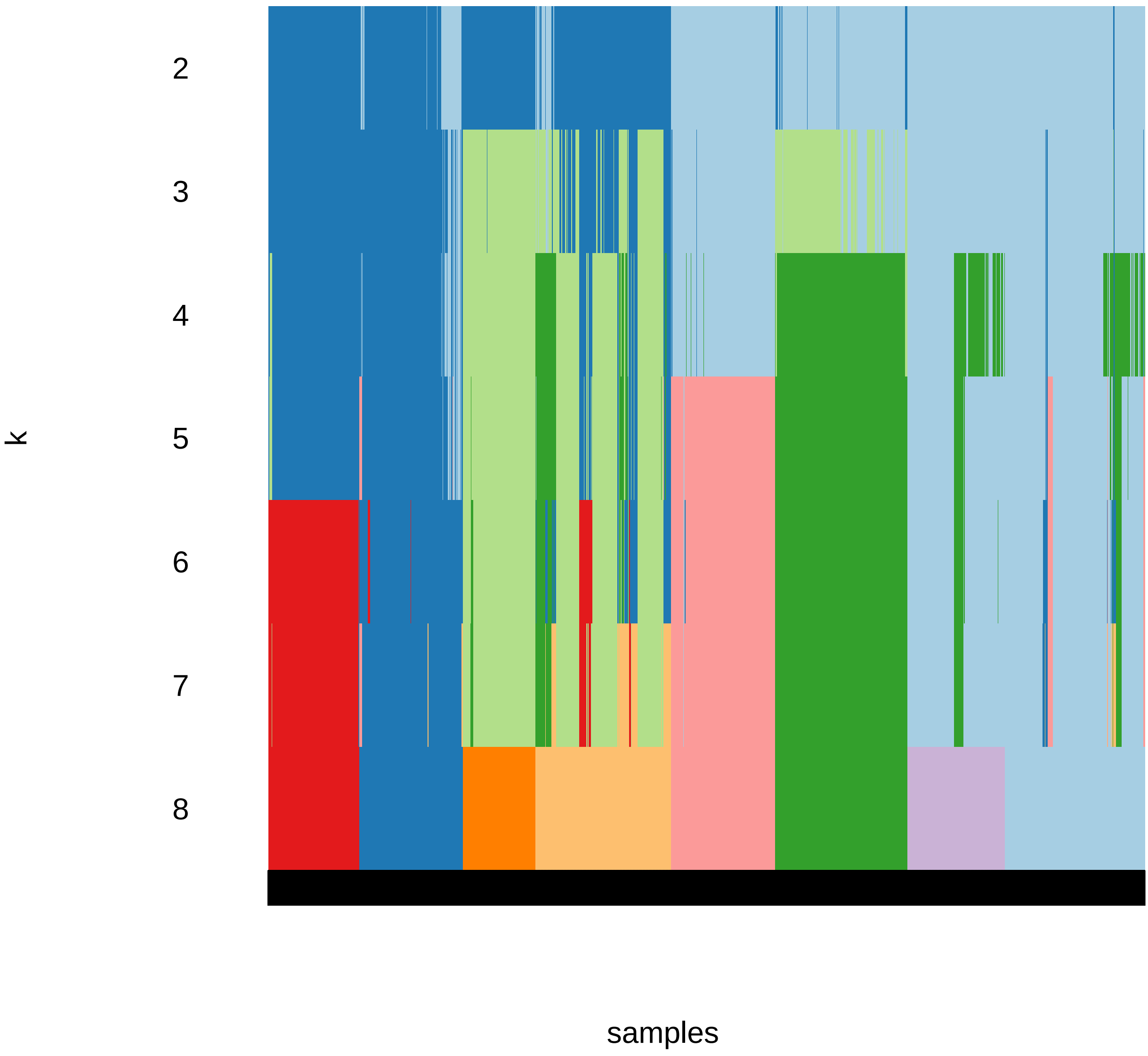

Supplement: Supplementary file 3 — Additional file3 (PDF 57226 KB) [file 12672_2026_5126_MOESM3_ESM.pdf]
