# Supplementary figures and images for: Integration of 117 machine learning algorithms and single-cell transcriptomics identifies macrophage polarization and ER stress signatures for cancer prognosis and precision therapy (part 2 of 2)
Source: Discov Oncol. 2026 Apr 30;17:917. doi: 10.1007/s12672-026-05126-6 (PMC13275951; doi:10.1007/s12672-026-05126-6)

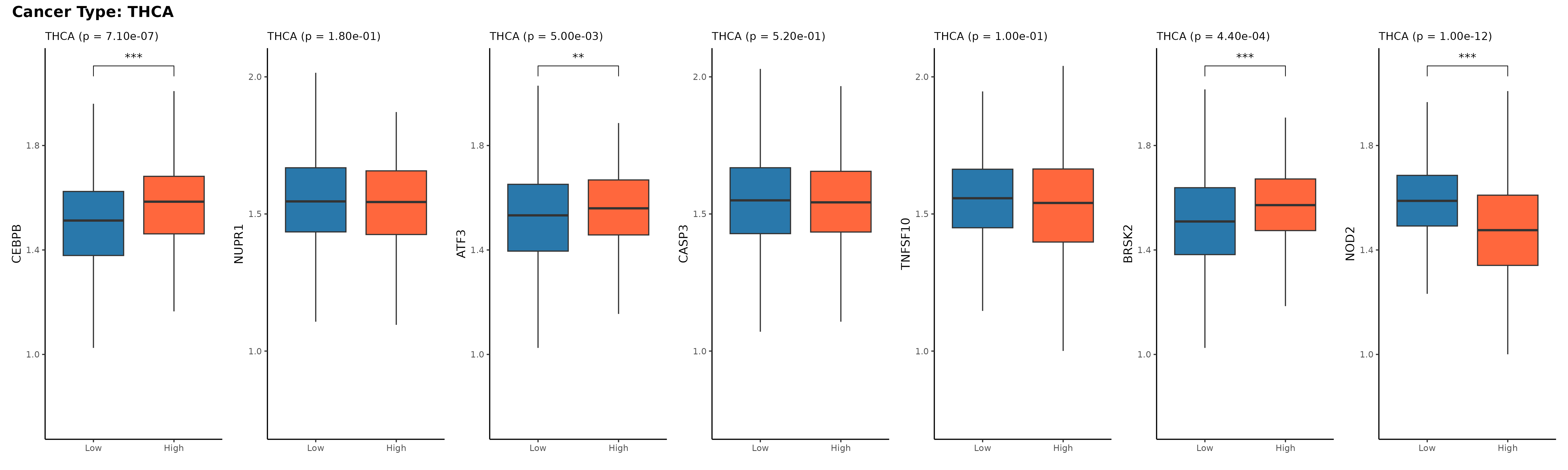

Supplement: Supplementary file 12 — Additional file12 (ZIP 3652 KB) [file 12672_2026_5126_MOESM12_ESM.zip › THCA_combined.png]

Cancer Type: THYM

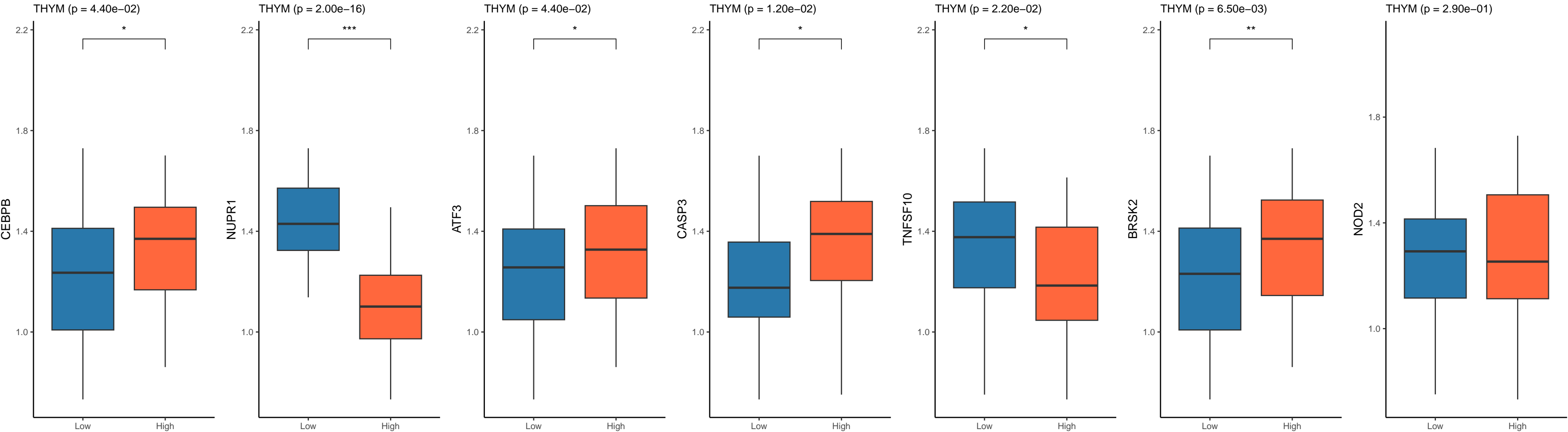

Supplement: Supplementary file 12 — Additional file12 (ZIP 3652 KB) [file 12672_2026_5126_MOESM12_ESM.zip › THYM_combined.pdf]

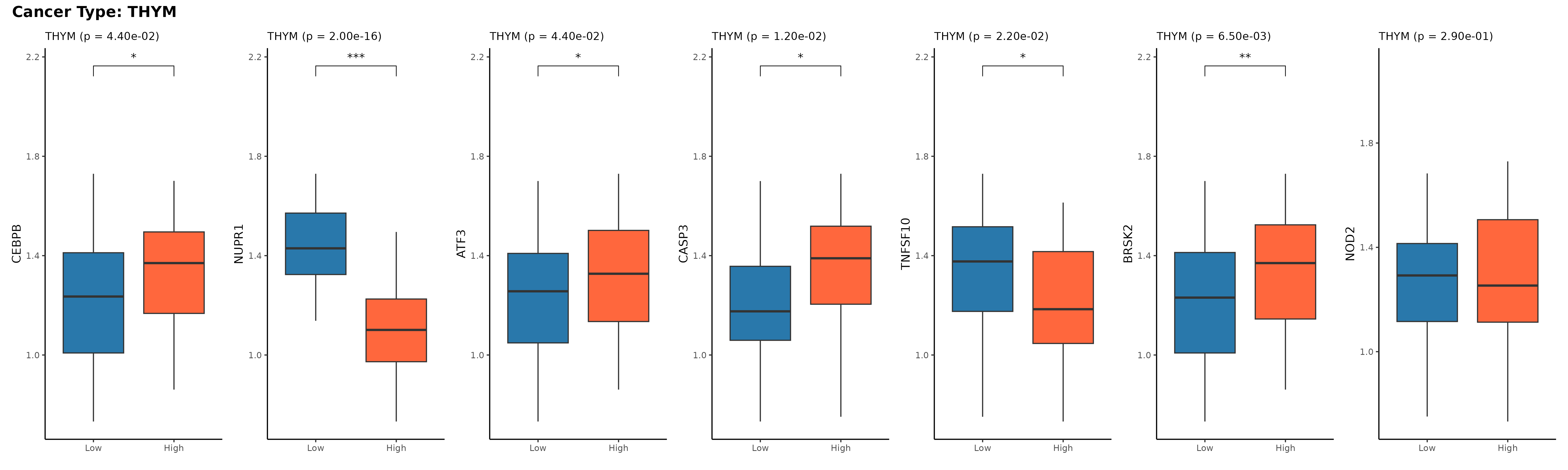

Supplement: Supplementary file 12 — Additional file12 (ZIP 3652 KB) [file 12672_2026_5126_MOESM12_ESM.zip › THYM_combined.png]

Cancer Type: UCEC

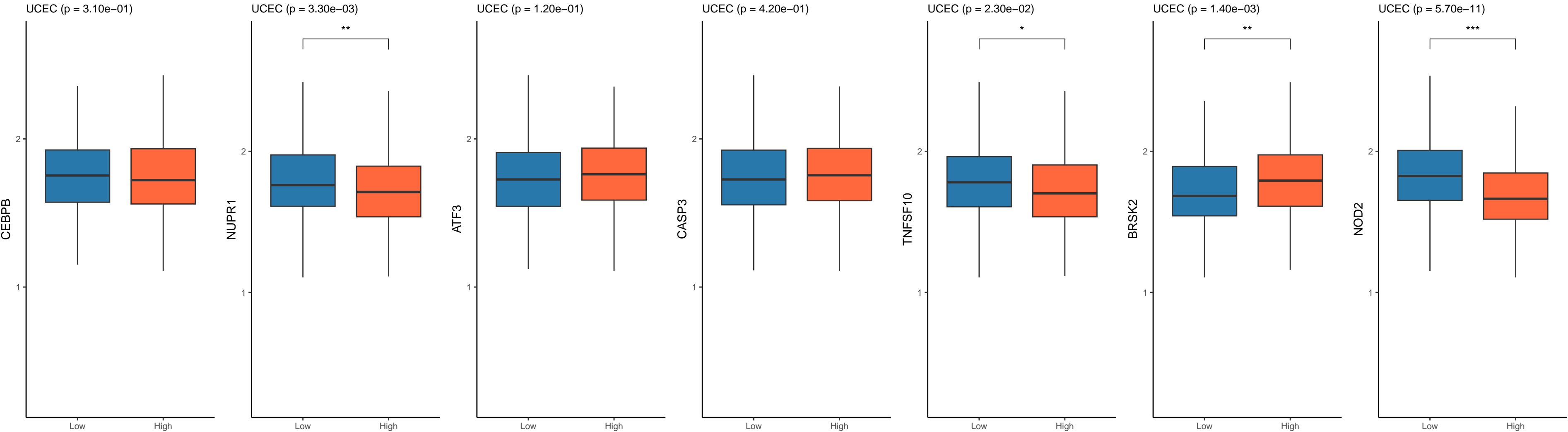

Supplement: Supplementary file 12 — Additional file12 (ZIP 3652 KB) [file 12672_2026_5126_MOESM12_ESM.zip › UCEC_combined.pdf]

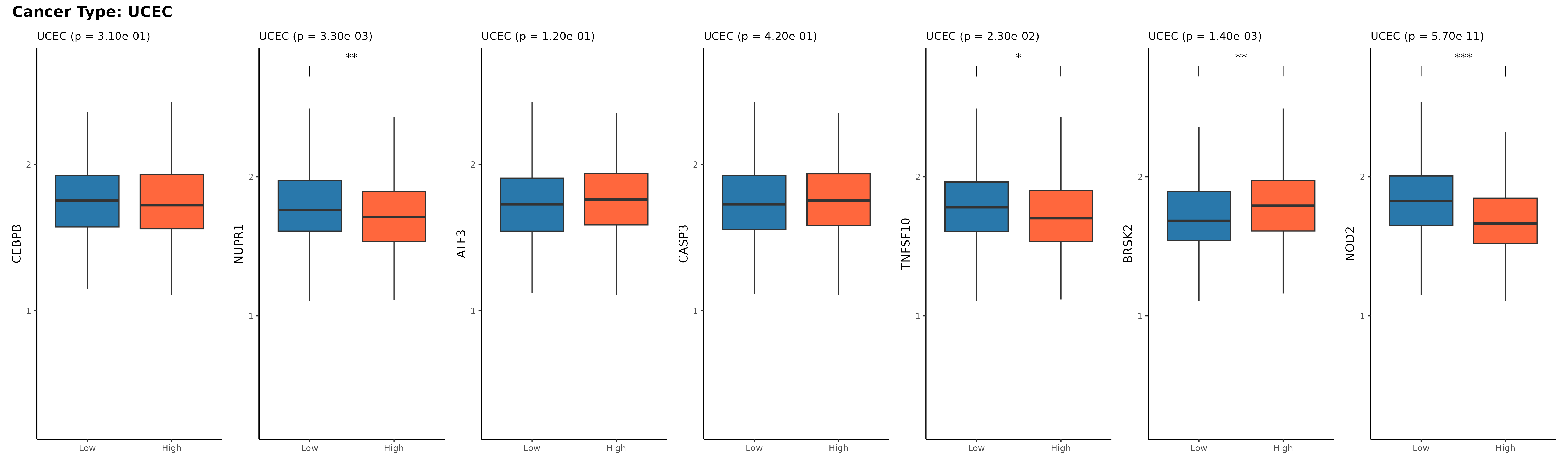

Supplement: Supplementary file 12 — Additional file12 (ZIP 3652 KB) [file 12672_2026_5126_MOESM12_ESM.zip › UCEC_combined.png]

Cancer Type: UCS

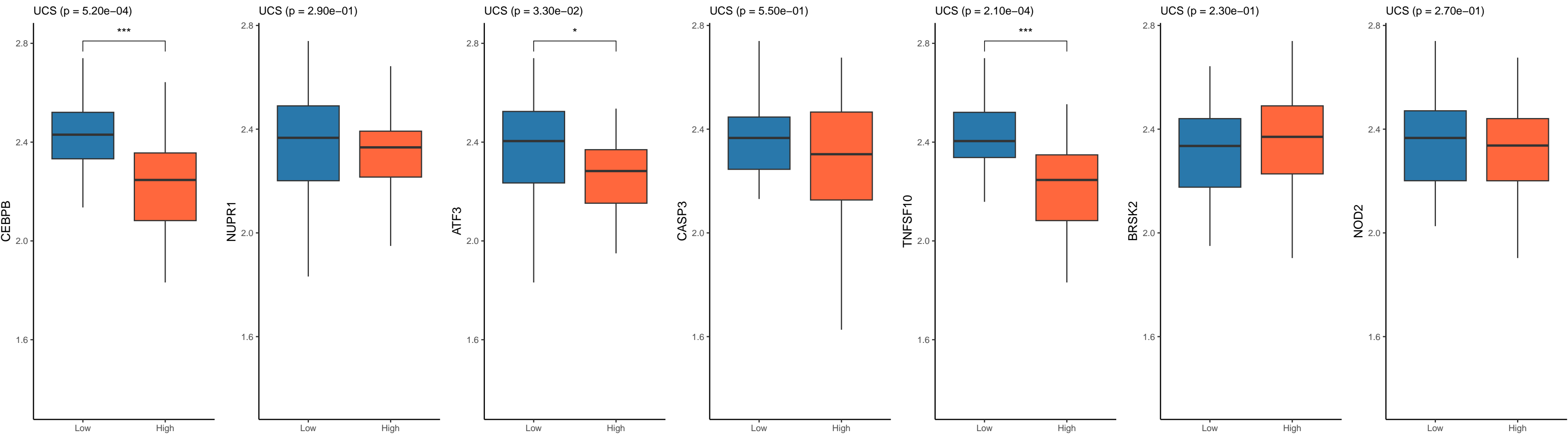

Supplement: Supplementary file 12 — Additional file12 (ZIP 3652 KB) [file 12672_2026_5126_MOESM12_ESM.zip › UCS_combined.pdf]

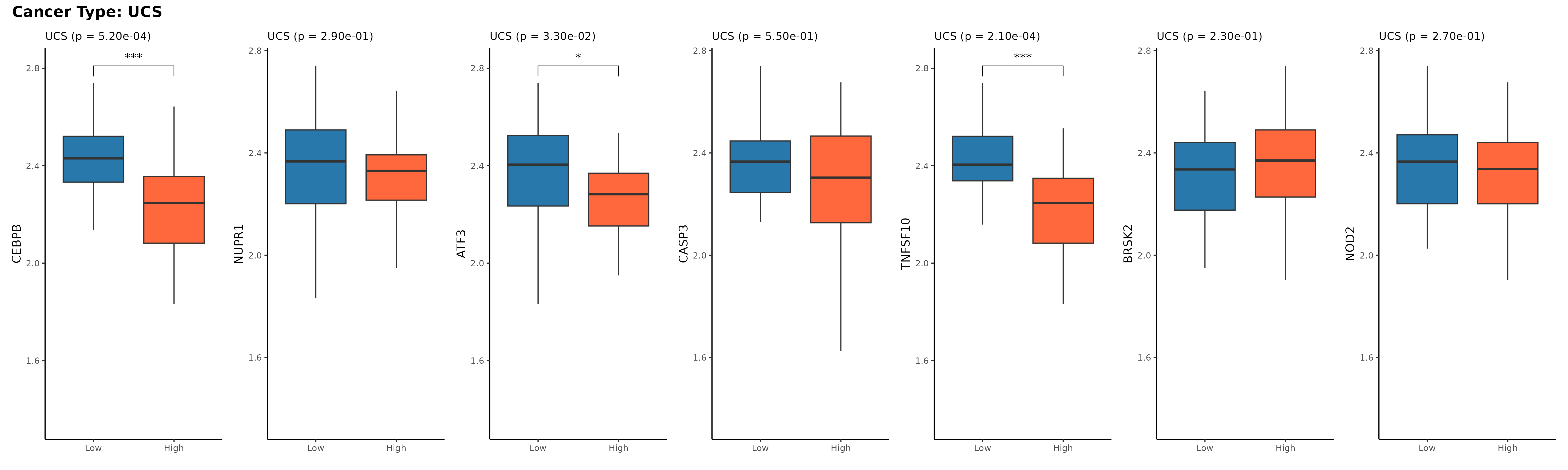

Supplement: Supplementary file 12 — Additional file12 (ZIP 3652 KB) [file 12672_2026_5126_MOESM12_ESM.zip › UCS_combined.png]

Cancer Type: UVM

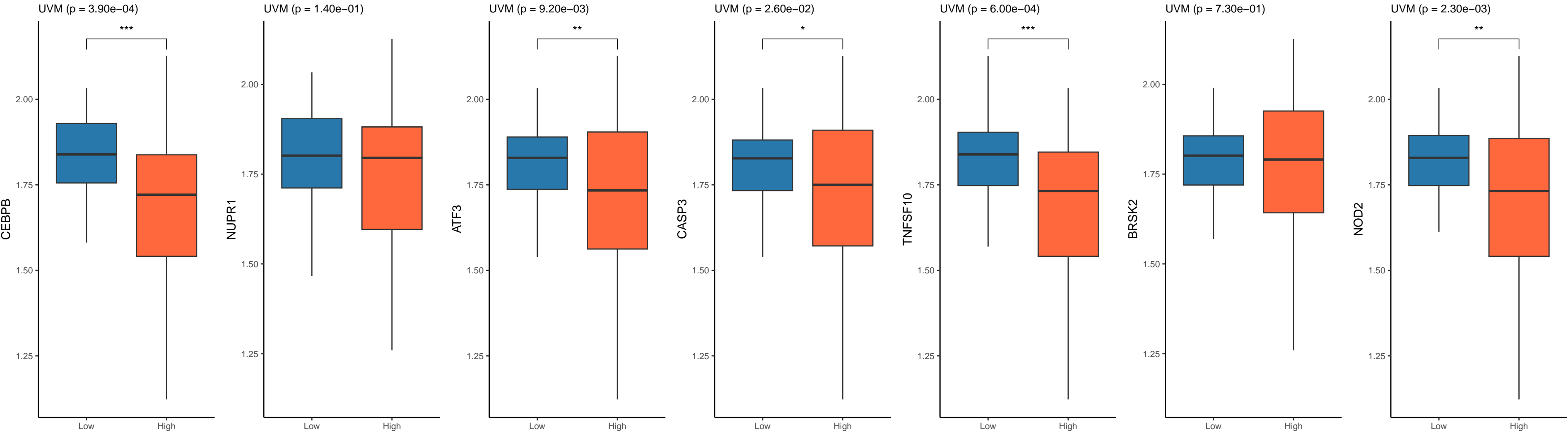

Supplement: Supplementary file 12 — Additional file12 (ZIP 3652 KB) [file 12672_2026_5126_MOESM12_ESM.zip › UVM_combined.pdf]

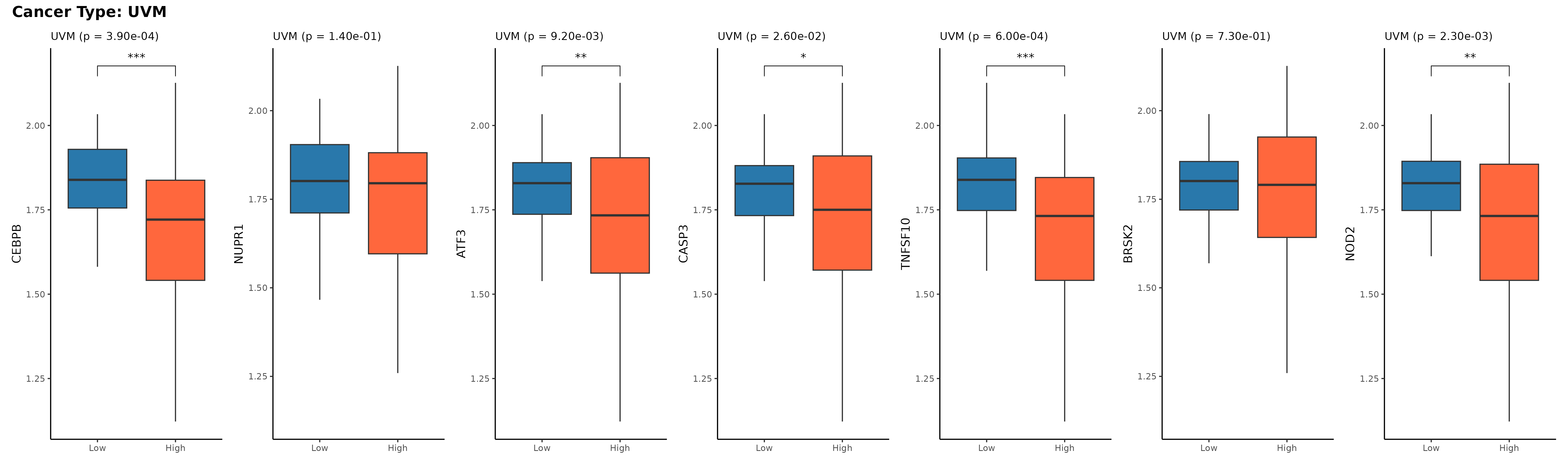

Supplement: Supplementary file 12 — Additional file12 (ZIP 3652 KB) [file 12672_2026_5126_MOESM12_ESM.zip › UVM_combined.png]
