# Supplementary material for: Integration of 117 machine learning algorithms and single-cell transcriptomics identifies macrophage polarization and ER stress signatures for cancer prognosis and precision therapy
Source: Discov Oncol. 2026 Apr 30;17:917. doi: 10.1007/s12672-026-05126-6 (PMC13275951; doi:10.1007/s12672-026-05126-6)
Supplement: Supplementary file 13 — Additional file13 (DOCX 18 KB) [file 12672_2026_5126_MOESM13_ESM.docx]

**Supplementary Methods**

**S1. Data Collection Parameters for MPERSRGs Identification**

From GeneCards, MPRGs were retained using filters: gene annotation = "protein coding" and relevance score > 3, yielding 7 MPRGs. ERSRGs were retained using: gene annotation = "protein coding" and relevance score > 5, yielding 145 ERSRGs. PubMed searches with "Macrophage Polarization" and "Endoplasmic Reticulum Stress" as keywords identified an additional 109 MPRGs and 14 ERSRGs from published literature, respectively. After merging and deduplication, 115 MPRGs (Table S2) and 153 ERSRGs (Table S3) were obtained.

**S2. Consensus Clustering Parameters**

*CCLE-based subgroup analysis (Section 2.2):* Consensus clustering was performed on the MPERSRGs mRNA expression matrix using the R package ConsensusClusterPlus. Parameters: maximum clusters K = 8; sample subsampling proportion = 80%; replicates = 100; clusterAlg = "km"; distance metric = "pearson". Cell lines were classified into two distinct subgroups.

*TCGA-based subgroup analysis (Section 2.10):* Consensus clustering was applied to the TCGA pan-cancer expression matrix. Parameters: maximum clusters K = 10; sample subsampling proportion = 80%; replicates = 100; clusterAlg = "km"; distance metric = "pearson". Samples were classified into Cluster1 and Cluster2.

**S3. GSEA Parameters**

For single-gene GSEA, samples were divided into high and low expression groups based on median expression of each MPERSRG. LogFC values between groups were calculated and genes were ranked in descending order. The R package clusterProfiler was used with the following parameters: seed = 2020; minimum gene set size = 10; maximum gene set size = 500. The c2 gene set (v2023.2.Hs.symbols.gmt) was obtained from MSigDB. Results were filtered at adj.p < 0.05 and FDR (q-value) < 0.25, with Benjamini-Hochberg (BH) correction for multiple testing.

**S4. GSVA Parameters**

GSVA was performed using the R package GSVA on the pan-cancer transcriptome expression matrix, utilizing the H.all.v7.4.symbols.gmt hallmark gene set from MSigDB. For fibroblast subtype analysis, the h.all.v2025.1.Hs.symbols.gmt gene set was used. The GSVA algorithm was applied with default parameters.

**S5. Machine Learning Algorithm Combinations (Supplementary Table S8)**

Ten classical algorithms were integrated: RSF, LASSO, GBM, Survival-SVM, SuperPC, Ridge Regression, plsRcox, CoxBoost, Stepwise Cox, and Elastic Net. Algorithms with inherent dimensionality reduction and variable selection capabilities (RSF, LASSO, CoxBoost, Stepwise Cox) were combined pairwise with the remaining algorithms, generating 117 unique algorithm combinations in total. C-indices for all 117 models across TCGA-LUAD, GSE19188, and GSE31210 cohorts are provided in Supplementary Table S8. The combination with the highest average C-index across all cohorts was selected as the optimal model.

**S6. Single-Cell RNA Sequencing Quality Control and Processing Parameters**

The Seurat object was created using "CreateSeuratObject" with the following minimum thresholds: minimum cells per gene = 3; minimum genes per cell = 200. Low-quality cells were filtered by removing cells with: UMI count < 500; RNA feature count < 250; log10GenesPerUMI < 0.8; mitochondrial gene content > 20%.

Normalization was performed using "NormalizeData" (method: "LogNormalize"). The top 2,000 hypervariable genes were identified using "FindVariableFeatures" (method: "vst"). Data scaling used "ScaleData" to eliminate sequencing depth effects. PCA was performed, and the top 30 significant principal components (selected by ElbowPlot) were used for UMAP dimensionality reduction via "RunUMAP". Clustering was performed using "FindNeighbors" (30 PCA dimensions, Euclidean distance) and "FindClusters" (resolution = 0.3, optimized by clustree).

**S7. CellChat Analysis Parameters**

Cell–cell communication was inferred using the R package CellChat, integrating single-cell expression profiles with the CellChatDB human ligand-receptor database. Significant ligand-receptor interaction pairs were identified through interaction probability computation and permutation testing. Communication networks were integrated by summing the number or strength of significant ligand-receptor pairs between cell types.

**S8. inferCNV Analysis Parameters**

The infercnv R package was used to identify CNVs by comparing expression patterns between tumor and reference cells. Count matrices for T lymphocytes, natural killer cells, myeloid cells, B lymphocytes, mast cells, and epithelial cells were extracted from the 22 LUAD samples in GSE131907. T lymphocytes, natural killer cells, myeloid cells, B lymphocytes, and mast cells were used as reference populations. The infercnv object was constructed using "CreateInfercnvObject". CNV analysis was run with parameters: cutoff = 0.1; k_nn = 100; denoise = TRUE. The entire epithelial cell cluster was analyzed at resolution = 0.3.
