# Supplementary material for: Integration of 117 machine learning algorithms and single-cell transcriptomics identifies macrophage polarization and ER stress signatures for cancer prognosis and precision therapy
Source: Discov Oncol. 2026 Apr 30;17:917. doi: 10.1007/s12672-026-05126-6 (PMC13275951; doi:10.1007/s12672-026-05126-6)
Supplement: Supplementary file 11 — Additional file11 (PDF 2684 KB) [file 12672_2026_5126_MOESM11_ESM.pdf]

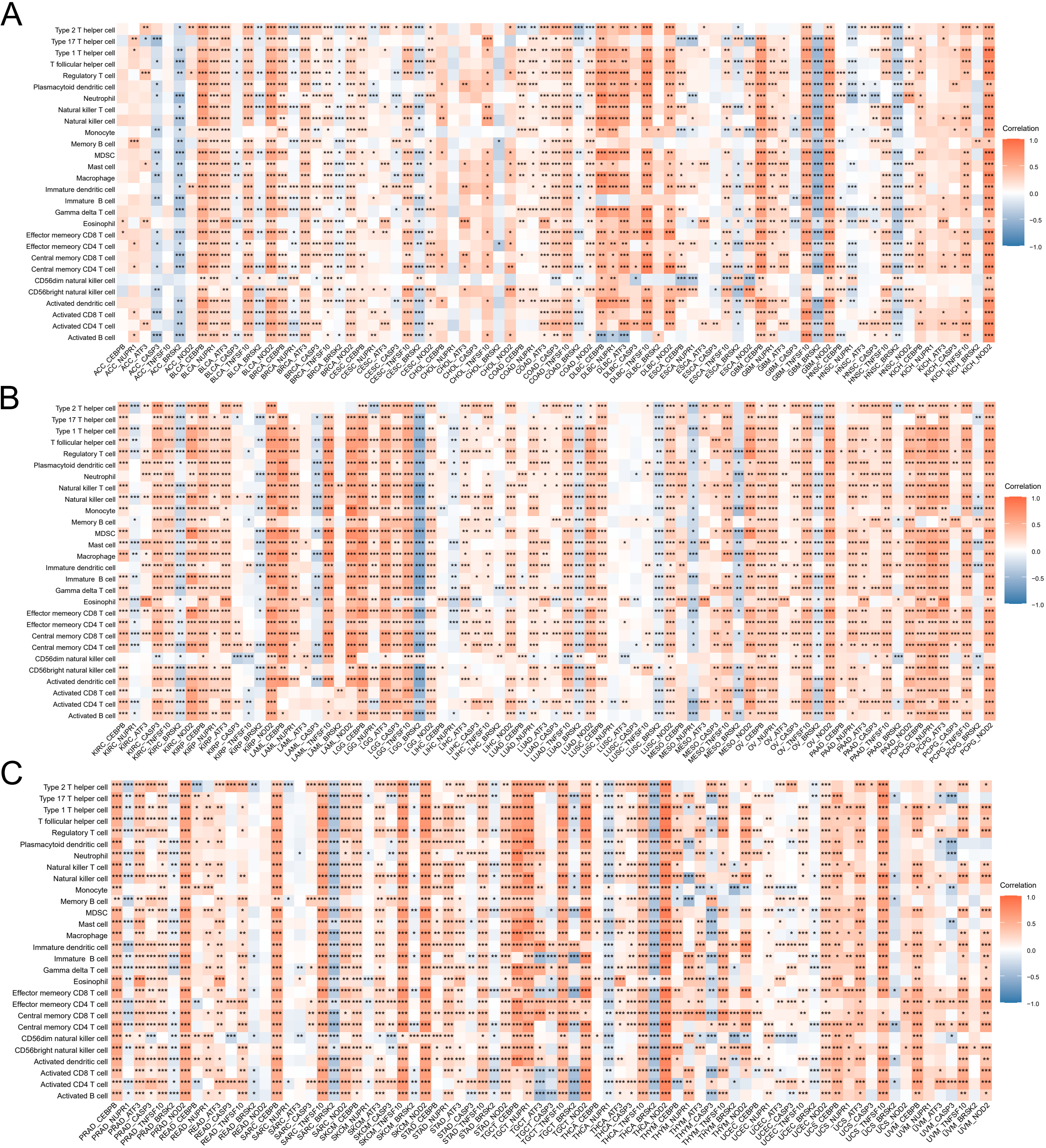
A
B
C
Type 2 T helper cell
Type 17 T helper cell
Type 1 T helper cell
T follicular helper cell
Regulatory T cell
Plasmacytoid dendritic cell
Neutrophil
Natural killer T cell
Natural killer cell
Monocyte
Memory B cell
MDSC
Mast cell
Macrophage
Immature dendritic cell
Immature B cell
Gamma delta T cell
Eosinophil
Effector memeory CD8 T cell
Effector memeory CD4 T cell
Central memory CD8 T cell
Central memory CD4 T cell
CD56dim natural killer cell
CD56bright natural killer cell
Activated dendritic cell
Activated CD8 T cell
Activated CD4 T cell
Activated B cell
Correlation
1.0
0.5
0.0
−0.5
−1.0
ACC_CEBPB ACC_NUPR1 ACC_ATF3 ACC_CASP3 ACC_TNFSF10 ACC_BRSK2 ACC_NOD2
BLCA_CEBPB BLCA_NUPR1 BLCA_ATF3 BLCA_CASP3 BLCA_TNFSF10 BLCA_BRSK2 BLCA_NOD2
BRCA_CEBPB BRCA_NUPR1 BRCA_ATF3 BRCA_CASP3 BRCA_TNFSF10 BRCA_BRSK2 BRCA_NOD2
CESC_CEBPB CESC_NUPR1 CESC_ATF3 CESC_CASP3 CESC_TNFSF10 CESC_BRSK2 CESC_NOD2
CHOL_CEBPB CHOL_NUPR1 CHOL_ATF3 CHOL_CASP3 CHOL_TNFSF10 CHOL_BRSK2 CHOL_NOD2
COAD_CEBPB COAD_NUPR1 COAD_ATF3 COAD_CASP3 COAD_TNFSF10 COAD_BRSK2 COAD_NOD2
DLBC_CEBPB DLBC_NUPR1 DLBC_ATF3 DLBC_CASP3 DLBC_TNFSF10 DLBC_BRSK2 DLBC_NOD2
ESCA_CEBPB ESCA_NUPR1 ESCA_ATF3 ESCA_CASP3 ESCA_TNFSF10 ESCA_BRSK2 ESCA_NOD2
GBM_CEBPB GBM_NUPR1 GBM_ATF3 GBM_CASP3 GBM_TNFSF10 GBM_BRSK2 GBM_NOD2
HNSC_CEBPB HNSC_NUPR1 HNSC_ATF3 HNSC_CASP3 HNSC_TNFSF10 HNSC_BRSK2 HNSC_NOD2
KICH_CEBPB KICH_NUPR1 KICH_ATF3 KICH_CASP3 KICH_TNFSF10 KICH_BRSK2 KICH_NOD2
KIRC_CEBPB KIRC_NUPR1 KIRC_ATF3 KIRC_CASP3 KIRC_TNFSF10 KIRC_BRSK2 KIRC_NOD2
KIRP_CEBPB KIRP_NUPR1 KIRP_ATF3 KIRP_CASP3 KIRP_TNFSF10 KIRP_BRSK2 KIRP_NOD2
LAML_CEBPB LAML_NUPR1 LAML_ATF3 LAML_CASP3 LAML_TNFSF10 LAML_BRSK2 LAML_NOD2
LGG_CEBPB LGG_NUPR1 LGG_ATF3 LGG_CASP3 LGG_TNFSF10 LGG_BRSK2 LGG_NOD2
LIHC_CEBPB LIHC_NUPR1 LIHC_ATF3 LIHC_CASP3 LIHC_TNFSF10 LIHC_BRSK2 LIHC_NOD2
LUAD_CEBPB LUAD_NUPR1 LUAD_ATF3 LUAD_CASP3 LUAD_TNFSF10 LUAD_BRSK2 LUAD_NOD2
LUSC_CEBPB LUSC_NUPR1 LUSC_ATF3 LUSC_CASP3 LUSC_TNFSF10 LUSC_BRSK2 LUSC_NOD2
MESO_CEBPB MESO_NUPR1 MESO_ATF3 MESO_CASP3 MESO_TNFSF10 MESO_BRSK2 MESO_NOD2
OV_CEBPB OV_NUPR1 OV_ATF3 OV_CASP3 OV_TNFSF10 OV_BRSK2 OV_NOD2
PAAD_CEBPB PAAD_NUPR1 PAAD_ATF3 PAAD_CASP3 PAAD_TNFSF10 PAAD_BRSK2 PAAD_NOD2
PCPG_CEBPB PCPG_NUPR1 PCPG_ATF3 PCPG_CASP3 PCPG_TNFSF10 PCPG_BRSK2 PCPG_NOD2
PRAD_CEBPB PRAD_NUPR1 PRAD_ATF3 PRAD_CASP3 PRAD_TNFSF10 PRAD_BRSK2 PRAD_NOD2
READ_CEBPB READ_NUPR1 READ_ATF3 READ_CASP3 READ_TNFSF10 READ_BRSK2 READ_NOD2
SARC_CEBPB SARC_NUPR1 SARC_ATF3 SARC_CASP3 SARC_TNFSF10 SARC_BRSK2 SARC_NOD2
SKCM_CEBPB SKCM_NUPR1 SKCM_ATF3 SKCM_CASP3 SKCM_TNFSF10 SKCM_BRSK2 SKCM_NOD2
STAD_CEBPB STAD_NUPR1 STAD_ATF3 STAD_CASP3 STAD_TNFSF10 STAD_BRSK2 STAD_NOD2
TGCT_CEBPB TGCT_NUPR1 TGCT_ATF3 TGCT_CASP3 TGCT_TNFSF10 TGCT_BRSK2 TGCT_NOD2
THCA_CEBPB THCA_NUPR1 THCA_ATF3 THCA_CASP3 THCA_TNFSF10 THCA_BRSK2 THCA_NOD2
THYM_CEBPB THYM_NUPR1 THYM_ATF3 THYM_CASP3 THYM_TNFSF10 THYM_BRSK2 THYM_NOD2
UCEC_CEBPB UCEC_NUPR1 UCEC_ATF3 UCEC_CASP3 UCEC_TNFSF10 UCEC_BRSK2 UCEC_NOD2
UCS_CEBPB UCS_NUPR1 UCS_ATF3 UCS_CASP3 UCS_TNFSF10 UCS_BRSK2 UCS_NOD2
UVM_CEBPB UVM_NUPR1 UVM_ATF3 UVM_CASP3 UVM_TNFSF10 UVM_BRSK2 UVM_NOD2
